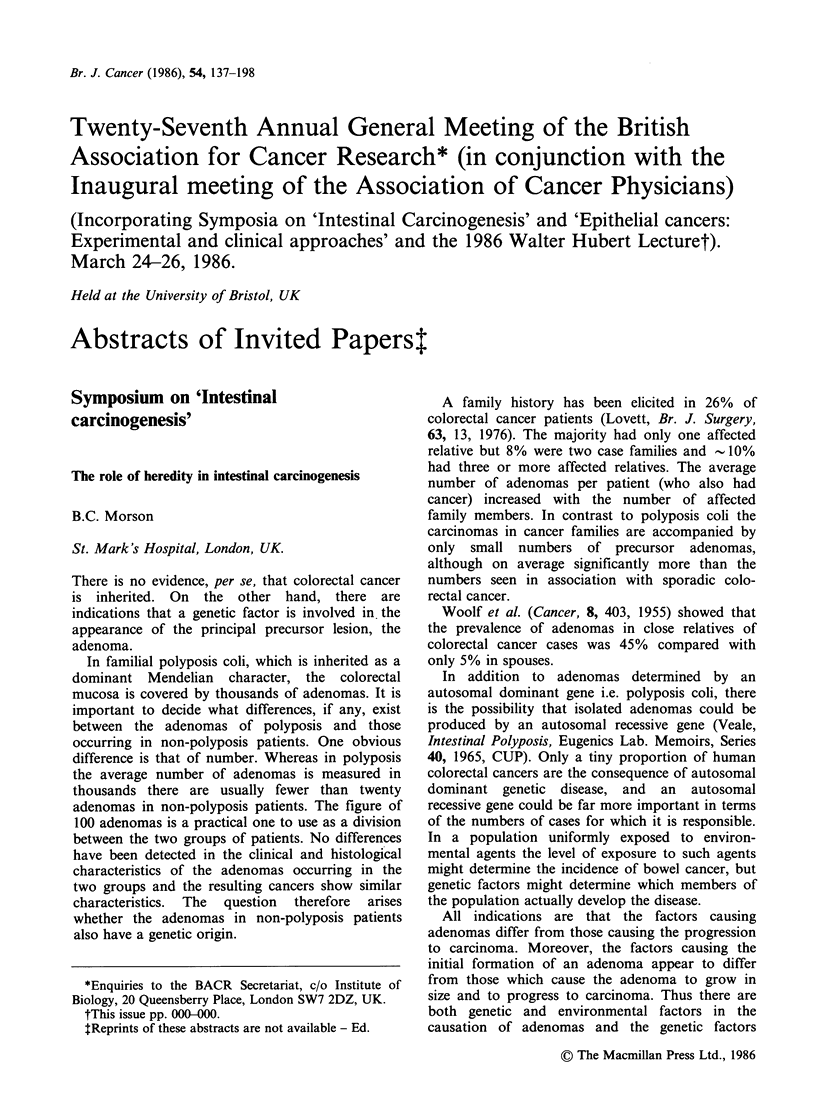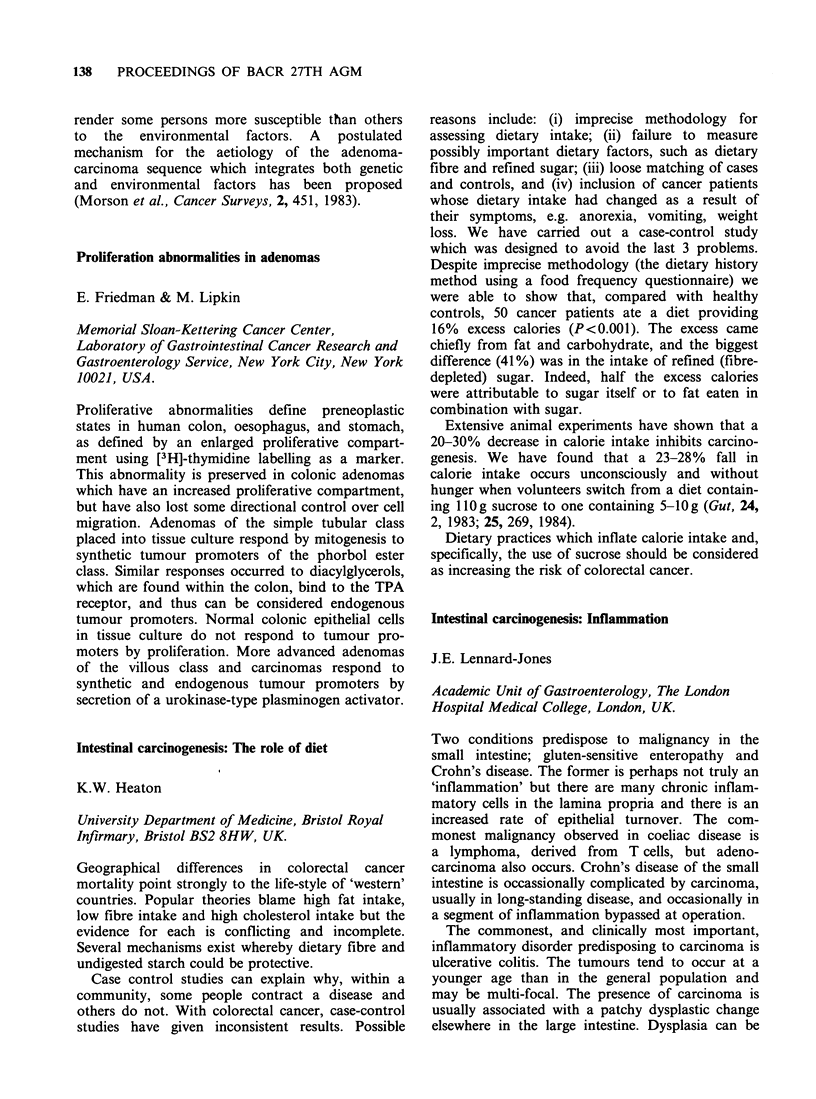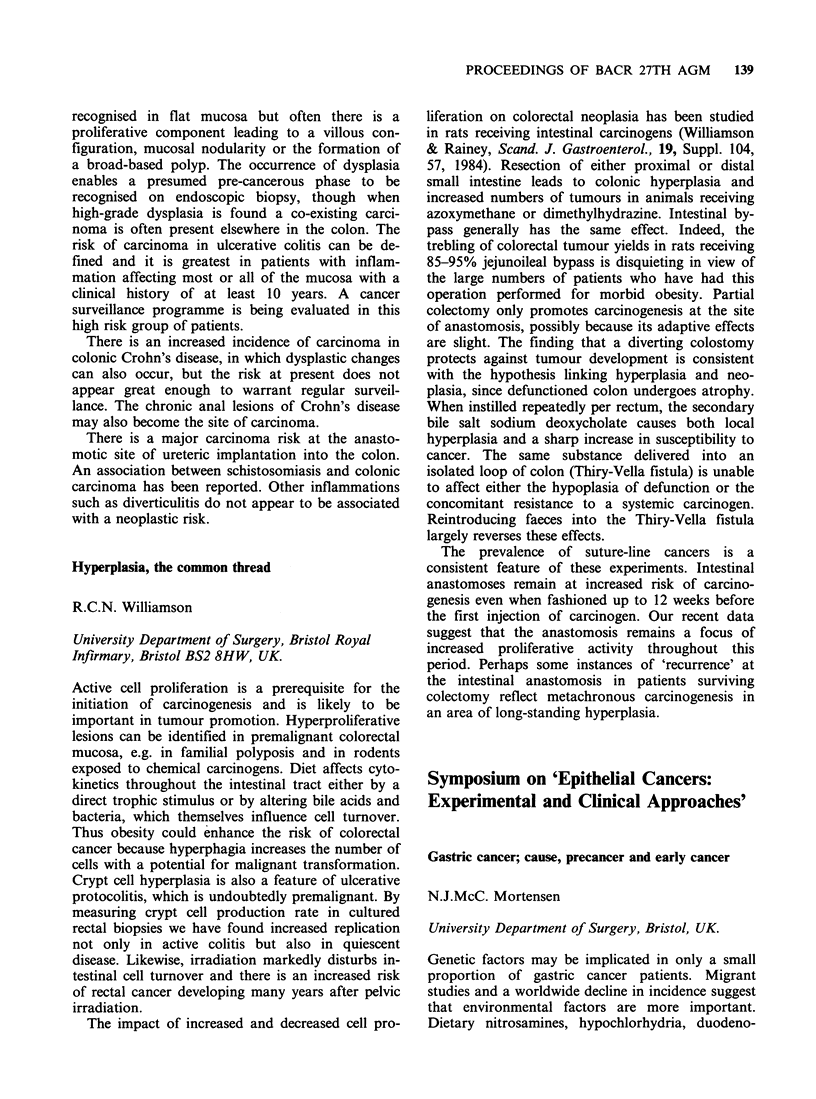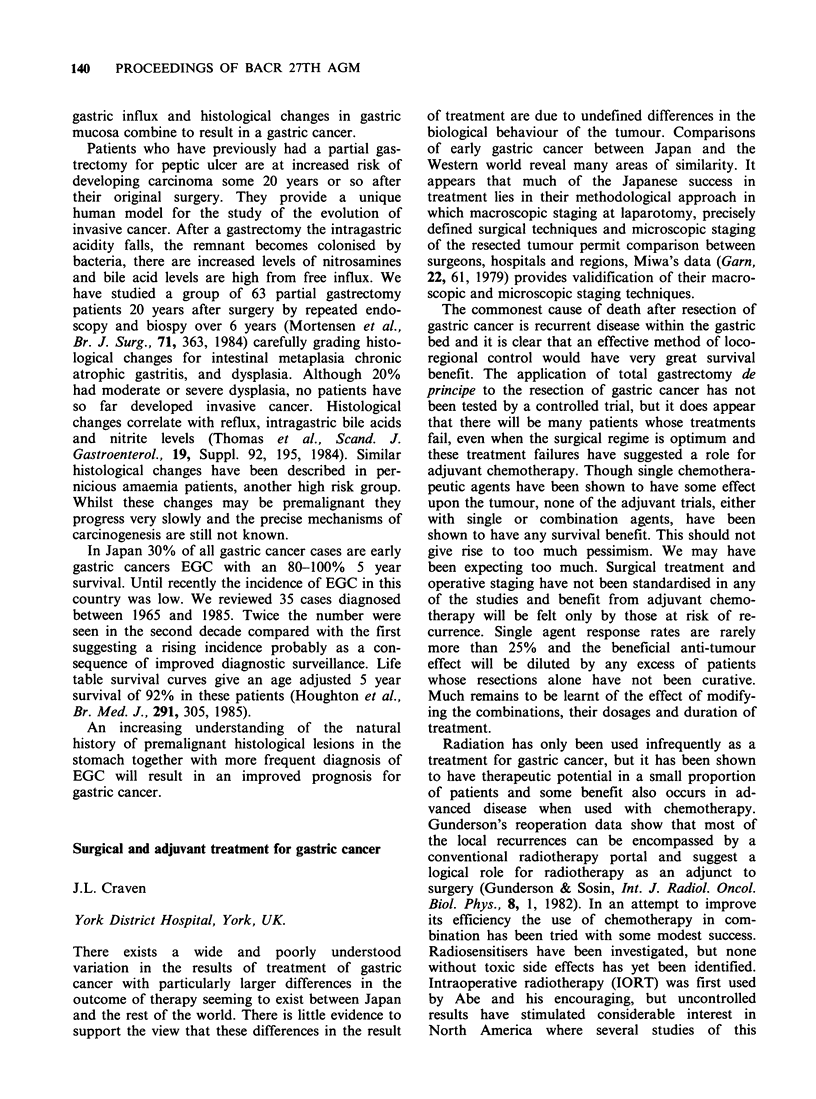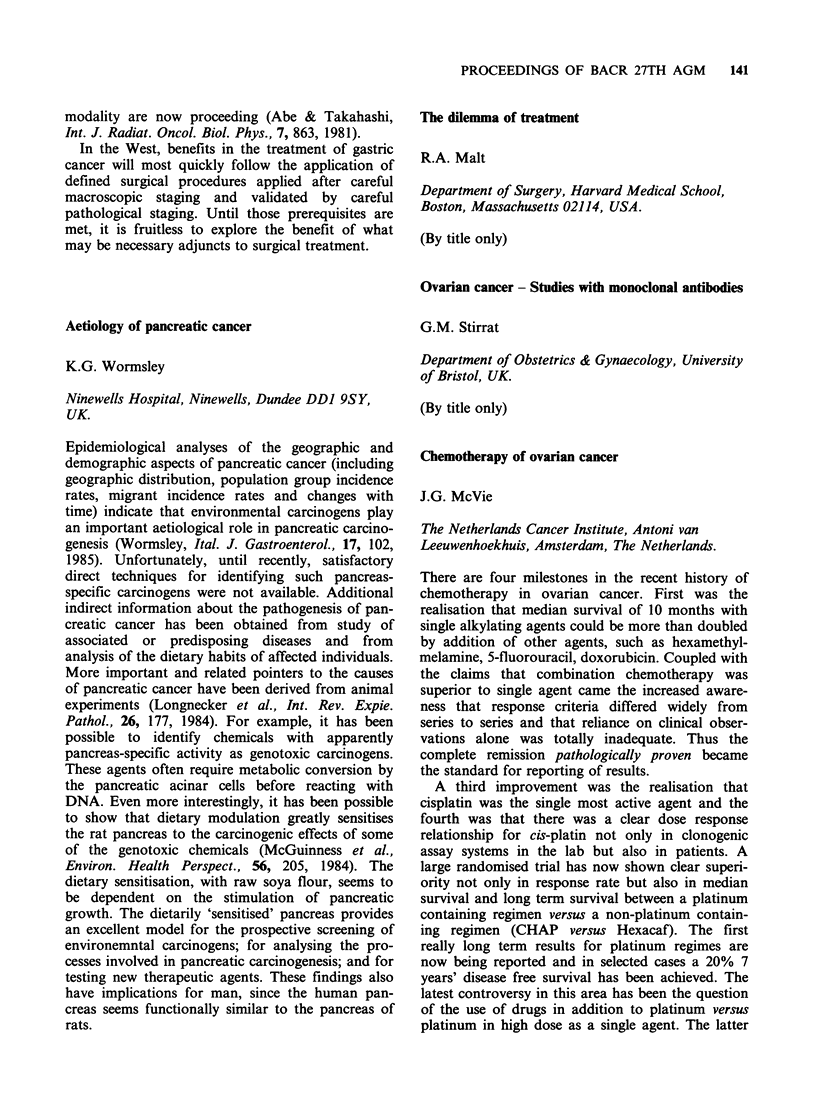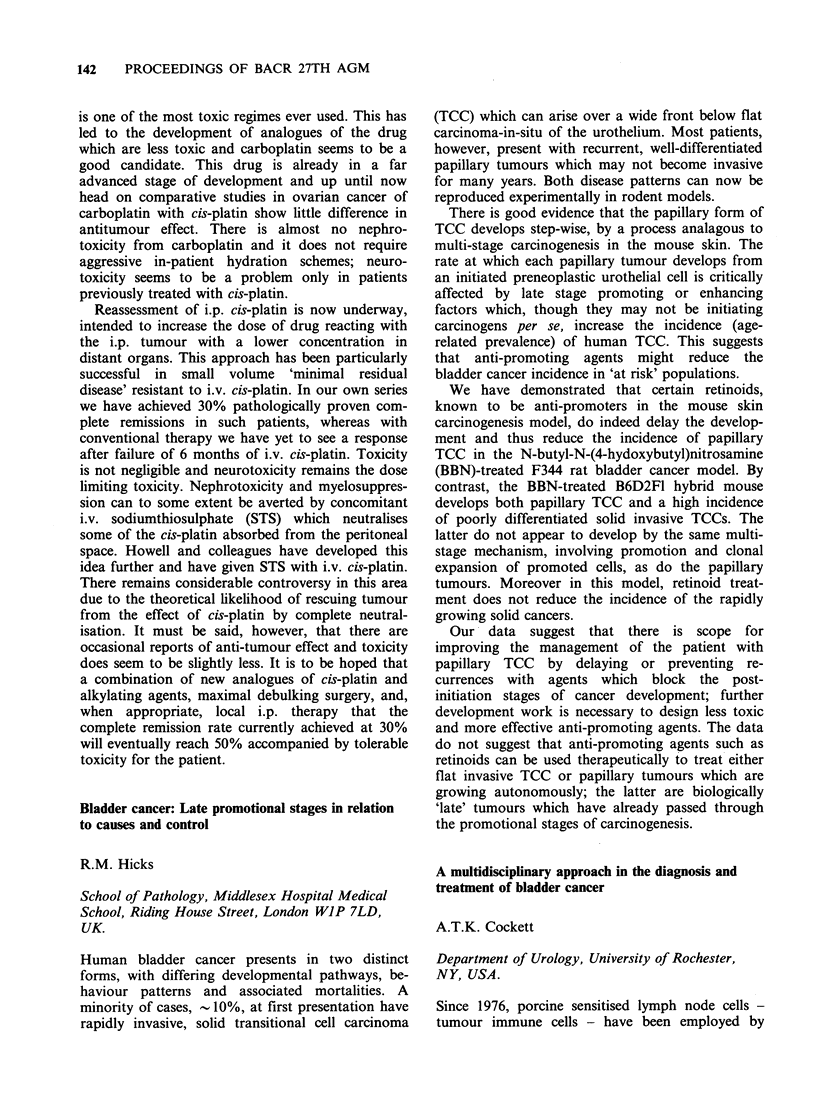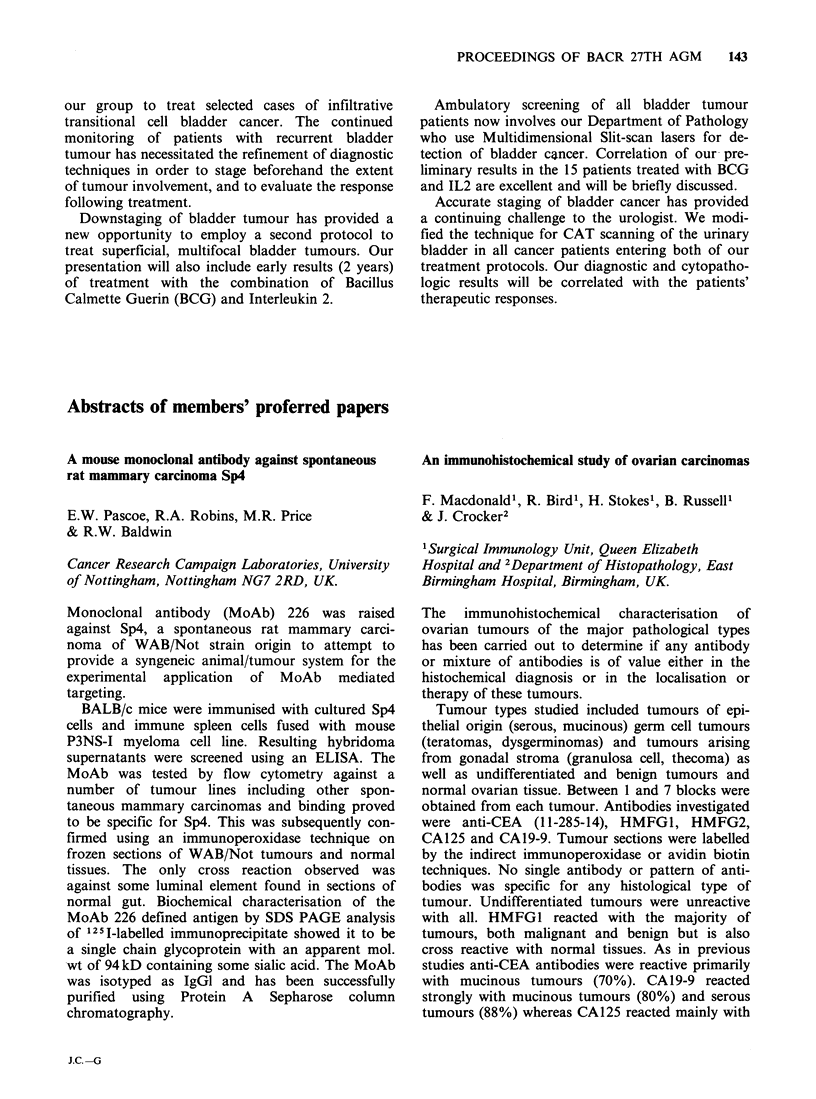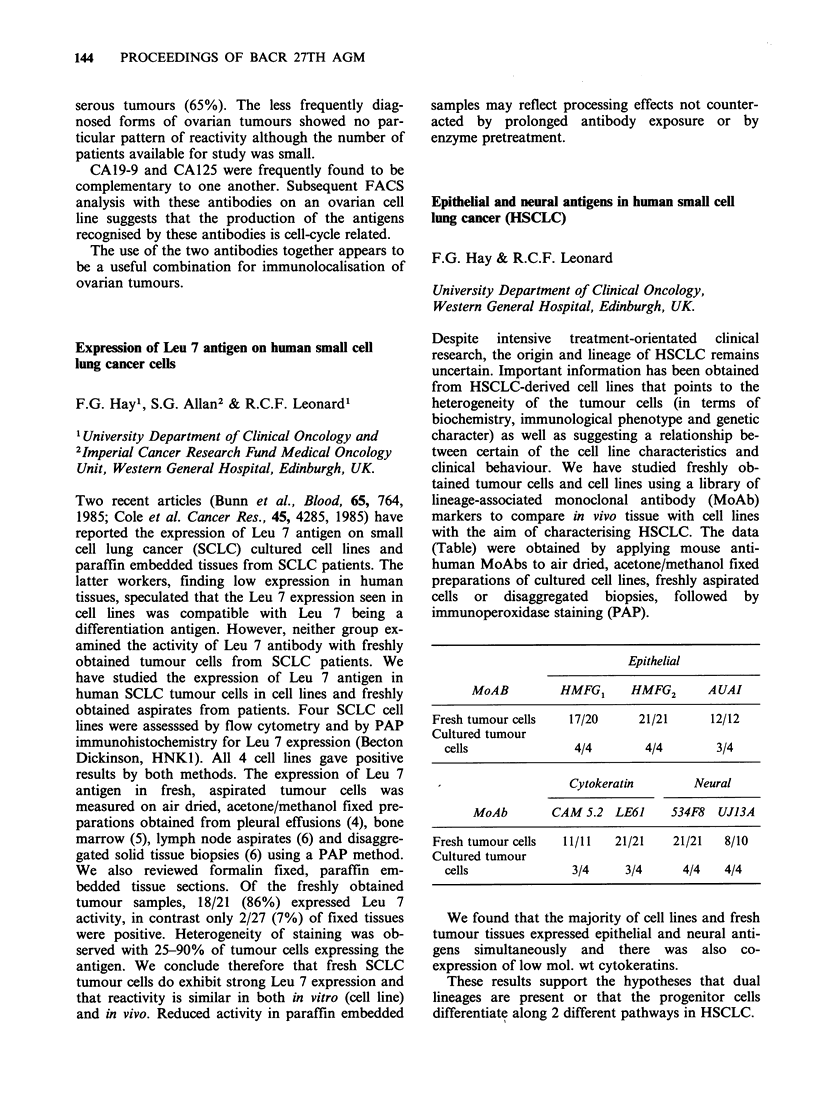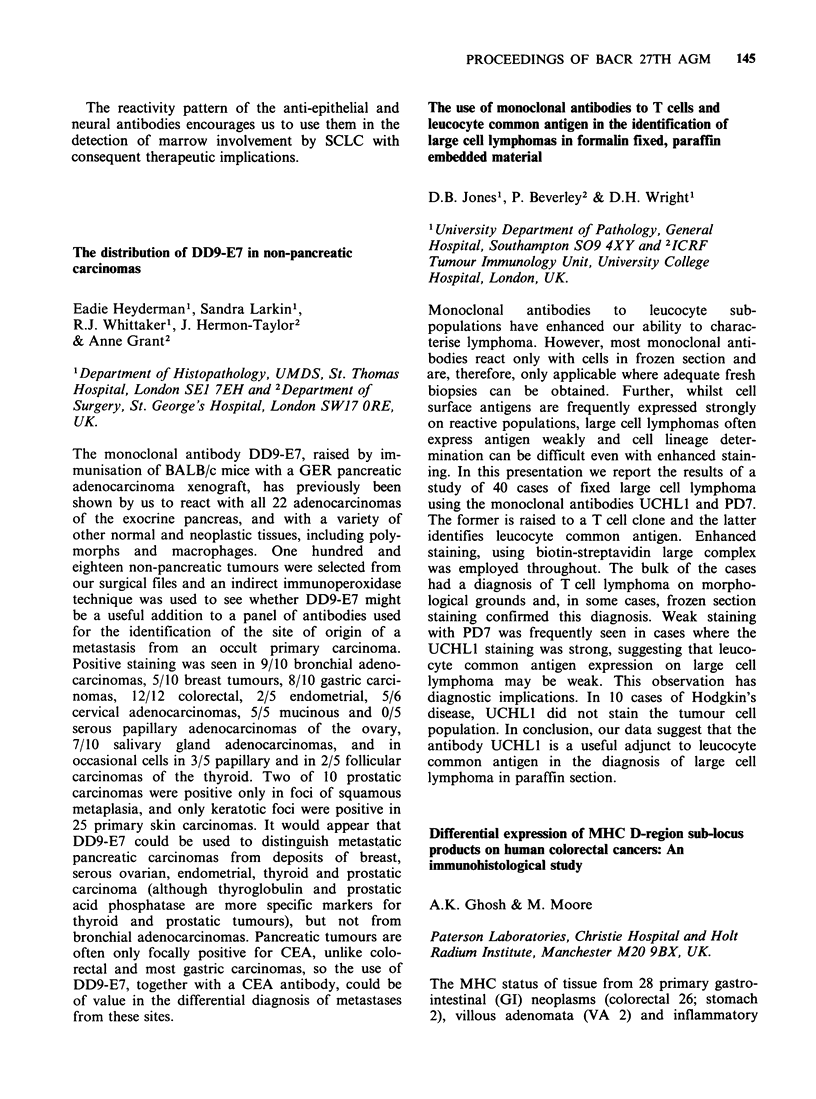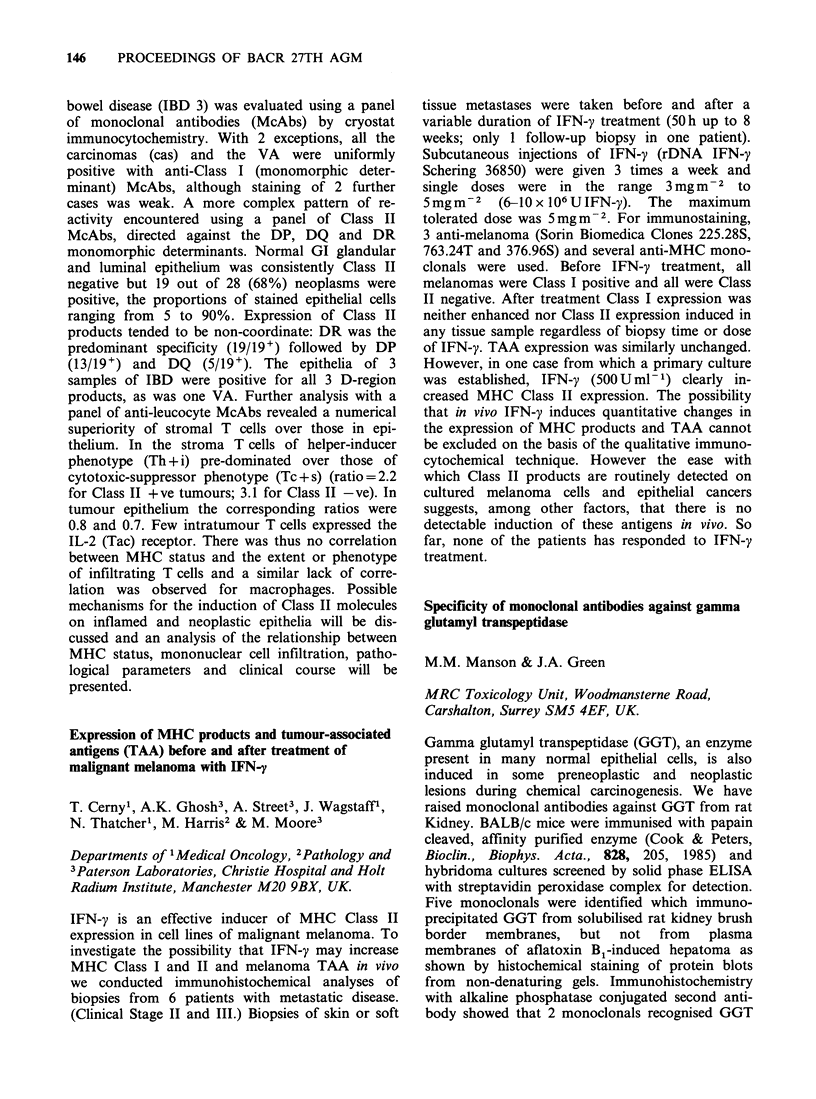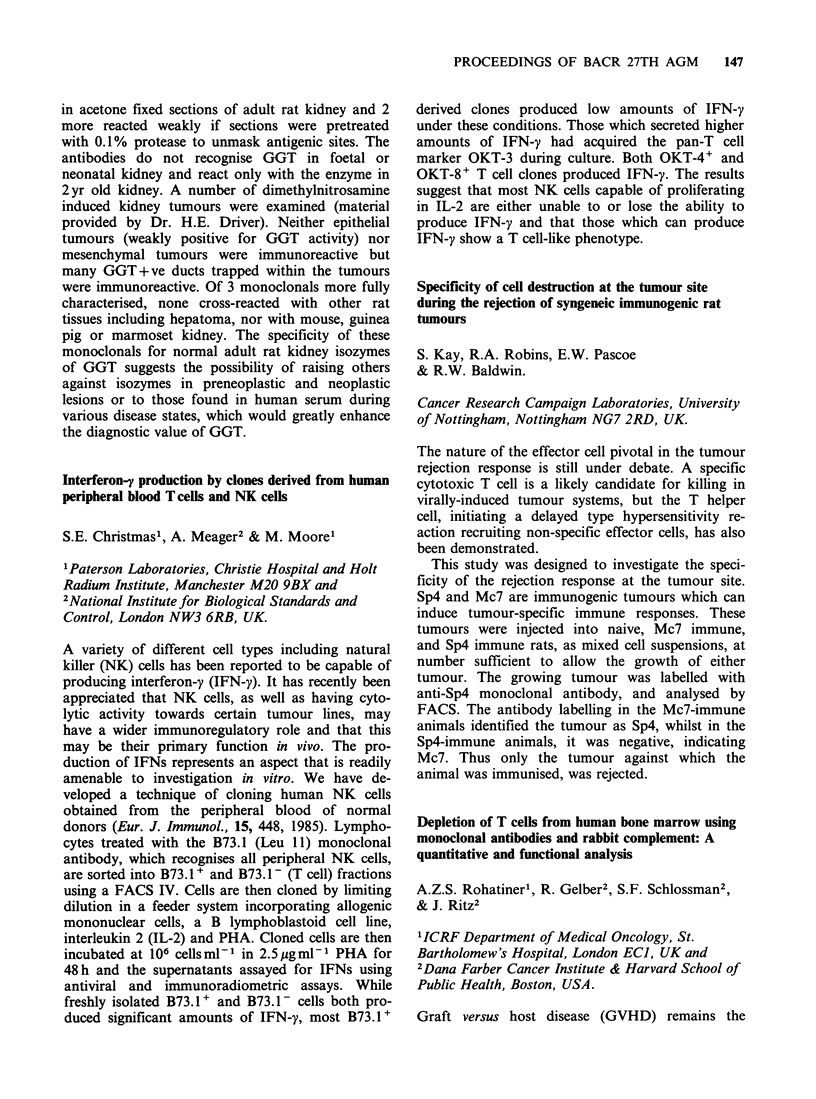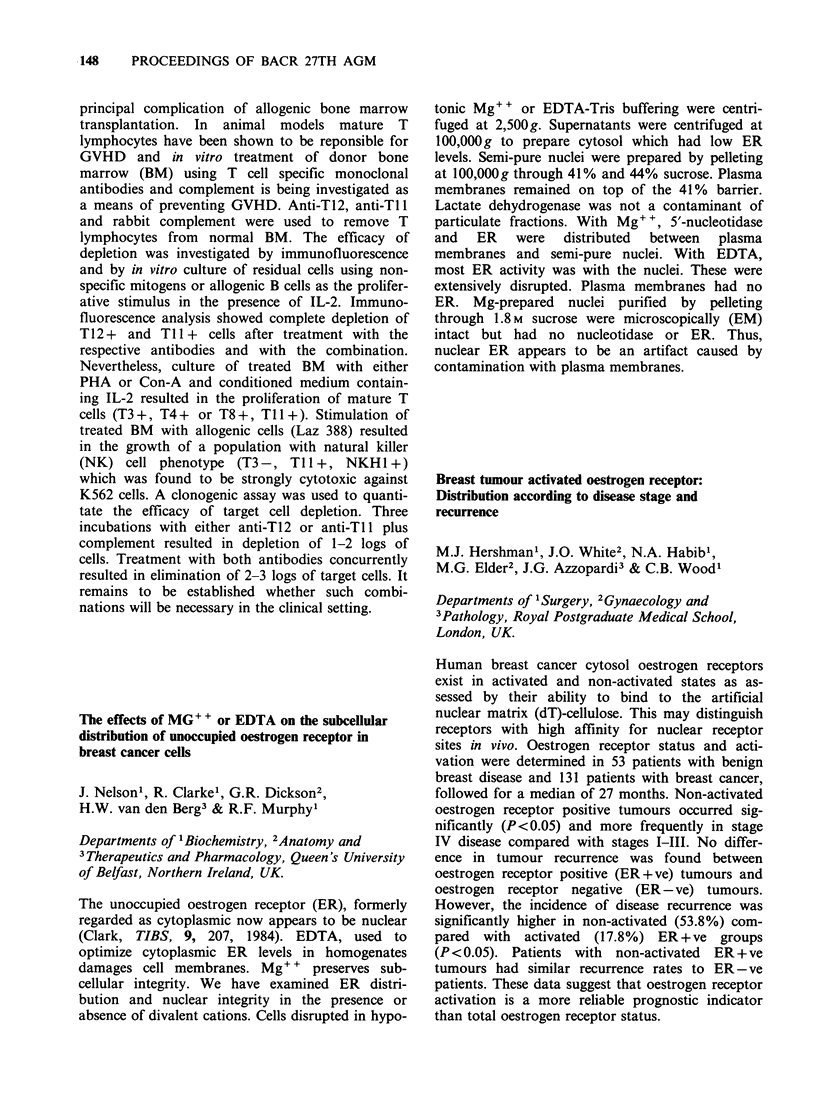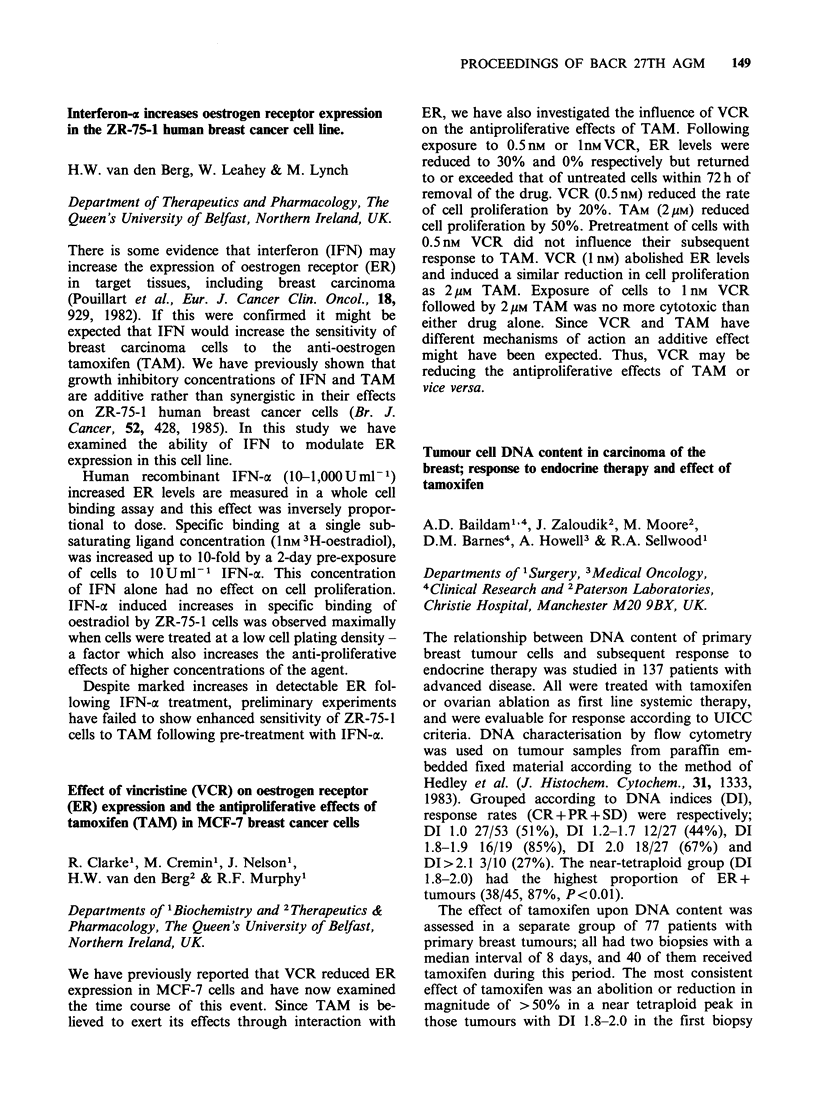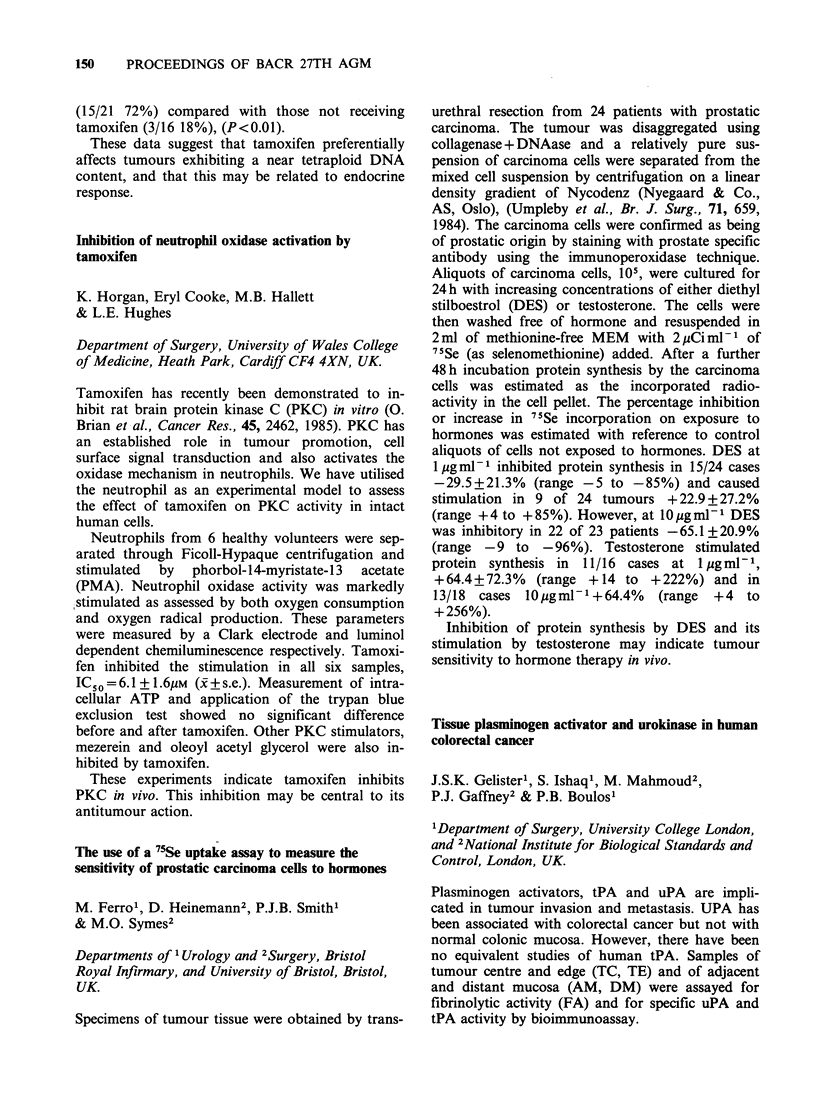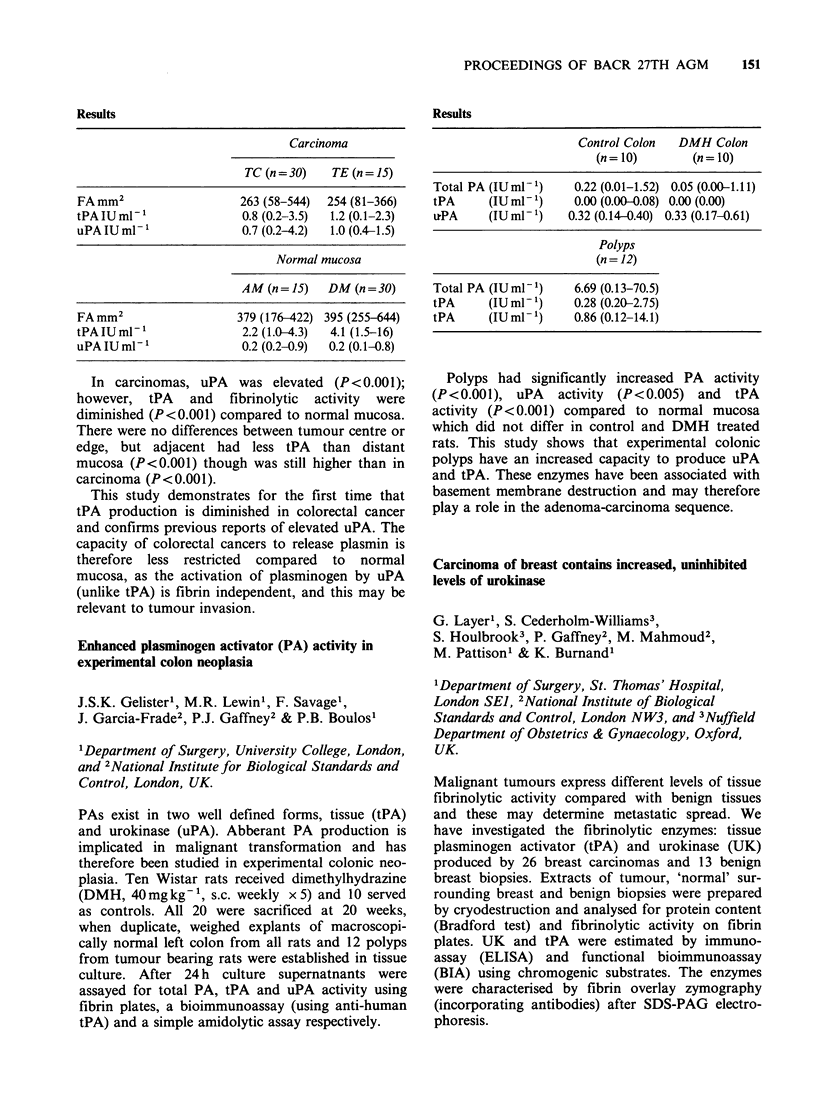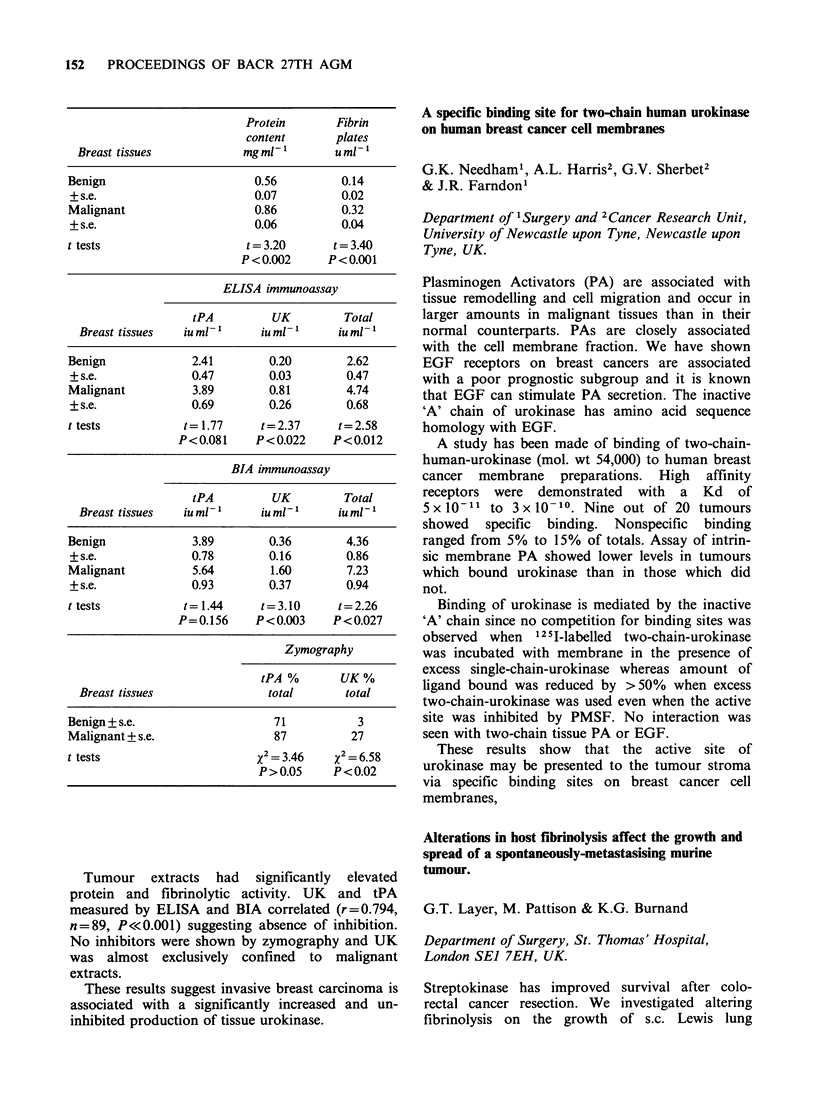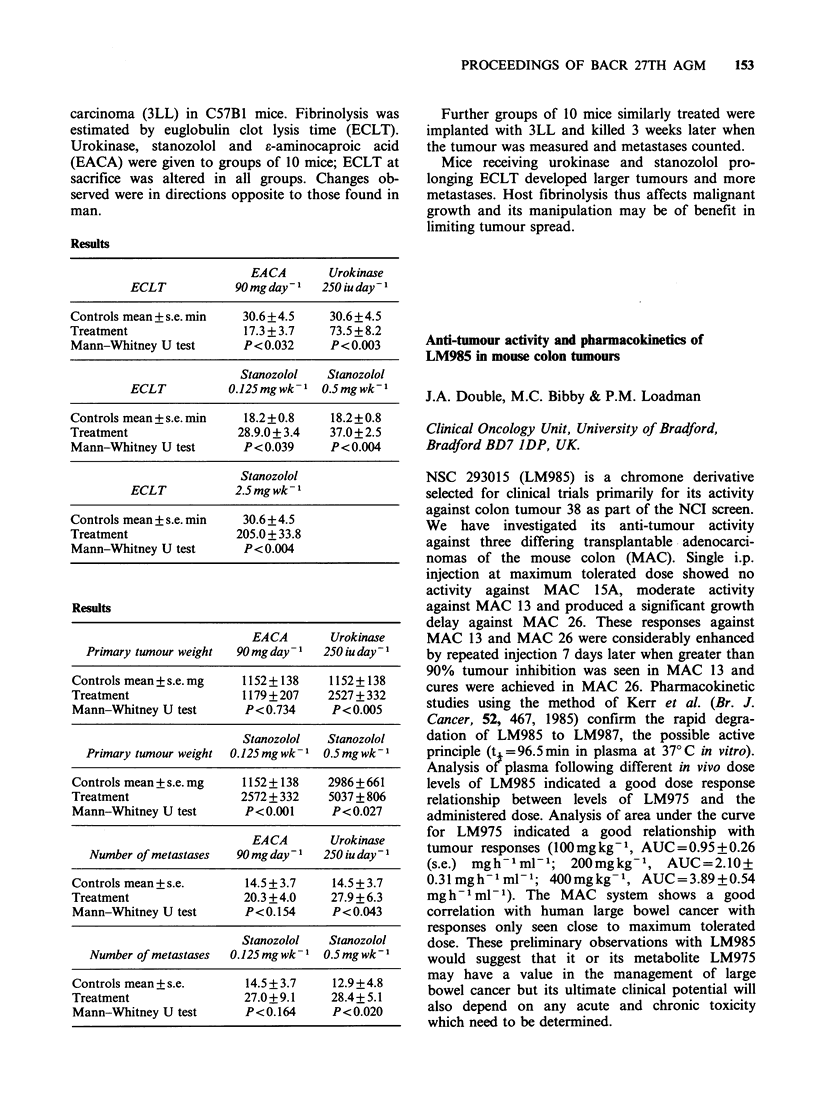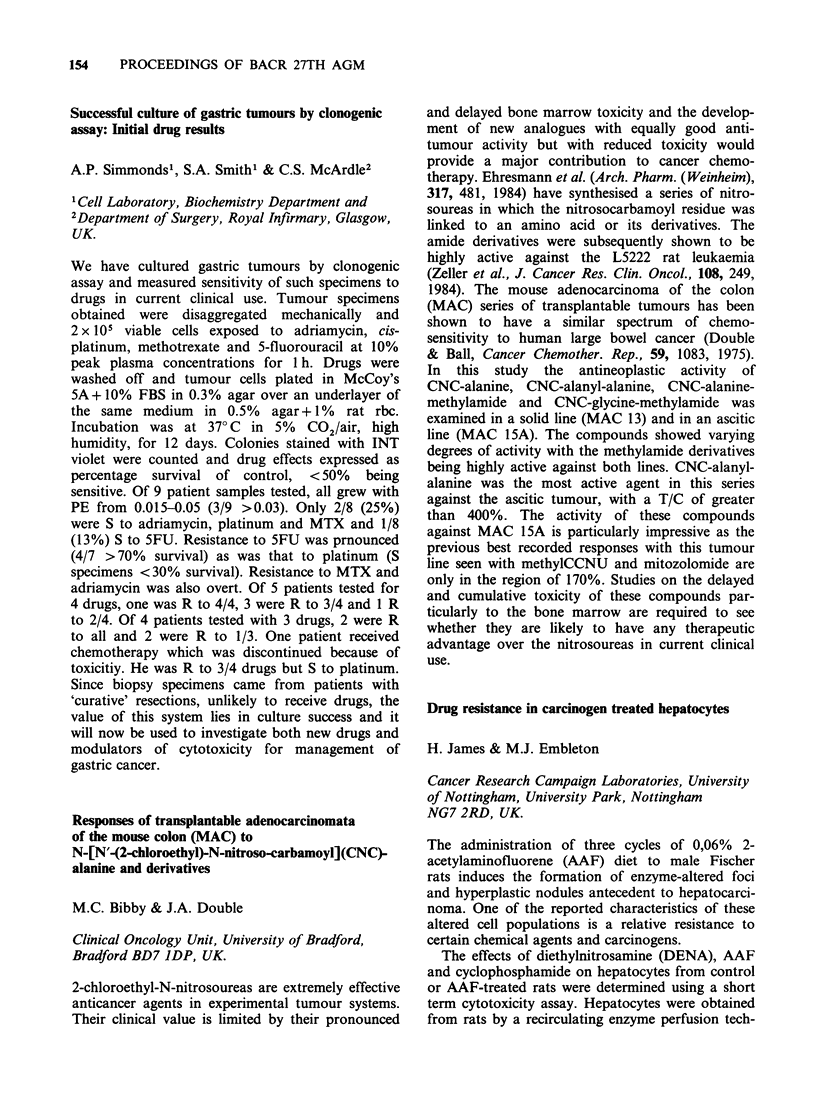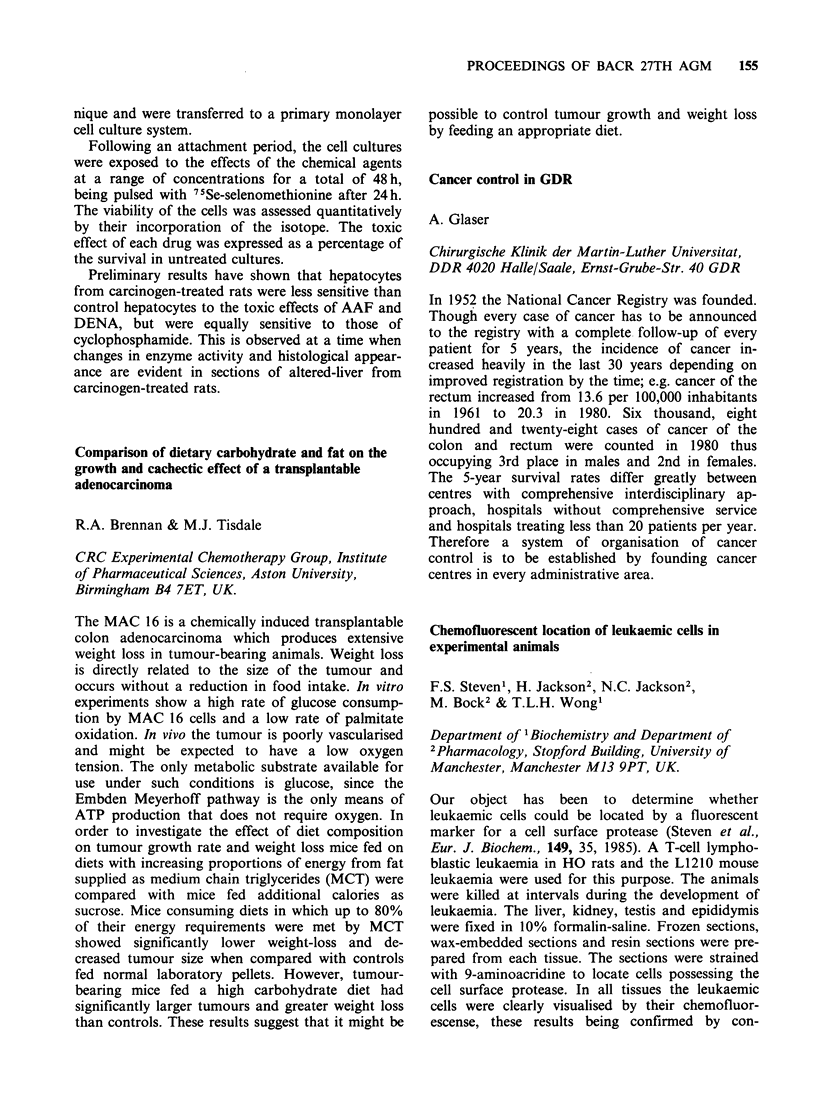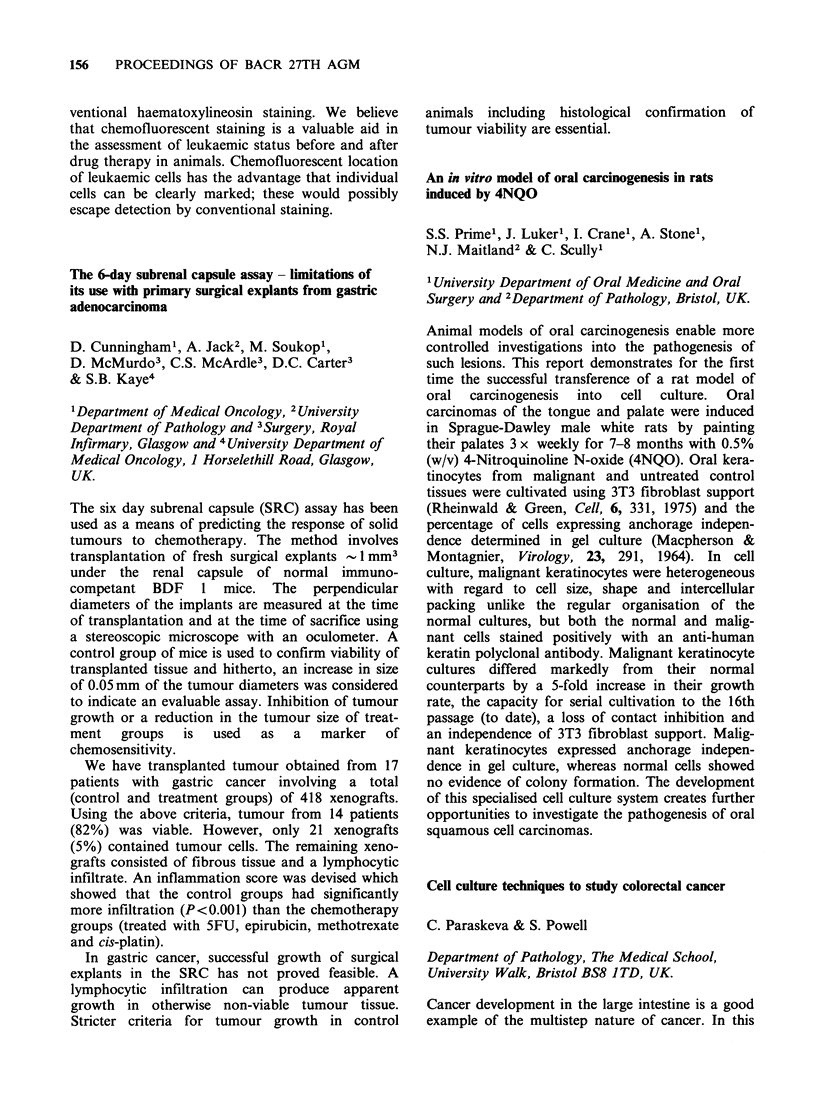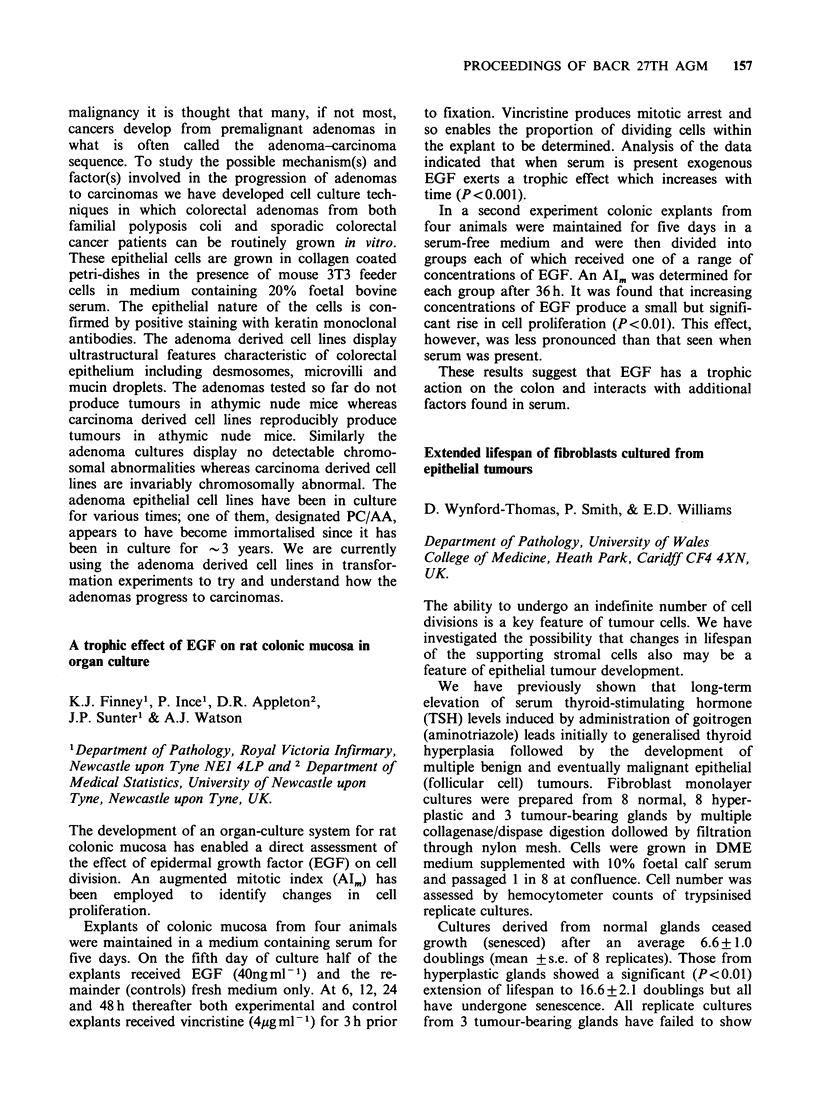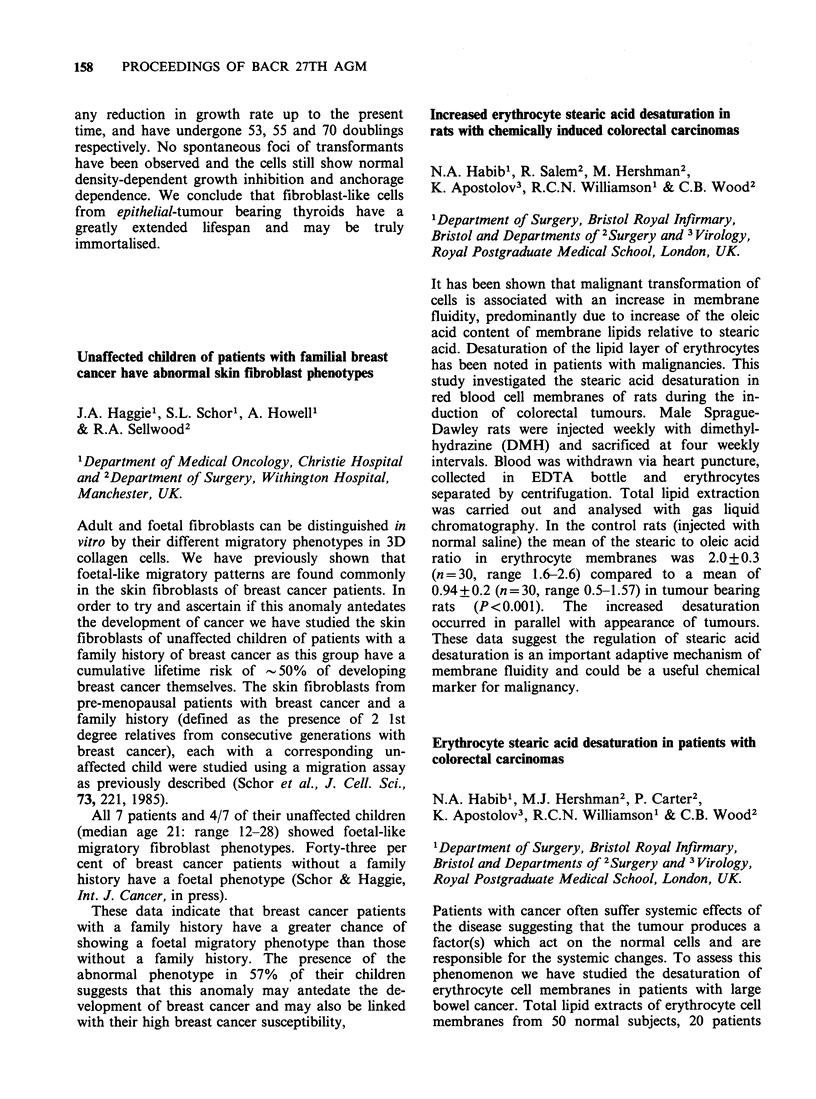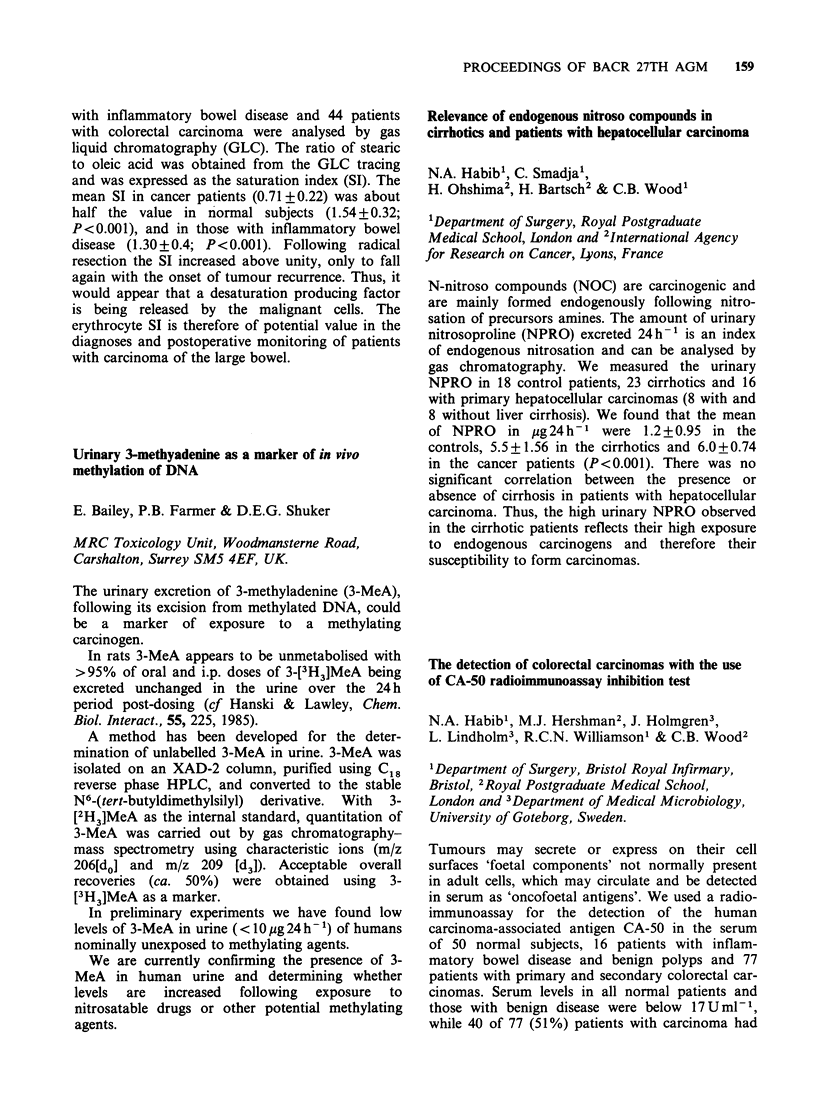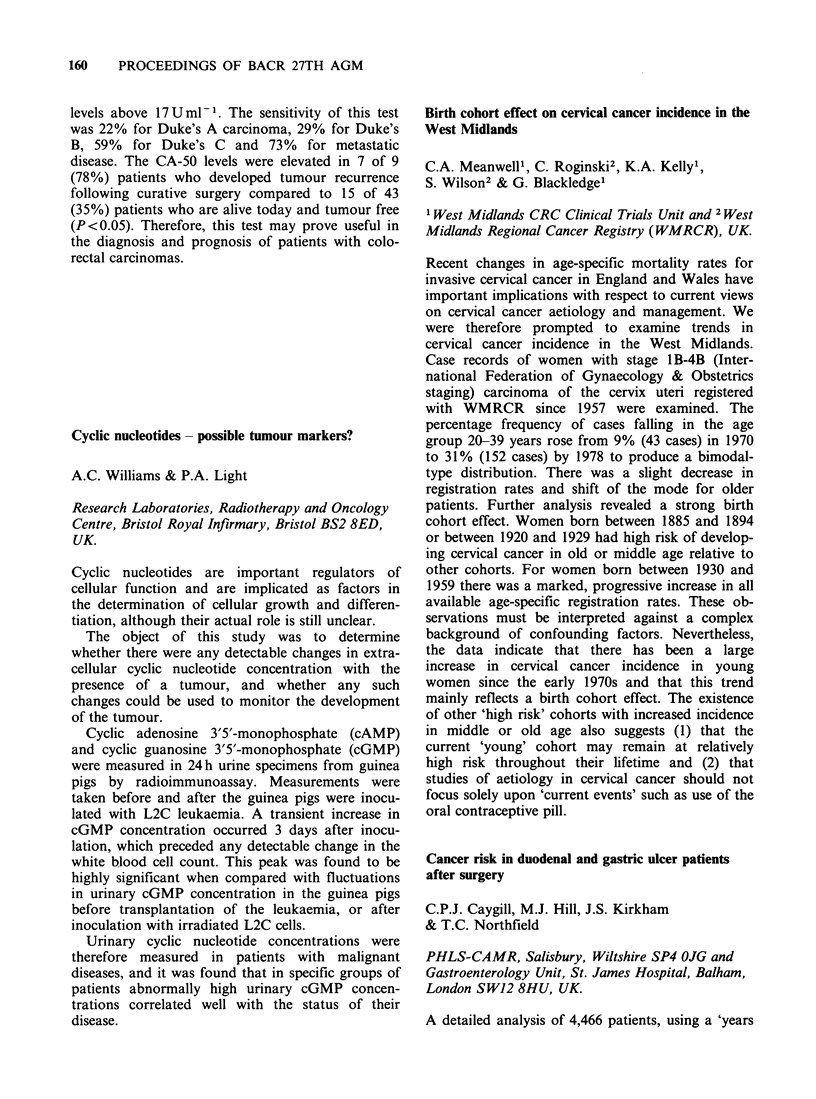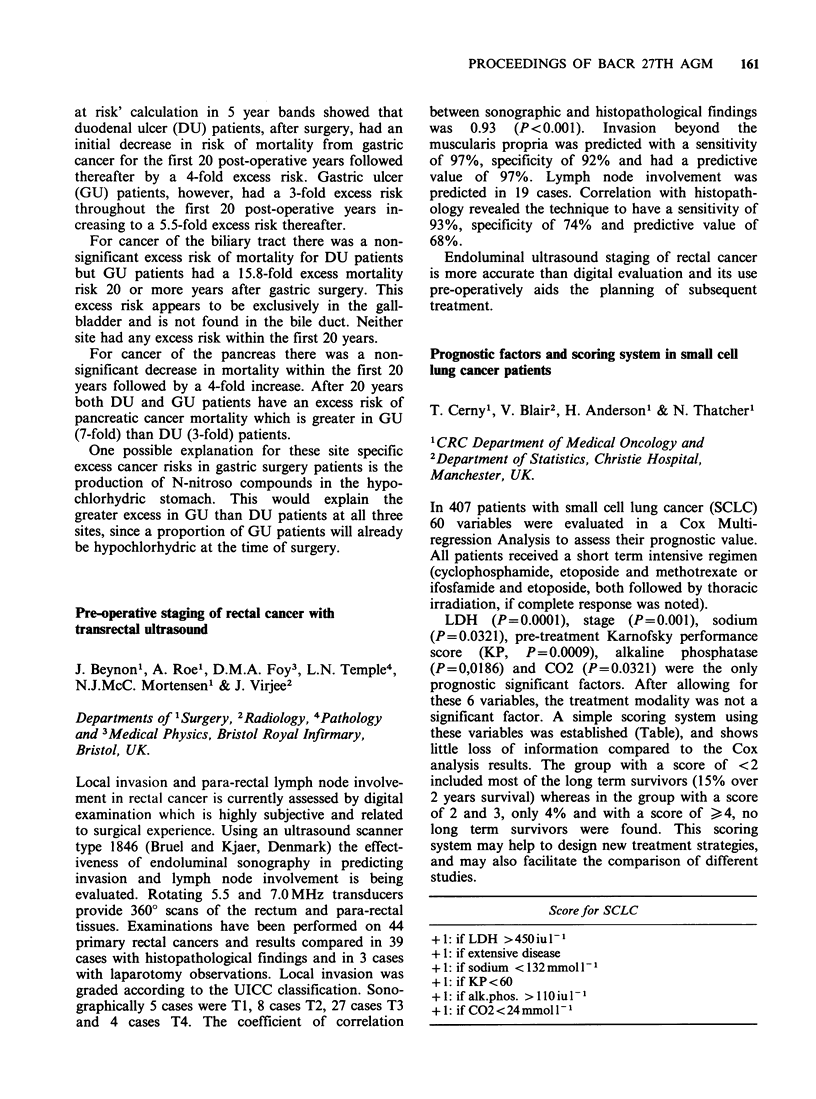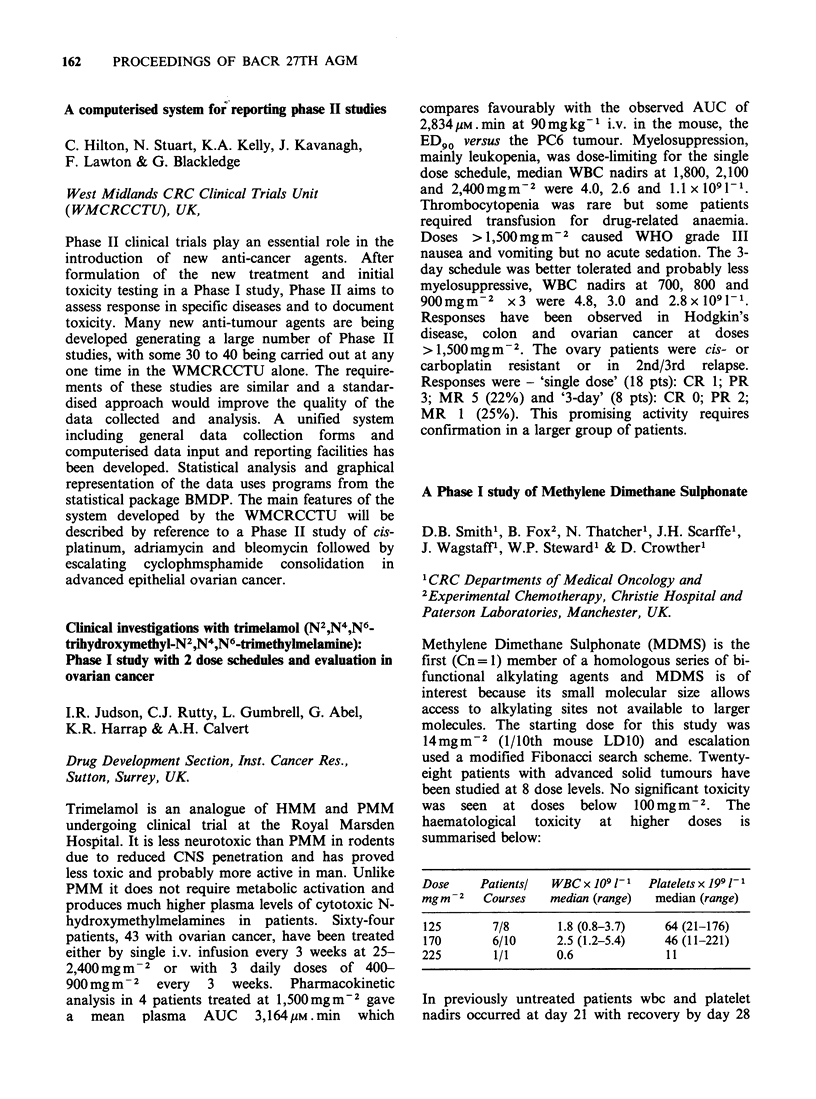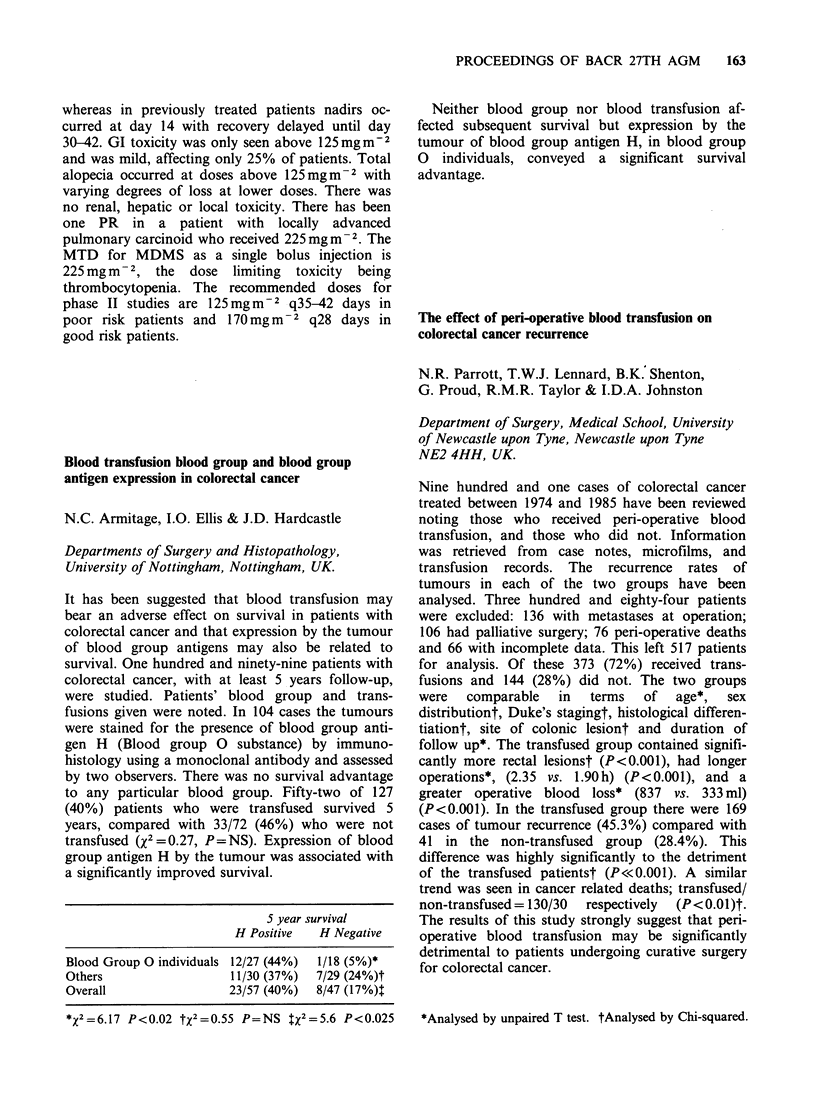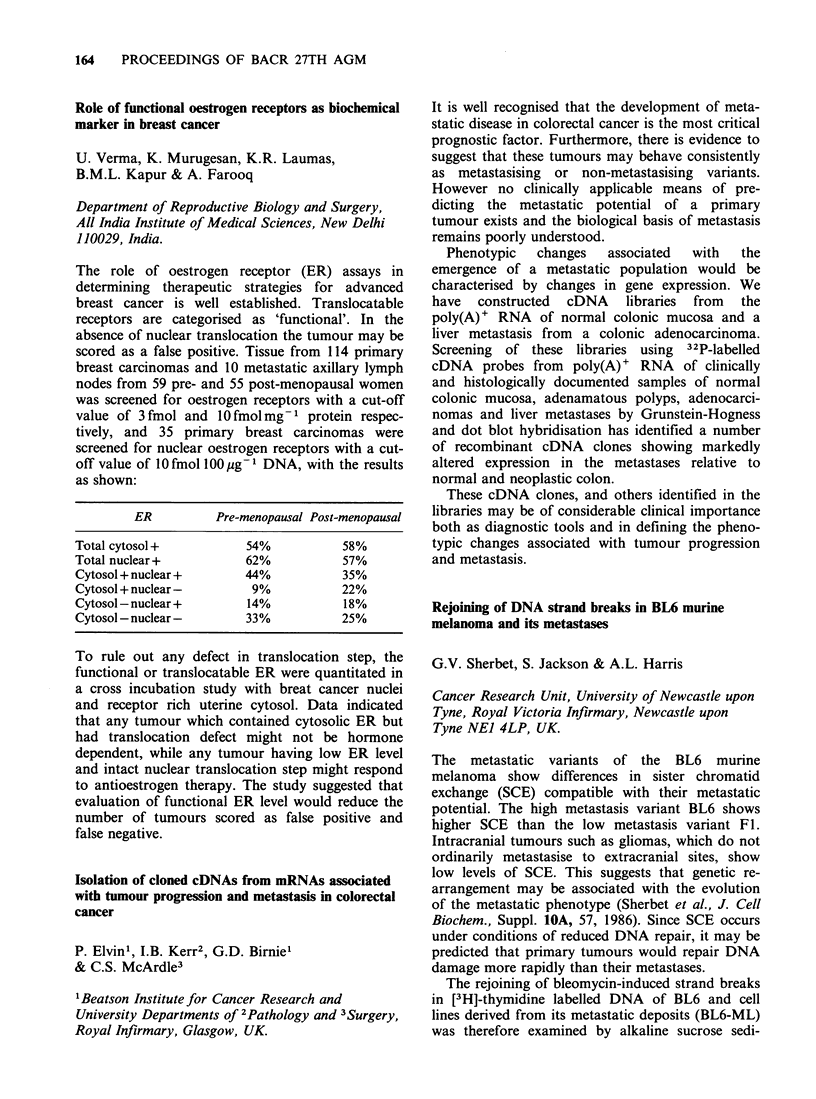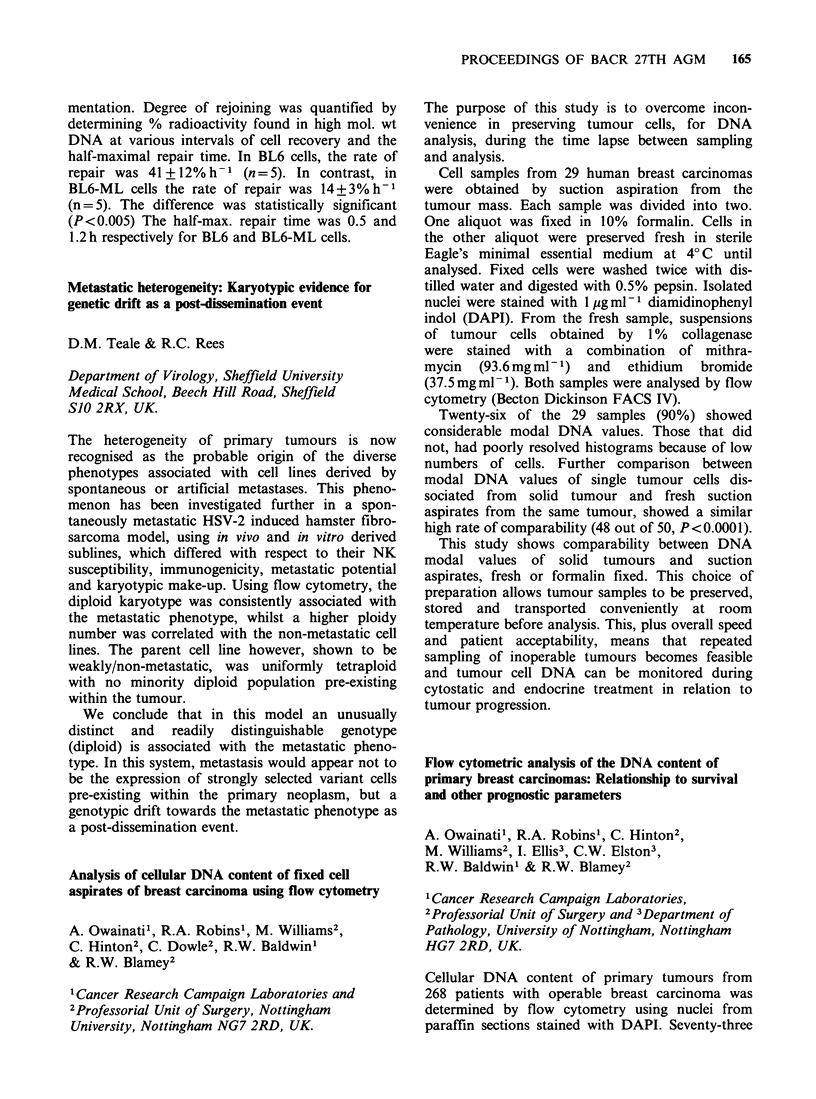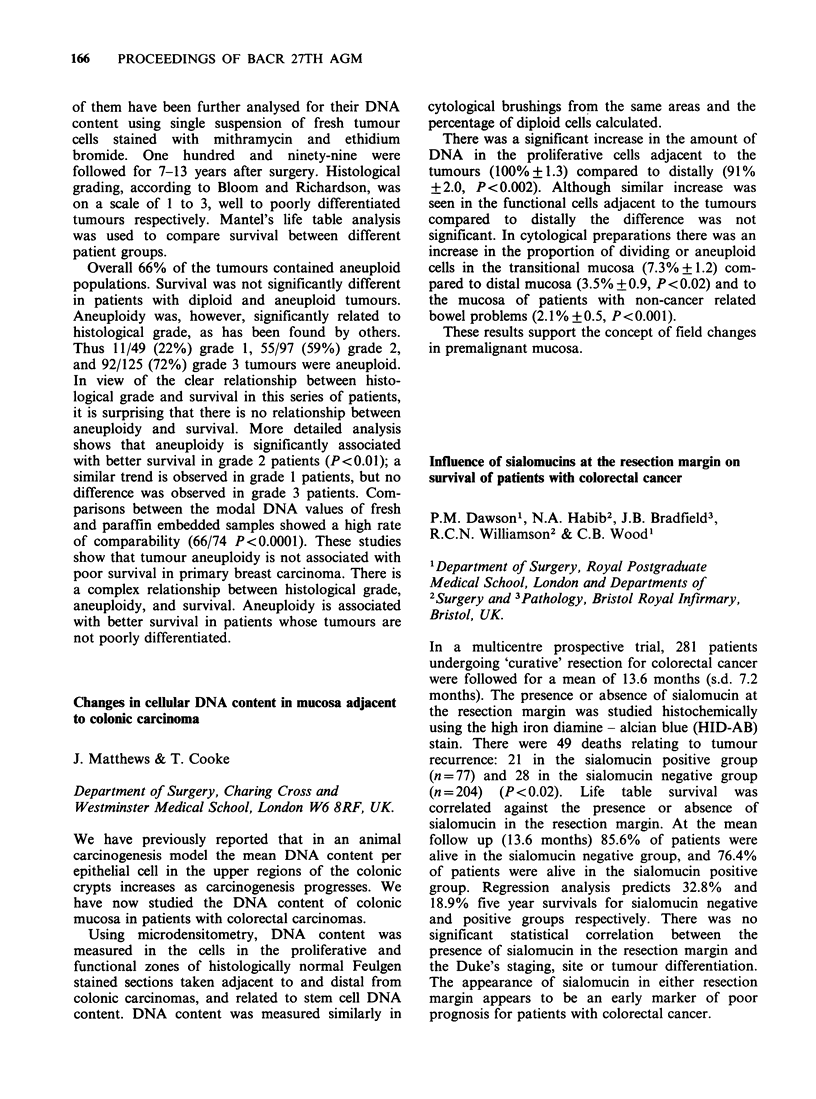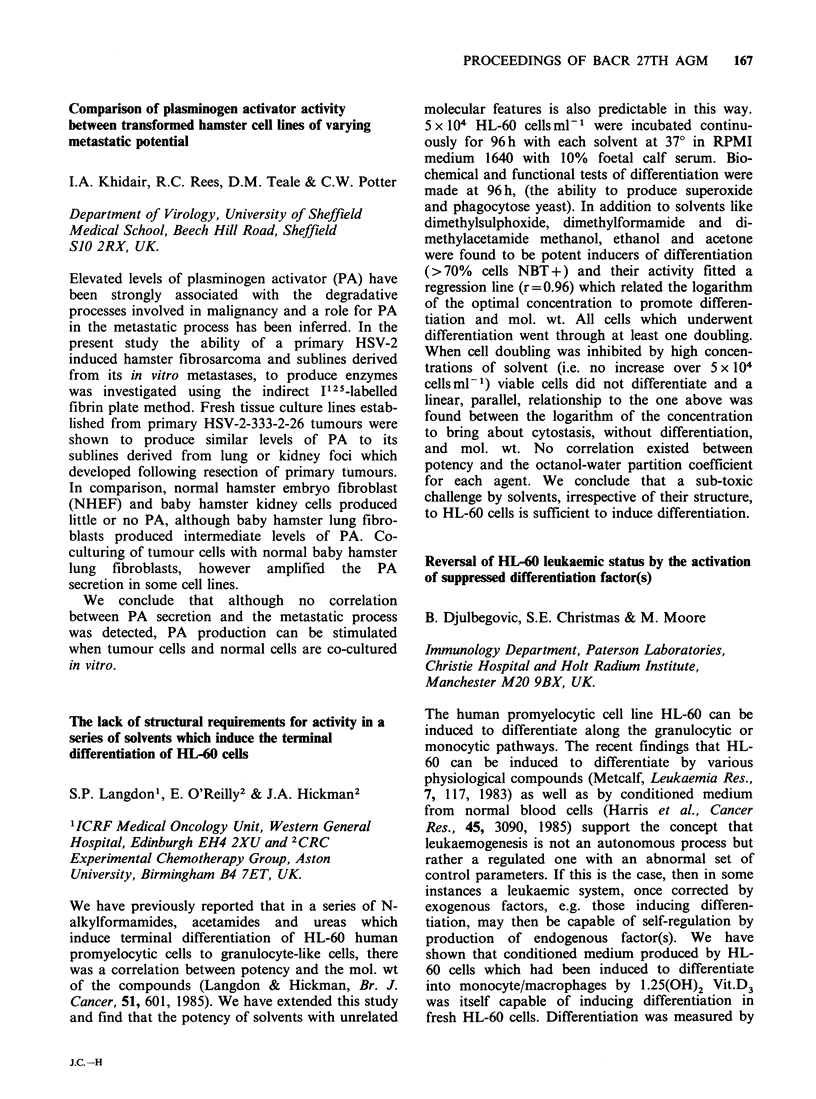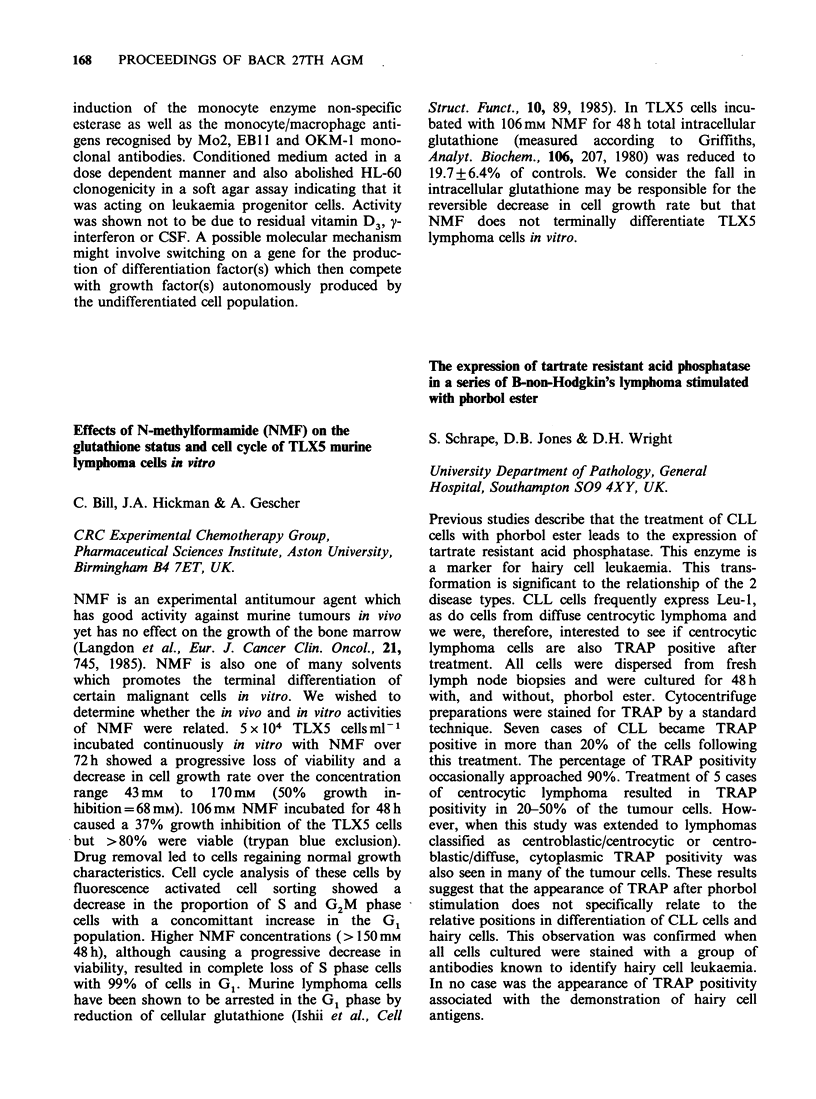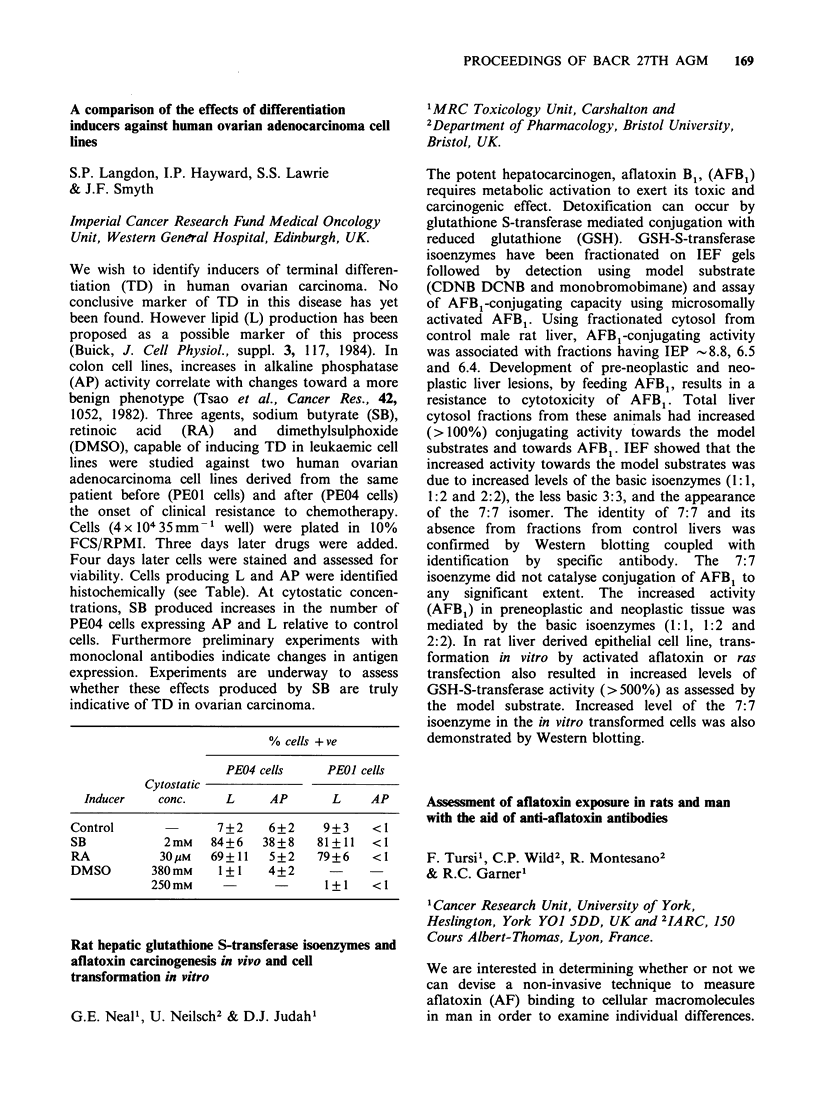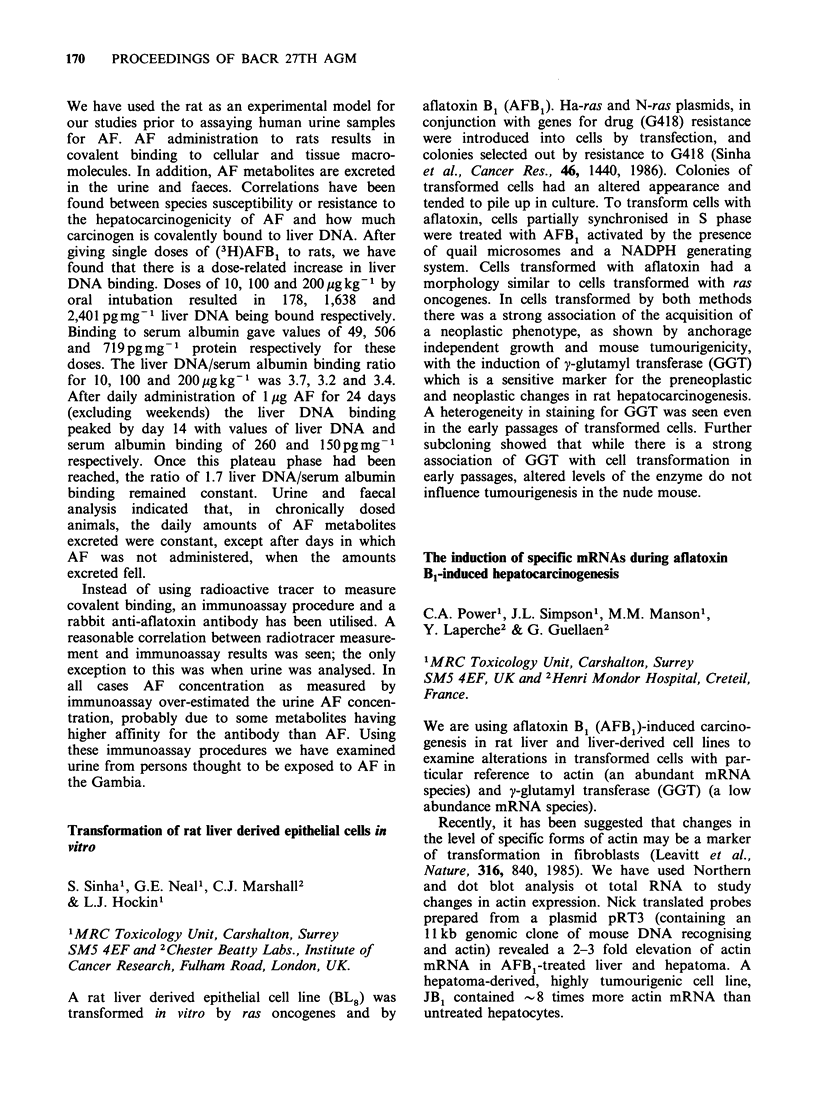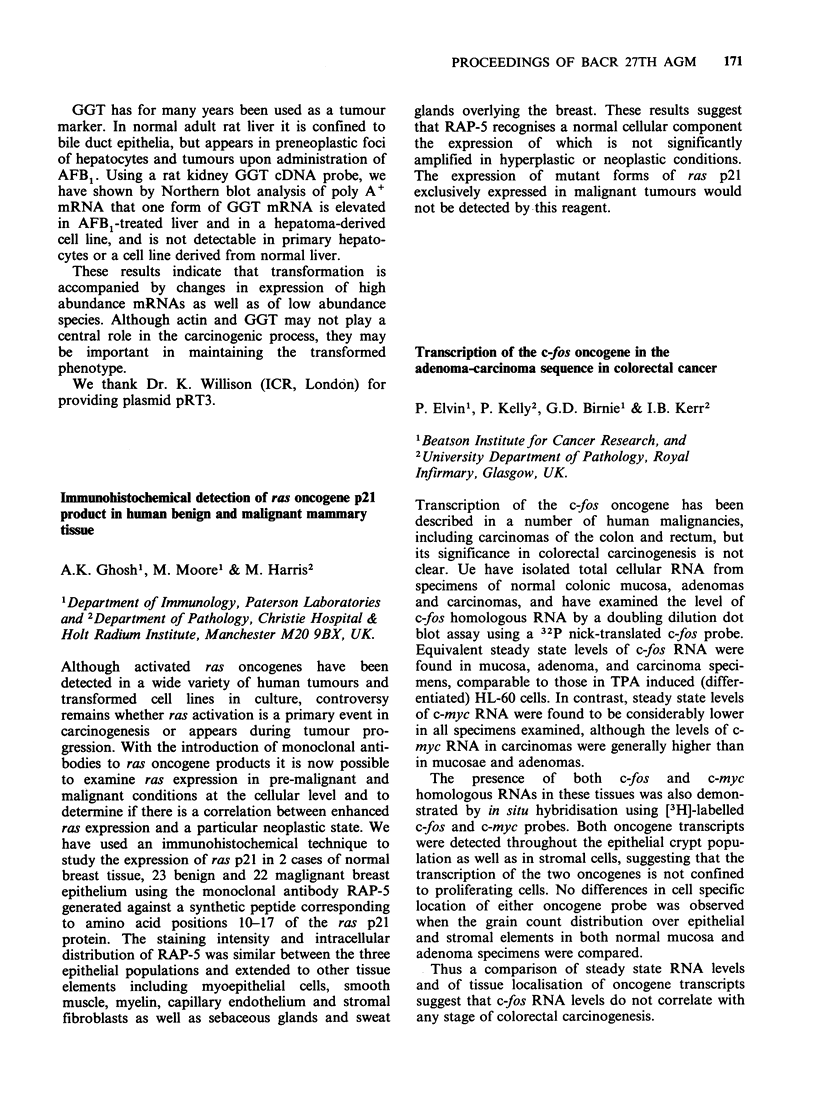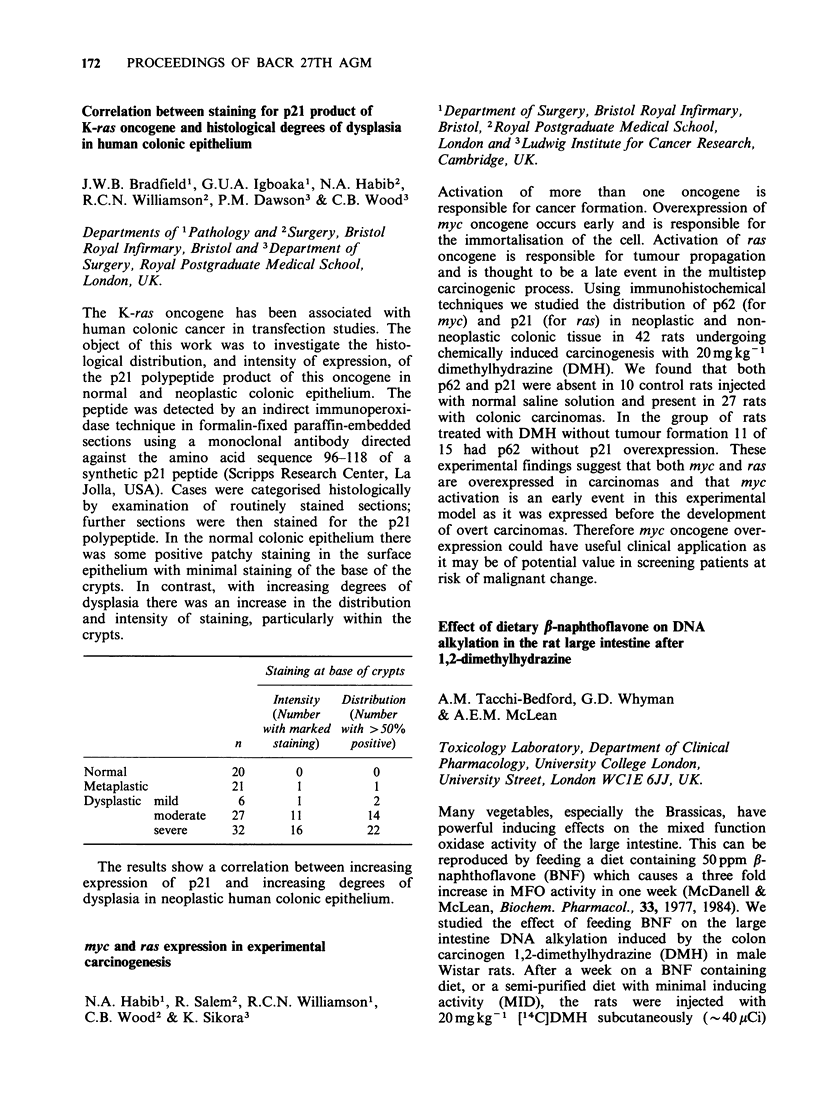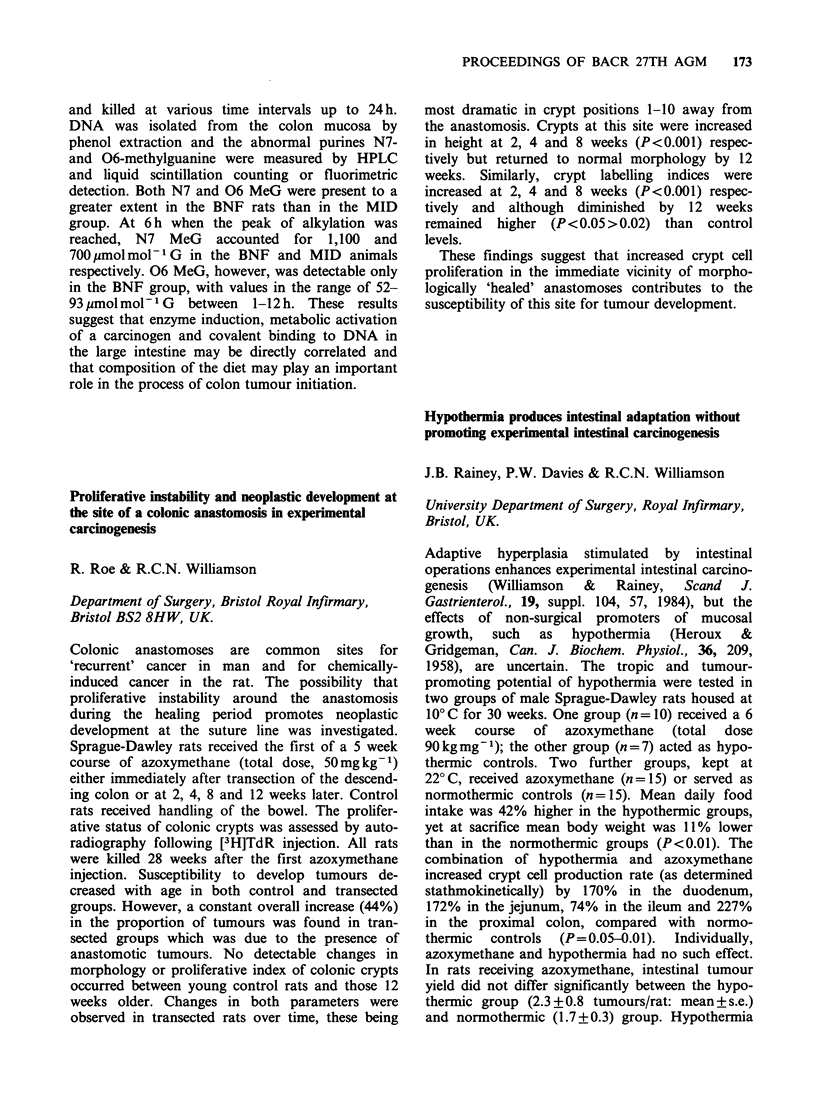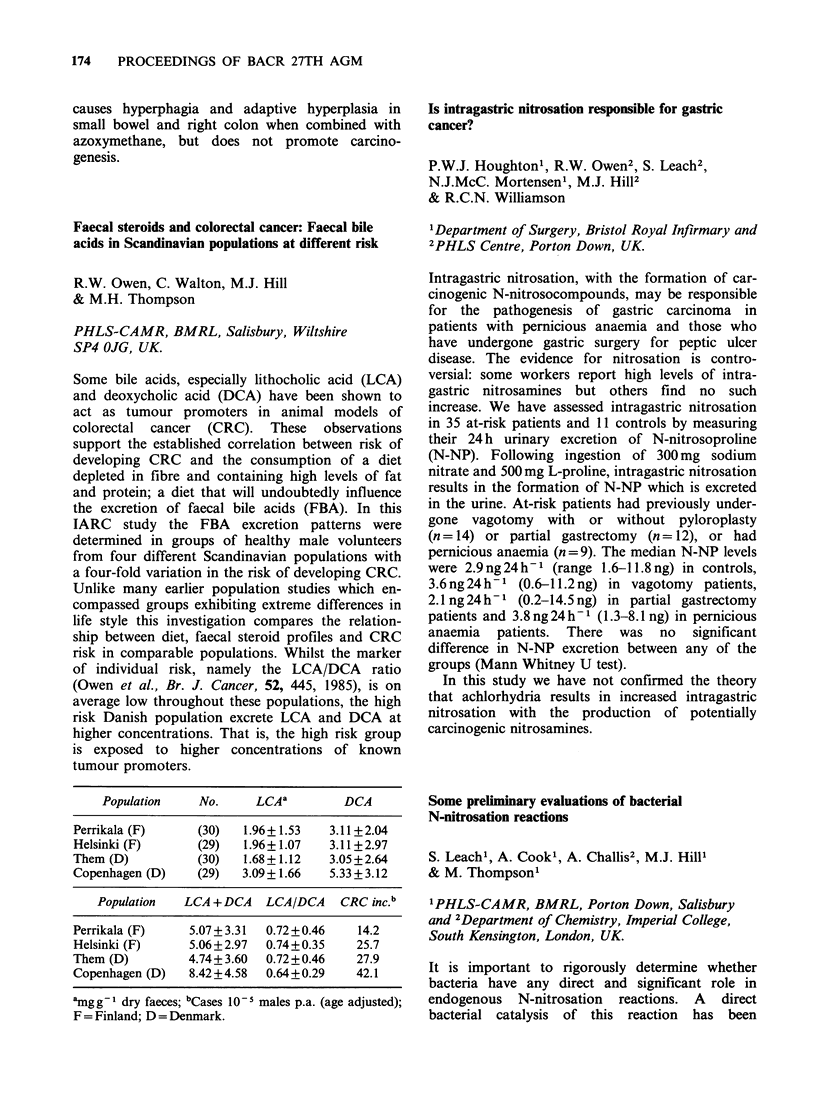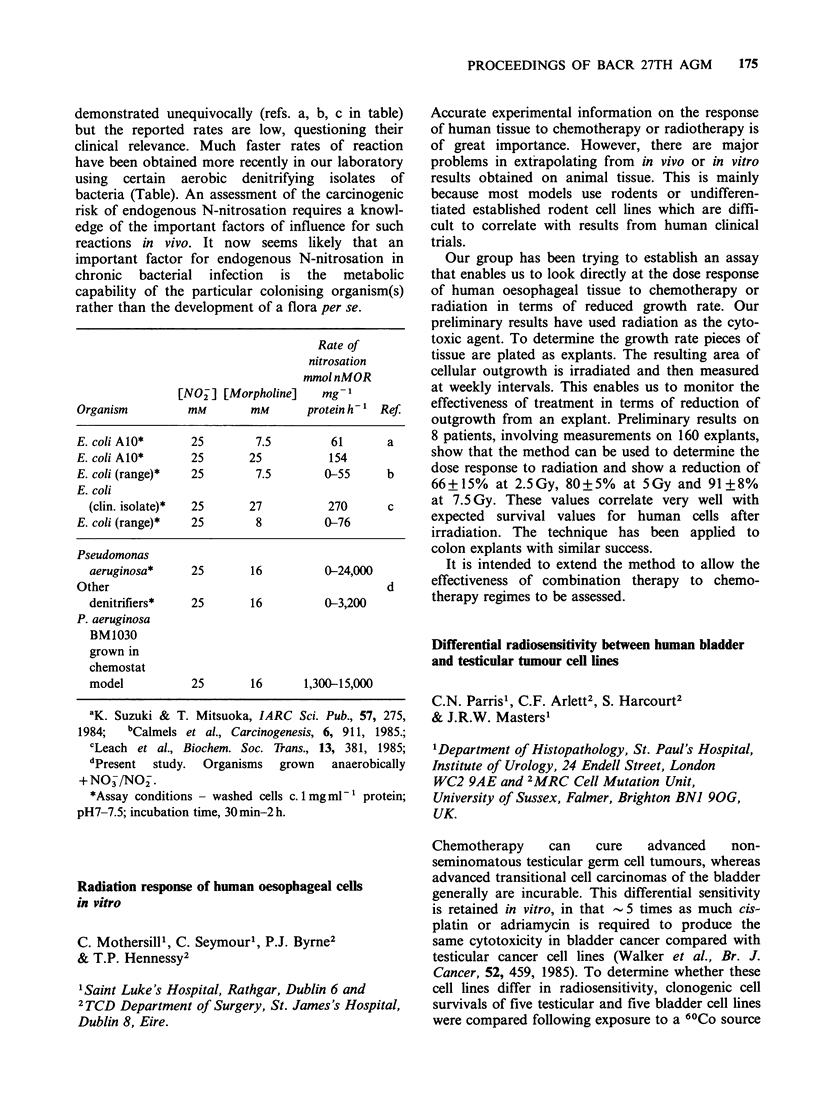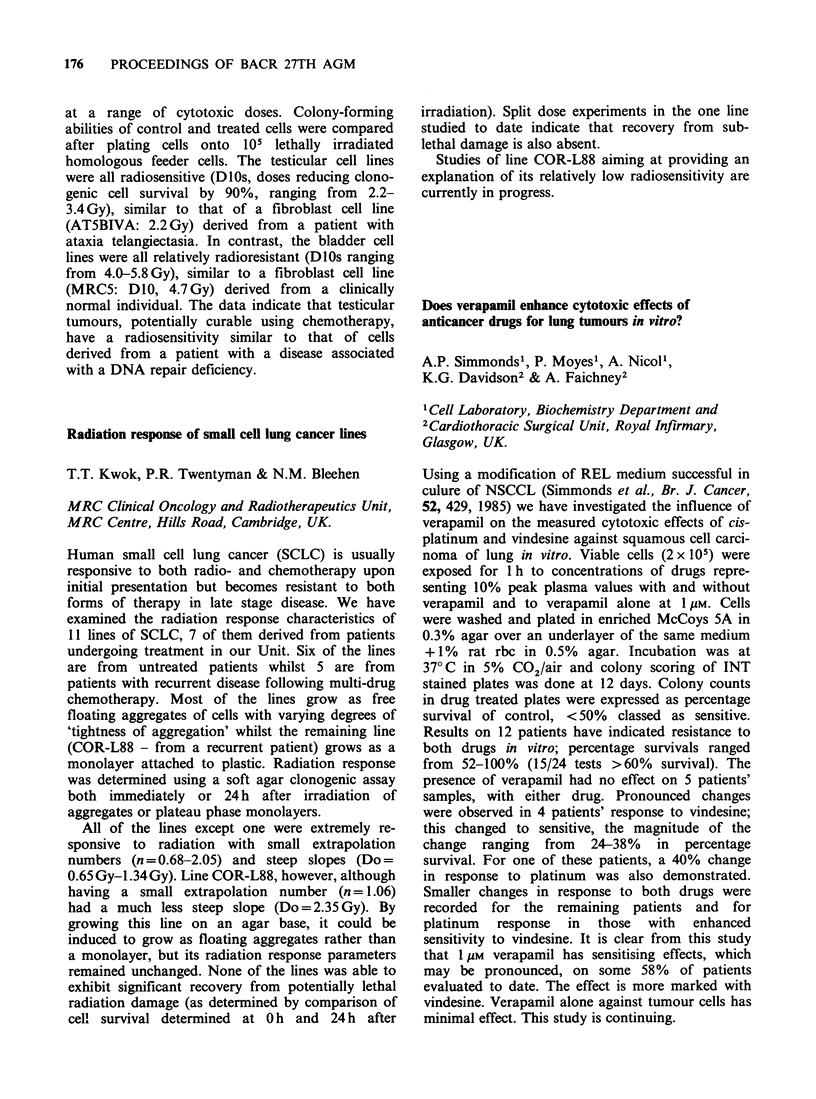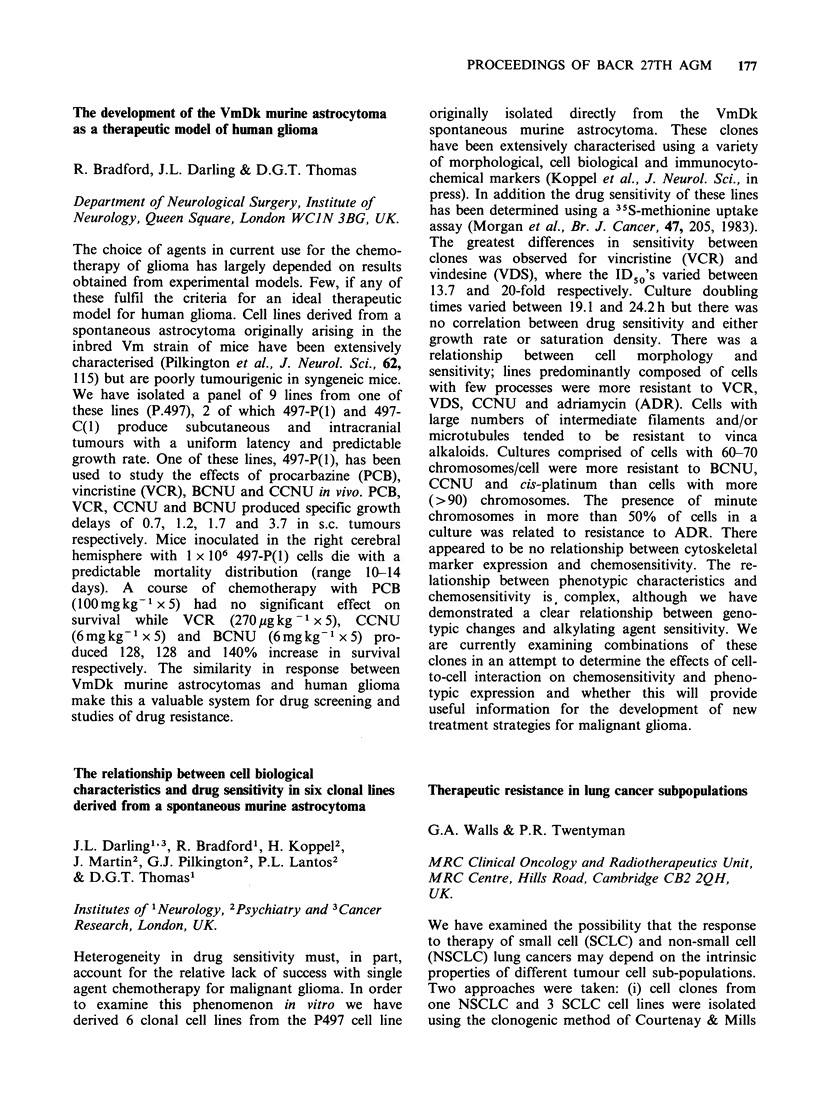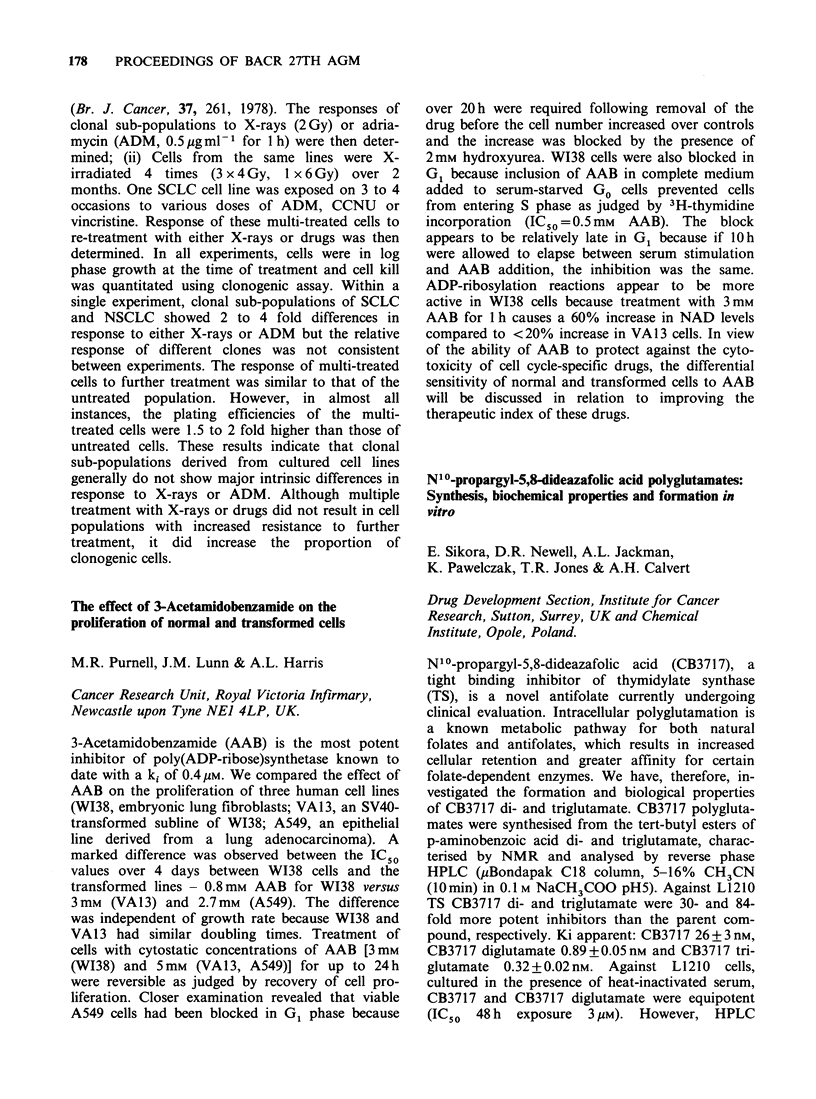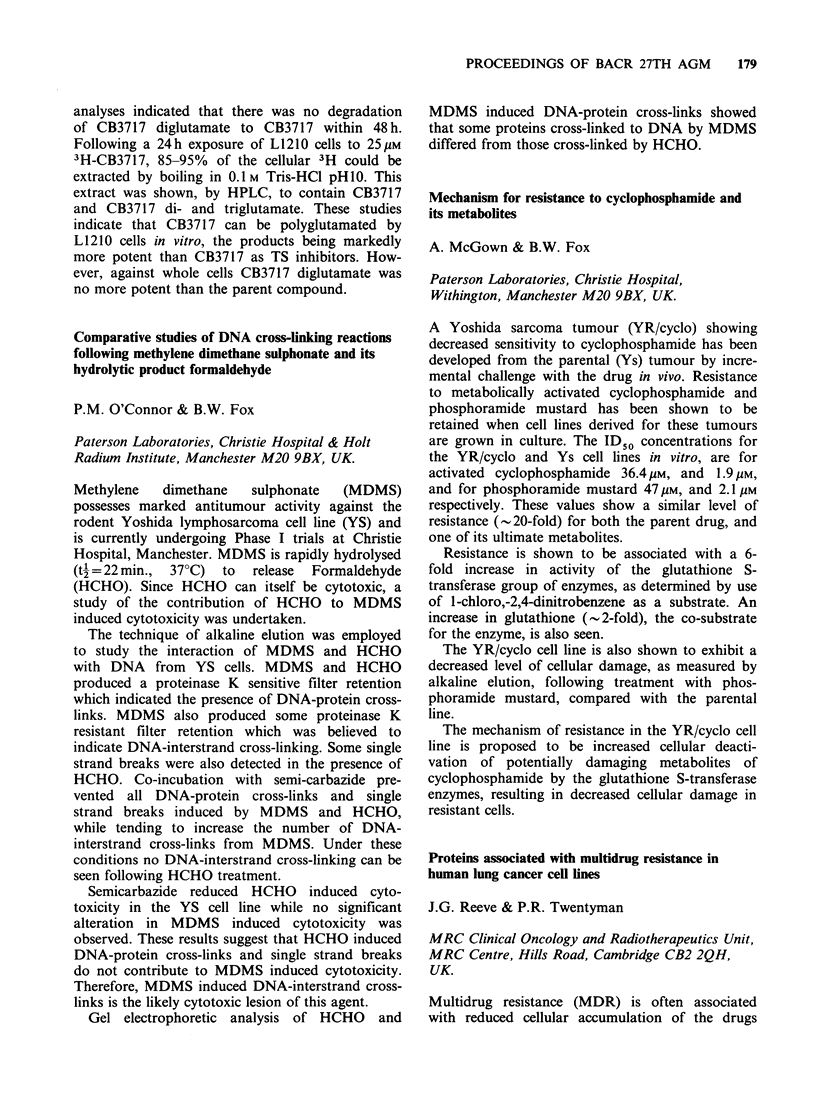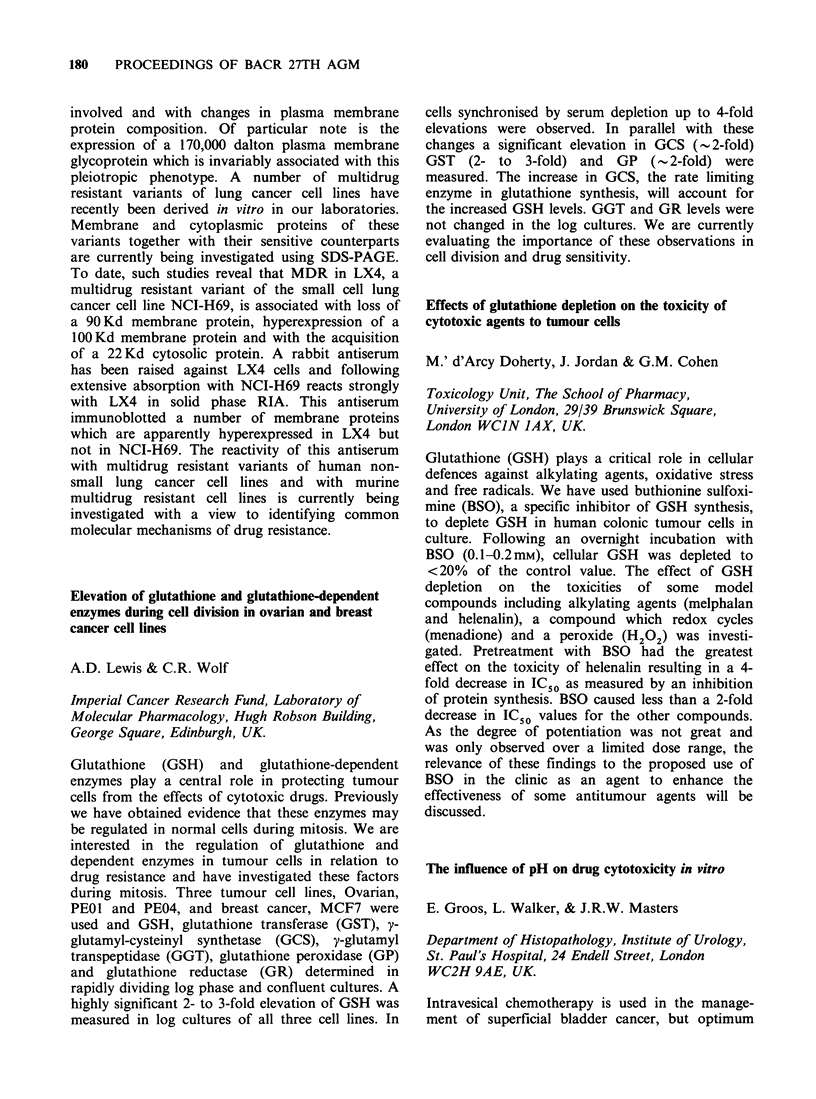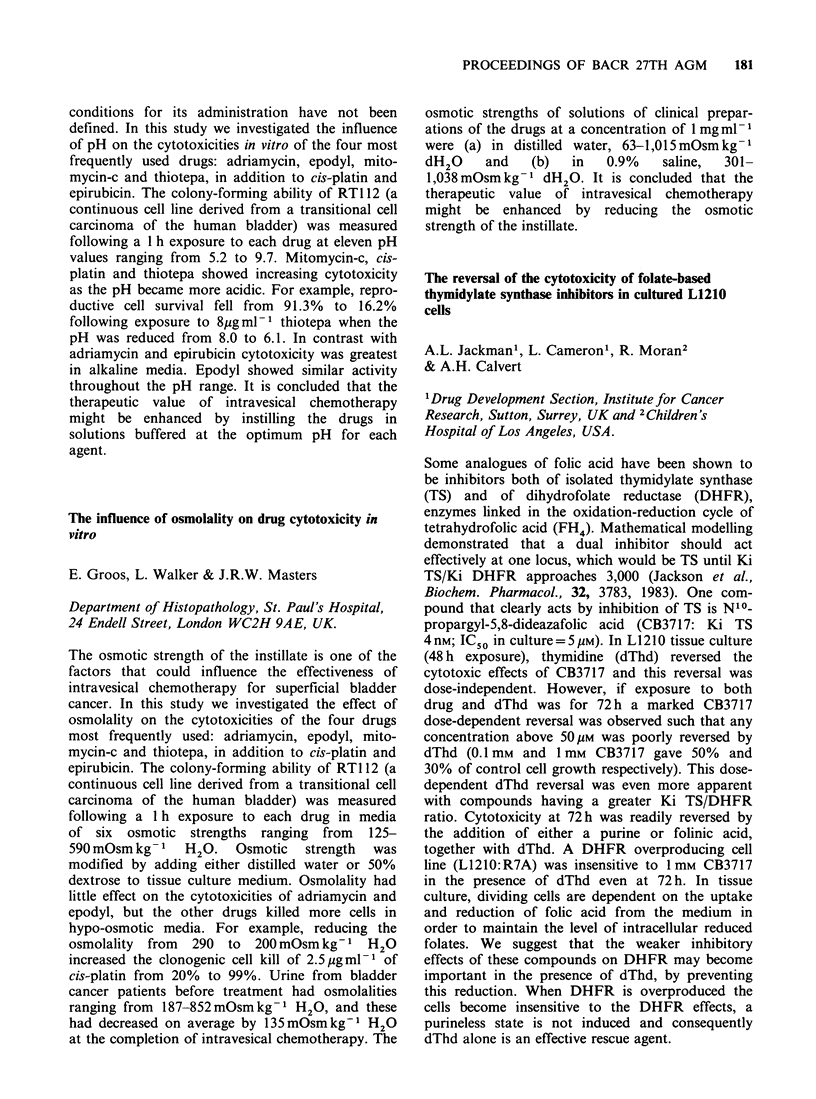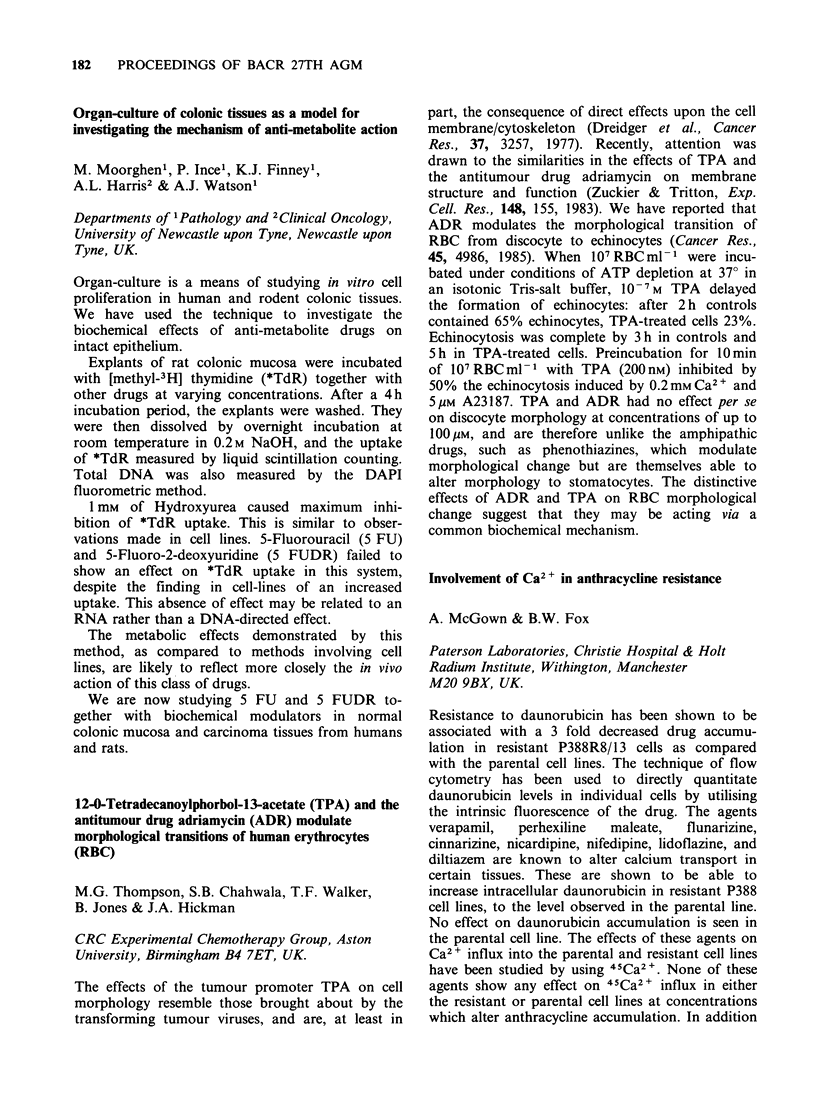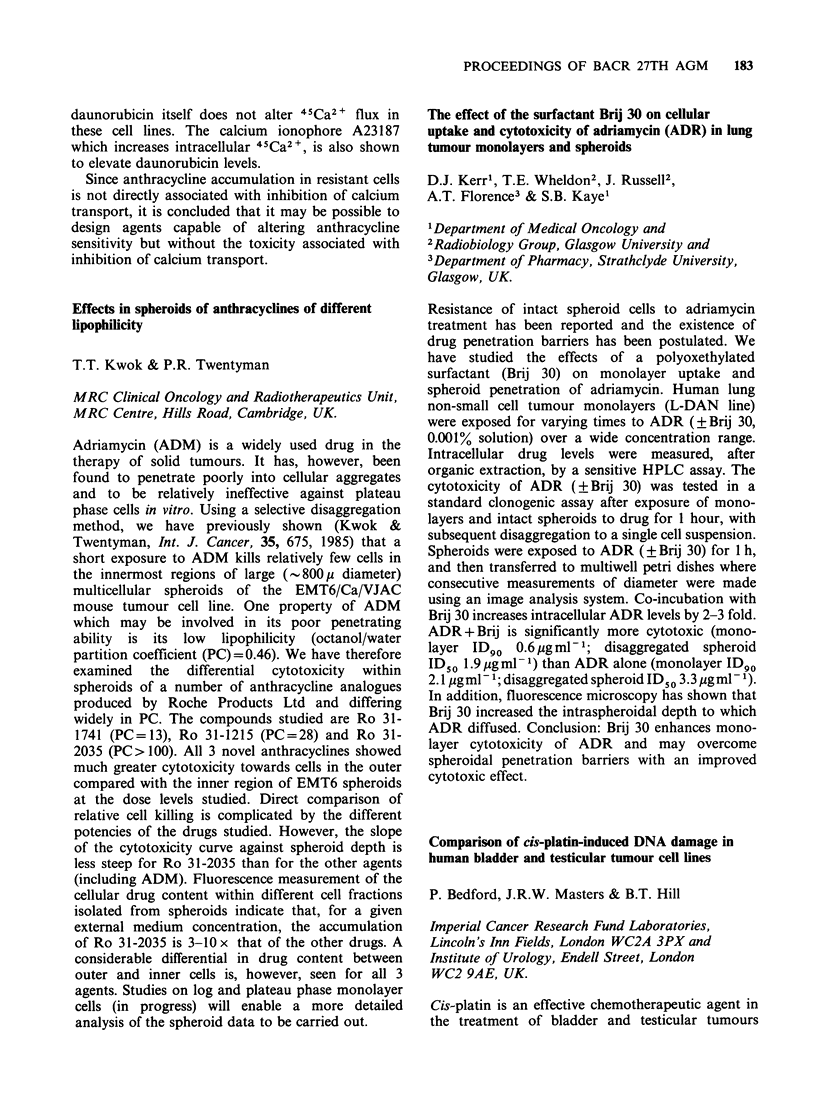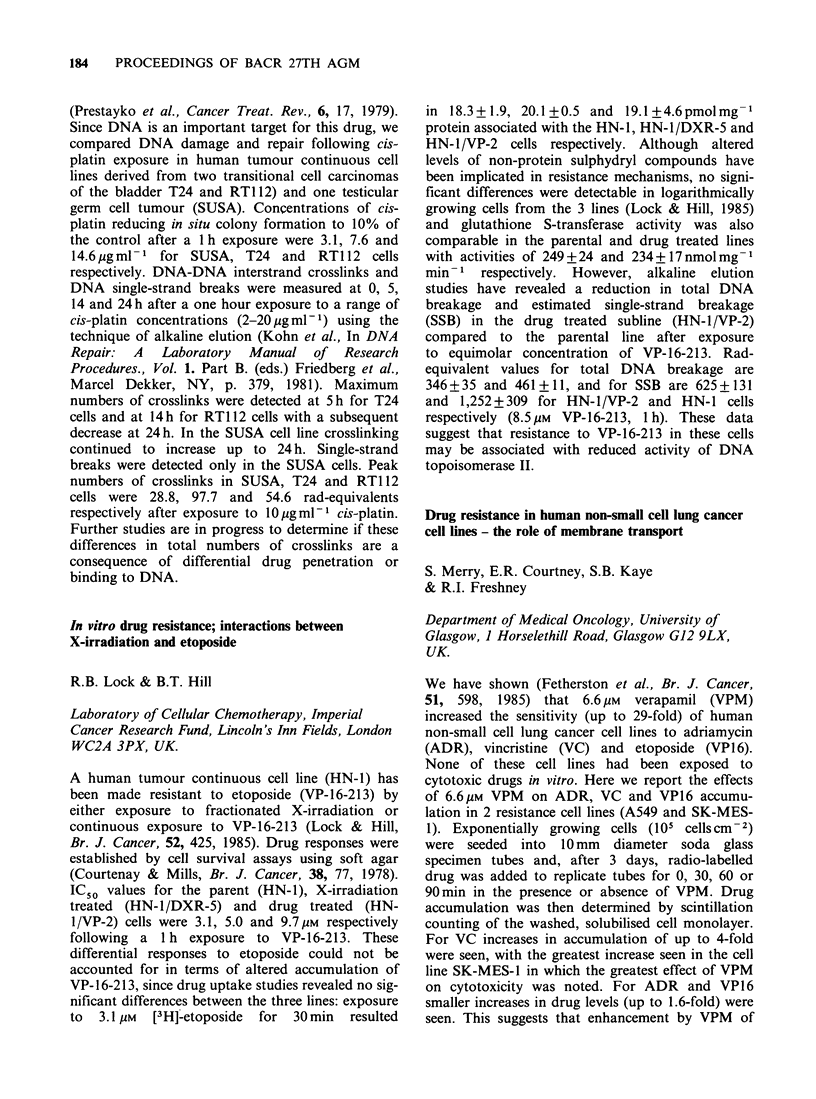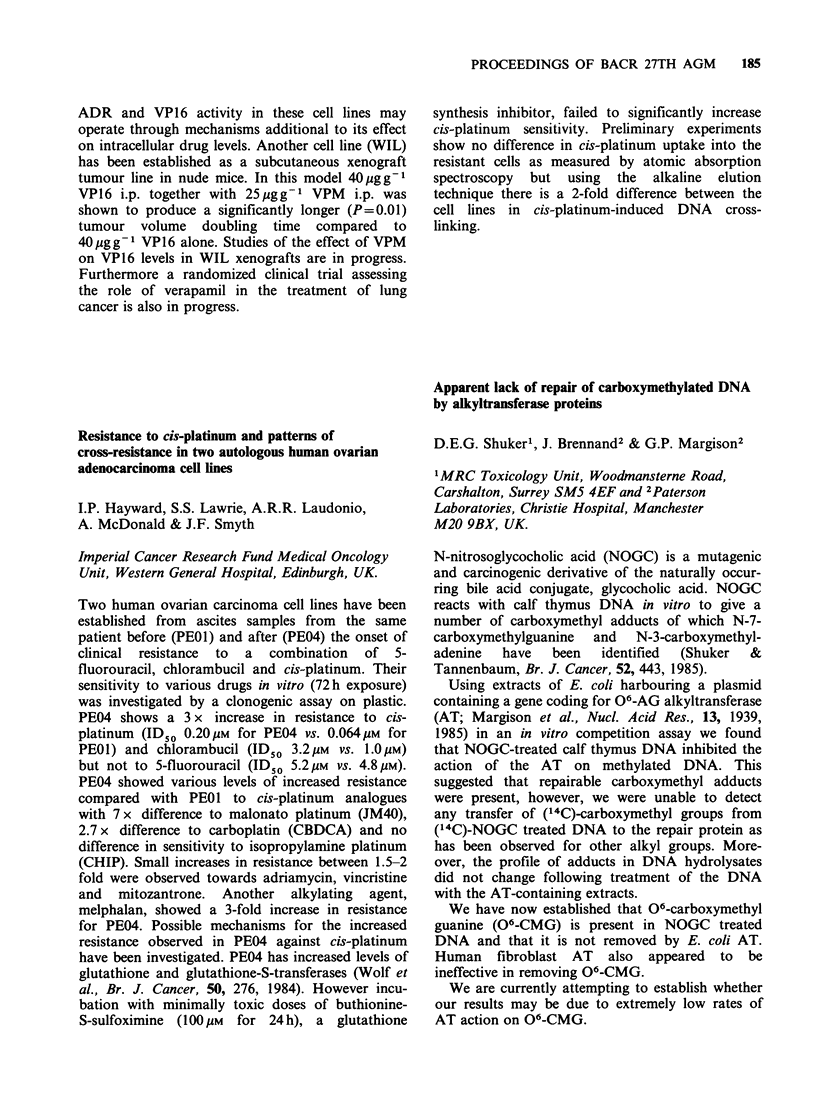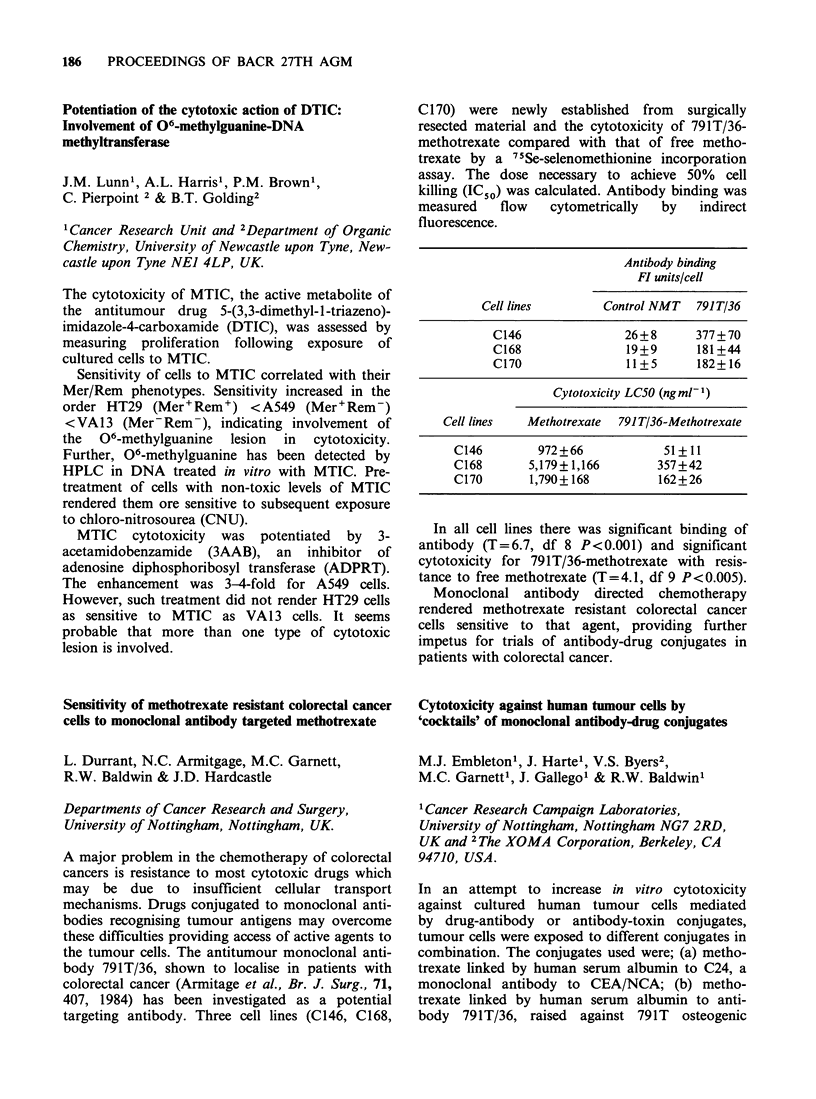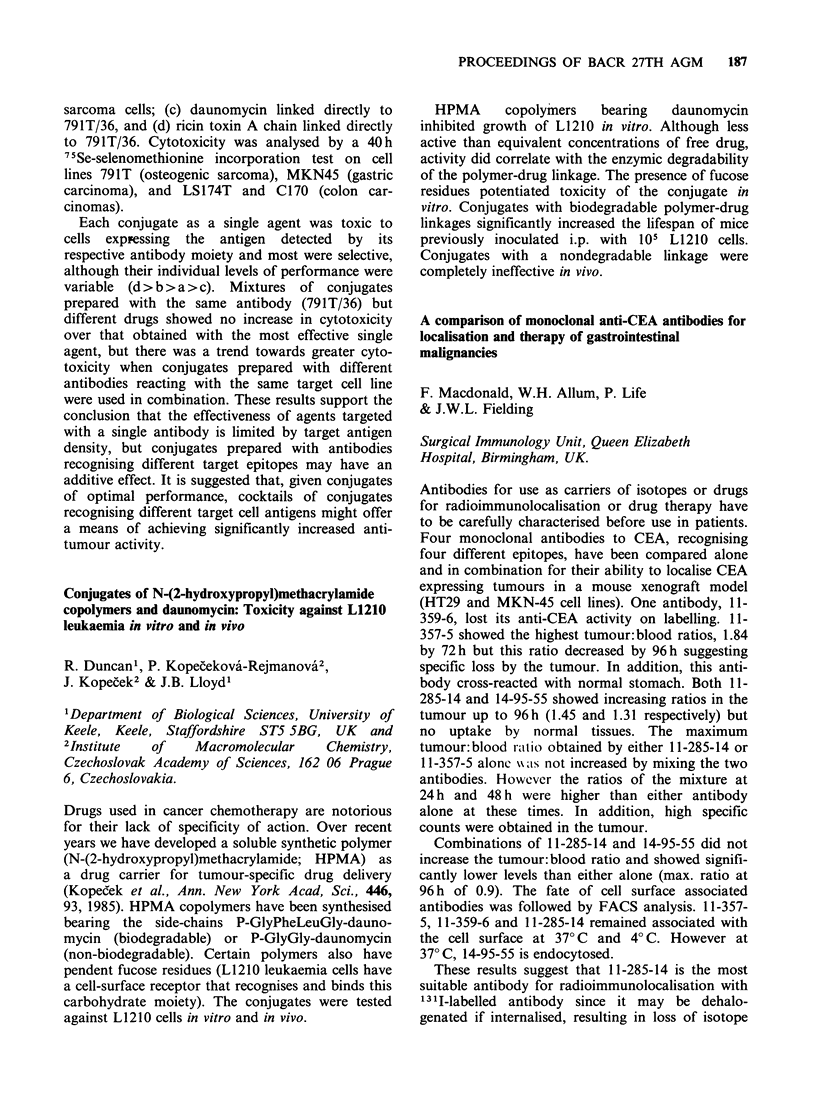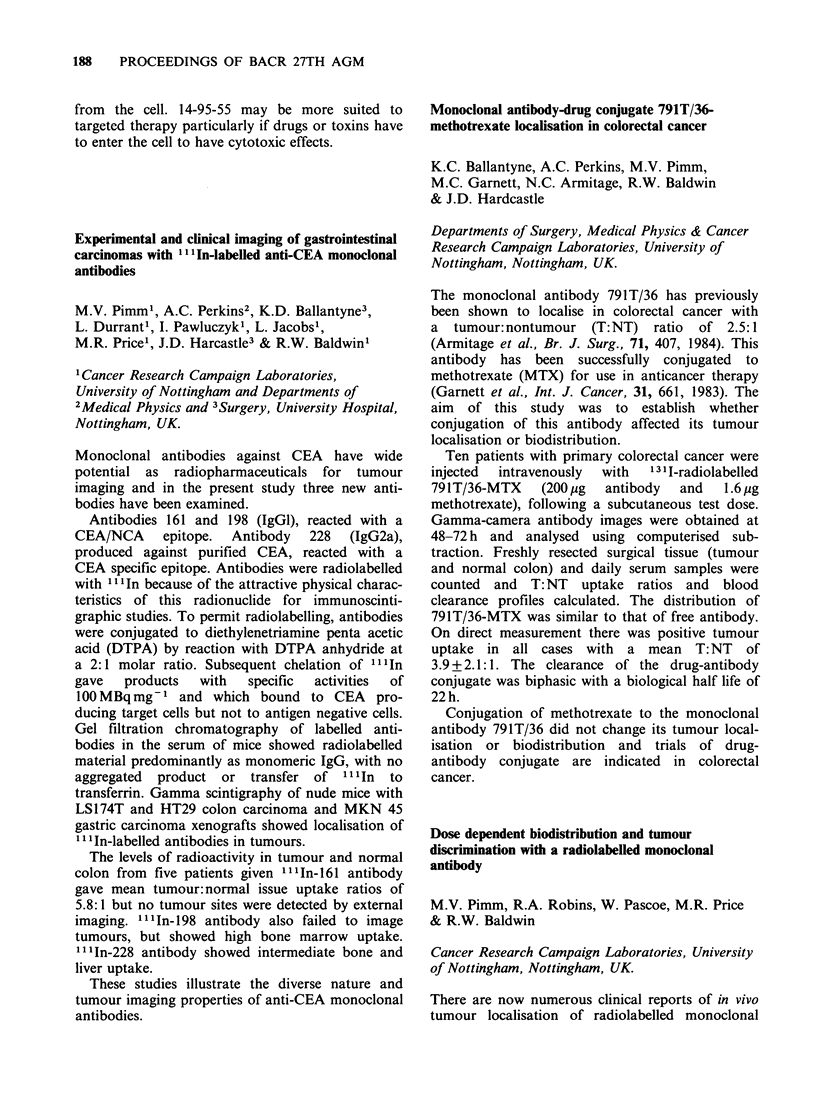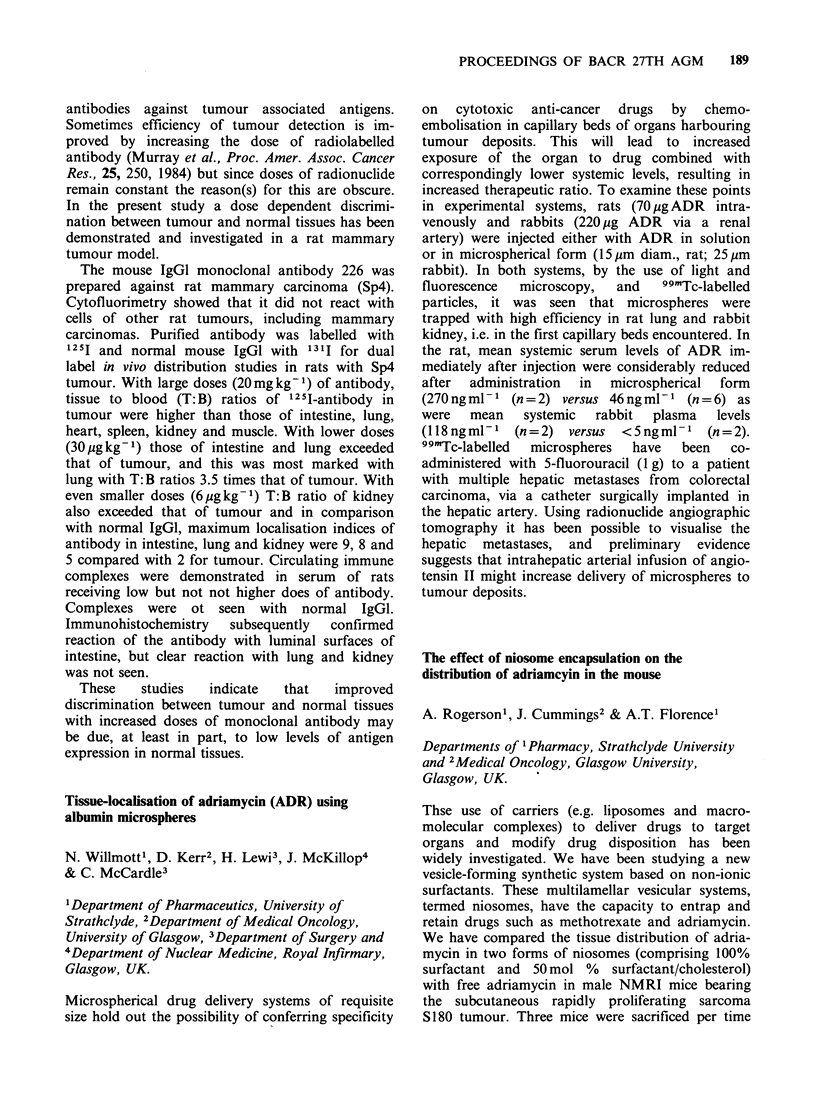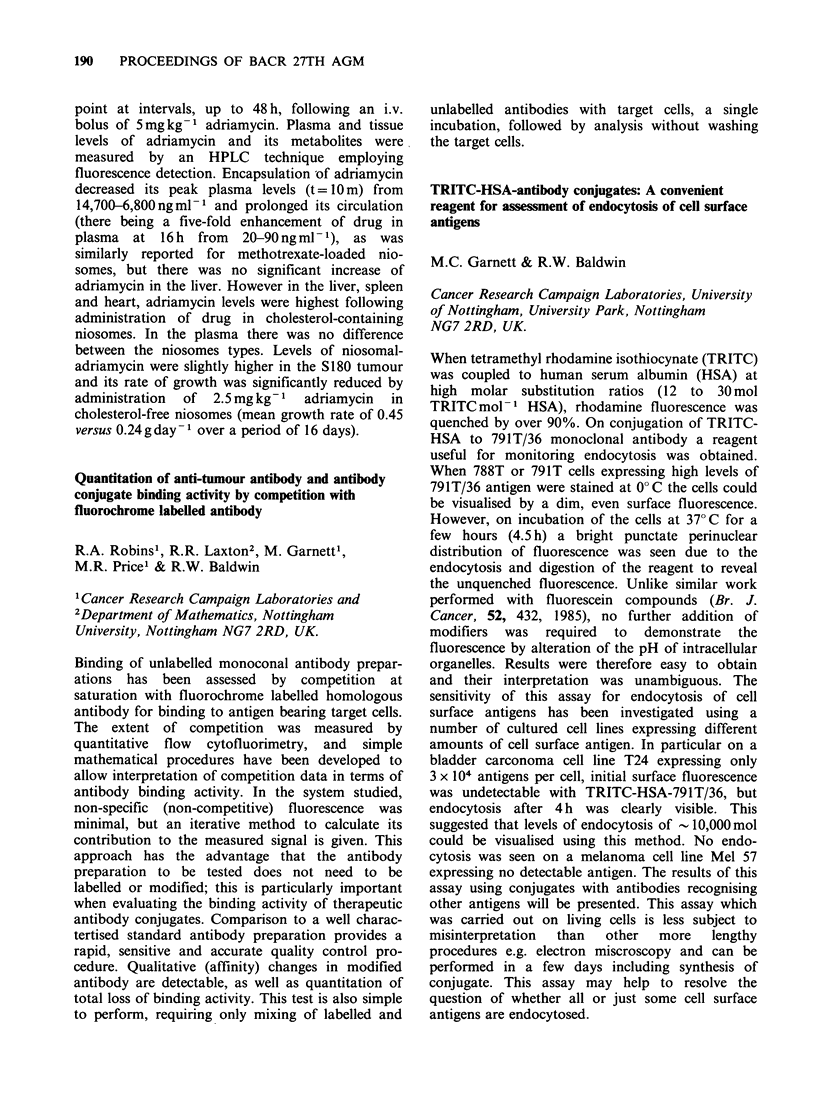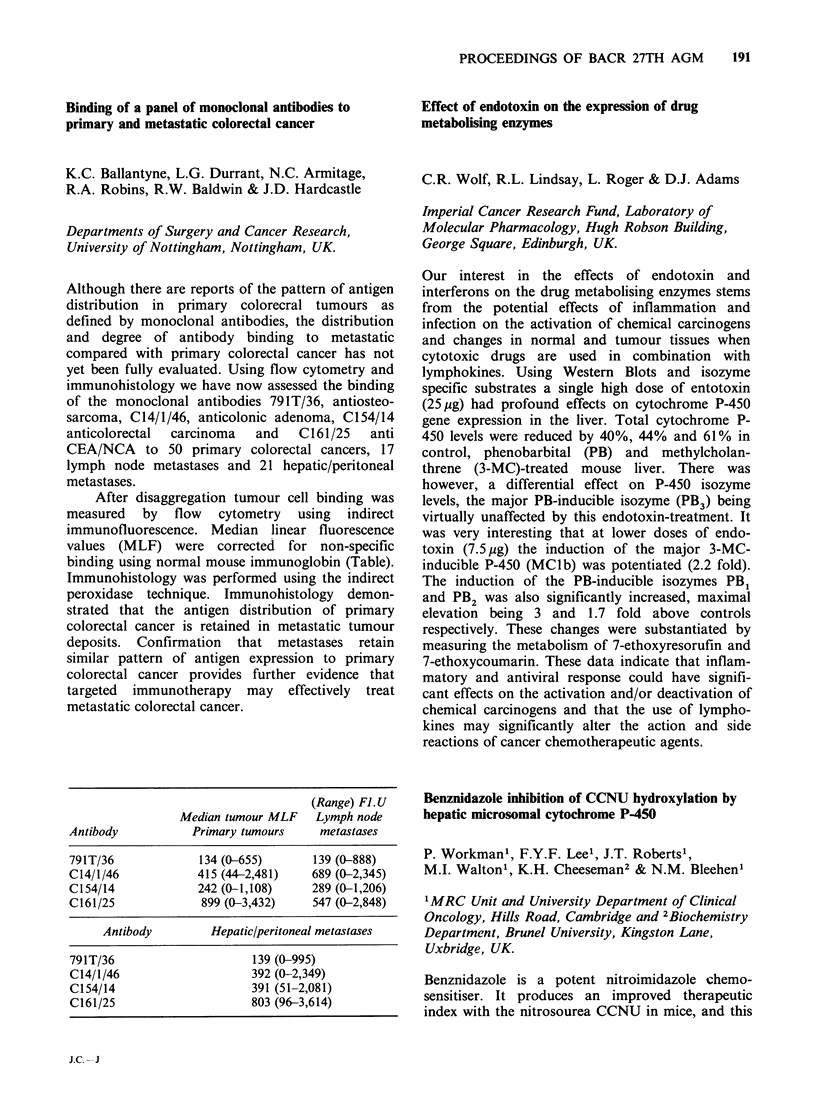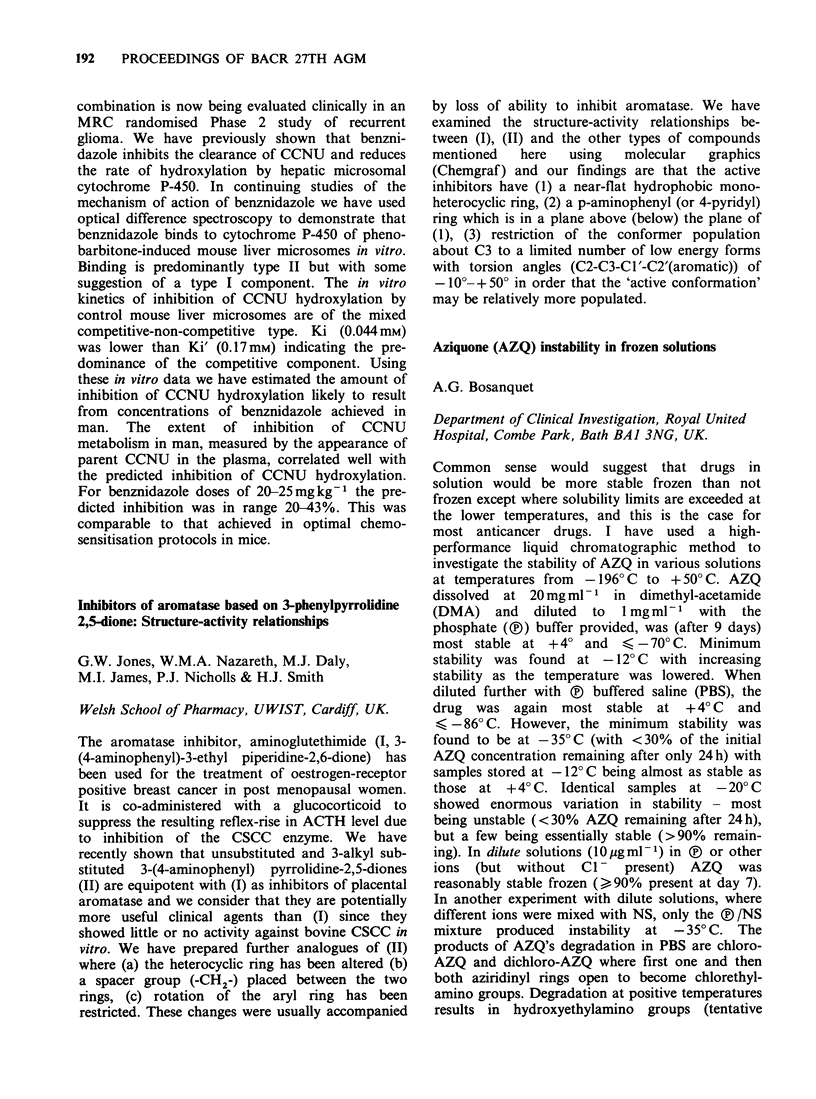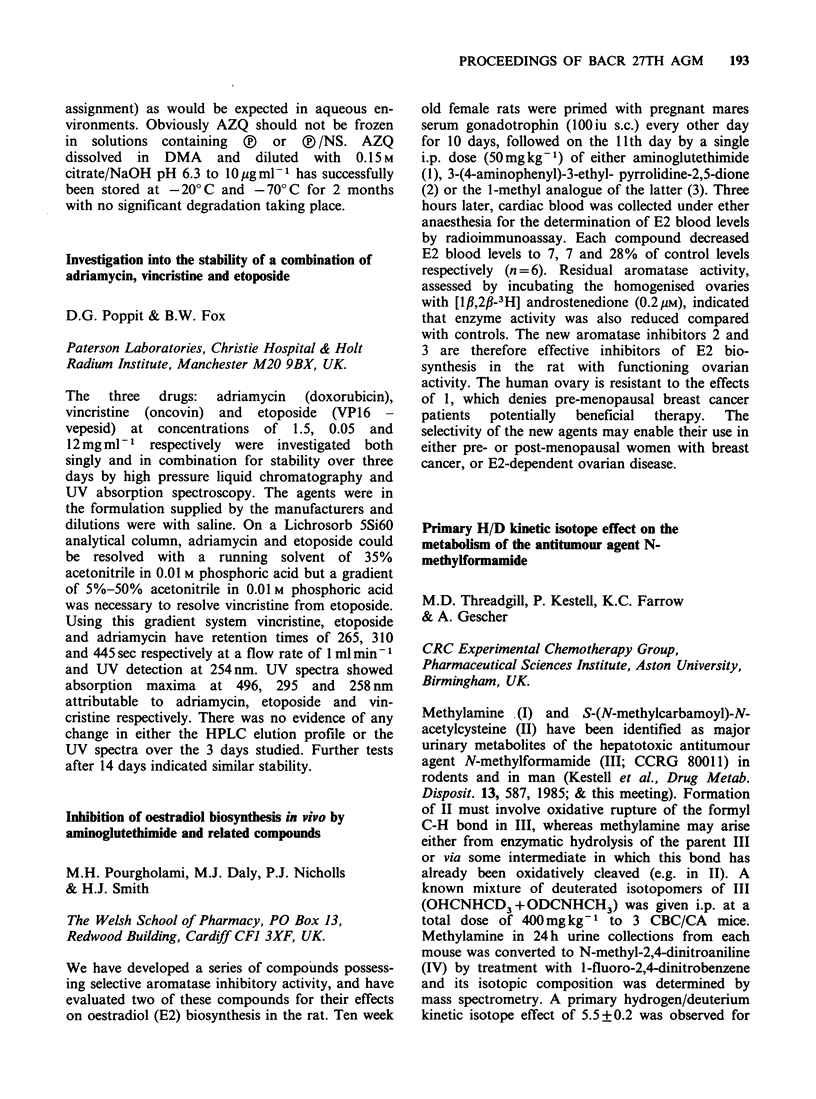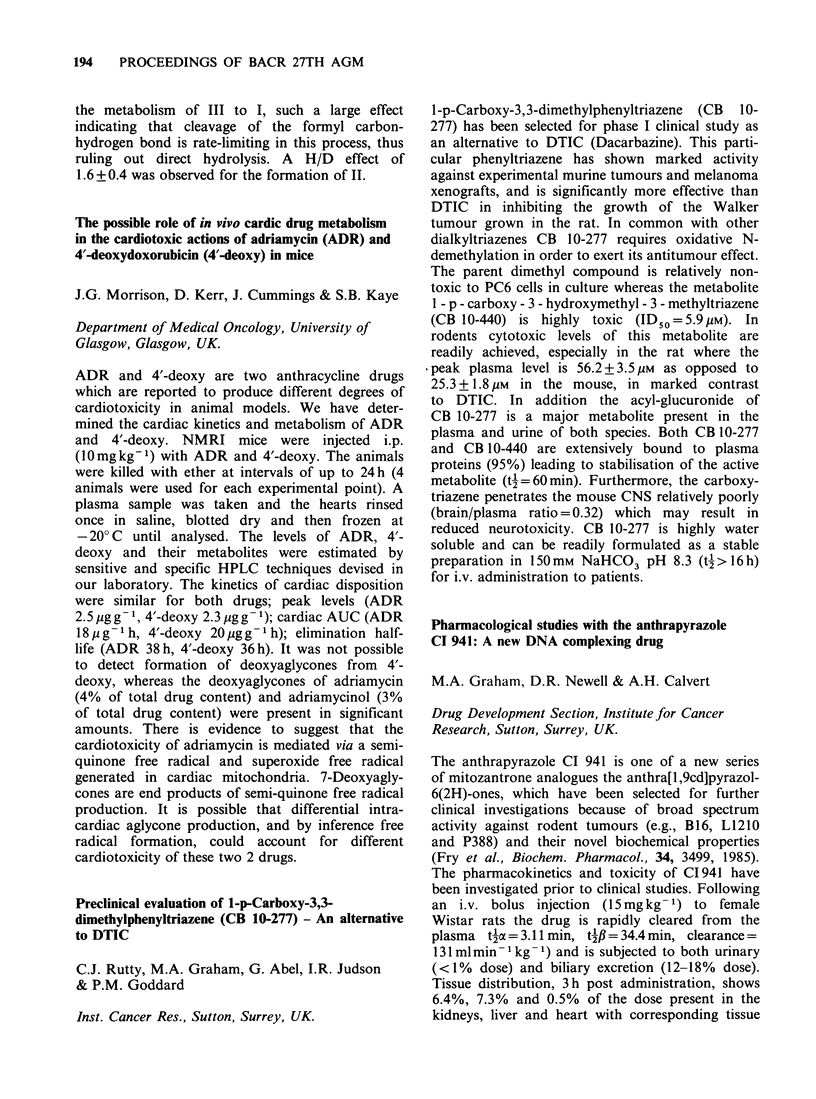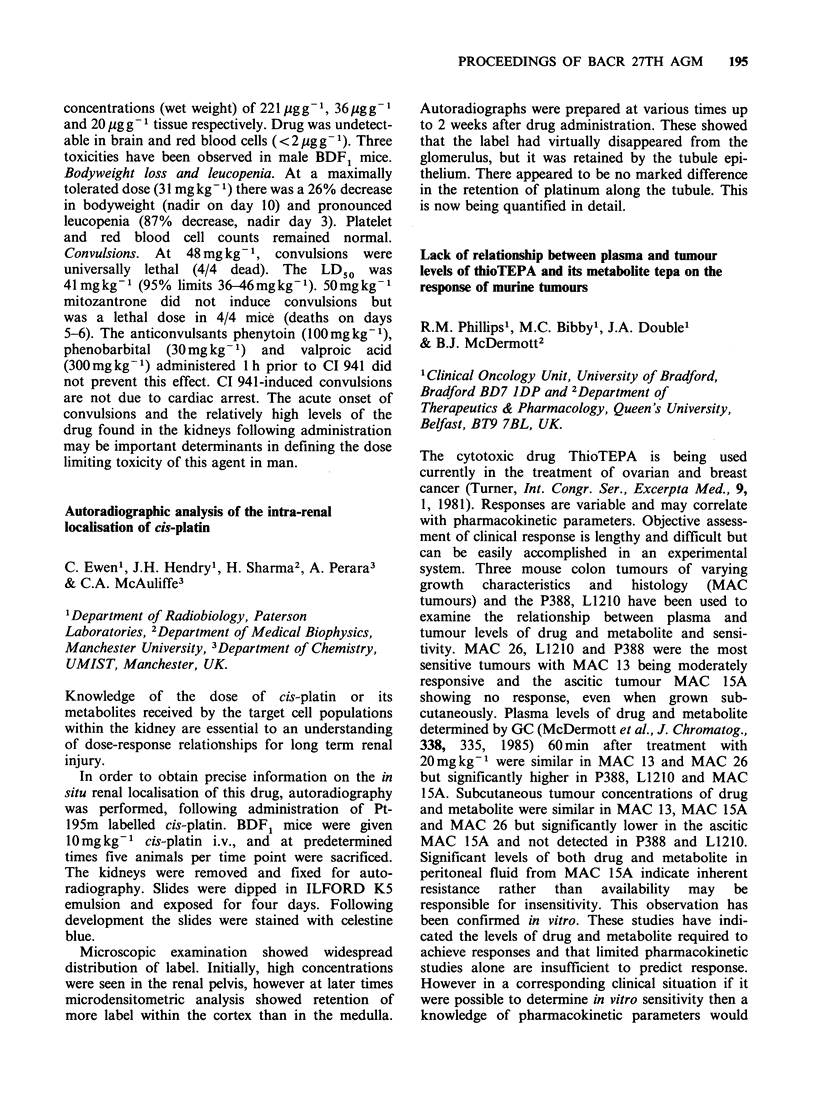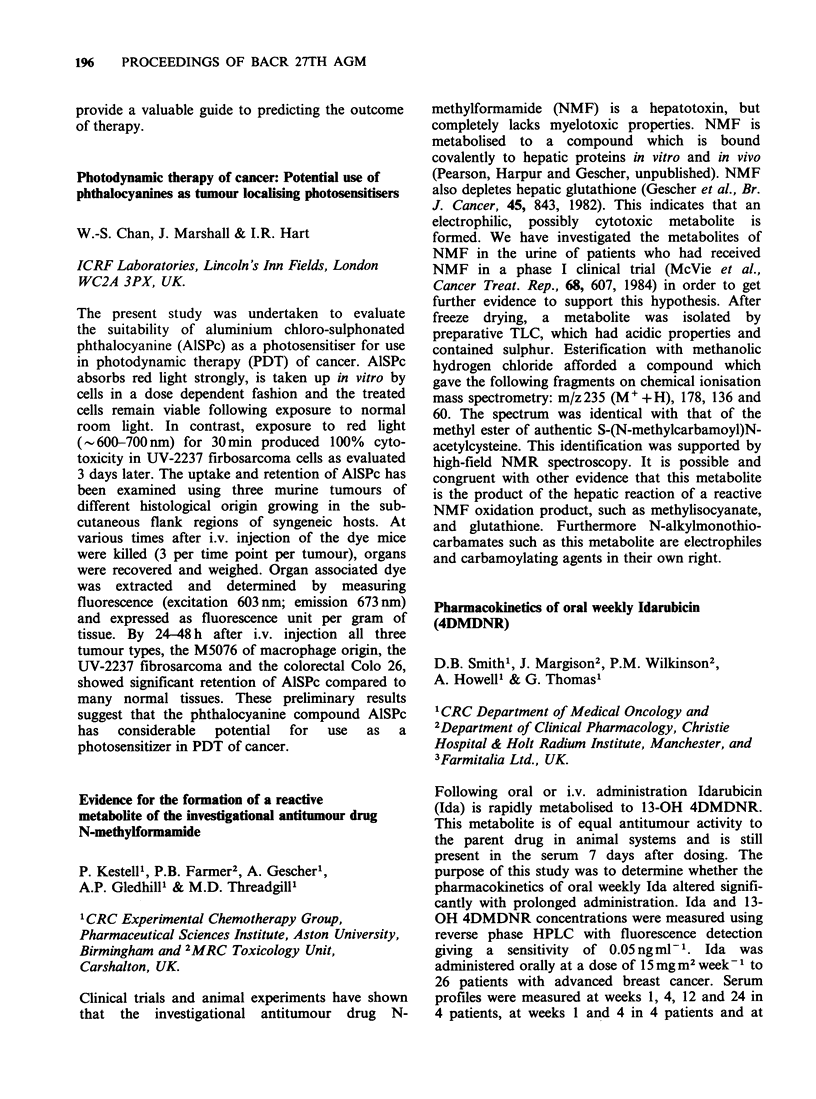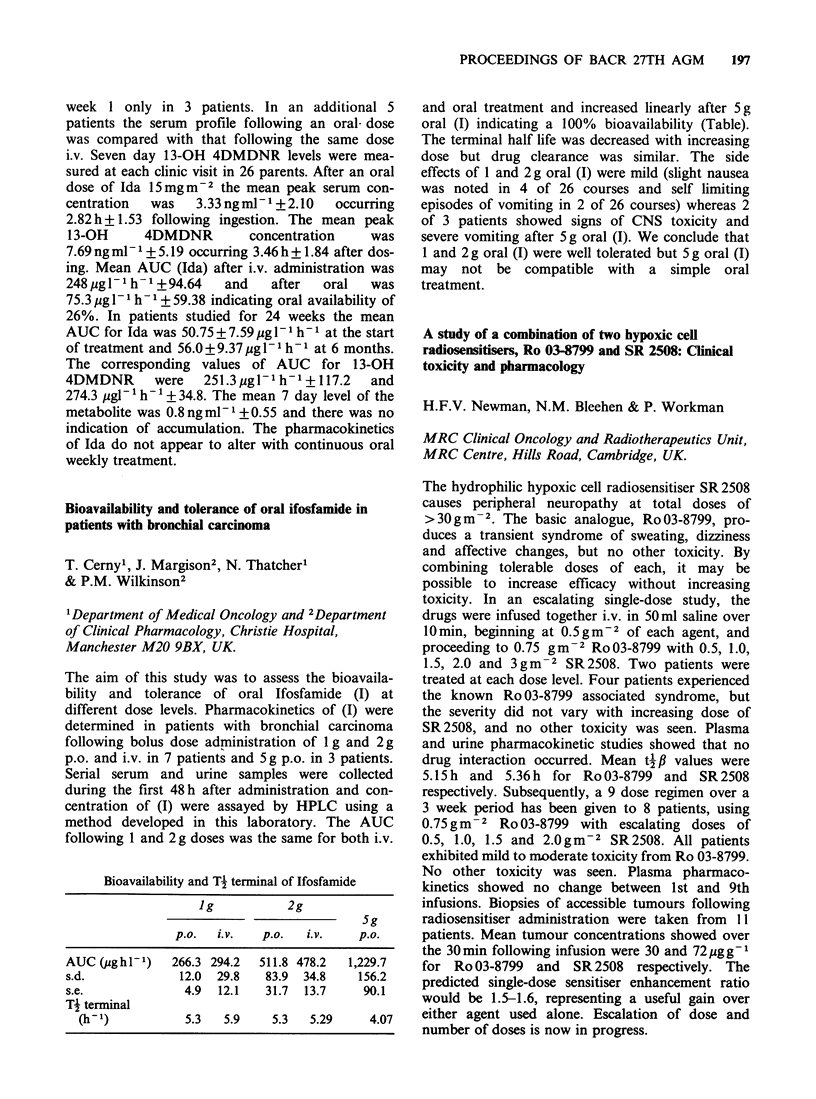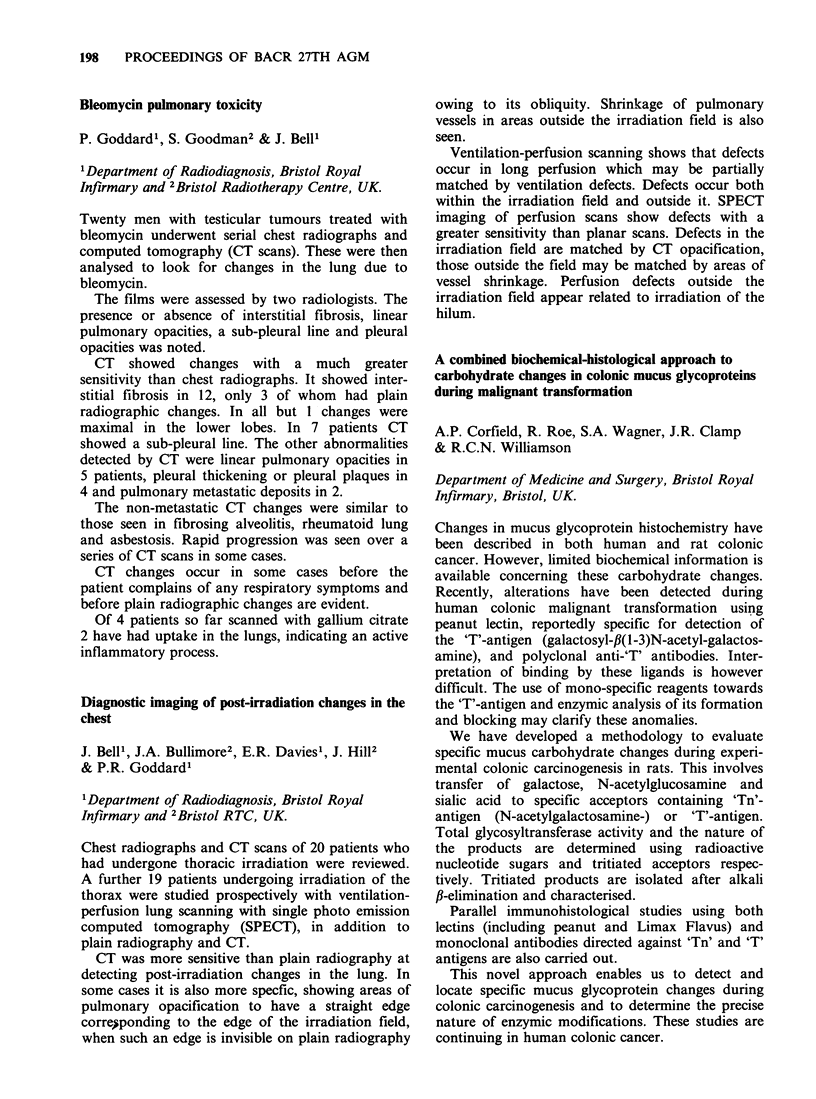# Twenty-seventh annual general meeting of the British Association for Cancer Research (in conjunction with the inaugural meeting of the Association of Cancer Physicians). March 24-26, 1986, Bristol, UK. Abstracts.

**DOI:** 10.1038/bjc.1986.164

**Published:** 1986-07

**Authors:** 


*Br. J. Cancer* (1986), **54**, 137–198

### Twenty-Seventh Annual General Meeting of the British Association for Cancer Research* (in conjunction with the Inaugural meeting of the Association of Cancer Physicians)

(Incorporating Symposia on 'Intestinal Carcinogenesis' and 'Epithelial cancers: Experimental and clinical approaches' and the 1986 Walter Hubert Lecture†). March 24–26, 1986.

*Held at the University of Bristol, UK*

### Abstracts of Invited Papers‡

### Symposium on 'Intestinal carcinogenesis'

### The role of heredity in intestinal carcinogenesis

B.C. Morson

*St. Mark's Hospital, London, UK.*

There is no evidence, *per se,* that colorectal cancer is inherited. On the other hand, there are indications that a genetic factor is involved in the appearance of the principal precursor lesion, the adenoma.

In familial polyposis coli, which is inherited as a dominant Mendelian character, the colorectal mucosa is covered by thousands of adenomas. It is important to decide what differences, if any, exist between the adenomas of polyposis and those occurring in non-polyposis patients. One obvious difference is that of number. Whereas in polyposis the average number of adenomas is measured in thousands there are usually fewer than twenty adenomas in non-polyposis patients. The figure of 100 adenomas is a practical one to use as a division between the two groups of patients. No differences have been detected in the clinical and histological characteristics of the adenomas occurring in the two groups and the resulting cancers show similar characteristics. The question therefore arises whether the adenomas in non-polyposis patients also have a genetic origin.

A family history has been elicited in 26% of colorectal cancer patients (Lovett, *Br. J. Surgery,* **63,** 13, 1976). The majority had only one affected relative but 8% were two case families and ~10% had three or more affected relatives. The average number of adenomas per patient (who also had cancer) increased with the number of affected family members. In contrast to polyposis coli the carcinomas in cancer families are accompanied by only small numbers of precursor adenomas, although on average significantly more than the numbers seen in association with sporadic colorectal cancer.

Woolf *et al.* (*Cancer,* **8,** 403, 1955) showed that the prevalence of adenomas in close relatives of colorectal cancer cases was 45% compared with only 5% in spouses.

In addition to adenomas determined by an autosomal dominant gene i.e. polyposis coli, there is the possibility that isolated adenomas could be produced by an autosomal recessive gene (Veale, *Intestinal Polyposis,* Eugenics Lab. Memoirs, Series **40,** 1965, CUP). Only a tiny proportion of human colorectal cancers are the consequence of autosomal dominant genetic disease, and an autosomal recessive gene could be far more important in terms of the numbers of cases for which it is responsible. In a population uniformly exposed to environmental agents the level of exposure to such agents might determine the incidence of bowel cancer, but genetic factors might determine which members of the population actually develop the disease.

All indications are that the factors causing adenomas differ from those causing the progression to carcinoma. Moreover, the factors causing the initial formation of an adenoma appear to differ from those which cause the adenoma to grow in size and to progress to carcinoma. Thus there are both genetic and environmental factors in the causation of adenomas and the genetic factors

*Enquiries to the BACR Secretariat, c/o Institute of Biology, 20 Queensberry Place, London SW7 2DZ, UK.
†This issue pp. 000–000.
‡Reprints of these abstracts are not available – Ed.

© The Macmillan Press Ltd., 1986

render some persons more susceptible than others to the environmental factors. A postulated mechanism for the aetiology of the adenoma-carcinoma sequence which integrates both genetic and environmental factors has been proposed (Morson *et al.*, *Cancer Surveys,* **2,** 451, 1983).

### Proliferation abnormalities in adenomas

E. Friedman & M. Lipkin

*Memorial Sloan-Kettering Cancer Center, Laboratory of Gastrointestinal Cancer Research and Gastroenterology Service, New York City, New York 10021, USA.*

Proliferative abnormalities define preneoplastic states in human colon, oesophagus, and stomach, as defined by an enlarged proliferative compartment using [$^3$H]-thymidine labelling as a marker. This abnormality is preserved in colonic adenomas which have an increased proliferative compartment, but have also lost some directional control over cell migration. Adenomas of the simple tubular class placed into tissue culture respond by mitogenesis to synthetic tumour promoters of the phorbol ester class. Similar responses occurred to diacylglycerols, which are found within the colon, bind to the TPA receptor, and thus can be considered endogenous tumour promoters. Normal colonic epithelial cells in tissue culture do not respond to tumour promoters by proliferation. More advanced adenomas of the villous class and carcinomas respond to synthetic and endogenous tumour promoters by secretion of a urokinase-type plasminogen activator.

### Intestinal carcinogenesis: The role of diet

K.W. Heaton

*University Department of Medicine, Bristol Royal Infirmary, Bristol BS2 8HW, UK.*

Geographical differences in colorectal cancer mortality point strongly to the life-style of 'western' countries. Popular theories blame high fat intake, low fibre intake and high cholesterol intake but the evidence for each is conflicting and incomplete. Several mechanisms exist whereby dietary fibre and undigested starch could be protective.

Case control studies can explain why, within a community, some people contract a disease and others do not. With colorectal cancer, case-control studies have given inconsistent results. Possible reasons include: (i) imprecise methodology for assessing dietary intake; (ii) failure to measure possibly important dietary factors, such as dietary fibre and refined sugar; (iii) loose matching of cases and controls, and (iv) inclusion of cancer patients whose dietary intake had changed as a result of their symptoms, e.g. anorexia, vomiting, weight loss. We have carried out a case-control study which was designed to avoid the last 3 problems. Despite imprecise methodology (the dietary history method using a food frequency questionnaire) we were able to show that, compared with healthy controls, 50 cancer patients ate a diet providing 16% excess calories ($P<0.001$). The excess came chiefly from fat and carbohydrate, and the biggest difference (41%) was in the intake of refined (fibre-depleted) sugar. Indeed, half the excess calories were attributable to sugar itself or to fat eaten in combination with sugar.

Extensive animal experiments have shown that a 20–30% decrease in calorie intake inhibits carcinogenesis. We have found that a 23–28% fall in calorie intake occurs unconsciously and without hunger when volunteers switch from a diet containing 110 g sucrose to one containing 5–10 g (*Gut,* **24,** 2, 1983; **25,** 269, 1984).

Dietary practices which inflate calorie intake and, specifically, the use of sucrose should be considered as increasing the risk of colorectal cancer.

### Intestinal carcinogenesis: Inflammation

J.E. Lennard-Jones

*Academic Unit of Gastroenterology, The London Hospital Medical College, London, UK.*

Two conditions predispose to malignancy in the small intestine; gluten-sensitive enteropathy and Crohn's disease. The former is perhaps not truly an 'inflammation' but there are many chronic inflammatory cells in the lamina propria and there is an increased rate of epithelial turnover. The commonest malignancy observed in coeliac disease is a lymphoma, derived from T cells, but adenocarcinoma also occurs. Crohn's disease of the small intestine is occassionally complicated by carcinoma, usually in long-standing disease, and occasionally in a segment of inflammation bypassed at operation.

The commonest, and clinically most important, inflammatory disorder predisposing to carcinoma is ulcerative colitis. The tumours tend to occur at a younger age than in the general population and may be multi-focal. The presence of carcinoma is usually associated with a patchy dysplastic change elsewhere in the large intestine. Dysplasia can be recognised in flat mucosa but often there is a proliferative component leading to a villous configuration, mucosal nodularity or the formation of a broad-based polyp. The occurrence of dysplasia enables a presumed pre-cancerous phase to be recognised on endoscopic biopsy, though when high-grade dysplasia is found a co-existing carcinoma is often present elsewhere in the colon. The risk of carcinoma in ulcerative colitis can be defined and it is greatest in patients with inflammation affecting most or all of the mucosa with a clinical history of at least 10 years. A cancer surveillance programme is being evaluated in this high risk group of patients.

There is an increased incidence of carcinoma in colonic Crohn's disease, in which dysplastic changes can also occur, but the risk at present does not appear great enough to warrant regular surveillance. The chronic anal lesions of Crohn's disease may also become the site of carcinoma.

There is a major carcinoma risk at the anastomotic site of ureteric implantation into the colon. An association between schistosomiasis and colonic carcinoma has been reported. Other inflammations such as diverticulitis do not appear to be associated with a neoplastic risk.

### Hyperplasia, the common thread

R.C.N. Williamson

*University Department of Surgery, Bristol Royal Infirmary, Bristol BS2 8HW, UK.*

Active cell proliferation is a prerequisite for the initiation of carcinogenesis and is likely to be important in tumour promotion. Hyperproliferative lesions can be identified in premalignant colorectal mucosa, e.g. in familial polyposis and in rodents exposed to chemical carcinogens. Diet affects cytokinetics throughout the intestinal tract either by a direct trophic stimulus or by altering bile acids and bacteria, which themselves influence cell turnover. Thus obesity could enhance the risk of colorectal cancer because hyperphagia increases the number of cells with a potential for malignant transformation. Crypt cell hyperplasia is also a feature of ulcerative protocolitis, which is undoubtedly premalignant. By measuring crypt cell production rate in cultured rectal biopsies we have found increased replication not only in active colitis but also in quiescent disease. Likewise, irradiation markedly disturbs intestinal cell turnover and there is an increased risk of rectal cancer developing many years after pelvic irradiation.

The impact of increased and decreased cell proliferation on colorectal neoplasia has been studied in rats receiving intestinal carcinogens (Williamson & Rainey, *Scand. J. Gastroenterol.*, **19**, Suppl. 104, 57, 1984). Resection of either proximal or distal small intestine leads to colonic hyperplasia and increased numbers of tumours in animals receiving azoxymethane or dimethylhydrazine. Intestinal bypass generally has the same effect. Indeed, the trebling of colorectal tumour yields in rats receiving 85–95% jejunoileal bypass is disquieting in view of the large numbers of patients who have had this operation performed for morbid obesity. Partial colectomy only promotes carcinogenesis at the site of anastomosis, possibly because its adaptive effects are slight. The finding that a diverting colostomy protects against tumour development is consistent with the hypothesis linking hyperplasia and neoplasia, since defunctioned colon undergoes atrophy. When instilled repeatedly per rectum, the secondary bile salt sodium deoxycholate causes both local hyperplasia and a sharp increase in susceptibility to cancer. The same substance delivered into an isolated loop of colon (Thiry-Vella fistula) is unable to affect either the hypoplasia of defunction or the concomitant resistance to a systemic carcinogen. Reintroducing faeces into the Thiry-Vella fistula largely reverses these effects.

The prevalence of suture-line cancers is a consistent feature of these experiments. Intestinal anastomoses remain at increased risk of carcinogenesis even when fashioned up to 12 weeks before the first injection of carcinogen. Our recent data suggest that the anastomosis remains a focus of increased proliferative activity throughout this period. Perhaps some instances of 'recurrence' at the intestinal anastomosis in patients surviving colectomy reflect metachronous carcinogenesis in an area of long-standing hyperplasia.

### Symposium on 'Epithelial Cancers: Experimental and Clinical Approaches'

### Gastric cancer; cause, precancer and early cancer

N.J.McC. Mortensen

*University Department of Surgery, Bristol, UK.*

Genetic factors may be implicated in only a small proportion of gastric cancer patients. Migrant studies and a worldwide decline in incidence suggest that environmental factors are more important. Dietary nitrosamines, hypochlorhydria, duodenogastric influx and histological changes in gastric mucosa combine to result in a gastric cancer.

Patients who have previously had a partial gastrectomy for peptic ulcer are at increased risk of developing carcinoma some 20 years or so after their original surgery. They provide a unique human model for the study of the evolution of invasive cancer. After a gastrectomy the intragastric acidity falls, the remnant becomes colonised by bacteria, there are increased levels of nitrosamines and bile acid levels are high from free influx. We have studied a group of 63 partial gastrectomy patients 20 years after surgery by repeated endoscopy and biospy over 6 years (Mortensen *et al.*, *Br. J. Surg.*, **71,** 363, 1984) carefully grading histological changes for intestinal metaplasia chronic atrophic gastritis, and dysplasia. Although 20% had moderate or severe dysplasia, no patients have so far developed invasive cancer. Histological changes correlate with reflux, intragastric bile acids and nitrite levels (Thomas *et al.*, *Scand. J. Gastroenterol.*, **19,** Suppl. 92, 195, 1984). Similar histological changes have been described in pernicious amaemia patients, another high risk group. Whilst these changes may be premalignant they progress very slowly and the precise mechanisms of carcinogenesis are still not known.

In Japan 30% of all gastric cancer cases are early gastric cancers EGC with an 80–100% 5 year survival. Until recently the incidence of EGC in this country was low. We reviewed 35 cases diagnosed between 1965 and 1985. Twice the number were seen in the second decade compared with the first suggesting a rising incidence probably as a consequence of improved diagnostic surveillance. Life table survival curves give an age adjusted 5 year survival of 92% in these patients (Houghton *et al.*, *Br. Med. J.*, **291,** 305, 1985).

An increasing understanding of the natural history of premalignant histological lesions in the stomach together with more frequent diagnosis of EGC will result in an improved prognosis for gastric cancer.

### Surgical and adjuvant treatment for gastric cancer

J.L. Craven

*York District Hospital, York, UK.*

There exists a wide and poorly understood variation in the results of treatment of gastric cancer with particularly larger differences in the outcome of therapy seeming to exist between Japan and the rest of the world. There is little evidence to support the view that these differences in the result of treatment are due to undefined differences in the biological behaviour of the tumour. Comparisons of early gastric cancer between Japan and the Western world reveal many areas of similarity. It appears that much of the Japanese success in treatment lies in their methodological approach in which macroscopic staging at laparotomy, precisely defined surgical techniques and microscopic staging of the resected tumour permit comparison between surgeons, hospitals and regions, Miwa's data (*Garn,* **22,** 61, 1979) provides validification of their macroscopic and microscopic staging techniques.

The commonest cause of death after resection of gastric cancer is recurrent disease within the gastric bed and it is clear that an effective method of locoregional control would have very great survival benefit. The application of total gastrectomy *de principe* to the resection of gastric cancer has not been tested by a controlled trial, but it does appear that there will be many patients whose treatments fail, even when the surgical regime is optimum and these treatment failures have suggested a role for adjuvant chemotherapy. Though single chemotherapeutic agents have been shown to have some effect upon the tumour, none of the adjuvant trials, either with single or combination agents, have been shown to have any survival benefit. This should not give rise to too much pessimism. We may have been expecting too much. Surgical treatment and operative staging have not been standardised in any of the studies and benefit from adjuvant chemotherapy will be felt only by those at risk of recurrence. Single agent response rates are rarely more than 25% and the beneficial anti-tumour effect will be diluted by any excess of patients whose resections alone have not been curative. Much remains to be learnt of the effect of modifying the combinations, their dosages and duration of treatment.

Radiation has only been used infrequently as a treatment for gastric cancer, but it has been shown to have therapeutic potential in a small proportion of patients and some benefit also occurs in advanced disease when used with chemotherapy. Gunderson's reoperation data show that most of the local recurrences can be encompassed by a conventional radiotherapy portal and suggest a logical role for radiotherapy as an adjunct to surgery (Gunderson & Sosin, *Int. J. Radiol. Oncol. Biol. Phys.*, **8,** 1, 1982). In an attempt to improve its efficiency the use of chemotherapy in combination has been tried with some modest success. Radiosensitisers have been investigated, but none without toxic side effects has yet been identified. Intraoperative radiotherapy (IORT) was first used by Abe and his encouraging, but uncontrolled results have stimulated considerable interest in North America where several studies of this modality are now proceeding (Abe & Takahashi, *Int. J. Radiat. Oncol. Biol. Phys.*, **7**, 863, 1981).

In the West, benefits in the treatment of gastric cancer will most quickly follow the application of defined surgical procedures applied after careful macroscopic staging and validated by careful pathological staging. Until those prerequisites are met, it is fruitless to explore the benefit of what may be necessary adjuncts to surgical treatment.

### Aetiology of pancreatic cancer

K.G. Wormsley

*Ninewells Hospital, Ninewells, Dundee DD1 9SY, UK.*

Epidemiological analyses of the geographic and demographic aspects of pancreatic cancer (including geographic distribution, population group incidence rates, migrant incidence rates and changes with time) indicate that environmental carcinogens play an important aetiological role in pancreatic carcinogenesis (Wormsley, *Ital. J. Gastroenterol.*, **17**, 102, 1985). Unfortunately, until recently, satisfactory direct techniques for identifying such pancreas-specific carcinogens were not available. Additional indirect information about the pathogenesis of pancreatic cancer has been obtained from study of associated or predisposing diseases and from analysis of the dietary habits of affected individuals. More important and related pointers to the causes of pancreatic cancer have been derived from animal experiments (Longnecker *et al.*, *Int. Rev. Expie. Pathol.*, **26**, 177, 1984). For example, it has been possible to identify chemicals with apparently pancreas-specific activity as genotoxic carcinogens. These agents often require metabolic conversion by the pancreatic acinar cells before reacting with DNA. Even more interestingly, it has been possible to show that dietary modulation greatly sensitises the rat pancreas to the carcinogenic effects of some of the genotoxic chemicals (McGuinness *et al.*, *Environ. Health Perspect.*, **56**, 205, 1984). The dietary sensitisation, with raw soya flour, seems to be dependent on the stimulation of pancreatic growth. The dietarily 'sensitised' pancreas provides an excellent model for the prospective screening of environemntal carcinogens; for analysing the processes involved in pancreatic carcinogenesis; and for testing new therapeutic agents. These findings also have implications for man, since the human pancreas seems functionally similar to the pancreas of rats.

### The dilemma of treatment

R.A. Malt

*Department of Surgery, Harvard Medical School, Boston, Massachusetts 02114, USA.*

(By title only)

### Ovarian cancer – Studies with monoclonal antibodies

G.M. Stirrat

*Department of Obstetrics & Gynaecology, University of Bristol, UK.*

(By title only)

### Chemotherapy of ovarian cancer

J.G. McVie

*The Netherlands Cancer Institute, Antoni van Leeuwenhoekhuis, Amsterdam, The Netherlands.*

There are four milestones in the recent history of chemotherapy in ovarian cancer. First was the realisation that median survival of 10 months with single alkylating agents could be more than doubled by addition of other agents, such as hexamethylmelamine, 5-fluorouracil, doxorubicin. Coupled with the claims that combination chemotherapy was superior to single agent came the increased awareness that response criteria differed widely from series to series and that reliance on clinical observations alone was totally inadequate. Thus the complete remission *pathologically proven* became the standard for reporting of results.

A third improvement was the realisation that cisplatin was the single most active agent and the fourth was that there was a clear dose response relationship for *cis*-platin not only in clonogenic assay systems in the lab but also in patients. A large randomised trial has now shown clear superiority not only in response rate but also in median survival and long term survival between a platinum containing regimen *versus* a non-platinum containing regimen (CHAP *versus* Hexacaf). The first really long term results for platinum regimes are now being reported and in selected cases a 20% 7 years' disease free survival has been achieved. The latest controversy in this area has been the question of the use of drugs in addition to platinum *versus* platinum in high dose as a single agent. The latter is one of the most toxic regimes ever used. This has led to the development of analogues of the drug which are less toxic and carboplatin seems to be a good candidate. This drug is already in a far advanced stage of development and up until now head on comparative studies in ovarian cancer of carboplatin with *cis*-platin show little difference in antitumour effect. There is almost no nephrotoxicity from carboplatin and it does not require aggressive in-patient hydration schemes; neurotoxicity seems to be a problem only in patients previously treated with *cis*-platin.

Reassessment of i.p. *cis*-platin is now underway, intended to increase the dose of drug reacting with the i.p. tumour with a lower concentration in distant organs. This approach has been particularly successful in small volume 'minimal residual disease' resistant to i.v. *cis*-platin. In our own series we have achieved 30% pathologically proven complete remissions in such patients, whereas with conventional therapy we have yet to see a response after failure of 6 months of i.v. *cis*-platin. Toxicity is not negligible and neurotoxicity remains the dose limiting toxicity. Nephrotoxicity and myelosuppression can to some extent be averted by concomitant i.v. sodiumthiosulphate (STS) which neutralises some of the *cis*-platin absorbed from the peritoneal space. Howell and colleagues have developed this idea further and have given STS with i.v. *cis*-platin. There remains considerable controversy in this area due to the theoretical likelihood of rescuing tumour from the effect of *cis*-platin by complete neutralisation. It must be said, however, that there are occasional reports of anti-tumour effect and toxicity does seem to be slightly less. It is to be hoped that a combination of new analogues of *cis*-platin and alkylating agents, maximal debulking surgery, and, when appropriate, local i.p. therapy that the complete remission rate currently achieved at 30% will eventually reach 50% accompanied by tolerable toxicity for the patient.

### Bladder cancer: Late promotional stages in relation to causes and control

R.M. Hicks

*School of Pathology, Middlesex Hospital Medical School, Riding House Street, London W1P 7LD, UK.*

Human bladder cancer presents in two distinct forms, with differing developmental pathways, behaviour patterns and associated mortalities. A minority of cases, ~10%, at first presentation have rapidly invasive, solid transitional cell carcinoma (TCC) which can arise over a wide front below flat carcinoma-in-situ of the urothelium. Most patients, however, present with recurrent, well-differentiated papillary tumours which may not become invasive for many years. Both disease patterns can now be reproduced experimentally in rodent models.

There is good evidence that the papillary form of TCC develops step-wise, by a process analagous to multi-stage carcinogenesis in the mouse skin. The rate at which each papillary tumour develops from an initiated preneoplastic urothelial cell is critically affected by late stage promoting or enhancing factors which, though they may not be initiating carcinogens *per se,* increase the incidence (age-related prevalence) of human TCC. This suggests that anti-promoting agents might reduce the bladder cancer incidence in 'at risk' populations.

We have demonstrated that certain retinoids, known to be anti-promoters in the mouse skin carcinogenesis model, do indeed delay the development and thus reduce the incidence of papillary TCC in the N-butyl-N-(4-hydoxybutyl)nitrosamine (BBN)-treated F344 rat bladder cancer model. By contrast, the BBN-treated B6D2Fl hybrid mouse develops both papillary TCC and a high incidence of poorly differentiated solid invasive TCCs. The latter do not appear to develop by the same multi-stage mechanism, involving promotion and clonal expansion of promoted cells, as do the papillary tumours. Moreover in this model, retinoid treatment does not reduce the incidence of the rapidly growing solid cancers.

Our data suggest that there is scope for improving the management of the patient with papillary TCC by delaying or preventing recurrences with agents which block the post-initiation stages of cancer development; further development work is necessary to design less toxic and more effective anti-promoting agents. The data do not suggest that anti-promoting agents such as retinoids can be used therapeutically to treat either flat invasive TCC or papillary tumours which are growing autonomously; the latter are biologically 'late' tumours which have already passed through the promotional stages of carcinogenesis.

### A multidisciplinary approach in the diagnosis and treatment of bladder cancer

A.T.K. Cockett

*Department of Urology, University of Rochester, NY, USA.*

Since 1976, porcine sensitised lymph node cells – tumour immune cells – have been employed by our group to treat selected cases of infiltrative transitional cell bladder cancer. The continued monitoring of patients with recurrent bladder tumour has necessitated the refinement of diagnostic techniques in order to stage beforehand the extent of tumour involvement, and to evaluate the response following treatment.

Downstaging of bladder tumour has provided a new opportunity to employ a second protocol to treat superficial, multifocal bladder tumours. Our presentation will also include early results (2 years) of treatment with the combination of Bacillus Calmette Guerin (BCG) and Interleukin 2.

Ambulatory screening of all bladder tumour patients now involves our Department of Pathology who use Multidimensional Slit-scan lasers for detection of bladder cancer. Correlation of our preliminary results in the 15 patients treated with BCG and IL2 are excellent and will be briefly discussed.

Accurate staging of bladder cancer has provided a continuing challenge to the urologist. We modified the technique for CAT scanning of the urinary bladder in all cancer patients entering both of our treatment protocols. Our diagnostic and cytopathologic results will be correlated with the patients' therapeutic responses.

### Abstracts of members' proferred papers

### A mouse monoclonal antibody against spontaneous rat mammary carcinoma Sp4

E.W. Pascoe, R.A. Robins, M.R. Price & R.W. Baldwin

*Cancer Research Campaign Laboratories, University of Nottingham, Nottingham NG7 2RD, UK.*

Monoclonal antibody (MoAb) 226 was raised against Sp4, a spontaneous rat mammary carcinoma of WAB/Not strain origin to attempt to provide a syngeneic animal/tumour system for the experimental application of MoAb mediated targeting.

BALB/c mice were immunised with cultured Sp4 cells and immune spleen cells fused with mouse P3NS-I myeloma cell line. Resulting hybridoma supernatants were screened using an ELISA. The MoAb was tested by flow cytometry against a number of tumour lines including other spontaneous mammary carcinomas and binding proved to be specific for Sp4. This was subsequently confirmed using an immunoperoxidase technique on frozen sections of WAB/Not tumours and normal tissues. The only cross reaction observed was against some luminal element found in sections of normal gut. Biochemical characterisation of the MoAb 226 defined antigen by SDS PAGE analysis of $^{125}$I-labelled immunoprecipitate showed it to be a single chain glycoprotein with an apparent mol. wt of 94 kD containing some sialic acid. The MoAb was isotyped as IgGl and has been successfully purified using Protein A Sepharose column chromatography.

### An immunohistochemical study of ovarian carcinomas

F. Macdonald[1], R. Bird[1], H. Stokes[1], B. Russell[1] & J. Crocker[2]

[1]*Surgical Immunology Unit, Queen Elizabeth Hospital and* [2]*Department of Histopathology, East Birmingham Hospital, Birmingham, UK.*

The immunohistochemical characterisation of ovarian tumours of the major pathological types has been carried out to determine if any antibody or mixture of antibodies is of value either in the histochemical diagnosis or in the localisation or therapy of these tumours.

Tumour types studied included tumours of epithelial origin (serous, mucinous) germ cell tumours (teratomas, dysgerminomas) and tumours arising from gonadal stroma (granulosa cell, thecoma) as well as undifferentiated and benign tumours and normal ovarian tissue. Between 1 and 7 blocks were obtained from each tumour. Antibodies investigated were anti-CEA (11-285-14), HMFG1, HMFG2, CA125 and CA19-9. Tumour sections were labelled by the indirect immunoperoxidase or avidin biotin techniques. No single antibody or pattern of antibodies was specific for any histological type of tumour. Undifferentiated tumours were unreactive with all. HMFG1 reacted with the majority of tumours, both malignant and benign but is also cross reactive with normal tissues. As in previous studies anti-CEA antibodies were reactive primarily with mucinous tumours (70%). CA19-9 reacted strongly with mucinous tumours (80%) and serous tumours (88%) whereas CA125 reacted mainly with serous tumours (65%). The less frequently diagnosed forms of ovarian tumours showed no particular pattern of reactivity although the number of patients available for study was small.

CA19-9 and CA125 were frequently found to be complementary to one another. Subsequent FACS analysis with these antibodies on an ovarian cell line suggests that the production of the antigens recognised by these antibodies is cell-cycle related.

The use of the two antibodies together appears to be a useful combination for immunolocalisation of ovarian tumours.

### Expression of Leu 7 antigen on human small cell lung cancer cells

F.G. Hay[1], S.G. Allan[2] & R.C.F. Leonard[1]

[1]*University Department of Clinical Oncology and* [2]*Imperial Cancer Research Fund Medical Oncology Unit, Western General Hospital, Edinburgh, UK.*

Two recent articles (Bunn *et al.*, *Blood*, **65**, 764, 1985; Cole *et al. Cancer Res.*, **45**, 4285, 1985) have reported the expression of Leu 7 antigen on small cell lung cancer (SCLC) cultured cell lines and paraffin embedded tissues from SCLC patients. The latter workers, finding low expression in human tissues, speculated that the Leu 7 expression seen in cell lines was compatible with Leu 7 being a differentiation antigen. However, neither group examined the activity of Leu 7 antibody with freshly obtained tumour cells from SCLC patients. We have studied the expression of Leu 7 antigen in human SCLC tumour cells in cell lines and freshly obtained aspirates from patients. Four SCLC cell lines were assesssed by flow cytometry and by PAP immunohistochemistry for Leu 7 expression (Becton Dickinson, HNK1). All 4 cell lines gave positive results by both methods. The expression of Leu 7 antigen in fresh, aspirated tumour cells was measured on air dried, acetone/methanol fixed preparations obtained from pleural effusions (4), bone marrow (5), lymph node aspirates (6) and disaggregated solid tissue biopsies (6) using a PAP method. We also reviewed formalin fixed, paraffin embedded tissue sections. Of the freshly obtained tumour samples, 18/21 (86%) expressed Leu 7 activity, in contrast only 2/27 (7%) of fixed tissues were positive. Heterogeneity of staining was observed with 25–90% of tumour cells expressing the antigen. We conclude therefore that fresh SCLC tumour cells do exhibit strong Leu 7 expression and that reactivity is similar in both *in vitro* (cell line) and *in vivo*. Reduced activity in paraffin embedded samples may reflect processing effects not counteracted by prolonged antibody exposure or by enzyme pretreatment.

### Epithelial and neural antigens in human small cell lung cancer (HSCLC)

F.G. Hay & R.C.F. Leonard

*University Department of Clinical Oncology, Western General Hospital, Edinburgh, UK.*

Despite intensive treatment-orientated clinical research, the origin and lineage of HSCLC remains uncertain. Important information has been obtained from HSCLC-derived cell lines that points to the heterogeneity of the tumour cells (in terms of biochemistry, immunological phenotype and genetic character) as well as suggesting a relationship between certain of the cell line characteristics and clinical behaviour. We have studied freshly obtained tumour cells and cell lines using a library of lineage-associated monoclonal antibody (MoAb) markers to compare *in vivo* tissue with cell lines with the aim of characterising HSCLC. The data (Table) were obtained by applying mouse anti-human MoAbs to air dried, acetone/methanol fixed preparations of cultured cell lines, freshly aspirated cells or disaggregated biopsies, followed by immunoperoxidase staining (PAP).

| | *Epithelial* | | |
|---|---|---|---|
| *MoAB* | $HMFG_1$ | $HMFG_2$ | *AUAI* |
| Fresh tumour cells | 17/20 | 21/21 | 12/12 |
| Cultured tumour cells | 4/4 | 4/4 | 3/4 |

| | *Cytokeratin* | | *Neural* | |
|---|---|---|---|---|
| *MoAb* | *CAM 5.2* | *LE61* | *534F8* | *UJ13A* |
| Fresh tumour cells | 11/11 | 21/21 | 21/21 | 8/10 |
| Cultured tumour cells | 3/4 | 3/4 | 4/4 | 4/4 |

We found that the majority of cell lines and fresh tumour tissues expressed epithelial and neural antigens simultaneously and there was also co-expression of low mol. wt cytokeratins.

These results support the hypotheses that dual lineages are present or that the progenitor cells differentiate along 2 different pathways in HSCLC.

The reactivity pattern of the anti-epithelial and neural antibodies encourages us to use them in the detection of marrow involvement by SCLC with consequent therapeutic implications.

**The distribution of DD9-E7 in non-pancreatic carcinomas**

Eadie Heyderman[1], Sandra Larkin[1], R.J. Whittaker[1], J. Hermon-Taylor[2] & Anne Grant[2]

[1]*Department of Histopathology, UMDS, St. Thomas Hospital, London SE1 7EH and* [2]*Department of Surgery, St. George's Hospital, London SW17 0RE, UK.*

The monoclonal antibody DD9-E7, raised by immunisation of BALB/c mice with a GER pancreatic adenocarcinoma xenograft, has previously been shown by us to react with all 22 adenocarcinomas of the exocrine pancreas, and with a variety of other normal and neoplastic tissues, including polymorphs and macrophages. One hundred and eighteen non-pancreatic tumours were selected from our surgical files and an indirect immunoperoxidase technique was used to see whether DD9-E7 might be a useful addition to a panel of antibodies used for the identification of the site of origin of a metastasis from an occult primary carcinoma. Positive staining was seen in 9/10 bronchial adenocarcinomas, 5/10 breast tumours, 8/10 gastric carcinomas, 12/12 colorectal, 2/5 endometrial, 5/6 cervical adenocarcinomas, 5/5 mucinous and 0/5 serous papillary adenocarcinomas of the ovary, 7/10 salivary gland adenocarcinomas, and in occasional cells in 3/5 papillary and in 2/5 follicular carcinomas of the thyroid. Two of 10 prostatic carcinomas were positive only in foci of squamous metaplasia, and only keratotic foci were positive in 25 primary skin carcinomas. It would appear that DD9-E7 could be used to distinguish metastatic pancreatic carcinomas from deposits of breast, serous ovarian, endometrial, thyroid and prostatic carcinoma (although thyroglobulin and prostatic acid phosphatase are more specific markers for thyroid and prostatic tumours), but not from bronchial adenocarcinomas. Pancreatic tumours are often only focally positive for CEA, unlike colorectal and most gastric carcinomas, so the use of DD9-E7, together with a CEA antibody, could be of value in the differential diagnosis of metastases from these sites.

**The use of monoclonal antibodies to T cells and leucocyte common antigen in the identification of large cell lymphomas in formalin fixed, paraffin embedded material**

D.B. Jones[1], P. Beverley[2] & D.H. Wright[1]

[1]*University Department of Pathology, General Hospital, Southampton SO9 4XY and* [2]*ICRF Tumour Immunology Unit, University College Hospital, London, UK.*

Monoclonal antibodies to leucocyte subpopulations have enhanced our ability to characterise lymphoma. However, most monoclonal antibodies react only with cells in frozen section and are, therefore, only applicable where adequate fresh biopsies can be obtained. Further, whilst cell surface antigens are frequently expressed strongly on reactive populations, large cell lymphomas often express antigen weakly and cell lineage determination can be difficult even with enhanced staining. In this presentation we report the results of a study of 40 cases of fixed large cell lymphoma using the monoclonal antibodies UCHL1 and PD7. The former is raised to a T cell clone and the latter identifies leucocyte common antigen. Enhanced staining, using biotin-streptavidin large complex was employed throughout. The bulk of the cases had a diagnosis of T cell lymphoma on morphological grounds and, in some cases, frozen section staining confirmed this diagnosis. Weak staining with PD7 was frequently seen in cases where the UCHL1 staining was strong, suggesting that leucocyte common antigen expression on large cell lymphoma may be weak. This observation has diagnostic implications. In 10 cases of Hodgkin's disease, UCHL1 did not stain the tumour cell population. In conclusion, our data suggest that the antibody UCHL1 is a useful adjunct to leucocyte common antigen in the diagnosis of large cell lymphoma in paraffin section.

**Differential expression of MHC D-region sub-locus products on human colorectal cancers: An immunohistological study**

A.K. Ghosh & M. Moore

*Paterson Laboratories, Christie Hospital and Holt Radium Institute, Manchester M20 9BX, UK.*

The MHC status of tissue from 28 primary gastrointestinal (GI) neoplasms (colorectal 26; stomach 2), villous adenomata (VA 2) and inflammatory bowel disease (IBD 3) was evaluated using a panel of monoclonal antibodies (McAbs) by cryostat immunocytochemistry. With 2 exceptions, all the carcinomas (cas) and the VA were uniformly positive with anti-Class I (monomorphic determinant) McAbs, although staining of 2 further cases was weak. A more complex pattern of reactivity encountered using a panel of Class II McAbs, directed against the DP, DQ and DR monomorphic determinants. Normal GI glandular and luminal epithelium was consistently Class II negative but 19 out of 28 (68%) neoplasms were positive, the proportions of stained epithelial cells ranging from 5 to 90%. Expression of Class II products tended to be non-coordinate: DR was the predominant specificity ($19/19^+$) followed by DP ($13/19^+$) and DQ ($5/19^+$). The epithelia of 3 samples of IBD were positive for all 3 D-region products, as was one VA. Further analysis with a panel of anti-leucocyte McAbs revealed a numerical superiority of stromal T cells over those in epithelium. In the stroma T cells of helper-inducer phenotype (Th+i) pre-dominated over those of cytotoxic-suppressor phenotype (Tc+s) (ratio=2.2 for Class II +ve tumours; 3.1 for Class II −ve). In tumour epithelium the corresponding ratios were 0.8 and 0.7. Few intratumour T cells expressed the IL-2 (Tac) receptor. There was thus no correlation between MHC status and the extent or phenotype of infiltrating T cells and a similar lack of correlation was observed for macrophages. Possible mechanisms for the induction of Class II molecules on inflamed and neoplastic epithelia will be discussed and an analysis of the relationship between MHC status, mononuclear cell infiltration, pathological parameters and clinical course will be presented.

### Expression of MHC products and tumour-associated antigens (TAA) before and after treatment of malignant melanoma with IFN-γ

T. Cerny[1], A.K. Ghosh[3], A. Street[3], J. Wagstaff[1], N. Thatcher[1], M. Harris[2] & M. Moore[3]

*Departments of [1]Medical Oncology, [2]Pathology and [3]Paterson Laboratories, Christie Hospital and Holt Radium Institute, Manchester M20 9BX, UK.*

IFN-γ is an effective inducer of MHC Class II expression in cell lines of malignant melanoma. To investigate the possibility that IFN-γ may increase MHC Class I and II and melanoma TAA *in vivo* we conducted immunohistochemical analyses of biopsies from 6 patients with metastatic disease. (Clinical Stage II and III.) Biopsies of skin or soft tissue metastases were taken before and after a variable duration of IFN-γ treatment (50 h up to 8 weeks; only 1 follow-up biopsy in one patient). Subcutaneous injections of IFN-γ (rDNA IFN-γ Schering 36850) were given 3 times a week and single doses were in the range $3\,mg\,m^{-2}$ to $5\,mg\,m^{-2}$ ($6–10 \times 10^6$ U IFN-γ). The maximum tolerated dose was $5\,mg\,m^{-2}$. For immunostaining, 3 anti-melanoma (Sorin Biomedica Clones 225.28S, 763.24T and 376.96S) and several anti-MHC monoclonals were used. Before IFN-γ treatment, all melanomas were Class I positive and all were Class II negative. After treatment Class I expression was neither enhanced nor Class II expression induced in any tissue sample regardless of biopsy time or dose of IFN-γ. TAA expression was similarly unchanged. However, in one case from which a primary culture was established, IFN-γ ($500\,U\,ml^{-1}$) clearly increased MHC Class II expression. The possibility that *in vivo* IFN-γ induces quantitative changes in the expression of MHC products and TAA cannot be excluded on the basis of the qualitative immunocytochemical technique. However the ease with which Class II products are routinely detected on cultured melanoma cells and epithelial cancers suggests, among other factors, that there is no detectable induction of these antigens *in vivo*. So far, none of the patients has responded to IFN-γ treatment.

### Specificity of monoclonal antibodies against gamma glutamyl transpeptidase

M.M. Manson & J.A. Green

*MRC Toxicology Unit, Woodmansterne Road, Carshalton, Surrey SM5 4EF, UK.*

Gamma glutamyl transpeptidase (GGT), an enzyme present in many normal epithelial cells, is also induced in some preneoplastic and neoplastic lesions during chemical carcinogenesis. We have raised monoclonal antibodies against GGT from rat Kidney. BALB/c mice were immunised with papain cleaved, affinity purified enzyme (Cook & Peters, *Bioclin., Biophys. Acta.*, **828**, 205, 1985) and hybridoma cultures screened by solid phase ELISA with streptavidin peroxidase complex for detection. Five monoclonals were identified which immunoprecipitated GGT from solubilised rat kidney brush border membranes, but not from plasma membranes of aflatoxin $B_1$-induced hepatoma as shown by histochemical staining of protein blots from non-denaturing gels. Immunohistochemistry with alkaline phosphatase conjugated second antibody showed that 2 monoclonals recognised GGT

in acetone fixed sections of adult rat kidney and 2 more reacted weakly if sections were pretreated with 0.1% protease to unmask antigenic sites. The antibodies do not recognise GGT in foetal or neonatal kidney and react only with the enzyme in 2 yr old kidney. A number of dimethylnitrosamine induced kidney tumours were examined (material provided by Dr. H.E. Driver). Neither epithelial tumours (weakly positive for GGT activity) nor mesenchymal tumours were immunoreactive but many GGT+ve ducts trapped within the tumours were immunoreactive. Of 3 monoclonals more fully characterised, none cross-reacted with other rat tissues including hepatoma, nor with mouse, guinea pig or marmoset kidney. The specificity of these monoclonals for normal adult rat kidney isozymes of GGT suggests the possibility of raising others against isozymes in preneoplastic and neoplastic lesions or to those found in human serum during various disease states, which would greatly enhance the diagnostic value of GGT.

### Interferon-γ production by clones derived from human peripheral blood T cells and NK cells

S.E. Christmas[1], A. Meager[2] & M. Moore[1]

[1]*Paterson Laboratories, Christie Hospital and Holt Radium Institute, Manchester M20 9BX and*
[2]*National Institute for Biological Standards and Control, London NW3 6RB, UK.*

A variety of different cell types including natural killer (NK) cells has been reported to be capable of producing interferon-γ (IFN-γ). It has recently been appreciated that NK cells, as well as having cytolytic activity towards certain tumour lines, may have a wider immunoregulatory role and that this may be their primary function *in vivo*. The production of IFNs represents an aspect that is readily amenable to investigation *in vitro*. We have developed a technique of cloning human NK cells obtained from the peripheral blood of normal donors (*Eur. J. Immunol.*, **15**, 448, 1985). Lymphocytes treated with the B73.1 (Leu 11) monoclonal antibody, which recognises all peripheral NK cells, are sorted into B73.1$^+$ and B73.1$^-$ (T cell) fractions using a FACS IV. Cells are then cloned by limiting dilution in a feeder system incorporating allogenic mononuclear cells, a B lymphoblastoid cell line, interleukin 2 (IL-2) and PHA. Cloned cells are then incubated at $10^6$ cells ml$^{-1}$ in 2.5 μg ml$^{-1}$ PHA for 48 h and the supernatants assayed for IFNs using antiviral and immunoradiometric assays. While freshly isolated B73.1$^+$ and B73.1$^-$ cells both produced significant amounts of IFN-γ, most B73.1$^+$ derived clones produced low amounts of IFN-γ under these conditions. Those which secreted higher amounts of IFN-γ had acquired the pan-T cell marker OKT-3 during culture. Both OKT-4$^+$ and OKT-8$^+$ T cell clones produced IFN-γ. The results suggest that most NK cells capable of proliferating in IL-2 are either unable to or lose the ability to produce IFN-γ and that those which can produce IFN-γ show a T cell-like phenotype.

### Specificity of cell destruction at the tumour site during the rejection of syngeneic immunogenic rat tumours

S. Kay, R.A. Robins, E.W. Pascoe & R.W. Baldwin.

*Cancer Research Campaign Laboratories, University of Nottingham, Nottingham NG7 2RD, UK.*

The nature of the effector cell pivotal in the tumour rejection response is still under debate. A specific cytotoxic T cell is a likely candidate for killing in virally-induced tumour systems, but the T helper cell, initiating a delayed type hypersensitivity reaction recruiting non-specific effector cells, has also been demonstrated.

This study was designed to investigate the specificity of the rejection response at the tumour site. Sp4 and Mc7 are immunogenic tumours which can induce tumour-specific immune responses. These tumours were injected into naive, Mc7 immune, and Sp4 immune rats, as mixed cell suspensions, at number sufficient to allow the growth of either tumour. The growing tumour was labelled with anti-Sp4 monoclonal antibody, and analysed by FACS. The antibody labelling in the Mc7-immune animals identified the tumour as Sp4, whilst in the Sp4-immune animals, it was negative, indicating Mc7. Thus only the tumour against which the animal was immunised, was rejected.

### Depletion of T cells from human bone marrow using monoclonal antibodies and rabbit complement: A quantitative and functional analysis

A.Z.S. Rohatiner[1], R. Gelber[2], S.F. Schlossman[2], & J. Ritz[2]

[1]*ICRF Department of Medical Oncology, St. Bartholomew's Hospital, London EC1, UK and*
[2]*Dana Farber Cancer Institute & Harvard School of Public Health, Boston, USA.*

Graft *versus* host disease (GVHD) remains the principal complication of allogenic bone marrow transplantation. In animal models mature T lymphocytes have been shown to be reponsible for GVHD and *in vitro* treatment of donor bone marrow (BM) using T cell specific monoclonal antibodies and complement is being investigated as a means of preventing GVHD. Anti-T12, anti-T11 and rabbit complement were used to remove T lymphocytes from normal BM. The efficacy of depletion was investigated by immunofluorescence and by *in vitro* culture of residual cells using non-specific mitogens or allogenic B cells as the proliferative stimulus in the presence of IL-2. Immunofluorescence analysis showed complete depletion of T12+ and T11+ cells after treatment with the respective antibodies and with the combination. Nevertheless, culture of treated BM with either PHA or Con-A and conditioned medium containing IL-2 resulted in the proliferation of mature T cells (T3+, T4+ or T8+, T11+). Stimulation of treated BM with allogenic cells (Laz 388) resulted in the growth of a population with natural killer (NK) cell phenotype (T3−, T11+, NKH1+) which was found to be strongly cytotoxic against K562 cells. A clonogenic assay was used to quantitate the efficacy of target cell depletion. Three incubations with either anti-T12 or anti-T11 plus complement resulted in depletion of 1–2 logs of cells. Treatment with both antibodies concurrently resulted in elimination of 2–3 logs of target cells. It remains to be established whether such combinations will be necessary in the clinical setting.

### The effects of $MG^{++}$ or EDTA on the subcellular distribution of unoccupied oestrogen receptor in breast cancer cells

J. Nelson[1], R. Clarke[1], G.R. Dickson[2], H.W. van den Berg[3] & R.F. Murphy[1]

*Departments of [1]Biochemistry, [2]Anatomy and [3]Therapeutics and Pharmacology, Queen's University of Belfast, Northern Ireland, UK.*

The unoccupied oestrogen receptor (ER), formerly regarded as cytoplasmic now appears to be nuclear (Clark, *TIBS*, **9**, 207, 1984). EDTA, used to optimize cytoplasmic ER levels in homogenates damages cell membranes. $Mg^{++}$ preserves subcellular integrity. We have examined ER distribution and nuclear integrity in the presence or absence of divalent cations. Cells disrupted in hypotonic $Mg^{++}$ or EDTA-Tris buffering were centrifuged at 2,500 *g*. Supernatants were centrifuged at 100,000 *g* to prepare cytosol which had low ER levels. Semi-pure nuclei were prepared by pelleting at 100,000 *g* through 41% and 44% sucrose. Plasma membranes remained on top of the 41% barrier. Lactate dehydrogenase was not a contaminant of particulate fractions. With $Mg^{++}$, 5′-nucleotidase and ER were distributed between plasma membranes and semi-pure nuclei. With EDTA, most ER activity was with the nuclei. These were extensively disrupted. Plasma membranes had no ER. Mg-prepared nuclei purified by pelleting through 1.8 M sucrose were microscopically (EM) intact but had no nucleotidase or ER. Thus, nuclear ER appears to be an artifact caused by contamination with plasma membranes.

### Breast tumour activated oestrogen receptor: Distribution according to disease stage and recurrence

M.J. Hershman[1], J.O. White[2], N.A. Habib[1], M.G. Elder[2], J.G. Azzopardi[3] & C.B. Wood[1]

*Departments of [1]Surgery, [2]Gynaecology and [3]Pathology, Royal Postgraduate Medical School, London, UK.*

Human breast cancer cytosol oestrogen receptors exist in activated and non-activated states as assessed by their ability to bind to the artificial nuclear matrix (dT)-cellulose. This may distinguish receptors with high affinity for nuclear receptor sites *in vivo*. Oestrogen receptor status and activation were determined in 53 patients with benign breast disease and 131 patients with breast cancer, followed for a median of 27 months. Non-activated oestrogen receptor positive tumours occurred significantly ($P<0.05$) and more frequently in stage IV disease compared with stages I–III. No difference in tumour recurrence was found between oestrogen receptor positive (ER+ve) tumours and oestrogen receptor negative (ER−ve) tumours. However, the incidence of disease recurrence was significantly higher in non-activated (53.8%) compared with activated (17.8%) ER+ve groups ($P<0.05$). Patients with non-activated ER+ve tumours had similar recurrence rates to ER−ve patients. These data suggest that oestrogen receptor activation is a more reliable prognostic indicator than total oestrogen receptor status.

**Interferon-α increases oestrogen receptor expression in the ZR-75-1 human breast cancer cell line.**

H.W. van den Berg, W. Leahey & M. Lynch

*Department of Therapeutics and Pharmacology, The Queen's University of Belfast, Northern Ireland, UK.*

There is some evidence that interferon (IFN) may increase the expression of oestrogen receptor (ER) in target tissues, including breast carcinoma (Pouillart *et al.*, *Eur. J. Cancer Clin. Oncol.*, **18**, 929, 1982). If this were confirmed it might be expected that IFN would increase the sensitivity of breast carcinoma cells to the anti-oestrogen tamoxifen (TAM). We have previously shown that growth inhibitory concentrations of IFN and TAM are additive rather than synergistic in their effects on ZR-75-1 human breast cancer cells (*Br. J. Cancer*, **52**, 428, 1985). In this study we have examined the ability of IFN to modulate ER expression in this cell line.

Human recombinant IFN-α (10–1,000 U $ml^{-1}$) increased ER levels are measured in a whole cell binding assay and this effect was inversely proportional to dose. Specific binding at a single sub-saturating ligand concentration (1nM $^3$H-oestradiol), was increased up to 10-fold by a 2-day pre-exposure of cells to 10 U $ml^{-1}$ IFN-α. This concentration of IFN alone had no effect on cell proliferation. IFN-α induced increases in specific binding of oestradiol by ZR-75-1 cells was observed maximally when cells were treated at a low cell plating density – a factor which also increases the anti-proliferative effects of higher concentrations of the agent.

Despite marked increases in detectable ER following IFN-α treatment, preliminary experiments have failed to show enhanced sensitivity of ZR-75-1 cells to TAM following pre-treatment with IFN-α.

**Effect of vincristine (VCR) on oestrogen receptor (ER) expression and the antiproliferative effects of tamoxifen (TAM) in MCF-7 breast cancer cells**

R. Clarke[1], M. Cremin[1], J. Nelson[1], H.W. van den Berg[2] & R.F. Murphy[1]

*Departments of [1]Biochemistry and [2]Therapeutics & Pharmacology, The Queen's University of Belfast, Northern Ireland, UK.*

We have previously reported that VCR reduced ER expression in MCF-7 cells and have now examined the time course of this event. Since TAM is believed to exert its effects through interaction with ER, we have also investigated the influence of VCR on the antiproliferative effects of TAM. Following exposure to 0.5 nM or 1nM VCR, ER levels were reduced to 30% and 0% respectively but returned to or exceeded that of untreated cells within 72 h of removal of the drug. VCR (0.5 nM) reduced the rate of cell proliferation by 20%. TAM (2 μM) reduced cell proliferation by 50%. Pretreatment of cells with 0.5 nM VCR did not influence their subsequent response to TAM. VCR (1 nM) abolished ER levels and induced a similar reduction in cell proliferation as 2 μM TAM. Exposure of cells to 1 nM VCR followed by 2 μM TAM was no more cytotoxic than either drug alone. Since VCR and TAM have different mechanisms of action an additive effect might have been expected. Thus, VCR may be reducing the antiproliferative effects of TAM or *vice versa*.

**Tumour cell DNA content in carcinoma of the breast; response to endocrine therapy and effect of tamoxifen**

A.D. Baildam[1,4], J. Zaloudik[2], M. Moore[2], D.M. Barnes[4], A. Howell[3] & R.A. Sellwood[1]

*Departments of [1]Surgery, [3]Medical Oncology, [4]Clinical Research and [2]Paterson Laboratories, Christie Hospital, Manchester M20 9BX, UK.*

The relationship between DNA content of primary breast tumour cells and subsequent response to endocrine therapy was studied in 137 patients with advanced disease. All were treated with tamoxifen or ovarian ablation as first line systemic therapy, and were evaluable for response according to UICC criteria. DNA characterisation by flow cytometry was used on tumour samples from paraffin embedded fixed material according to the method of Hedley *et al.* (*J. Histochem. Cytochem.*, **31**, 1333, 1983). Grouped according to DNA indices (DI), response rates (CR+PR+SD) were respectively; DI 1.0 27/53 (51%), DI 1.2–1.7 12/27 (44%), DI 1.8–1.9 16/19 (85%), DI 2.0 18/27 (67%) and DI>2.1 3/10 (27%). The near-tetraploid group (DI 1.8–2.0) had the highest proportion of ER+ tumours (38/45, 87%, $P<0.01$).

The effect of tamoxifen upon DNA content was assessed in a separate group of 77 patients with primary breast tumours; all had two biopsies with a median interval of 8 days, and 40 of them received tamoxifen during this period. The most consistent effect of tamoxifen was an abolition or reduction in magnitude of >50% in a near tetraploid peak in those tumours with DI 1.8–2.0 in the first biopsy

(15/21 72%) compared with those not receiving tamoxifen (3/16 18%), ($P<0.01$).

These data suggest that tamoxifen preferentially affects tumours exhibiting a near tetraploid DNA content, and that this may be related to endocrine response.

### Inhibition of neutrophil oxidase activation by tamoxifen

K. Horgan, Eryl Cooke, M.B. Hallett & L.E. Hughes

*Department of Surgery, University of Wales College of Medicine, Heath Park, Cardiff CF4 4XN, UK.*

Tamoxifen has recently been demonstrated to inhibit rat brain protein kinase C (PKC) *in vitro* (O. Brian *et al., Cancer Res.*, **45**, 2462, 1985). PKC has an established role in tumour promotion, cell surface signal transduction and also activates the oxidase mechanism in neutrophils. We have utilised the neutrophil as an experimental model to assess the effect of tamoxifen on PKC activity in intact human cells.

Neutrophils from 6 healthy volunteers were separated through Ficoll-Hypaque centrifugation and stimulated by phorbol-14-myristate-13 acetate (PMA). Neutrophil oxidase activity was markedly stimulated as assessed by both oxygen consumption and oxygen radical production. These parameters were measured by a Clark electrode and luminol dependent chemiluminescence respectively. Tamoxifen inhibited the stimulation in all six samples, $IC_{50}=6.1\pm1.6\mu M$ ($\bar{x}\pm$s.e.). Measurement of intracellular ATP and application of the trypan blue exclusion test showed no significant difference before and after tamoxifen. Other PKC stimulators, mezerein and oleoyl acetyl glycerol were also inhibited by tamoxifen.

These experiments indicate tamoxifen inhibits PKC *in vivo*. This inhibition may be central to its antitumour action.

### The use of a $^{75}$Se uptake assay to measure the sensitivity of prostatic carcinoma cells to hormones

M. Ferro[1], D. Heinemann[2], P.J.B. Smith[1] & M.O. Symes[2]

*Departments of [1]Urology and [2]Surgery, Bristol Royal Infirmary, and University of Bristol, Bristol, UK.*

Specimens of tumour tissue were obtained by transurethral resection from 24 patients with prostatic carcinoma. The tumour was disaggregated using collagenase+DNAase and a relatively pure suspension of carcinoma cells were separated from the mixed cell suspension by centrifugation on a linear density gradient of Nycodenz (Nyegaard & Co., AS, Oslo), (Umpleby *et al., Br. J. Surg.*, **71**, 659, 1984). The carcinoma cells were confirmed as being of prostatic origin by staining with prostate specific antibody using the immunoperoxidase technique. Aliquots of carcinoma cells, $10^5$, were cultured for 24 h with increasing concentrations of either diethyl stilboestrol (DES) or testosterone. The cells were then washed free of hormone and resuspended in 2 ml of methionine-free MEM with $2\mu Ci\,ml^{-1}$ of $^{75}$Se (as selenomethionine) added. After a further 48 h incubation protein synthesis by the carcinoma cells was estimated as the incorporated radioactivity in the cell pellet. The percentage inhibition or increase in $^{75}$Se incorporation on exposure to hormones was estimated with reference to control aliquots of cells not exposed to hormones. DES at $1\mu g\,ml^{-1}$ inhibited protein synthesis in 15/24 cases $-29.5\pm21.3\%$ (range $-5$ to $-85\%$) and caused stimulation in 9 of 24 tumours $+22.9\pm27.2\%$ (range $+4$ to $+85\%$). However, at $10\mu g\,ml^{-1}$ DES was inhibitory in 22 of 23 patients $-65.1\pm20.9\%$ (range $-9$ to $-96\%$). Testosterone stimulated protein synthesis in 11/16 cases at $1\mu g\,ml^{-1}$, $+64.4\pm72.3\%$ (range $+14$ to $+222\%$) and in 13/18 cases $10\mu g\,ml^{-1}$ $+64.4\%$ (range $+4$ to $+256\%$).

Inhibition of protein synthesis by DES and its stimulation by testosterone may indicate tumour sensitivity to hormone therapy *in vivo*.

### Tissue plasminogen activator and urokinase in human colorectal cancer

J.S.K. Gelister[1], S. Ishaq[1], M. Mahmoud[2], P.J. Gaffney[2] & P.B. Boulos[1]

*[1]Department of Surgery, University College London, and [2]National Institute for Biological Standards and Control, London, UK.*

Plasminogen activators, tPA and uPA are implicated in tumour invasion and metastasis. UPA has been associated with colorectal cancer but not with normal colonic mucosa. However, there have been no equivalent studies of human tPA. Samples of tumour centre and edge (TC, TE) and of adjacent and distant mucosa (AM, DM) were assayed for fibrinolytic activity (FA) and for specific uPA and tPA activity by bioimmunoassay.

**Results**

| | *Carcinoma* | |
|---|---|---|
| | *TC* ($n=30$) | *TE* ($n=15$) |
| FA mm$^2$ | 263 (58–544) | 254 (81–366) |
| tPA IU ml$^{-1}$ | 0.8 (0.2–3.5) | 1.2 (0.1–2.3) |
| uPA IU ml$^{-1}$ | 0.7 (0.2–4.2) | 1.0 (0.4–1.5) |
| | *Normal mucosa* | |
| | *AM* ($n=15$) | *DM* ($n=30$) |
| FA mm$^2$ | 379 (176–422) | 395 (255–644) |
| tPA IU ml$^{-1}$ | 2.2 (1.0–4.3) | 4.1 (1.5–16) |
| uPA IU ml$^{-1}$ | 0.2 (0.2–0.9) | 0.2 (0.1–0.8) |

In carcinomas, uPA was elevated ($P<0.001$); however, tPA and fibrinolytic activity were diminished ($P<0.001$) compared to normal mucosa. There were no differences between tumour centre or edge, but adjacent had less tPA than distant mucosa ($P<0.001$) though was still higher than in carcinoma ($P<0.001$).

This study demonstrates for the first time that tPA production is diminished in colorectal cancer and confirms previous reports of elevated uPA. The capacity of colorectal cancers to release plasmin is therefore less restricted compared to normal mucosa, as the activation of plasminogen by uPA (unlike tPA) is fibrin independent, and this may be relevant to tumour invasion.

### Enhanced plasminogen activator (PA) activity in experimental colon neoplasia

J.S.K. Gelister[1], M.R. Lewin[1], F. Savage[1], J. Garcia-Frade[2], P.J. Gaffney[2] & P.B. Boulos[1]

[1]*Department of Surgery, University College, London, and* [2]*National Institute for Biological Standards and Control, London, UK.*

PAs exist in two well defined forms, tissue (tPA) and urokinase (uPA). Abberant PA production is implicated in malignant transformation and has therefore been studied in experimental colonic neoplasia. Ten Wistar rats received dimethylhydrazine (DMH, 40 mg kg$^{-1}$, s.c. weekly $\times 5$) and 10 served as controls. All 20 were sacrificed at 20 weeks, when duplicate, weighed explants of macroscopically normal left colon from all rats and 12 polyps from tumour bearing rats were established in tissue culture. After 24 h culture supernatnants were assayed for total PA, tPA and uPA activity using fibrin plates, a bioimmunoassay (using anti-human tPA) and a simple amidolytic assay respectively.

**Results**

| | | *Control Colon* ($n=10$) | *DMH Colon* ($n=10$) |
|---|---|---|---|
| Total PA | (IU ml$^{-1}$) | 0.22 (0.01–1.52) | 0.05 (0.00–1.11) |
| tPA | (IU ml$^{-1}$) | 0.00 (0.00–0.08) | 0.00 (0.00) |
| uPA | (IU ml$^{-1}$) | 0.32 (0.14–0.40) | 0.33 (0.17–0.61) |
| | | *Polyps* ($n=12$) | |
| Total PA | (IU ml$^{-1}$) | 6.69 (0.13–70.5) | |
| tPA | (IU ml$^{-1}$) | 0.28 (0.20–2.75) | |
| tPA | (IU ml$^{-1}$) | 0.86 (0.12–14.1) | |

Polyps had significantly increased PA activity ($P<0.001$), uPA activity ($P<0.005$) and tPA activity ($P<0.001$) compared to normal mucosa which did not differ in control and DMH treated rats. This study shows that experimental colonic polyps have an increased capacity to produce uPA and tPA. These enzymes have been associated with basement membrane destruction and may therefore play a role in the adenoma-carcinoma sequence.

### Carcinoma of breast contains increased, uninhibited levels of urokinase

G. Layer[1], S. Cederholm-Williams[3], S. Houlbrook[3], P. Gaffney[2], M. Mahmoud[2], M. Pattison[1] & K. Burnand[1]

[1]*Department of Surgery, St. Thomas' Hospital, London SE1,* [2]*National Institute of Biological Standards and Control, London NW3, and* [3]*Nuffield Department of Obstetrics & Gynaecology, Oxford, UK.*

Malignant tumours express different levels of tissue fibrinolytic activity compared with benign tissues and these may determine metastatic spread. We have investigated the fibrinolytic enzymes: tissue plasminogen activator (tPA) and urokinase (UK) produced by 26 breast carcinomas and 13 benign breast biopsies. Extracts of tumour, 'normal' surrounding breast and benign biopsies were prepared by cryodestruction and analysed for protein content (Bradford test) and fibrinolytic activity on fibrin plates. UK and tPA were estimated by immunoassay (ELISA) and functional bioimmunoassay (BIA) using chromogenic substrates. The enzymes were characterised by fibrin overlay zymography (incorporating antibodies) after SDS-PAG electrophoresis.

| *Breast tissues* | *Protein content mg ml$^{-1}$* | *Fibrin plates u ml$^{-1}$* |
|---|---|---|
| Benign | 0.56 | 0.14 |
| ±s.e. | 0.07 | 0.02 |
| Malignant | 0.86 | 0.32 |
| ±s.e. | 0.06 | 0.04 |
| *t* tests | $t=3.20$ $P<0.002$ | $t=3.40$ $P<0.001$ |

| | *ELISA immunoassay* | | |
|---|---|---|---|
| *Breast tissues* | *tPA iu ml$^{-1}$* | *UK iu ml$^{-1}$* | *Total iu ml$^{-1}$* |
| Benign | 2.41 | 0.20 | 2.62 |
| ±s.e. | 0.47 | 0.03 | 0.47 |
| Malignant | 3.89 | 0.81 | 4.74 |
| ±s.e. | 0.69 | 0.26 | 0.68 |
| *t* tests | $t=1.77$ $P<0.081$ | $t=2.37$ $P<0.022$ | $t=2.58$ $P<0.012$ |

| | *BIA immunoassay* | | |
|---|---|---|---|
| *Breast tissues* | *tPA iu ml$^{-1}$* | *UK iu ml$^{-1}$* | *Total iu ml$^{-1}$* |
| Benign | 3.89 | 0.36 | 4.36 |
| ±s.e. | 0.78 | 0.16 | 0.86 |
| Malignant | 5.64 | 1.60 | 7.23 |
| ±s.e. | 0.93 | 0.37 | 0.94 |
| *t* tests | $t=1.44$ $P=0.156$ | $t=3.10$ $P<0.003$ | $t=2.26$ $P<0.027$ |

| | *Zymography* | |
|---|---|---|
| *Breast tissues* | *tPA % total* | *UK % total* |
| Benign ± s.e. | 71 | 3 |
| Malignant ± s.e. | 87 | 27 |
| *t* tests | $\chi^2=3.46$ $P>0.05$ | $\chi^2=6.58$ $P<0.02$ |

Tumour extracts had significantly elevated protein and fibrinolytic activity. UK and tPA measured by ELISA and BIA correlated ($r=0.794$, $n=89$, $P\ll0.001$) suggesting absence of inhibition. No inhibitors were shown by zymography and UK was almost exclusively confined to malignant extracts.

These results suggest invasive breast carcinoma is associated with a significantly increased and uninhibited production of tissue urokinase.

### A specific binding site for two-chain human urokinase on human breast cancer cell membranes

G.K. Needham[1], A.L. Harris[2], G.V. Sherbet[2] & J.R. Farndon[1]

*Department of [1]Surgery and [2]Cancer Research Unit, University of Newcastle upon Tyne, Newcastle upon Tyne, UK.*

Plasminogen Activators (PA) are associated with tissue remodelling and cell migration and occur in larger amounts in malignant tissues than in their normal counterparts. PAs are closely associated with the cell membrane fraction. We have shown EGF receptors on breast cancers are associated with a poor prognostic subgroup and it is known that EGF can stimulate PA secretion. The inactive 'A' chain of urokinase has amino acid sequence homology with EGF.

A study has been made of binding of two-chain-human-urokinase (mol. wt 54,000) to human breast cancer membrane preparations. High affinity receptors were demonstrated with a Kd of $5\times10^{-11}$ to $3\times10^{-10}$. Nine out of 20 tumours showed specific binding. Nonspecific binding ranged from 5% to 15% of totals. Assay of intrinsic membrane PA showed lower levels in tumours which bound urokinase than in those which did not.

Binding of urokinase is mediated by the inactive 'A' chain since no competition for binding sites was observed when $^{125}$I-labelled two-chain-urokinase was incubated with membrane in the presence of excess single-chain-urokinase whereas amount of ligand bound was reduced by >50% when excess two-chain-urokinase was used even when the active site was inhibited by PMSF. No interaction was seen with two-chain tissue PA or EGF.

These results show that the active site of urokinase may be presented to the tumour stroma via specific binding sites on breast cancer cell membranes,

### Alterations in host fibrinolysis affect the growth and spread of a spontaneously-metastasising murine tumour.

G.T. Layer, M. Pattison & K.G. Burnand

*Department of Surgery, St. Thomas' Hospital, London SE1 7EH, UK.*

Streptokinase has improved survival after colorectal cancer resection. We investigated altering fibrinolysis on the growth of s.c. Lewis lung carcinoma (3LL) in C57B1 mice. Fibrinolysis was estimated by euglobulin clot lysis time (ECLT). Urokinase, stanozolol and ε-aminocaproic acid (EACA) were given to groups of 10 mice; ECLT at sacrifice was altered in all groups. Changes observed were in directions opposite to those found in man.

**Results**

| *ECLT* | *EACA 90 mg day$^{-1}$* | *Urokinase 250 iu day$^{-1}$* |
|---|---|---|
| Controls mean ± s.e. min | 30.6 ± 4.5 | 30.6 ± 4.5 |
| Treatment | 17.3 ± 3.7 | 73.5 ± 8.2 |
| Mann–Whitney U test | $P<0.032$ | $P<0.003$ |
| *ECLT* | *Stanozolol 0.125 mg wk$^{-1}$* | *Stanozolol 0.5 mg wk$^{-1}$* |
| Controls mean ± s.e. min | 18.2 ± 0.8 | 18.2 ± 0.8 |
| Treatment | 28.9.0 ± 3.4 | 37.0 ± 2.5 |
| Mann–Whitney U test | $P<0.039$ | $P<0.004$ |
| *ECLT* | *Stanozolol 2.5 mg wk$^{-1}$* | |
| Controls mean ± s.e. min | 30.6 ± 4.5 | |
| Treatment | 205.0 ± 33.8 | |
| Mann–Whitney U test | $P<0.004$ | |

**Results**

| *Primary tumour weight* | *EACA 90 mg day$^{-1}$* | *Urokinase 250 iu day$^{-1}$* |
|---|---|---|
| Controls mean ± s.e. mg | 1152 ± 138 | 1152 ± 138 |
| Treatment | 1179 ± 207 | 2527 ± 332 |
| Mann–Whitney U test | $P<0.734$ | $P<0.005$ |
| *Primary tumour weight* | *Stanozolol 0.125 mg wk$^{-1}$* | *Stanozolol 0.5 mg wk$^{-1}$* |
| Controls mean ± s.e. mg | 1152 ± 138 | 2986 ± 661 |
| Treatment | 2572 ± 332 | 5037 ± 806 |
| Mann–Whitney U test | $P<0.001$ | $P<0.027$ |
| *Number of metastases* | *EACA 90 mg day$^{-1}$* | *Urokinase 250 iu day$^{-1}$* |
| Controls mean ± s.e. | 14.5 ± 3.7 | 14.5 ± 3.7 |
| Treatment | 20.3 ± 4.0 | 27.9 ± 6.3 |
| Mann–Whitney U test | $P<0.154$ | $P<0.043$ |
| *Number of metastases* | *Stanozolol 0.125 mg wk$^{-1}$* | *Stanozolol 0.5 mg wk$^{-1}$* |
| Controls mean ± s.e. | 14.5 ± 3.7 | 12.9 ± 4.8 |
| Treatment | 27.0 ± 9.1 | 28.4 ± 5.1 |
| Mann–Whitney U test | $P<0.164$ | $P<0.020$ |

Further groups of 10 mice similarly treated were implanted with 3LL and killed 3 weeks later when the tumour was measured and metastases counted.

Mice receiving urokinase and stanozolol prolonging ECLT developed larger tumours and more metastases. Host fibrinolysis thus affects malignant growth and its manipulation may be of benefit in limiting tumour spread.

### Anti-tumour activity and pharmacokinetics of LM985 in mouse colon tumours

J.A. Double, M.C. Bibby & P.M. Loadman

*Clinical Oncology Unit, University of Bradford, Bradford BD7 1DP, UK.*

NSC 293015 (LM985) is a chromone derivative selected for clinical trials primarily for its activity against colon tumour 38 as part of the NCI screen. We have investigated its anti-tumour activity against three differing transplantable adenocarcinomas of the mouse colon (MAC). Single i.p. injection at maximum tolerated dose showed no activity against MAC 15A, moderate activity against MAC 13 and produced a significant growth delay against MAC 26. These responses against MAC 13 and MAC 26 were considerably enhanced by repeated injection 7 days later when greater than 90% tumour inhibition was seen in MAC 13 and cures were achieved in MAC 26. Pharmacokinetic studies using the method of Kerr *et al.* (*Br. J. Cancer*, **52**, 467, 1985) confirm the rapid degradation of LM985 to LM987, the possible active principle ($t_{\frac{1}{2}}=96.5$ min in plasma at 37°C *in vitro*). Analysis of plasma following different *in vivo* dose levels of LM985 indicated a good dose response relationship between levels of LM975 and the administered dose. Analysis of area under the curve for LM975 indicated a good relationship with tumour responses (100 mg kg$^{-1}$, AUC = 0.95 ± 0.26 (s.e.) mg h$^{-1}$ ml$^{-1}$; 200 mg kg$^{-1}$, AUC = 2.10 ± 0.31 mg h$^{-1}$ ml$^{-1}$; 400 mg kg$^{-1}$, AUC = 3.89 ± 0.54 mg h$^{-1}$ ml$^{-1}$). The MAC system shows a good correlation with human large bowel cancer with responses only seen close to maximum tolerated dose. These preliminary observations with LM985 would suggest that it or its metabolite LM975 may have a value in the management of large bowel cancer but its ultimate clinical potential will also depend on any acute and chronic toxicity which need to be determined.

### Successful culture of gastric tumours by clonogenic assay: Initial drug results

A.P. Simmonds[1], S.A. Smith[1] & C.S. McArdle[2]

[1]*Cell Laboratory, Biochemistry Department and* [2]*Department of Surgery, Royal Infirmary, Glasgow, UK.*

We have cultured gastric tumours by clonogenic assay and measured sensitivity of such specimens to drugs in current clinical use. Tumour specimens obtained were disaggregated mechanically and $2 \times 10^5$ viable cells exposed to adriamycin, *cis*-platinum, methotrexate and 5-fluorouracil at 10% peak plasma concentrations for 1 h. Drugs were washed off and tumour cells plated in McCoy's 5A+10% FBS in 0.3% agar over an underlayer of the same medium in 0.5% agar+1% rat rbc. Incubation was at 37°C in 5% $CO_2$/air, high humidity, for 12 days. Colonies stained with INT violet were counted and drug effects expressed as percentage survival of control, <50% being sensitive. Of 9 patient samples tested, all grew with PE from 0.015–0.05 (3/9 >0.03). Only 2/8 (25%) were S to adriamycin, platinum and MTX and 1/8 (13%) S to 5FU. Resistance to 5FU was prnounced (4/7 >70% survival) as was that to platinum (S specimens <30% survival). Resistance to MTX and adriamycin was also overt. Of 5 patients tested for 4 drugs, one was R to 4/4, 3 were R to 3/4 and 1 R to 2/4. Of 4 patients tested with 3 drugs, 2 were R to all and 2 were R to 1/3. One patient received chemotherapy which was discontinued because of toxicitiy. He was R to 3/4 drugs but S to platinum. Since biopsy specimens came from patients with 'curative' resections, unlikely to receive drugs, the value of this system lies in culture success and it will now be used to investigate both new drugs and modulators of cytotoxicity for management of gastric cancer.

### Responses of transplantable adenocarcinomata of the mouse colon (MAC) to N-[N′-(2-chloroethyl)-N-nitroso-carbamoyl](CNC)-alanine and derivatives

M.C. Bibby & J.A. Double

*Clinical Oncology Unit, University of Bradford, Bradford BD7 1DP, UK.*

2-chloroethyl-N-nitrosoureas are extremely effective anticancer agents in experimental tumour systems. Their clinical value is limited by their pronounced and delayed bone marrow toxicity and the development of new analogues with equally good anti-tumour activity but with reduced toxicity would provide a major contribution to cancer chemotherapy. Ehresmann *et al.* (*Arch. Pharm.* (*Weinheim*), **317,** 481, 1984) have synthesised a series of nitrosoureas in which the nitrosocarbamoyl residue was linked to an amino acid or its derivatives. The amide derivatives were subsequently shown to be highly active against the L5222 rat leukaemia (Zeller *et al., J. Cancer Res. Clin. Oncol.,* **108,** 249, 1984). The mouse adenocarcinoma of the colon (MAC) series of transplantable tumours has been shown to have a similar spectrum of chemosensitivity to human large bowel cancer (Double & Ball, *Cancer Chemother. Rep.,* **59,** 1083, 1975). In this study the antineoplastic activity of CNC-alanine, CNC-alanyl-alanine, CNC-alanine-methylamide and CNC-glycine-methylamide was examined in a solid line (MAC 13) and in an ascitic line (MAC 15A). The compounds showed varying degrees of activity with the methylamide derivatives being highly active against both lines. CNC-alanyl-alanine was the most active agent in this series against the ascitic tumour, with a T/C of greater than 400%. The activity of these compounds against MAC 15A is particularly impressive as the previous best recorded responses with this tumour line seen with methylCCNU and mitozolomide are only in the region of 170%. Studies on the delayed and cumulative toxicity of these compounds particularly to the bone marrow are required to see whether they are likely to have any therapeutic advantage over the nitrosoureas in current clinical use.

### Drug resistance in carcinogen treated hepatocytes

H. James & M.J. Embleton

*Cancer Research Campaign Laboratories, University of Nottingham, University Park, Nottingham NG7 2RD, UK.*

The administration of three cycles of 0,06% 2-acetylaminofluorene (AAF) diet to male Fischer rats induces the formation of enzyme-altered foci and hyperplastic nodules antecedent to hepatocarcinoma. One of the reported characteristics of these altered cell populations is a relative resistance to certain chemical agents and carcinogens.

The effects of diethylnitrosamine (DENA), AAF and cyclophosphamide on hepatocytes from control or AAF-treated rats were determined using a short term cytotoxicity assay. Hepatocytes were obtained from rats by a recirculating enzyme perfusion technique and were transferred to a primary monolayer cell culture system.

Following an attachment period, the cell cultures were exposed to the effects of the chemical agents at a range of concentrations for a total of 48 h, being pulsed with $^{75}$Se-selenomethionine after 24 h. The viability of the cells was assessed quantitatively by their incorporation of the isotope. The toxic effect of each drug was expressed as a percentage of the survival in untreated cultures.

Preliminary results have shown that hepatocytes from carcinogen-treated rats were less sensitive than control hepatocytes to the toxic effects of AAF and DENA, but were equally sensitive to those of cyclophosphamide. This is observed at a time when changes in enzyme activity and histological appearance are evident in sections of altered-liver from carcinogen-treated rats.

### Comparison of dietary carbohydrate and fat on the growth and cachectic effect of a transplantable adenocarcinoma

R.A. Brennan & M.J. Tisdale

*CRC Experimental Chemotherapy Group, Institute of Pharmaceutical Sciences, Aston University, Birmingham B4 7ET, UK.*

The MAC 16 is a chemically induced transplantable colon adenocarcinoma which produces extensive weight loss in tumour-bearing animals. Weight loss is directly related to the size of the tumour and occurs without a reduction in food intake. *In vitro* experiments show a high rate of glucose consumption by MAC 16 cells and a low rate of palmitate oxidation. *In vivo* the tumour is poorly vascularised and might be expected to have a low oxygen tension. The only metabolic substrate available for use under such conditions is glucose, since the Embden Meyerhoff pathway is the only means of ATP production that does not require oxygen. In order to investigate the effect of diet composition on tumour growth rate and weight loss mice fed on diets with increasing proportions of energy from fat supplied as medium chain triglycerides (MCT) were compared with mice fed additional calories as sucrose. Mice consuming diets in which up to 80% of their energy requirements were met by MCT showed significantly lower weight-loss and decreased tumour size when compared with controls fed normal laboratory pellets. However, tumour-bearing mice fed a high carbohydrate diet had significantly larger tumours and greater weight loss than controls. These results suggest that it might be possible to control tumour growth and weight loss by feeding an appropriate diet.

### Cancer control in GDR

A. Glaser

*Chirurgische Klinik der Martin-Luther Universitat, DDR 4020 Halle/Saale, Ernst-Grube-Str. 40 GDR*

In 1952 the National Cancer Registry was founded. Though every case of cancer has to be announced to the registry with a complete follow-up of every patient for 5 years, the incidence of cancer increased heavily in the last 30 years depending on improved registration by the time; e.g. cancer of the rectum increased from 13.6 per 100,000 inhabitants in 1961 to 20.3 in 1980. Six thousand, eight hundred and twenty-eight cases of cancer of the colon and rectum were counted in 1980 thus occupying 3rd place in males and 2nd in females. The 5-year survival rates differ greatly between centres with comprehensive interdisciplinary approach, hospitals without comprehensive service and hospitals treating less than 20 patients per year. Therefore a system of organisation of cancer control is to be established by founding cancer centres in every administrative area.

### Chemofluorescent location of leukaemic cells in experimental animals

F.S. Steven[1], H. Jackson[2], N.C. Jackson[2], M. Bock[2] & T.L.H. Wong[1]

*Department of [1]Biochemistry and Department of [2]Pharmacology, Stopford Building, University of Manchester, Manchester M13 9PT, UK.*

Our object has been to determine whether leukaemic cells could be located by a fluorescent marker for a cell surface protease (Steven *et al.*, *Eur. J. Biochem.*, **149**, 35, 1985). A T-cell lymphoblastic leukaemia in HO rats and the L1210 mouse leukaemia were used for this purpose. The animals were killed at intervals during the development of leukaemia. The liver, kidney, testis and epididymis were fixed in 10% formalin-saline. Frozen sections, wax-embedded sections and resin sections were prepared from each tissue. The sections were strained with 9-aminoacridine to locate cells possessing the cell surface protease. In all tissues the leukaemic cells were clearly visualised by their chemofluorescense, these results being confirmed by conventional haematoxylineosin staining. We believe that chemofluorescent staining is a valuable aid in the assessment of leukaemic status before and after drug therapy in animals. Chemofluorescent location of leukaemic cells has the advantage that individual cells can be clearly marked; these would possibly escape detection by conventional staining.

### The 6-day subrenal capsule assay – limitations of its use with primary surgical explants from gastric adenocarcinoma

D. Cunningham[1], A. Jack[2], M. Soukop[1], D. McMurdo[3], C.S. McArdle[3], D.C. Carter[3] & S.B. Kaye[4]

[1]*Department of Medical Oncology,* [2]*University Department of Pathology and* [3]*Surgery, Royal Infirmary, Glasgow and* [4]*University Department of Medical Oncology, 1 Horselethill Road, Glasgow, UK.*

The six day subrenal capsule (SRC) assay has been used as a means of predicting the response of solid tumours to chemotherapy. The method involves transplantation of fresh surgical explants $\sim 1\,mm^3$ under the renal capsule of normal immunocompetant BDF 1 mice. The perpendicular diameters of the implants are measured at the time of transplantation and at the time of sacrifice using a stereoscopic microscope with an oculometer. A control group of mice is used to confirm viability of transplanted tissue and hitherto, an increase in size of 0.05 mm of the tumour diameters was considered to indicate an evaluable assay. Inhibition of tumour growth or a reduction in the tumour size of treatment groups is used as a marker of chemosensitivity.

We have transplanted tumour obtained from 17 patients with gastric cancer involving a total (control and treatment groups) of 418 xenografts. Using the above criteria, tumour from 14 patients (82%) was viable. However, only 21 xenografts (5%) contained tumour cells. The remaining xenografts consisted of fibrous tissue and a lymphocytic infiltrate. An inflammation score was devised which showed that the control groups had significantly more infiltration ($P<0.001$) than the chemotherapy groups (treated with 5FU, epirubicin, methotrexate and *cis*-platin).

In gastric cancer, successful growth of surgical explants in the SRC has not proved feasible. A lymphocytic infiltration can produce apparent growth in otherwise non-viable tumour tissue. Stricter criteria for tumour growth in control animals including histological confirmation of tumour viability are essential.

### An *in vitro* model of oral carcinogenesis in rats induced by 4NQO

S.S. Prime[1], J. Luker[1], I. Crane[1], A. Stone[1], N.J. Maitland[2] & C. Scully[1]

[1]*University Department of Oral Medicine and Oral Surgery and* [2]*Department of Pathology, Bristol, UK.*

Animal models of oral carcinogenesis enable more controlled investigations into the pathogenesis of such lesions. This report demonstrates for the first time the successful transference of a rat model of oral carcinogenesis into cell culture. Oral carcinomas of the tongue and palate were induced in Sprague-Dawley male white rats by painting their palates 3× weekly for 7–8 months with 0.5% (w/v) 4-Nitroquinoline N-oxide (4NQO). Oral keratinocytes from malignant and untreated control tissues were cultivated using 3T3 fibroblast support (Rheinwald & Green, *Cell,* **6,** 331, 1975) and the percentage of cells expressing anchorage independence determined in gel culture (Macpherson & Montagnier, *Virology,* **23,** 291, 1964). In cell culture, malignant keratinocytes were heterogeneous with regard to cell size, shape and intercellular packing unlike the regular organisation of the normal cultures, but both the normal and malignant cells stained positively with an anti-human keratin polyclonal antibody. Malignant keratinocyte cultures differed markedly from their normal counterparts by a 5-fold increase in their growth rate, the capacity for serial cultivation to the 16th passage (to date), a loss of contact inhibition and an independence of 3T3 fibroblast support. Malignant keratinocytes expressed anchorage independence in gel culture, whereas normal cells showed no evidence of colony formation. The development of this specialised cell culture system creates further opportunities to investigate the pathogenesis of oral squamous cell carcinomas.

### Cell culture techniques to study colorectal cancer

C. Paraskeva & S. Powell

*Department of Pathology, The Medical School, University Walk, Bristol BS8 1TD, UK.*

Cancer development in the large intestine is a good example of the multistep nature of cancer. In this malignancy it is thought that many, if not most, cancers develop from premalignant adenomas in what is often called the adenoma–carcinoma sequence. To study the possible mechanism(s) and factor(s) involved in the progression of adenomas to carcinomas we have developed cell culture techniques in which colorectal adenomas from both familial polyposis coli and sporadic colorectal cancer patients can be routinely grown *in vitro*. These epithelial cells are grown in collagen coated petri-dishes in the presence of mouse 3T3 feeder cells in medium containing 20% foetal bovine serum. The epithelial nature of the cells is confirmed by positive staining with keratin monoclonal antibodies. The adenoma derived cell lines display ultrastructural features characteristic of colorectal epithelium including desmosomes, microvilli and mucin droplets. The adenomas tested so far do not produce tumours in athymic nude mice whereas carcinoma derived cell lines reproducibly produce tumours in athymic nude mice. Similarly the adenoma cultures display no detectable chromosomal abnormalities whereas carcinoma derived cell lines are invariably chromosomally abnormal. The adenoma epithelial cell lines have been in culture for various times; one of them, designated PC/AA, appears to have become immortalised since it has been in culture for ~3 years. We are currently using the adenoma derived cell lines in transformation experiments to try and understand how the adenomas progress to carcinomas.

### A trophic effect of EGF on rat colonic mucosa in organ culture

K.J. Finney[1], P. Ince[1], D.R. Appleton[2], J.P. Sunter[1] & A.J. Watson

[1]*Department of Pathology, Royal Victoria Infirmary, Newcastle upon Tyne NE1 4LP and* [2] *Department of Medical Statistics, University of Newcastle upon Tyne, Newcastle upon Tyne, UK.*

The development of an organ-culture system for rat colonic mucosa has enabled a direct assessment of the effect of epidermal growth factor (EGF) on cell division. An augmented mitotic index ($AI_m$) has been employed to identify changes in cell proliferation.

Explants of colonic mucosa from four animals were maintained in a medium containing serum for five days. On the fifth day of culture half of the explants received EGF (40ng ml$^{-1}$) and the remainder (controls) fresh medium only. At 6, 12, 24 and 48 h thereafter both experimental and control explants received vincristine (4$\mu$g ml$^{-1}$) for 3 h prior to fixation. Vincristine produces mitotic arrest and so enables the proportion of dividing cells within the explant to be determined. Analysis of the data indicated that when serum is present exogenous EGF exerts a trophic effect which increases with time ($P<0.001$).

In a second experiment colonic explants from four animals were maintained for five days in a serum-free medium and were then divided into groups each of which received one of a range of concentrations of EGF. An $AI_m$ was determined for each group after 36 h. It was found that increasing concentrations of EGF produce a small but significant rise in cell proliferation ($P<0.01$). This effect, however, was less pronounced than that seen when serum was present.

These results suggest that EGF has a trophic action on the colon and interacts with additional factors found in serum.

### Extended lifespan of fibroblasts cultured from epithelial tumours

D. Wynford-Thomas, P. Smith, & E.D. Williams

*Department of Pathology, University of Wales College of Medicine, Heath Park, Caridff CF4 4XN, UK.*

The ability to undergo an indefinite number of cell divisions is a key feature of tumour cells. We have investigated the possibility that changes in lifespan of the supporting stromal cells also may be a feature of epithelial tumour development.

We have previously shown that long-term elevation of serum thyroid-stimulating hormone (TSH) levels induced by administration of goitrogen (aminotriazole) leads initially to generalised thyroid hyperplasia followed by the development of multiple benign and eventually malignant epithelial (follicular cell) tumours. Fibroblast monolayer cultures were prepared from 8 normal, 8 hyperplastic and 3 tumour-bearing glands by multiple collagenase/dispase digestion dollowed by filtration through nylon mesh. Cells were grown in DME medium supplemented with 10% foetal calf serum and passaged 1 in 8 at confluence. Cell number was assessed by hemocytometer counts of trypsinised replicate cultures.

Cultures derived from normal glands ceased growth (senesced) after an average $6.6 \pm 1.0$ doublings (mean $\pm$s.e. of 8 replicates). Those from hyperplastic glands showed a significant ($P<0.01$) extension of lifespan to $16.6 \pm 2.1$ doublings but all have undergone senescence. All replicate cultures from 3 tumour-bearing glands have failed to show any reduction in growth rate up to the present time, and have undergone 53, 55 and 70 doublings respectively. No spontaneous foci of transformants have been observed and the cells still show normal density-dependent growth inhibition and anchorage dependence. We conclude that fibroblast-like cells from *epithelial*-tumour bearing thyroids have a greatly extended lifespan and may be truly immortalised.

**Unaffected children of patients with familial breast cancer have abnormal skin fibroblast phenotypes**

J.A. Haggie[1], S.L. Schor[1], A. Howell[1] & R.A. Sellwood[2]

[1]*Department of Medical Oncology, Christie Hospital and* [2]*Department of Surgery, Withington Hospital, Manchester, UK.*

Adult and foetal fibroblasts can be distinguished *in vitro* by their different migratory phenotypes in 3D collagen cells. We have previously shown that foetal-like migratory patterns are found commonly in the skin fibroblasts of breast cancer patients. In order to try and ascertain if this anomaly antedates the development of cancer we have studied the skin fibroblasts of unaffected children of patients with a family history of breast cancer as this group have a cumulative lifetime risk of $\sim$50% of developing breast cancer themselves. The skin fibroblasts from pre-menopausal patients with breast cancer and a family history (defined as the presence of 2 1st degree relatives from consecutive generations with breast cancer), each with a corresponding unaffected child were studied using a migration assay as previously described (Schor *et al.*, *J. Cell. Sci.*, **73**, 221, 1985).

All 7 patients and 4/7 of their unaffected children (median age 21: range 12–28) showed foetal-like migratory fibroblast phenotypes. Forty-three per cent of breast cancer patients without a family history have a foetal phenotype (Schor & Haggie, *Int. J. Cancer*, in press).

These data indicate that breast cancer patients with a family history have a greater chance of showing a foetal migratory phenotype than those without a family history. The presence of the abnormal phenotype in 57% of their children suggests that this anomaly may antedate the development of breast cancer and may also be linked with their high breast cancer susceptibility,

**Increased erythrocyte stearic acid desaturation in rats with chemically induced colorectal carcinomas**

N.A. Habib[1], R. Salem[2], M. Hershman[2], K. Apostolov[3], R.C.N. Williamson[1] & C.B. Wood[2]

[1]*Department of Surgery, Bristol Royal Infirmary, Bristol and Departments of* [2]*Surgery and* [3]*Virology, Royal Postgraduate Medical School, London, UK.*

It has been shown that malignant transformation of cells is associated with an increase in membrane fluidity, predominantly due to increase of the oleic acid content of membrane lipids relative to stearic acid. Desaturation of the lipid layer of erythrocytes has been noted in patients with malignancies. This study investigated the stearic acid desaturation in red blood cell membranes of rats during the induction of colorectal tumours. Male Sprague-Dawley rats were injected weekly with dimethylhydrazine (DMH) and sacrificed at four weekly intervals. Blood was withdrawn via heart puncture, collected in EDTA bottle and erythrocytes separated by centrifugation. Total lipid extraction was carried out and analysed with gas liquid chromatography. In the control rats (injected with normal saline) the mean of the stearic to oleic acid ratio in erythrocyte membranes was $2.0 \pm 0.3$ ($n=30$, range 1.6–2.6) compared to a mean of $0.94 \pm 0.2$ ($n=30$, range 0.5–1.57) in tumour bearing rats ($P<0.001$). The increased desaturation occurred in parallel with appearance of tumours. These data suggest the regulation of stearic acid desaturation is an important adaptive mechanism of membrane fluidity and could be a useful chemical marker for malignancy.

**Erythrocyte stearic acid desaturation in patients with colorectal carcinomas**

N.A. Habib[1], M.J. Hershman[2], P. Carter[2], K. Apostolov[3], R.C.N. Williamson[1] & C.B. Wood[2]

[1]*Department of Surgery, Bristol Royal Infirmary, Bristol and Departments of* [2]*Surgery and* [3]*Virology, Royal Postgraduate Medical School, London, UK.*

Patients with cancer often suffer systemic effects of the disease suggesting that the tumour produces a factor(s) which act on the normal cells and are responsible for the systemic changes. To assess this phenomenon we have studied the desaturation of erythrocyte cell membranes in patients with large bowel cancer. Total lipid extracts of erythrocyte cell membranes from 50 normal subjects, 20 patients with inflammatory bowel disease and 44 patients with colorectal carcinoma were analysed by gas liquid chromatography (GLC). The ratio of stearic to oleic acid was obtained from the GLC tracing and was expressed as the saturation index (SI). The mean SI in cancer patients (0.71 ± 0.22) was about half the value in normal subjects (1.54 ± 0.32; $P < 0.001$), and in those with inflammatory bowel disease (1.30 ± 0.4; $P < 0.001$). Following radical resection the SI increased above unity, only to fall again with the onset of tumour recurrence. Thus, it would appear that a desaturation producing factor is being released by the malignant cells. The erythrocyte SI is therefore of potential value in the diagnoses and postoperative monitoring of patients with carcinoma of the large bowel.

### Urinary 3-methyadenine as a marker of *in vivo* methylation of DNA

E. Bailey, P.B. Farmer & D.E.G. Shuker

*MRC Toxicology Unit, Woodmansterne Road, Carshalton, Surrey SM5 4EF, UK.*

The urinary excretion of 3-methyladenine (3-MeA), following its excision from methylated DNA, could be a marker of exposure to a methylating carcinogen.

In rats 3-MeA appears to be unmetabolised with >95% of oral and i.p. doses of 3-[$^3H_3$]MeA being excreted unchanged in the urine over the 24 h period post-dosing (*cf* Hanski & Lawley, *Chem. Biol. Interact.*, **55**, 225, 1985).

A method has been developed for the determination of unlabelled 3-MeA in urine. 3-MeA was isolated on an XAD-2 column, purified using $C_{18}$ reverse phase HPLC, and converted to the stable $N^6$-(*tert*-butyldimethylsilyl) derivative. With 3-[$^2H_3$]MeA as the internal standard, quantitation of 3-MeA was carried out by gas chromatography–mass spectrometry using characteristic ions (m/z 206[$d_0$] and m/z 209 [$d_3$]). Acceptable overall recoveries (*ca.* 50%) were obtained using 3-[$^3H_3$]MeA as a marker.

In preliminary experiments we have found low levels of 3-MeA in urine ($<10\,\mu g\,24\,h^{-1}$) of humans nominally unexposed to methylating agents.

We are currently confirming the presence of 3-MeA in human urine and determining whether levels are increased following exposure to nitrosatable drugs or other potential methylating agents.

### Relevance of endogenous nitroso compounds in cirrhotics and patients with hepatocellular carcinoma

N.A. Habib[1], C. Smadja[1], H. Ohshima[2], H. Bartsch[2] & C.B. Wood[1]

[1]*Department of Surgery, Royal Postgraduate Medical School, London and* [2]*International Agency for Research on Cancer, Lyons, France*

N-nitroso compounds (NOC) are carcinogenic and are mainly formed endogenously following nitrosation of precursors amines. The amount of urinary nitrosoproline (NPRO) excreted $24\,h^{-1}$ is an index of endogenous nitrosation and can be analysed by gas chromatography. We measured the urinary NPRO in 18 control patients, 23 cirrhotics and 16 with primary hepatocellular carcinomas (8 with and 8 without liver cirrhosis). We found that the mean of NPRO in $\mu g\,24\,h^{-1}$ were 1.2 ± 0.95 in the controls, 5.5 ± 1.56 in the cirrhotics and 6.0 ± 0.74 in the cancer patients ($P < 0.001$). There was no significant correlation between the presence or absence of cirrhosis in patients with hepatocellular carcinoma. Thus, the high urinary NPRO observed in the cirrhotic patients reflects their high exposure to endogenous carcinogens and therefore their susceptibility to form carcinomas.

### The detection of colorectal carcinomas with the use of CA-50 radioimmunoassay inhibition test

N.A. Habib[1], M.J. Hershman[2], J. Holmgren[3], L. Lindholm[3], R.C.N. Williamson[1] & C.B. Wood[2]

[1]*Department of Surgery, Bristol Royal Infirmary, Bristol,* [2]*Royal Postgraduate Medical School, London and* [3]*Department of Medical Microbiology, University of Goteborg, Sweden.*

Tumours may secrete or express on their cell surfaces ‘foetal components’ not normally present in adult cells, which may circulate and be detected in serum as ‘oncofoetal antigens’. We used a radioimmunoassay for the detection of the human carcinoma-associated antigen CA-50 in the serum of 50 normal subjects, 16 patients with inflammatory bowel disease and benign polyps and 77 patients with primary and secondary colorectal carcinomas. Serum levels in all normal patients and those with benign disease were below $17\,U\,ml^{-1}$, while 40 of 77 (51%) patients with carcinoma had levels above 17 U ml$^{-1}$. The sensitivity of this test was 22% for Duke's A carcinoma, 29% for Duke's B, 59% for Duke's C and 73% for metastatic disease. The CA-50 levels were elevated in 7 of 9 (78%) patients who developed tumour recurrence following curative surgery compared to 15 of 43 (35%) patients who are alive today and tumour free ($P < 0.05$). Therefore, this test may prove useful in the diagnosis and prognosis of patients with colorectal carcinomas.

**Cyclic nucleotides – possible tumour markers?**

A.C. Williams & P.A. Light

*Research Laboratories, Radiotherapy and Oncology Centre, Bristol Royal Infirmary, Bristol BS2 8ED, UK.*

Cyclic nucleotides are important regulators of cellular function and are implicated as factors in the determination of cellular growth and differentiation, although their actual role is still unclear.

The object of this study was to determine whether there were any detectable changes in extracellular cyclic nucleotide concentration with the presence of a tumour, and whether any such changes could be used to monitor the development of the tumour.

Cyclic adenosine 3′5′-monophosphate (cAMP) and cyclic guanosine 3′5′-monophosphate (cGMP) were measured in 24 h urine specimens from guinea pigs by radioimmunoassay. Measurements were taken before and after the guinea pigs were inoculated with L2C leukaemia. A transient increase in cGMP concentration occurred 3 days after inoculation, which preceded any detectable change in the white blood cell count. This peak was found to be highly significant when compared with fluctuations in urinary cGMP concentration in the guinea pigs before transplantation of the leukaemia, or after inoculation with irradiated L2C cells.

Urinary cyclic nucleotide concentrations were therefore measured in patients with malignant diseases, and it was found that in specific groups of patients abnormally high urinary cGMP concentrations correlated well with the status of their disease.

**Birth cohort effect on cervical cancer incidence in the West Midlands**

C.A. Meanwell[1], C. Roginski[2], K.A. Kelly[1], S. Wilson[2] & G. Blackledge[1]

[1] *West Midlands CRC Clinical Trials Unit and* [2] *West Midlands Regional Cancer Registry (WMRCR), UK.*

Recent changes in age-specific mortality rates for invasive cervical cancer in England and Wales have important implications with respect to current views on cervical cancer aetiology and management. We were therefore prompted to examine trends in cervical cancer incidence in the West Midlands. Case records of women with stage 1B-4B (International Federation of Gynaecology & Obstetrics staging) carcinoma of the cervix uteri registered with WMRCR since 1957 were examined. The percentage frequency of cases falling in the age group 20–39 years rose from 9% (43 cases) in 1970 to 31% (152 cases) by 1978 to produce a bimodal-type distribution. There was a slight decrease in registration rates and shift of the mode for older patients. Further analysis revealed a strong birth cohort effect. Women born between 1885 and 1894 or between 1920 and 1929 had high risk of developing cervical cancer in old or middle age relative to other cohorts. For women born between 1930 and 1959 there was a marked, progressive increase in all available age-specific registration rates. These observations must be interpreted against a complex background of confounding factors. Nevertheless, the data indicate that there has been a large increase in cervical cancer incidence in young women since the early 1970s and that this trend mainly reflects a birth cohort effect. The existence of other 'high risk' cohorts with increased incidence in middle or old age also suggests (1) that the current 'young' cohort may remain at relatively high risk throughout their lifetime and (2) that studies of aetiology in cervical cancer should not focus solely upon 'current events' such as use of the oral contraceptive pill.

**Cancer risk in duodenal and gastric ulcer patients after surgery**

C.P.J. Caygill, M.J. Hill, J.S. Kirkham & T.C. Northfield

*PHLS-CAMR, Salisbury, Wiltshire SP4 0JG and Gastroenterology Unit, St. James Hospital, Balham, London SW12 8HU, UK.*

A detailed analysis of 4,466 patients, using a 'years at risk' calculation in 5 year bands showed that duodenal ulcer (DU) patients, after surgery, had an initial decrease in risk of mortality from gastric cancer for the first 20 post-operative years followed thereafter by a 4-fold excess risk. Gastric ulcer (GU) patients, however, had a 3-fold excess risk throughout the first 20 post-operative years increasing to a 5.5-fold excess risk thereafter.

For cancer of the biliary tract there was a non-significant excess risk of mortality for DU patients but GU patients had a 15.8-fold excess mortality risk 20 or more years after gastric surgery. This excess risk appears to be exclusively in the gall-bladder and is not found in the bile duct. Neither site had any excess risk within the first 20 years.

For cancer of the pancreas there was a non-significant decrease in mortality within the first 20 years followed by a 4-fold increase. After 20 years both DU and GU patients have an excess risk of pancreatic cancer mortality which is greater in GU (7-fold) than DU (3-fold) patients.

One possible explanation for these site specific excess cancer risks in gastric surgery patients is the production of N-nitroso compounds in the hypochlorhydric stomach. This would explain the greater excess in GU than DU patients at all three sites, since a proportion of GU patients will already be hypochlorhydric at the time of surgery.

### Pre-operative staging of rectal cancer with transrectal ultrasound

J. Beynon[1], A. Roe[1], D.M.A. Foy[3], L.N. Temple[4], N.J.McC. Mortensen[1] & J. Virjee[2]

*Departments of [1]Surgery, [2]Radiology, [4]Pathology and [3]Medical Physics, Bristol Royal Infirmary, Bristol, UK.*

Local invasion and para-rectal lymph node involvement in rectal cancer is currently assessed by digital examination which is highly subjective and related to surgical experience. Using an ultrasound scanner type 1846 (Bruel and Kjaer, Denmark) the effectiveness of endoluminal sonography in predicting invasion and lymph node involvement is being evaluated. Rotating 5.5 and 7.0 MHz transducers provide 360° scans of the rectum and para-rectal tissues. Examinations have been performed on 44 primary rectal cancers and results compared in 39 cases with histopathological findings and in 3 cases with laparotomy observations. Local invasion was graded according to the UICC classification. Sonographically 5 cases were T1, 8 cases T2, 27 cases T3 and 4 cases T4. The coefficient of correlation between sonographic and histopathological findings was 0.93 ($P<0.001$). Invasion beyond the muscularis propria was predicted with a sensitivity of 97%, specificity of 92% and had a predictive value of 97%. Lymph node involvement was predicted in 19 cases. Correlation with histopathology revealed the technique to have a sensitivity of 93%, specificity of 74% and predictive value of 68%.

Endoluminal ultrasound staging of rectal cancer is more accurate than digital evaluation and its use pre-operatively aids the planning of subsequent treatment.

### Prognostic factors and scoring system in small cell lung cancer patients

T. Cerny[1], V. Blair[2], H. Anderson[1] & N. Thatcher[1]

*[1]CRC Department of Medical Oncology and [2]Department of Statistics, Christie Hospital, Manchester, UK.*

In 407 patients with small cell lung cancer (SCLC) 60 variables were evaluated in a Cox Multiregression Analysis to assess their prognostic value. All patients received a short term intensive regimen (cyclophosphamide, etoposide and methotrexate or ifosfamide and etoposide, both followed by thoracic irradiation, if complete response was noted).

LDH ($P=0.0001$), stage ($P=0.001$), sodium ($P=0.0321$), pre-treatment Karnofsky performance score (KP, $P=0.0009$), alkaline phosphatase ($P=0,0186$) and CO2 ($P=0.0321$) were the only prognostic significant factors. After allowing for these 6 variables, the treatment modality was not a significant factor. A simple scoring system using these variables was established (Table), and shows little loss of information compared to the Cox analysis results. The group with a score of <2 included most of the long term survivors (15% over 2 years survival) whereas in the group with a score of 2 and 3, only 4% and with a score of ⩾4, no long term survivors were found. This scoring system may help to design new treatment strategies, and may also facilitate the comparison of different studies.

| *Score for SCLC* |
| --- |
| +1: if LDH $>450\,iu\,l^{-1}$ |
| +1: if extensive disease |
| +1: if sodium $<132\,mmol\,l^{-1}$ |
| +1: if KP<60 |
| +1: if alk.phos. $>110\,iu\,l^{-1}$ |
| +1: if CO2 $<24\,mmol\,l^{-1}$ |

**A computerised system for reporting phase II studies**

C. Hilton, N. Stuart, K.A. Kelly, J. Kavanagh, F. Lawton & G. Blackledge

*West Midlands CRC Clinical Trials Unit (WMCRCCTU), UK,*

Phase II clinical trials play an essential role in the introduction of new anti-cancer agents. After formulation of the new treatment and initial toxicity testing in a Phase I study, Phase II aims to assess response in specific diseases and to document toxicity. Many new anti-tumour agents are being developed generating a large number of Phase II studies, with some 30 to 40 being carried out at any one time in the WMCRCCTU alone. The requirements of these studies are similar and a standardised approach would improve the quality of the data collected and analysis. A unified system including general data collection forms and computerised data input and reporting facilities has been developed. Statistical analysis and graphical representation of the data uses programs from the statistical package BMDP. The main features of the system developed by the WMCRCCTU will be described by reference to a Phase II study of *cis*-platinum, adriamycin and bleomycin followed by escalating cyclophmsphamide consolidation in advanced epithelial ovarian cancer.

**Clinical investigations with trimelamol ($N^2$,$N^4$,$N^6$-trihydroxymethyl-$N^2$,$N^4$,$N^6$-trimethylmelamine): Phase I study with 2 dose schedules and evaluation in ovarian cancer**

I.R. Judson, C.J. Rutty, L. Gumbrell, G. Abel, K.R. Harrap & A.H. Calvert

*Drug Development Section, Inst. Cancer Res., Sutton, Surrey, UK.*

Trimelamol is an analogue of HMM and PMM undergoing clinical trial at the Royal Marsden Hospital. It is less neurotoxic than PMM in rodents due to reduced CNS penetration and has proved less toxic and probably more active in man. Unlike PMM it does not require metabolic activation and produces much higher plasma levels of cytotoxic N-hydroxymethylmelamines in patients. Sixty-four patients, 43 with ovarian cancer, have been treated either by single i.v. infusion every 3 weeks at 25–2,400 mg m$^{-2}$ or with 3 daily doses of 400–900 mg m$^{-2}$ every 3 weeks. Pharmacokinetic analysis in 4 patients treated at 1,500 mg m$^{-2}$ gave a mean plasma AUC 3,164 $\mu$M.min which compares favourably with the observed AUC of 2,834 $\mu$M.min at 90 mg kg$^{-1}$ i.v. in the mouse, the $ED_{90}$ *versus* the PC6 tumour. Myelosuppression, mainly leukopenia, was dose-limiting for the single dose schedule, median WBC nadirs at 1,800, 2,100 and 2,400 mg m$^{-2}$ were 4.0, 2.6 and $1.1 \times 10^9 l^{-1}$. Thrombocytopenia was rare but some patients required transfusion for drug-related anaemia. Doses >1,500 mg m$^{-2}$ caused WHO grade III nausea and vomiting but no acute sedation. The 3-day schedule was better tolerated and probably less myelosuppressive, WBC nadirs at 700, 800 and 900 mg m$^{-2}$ ×3 were 4.8, 3.0 and $2.8 \times 10^9 l^{-1}$. Responses have been observed in Hodgkin's disease, colon and ovarian cancer at doses >1,500 mg m$^{-2}$. The ovary patients were *cis*- or carboplatin resistant or in 2nd/3rd relapse. Responses were – 'single dose' (18 pts): CR 1; PR 3; MR 5 (22%) and '3-day' (8 pts): CR 0; PR 2; MR 1 (25%). This promising activity requires confirmation in a larger group of patients.

**A Phase I study of Methylene Dimethane Sulphonate**

D.B. Smith[1], B. Fox[2], N. Thatcher[1], J.H. Scarffe[1], J. Wagstaff[1], W.P. Steward[1] & D. Crowther[1]

[1]*CRC Departments of Medical Oncology and* [2]*Experimental Chemotherapy, Christie Hospital and Paterson Laboratories, Manchester, UK.*

Methylene Dimethane Sulphonate (MDMS) is the first (Cn = 1) member of a homologous series of bi-functional alkylating agents and MDMS is of interest because its small molecular size allows access to alkylating sites not available to larger molecules. The starting dose for this study was 14 mg m$^{-2}$ (1/10th mouse LD10) and escalation used a modified Fibonacci search scheme. Twenty-eight patients with advanced solid tumours have been studied at 8 dose levels. No significant toxicity was seen at doses below 100 mg m$^{-2}$. The haematological toxicity at higher doses is summarised below:

| *Dose* mg m$^{-2}$ | *Patients/ Courses* | *WBC* × $10^9 l^{-1}$ *median (range)* | *Platelets* × $19^9 l^{-1}$ median (*range*) |
|---|---|---|---|
| 125 | 7/8 | 1.8 (0.8–3.7) | 64 (21–176) |
| 170 | 6/10 | 2.5 (1.2–5.4) | 46 (11–221) |
| 225 | 1/1 | 0.6 | 11 |

In previously untreated patients wbc and platelet nadirs occurred at day 21 with recovery by day 28

whereas in previously treated patients nadirs occurred at day 14 with recovery delayed until day 30–42. GI toxicity was only seen above 125 mg m$^{-2}$ and was mild, affecting only 25% of patients. Total alopecia occurred at doses above 125 mg m$^{-2}$ with varying degrees of loss at lower doses. There was no renal, hepatic or local toxicity. There has been one PR in a patient with locally advanced pulmonary carcinoid who received 225 mg m$^{-2}$. The MTD for MDMS as a single bolus injection is 225 mg m$^{-2}$, the dose limiting toxicity being thrombocytopenia. The recommended doses for phase II studies are 125 mg m$^{-2}$ q35–42 days in poor risk patients and 170 mg m$^{-2}$ q28 days in good risk patients.

### Blood transfusion blood group and blood group antigen expression in colorectal cancer

N.C. Armitage, I.O. Ellis & J.D. Hardcastle

*Departments of Surgery and Histopathology, University of Nottingham, Nottingham, UK.*

It has been suggested that blood transfusion may bear an adverse effect on survival in patients with colorectal cancer and that expression by the tumour of blood group antigens may also be related to survival. One hundred and ninety-nine patients with colorectal cancer, with at least 5 years follow-up, were studied. Patients' blood group and transfusions given were noted. In 104 cases the tumours were stained for the presence of blood group antigen H (Blood group O substance) by immunohistology using a monoclonal antibody and assessed by two observers. There was no survival advantage to any particular blood group. Fifty-two of 127 (40%) patients who were transfused survived 5 years, compared with 33/72 (46%) who were not transfused ($\chi^2=0.27$, $P$=NS). Expression of blood group antigen H by the tumour was associated with a significantly improved survival.

| | *5 year survival* | |
|---|---|---|
| | *H Positive* | *H Negative* |
| Blood Group O individuals | 12/27 (44%) | 1/18 (5%)* |
| Others | 11/30 (37%) | 7/29 (24%)† |
| Overall | 23/57 (40%) | 8/47 (17%)‡ |

*$\chi^2=6.17$ $P<0.02$ †$\chi^2=0.55$ $P$=NS ‡$\chi^2=5.6$ $P<0.025$

Neither blood group nor blood transfusion affected subsequent survival but expression by the tumour of blood group antigen H, in blood group O individuals, conveyed a significant survival advantage.

### The effect of peri-operative blood transfusion on colorectal cancer recurrence

N.R. Parrott, T.W.J. Lennard, B.K. Shenton, G. Proud, R.M.R. Taylor & I.D.A. Johnston

*Department of Surgery, Medical School, University of Newcastle upon Tyne, Newcastle upon Tyne NE2 4HH, UK.*

Nine hundred and one cases of colorectal cancer treated between 1974 and 1985 have been reviewed noting those who received peri-operative blood transfusion, and those who did not. Information was retrieved from case notes, microfilms, and transfusion records. The recurrence rates of tumours in each of the two groups have been analysed. Three hundred and eighty-four patients were excluded: 136 with metastases at operation; 106 had palliative surgery; 76 peri-operative deaths and 66 with incomplete data. This left 517 patients for analysis. Of these 373 (72%) received transfusions and 144 (28%) did not. The two groups were comparable in terms of age*, sex distribution†, Duke's staging†, histological differentiation†, site of colonic lesion† and duration of follow up*. The transfused group contained significantly more rectal lesions† ($P<0.001$), had longer operations*, (2.35 *vs.* 1.90 h) ($P<0.001$), and a greater operative blood loss* (837 *vs.* 333 ml) ($P<0.001$). In the transfused group there were 169 cases of tumour recurrence (45.3%) compared with 41 in the non-transfused group (28.4%). This difference was highly significantly to the detriment of the transfused patients† ($P\ll0.001$). A similar trend was seen in cancer related deaths; transfused/non-transfused = 130/30 respectively ($P<0.01$)†. The results of this study strongly suggest that peri-operative blood transfusion may be significantly detrimental to patients undergoing curative surgery for colorectal cancer.

*Analysed by unpaired T test. †Analysed by Chi-squared.

### Role of functional oestrogen receptors as biochemical marker in breast cancer

U. Verma, K. Murugesan, K.R. Laumas, B.M.L. Kapur & A. Farooq

*Department of Reproductive Biology and Surgery, All India Institute of Medical Sciences, New Delhi 110029, India.*

The role of oestrogen receptor (ER) assays in determining therapeutic strategies for advanced breast cancer is well established. Translocatable receptors are categorised as 'functional'. In the absence of nuclear translocation the tumour may be scored as a false positive. Tissue from 114 primary breast carcinomas and 10 metastatic axillary lymph nodes from 59 pre- and 55 post-menopausal women was screened for oestrogen receptors with a cut-off value of 3 fmol and 10 fmol mg$^{-1}$ protein respectively, and 35 primary breast carcinomas were screened for nuclear oestrogen receptors with a cut-off value of 10 fmol 100 $\mu$g$^{-1}$ DNA, with the results as shown:

| *ER* | *Pre-menopausal* | *Post-menopausal* |
|---|---|---|
| Total cytosol + | 54% | 58% |
| Total nuclear + | 62% | 57% |
| Cytosol + nuclear + | 44% | 35% |
| Cytosol + nuclear − | 9% | 22% |
| Cytosol − nuclear + | 14% | 18% |
| Cytosol − nuclear − | 33% | 25% |

To rule out any defect in translocation step, the functional or translocatable ER were quantitated in a cross incubation study with breat cancer nuclei and receptor rich uterine cytosol. Data indicated that any tumour which contained cytosolic ER but had translocation defect might not be hormone dependent, while any tumour having low ER level and intact nuclear translocation step might respond to antioestrogen therapy. The study suggested that evaluation of functional ER level would reduce the number of tumours scored as false positive and false negative.

### Isolation of cloned cDNAs from mRNAs associated with tumour progression and metastasis in colorectal cancer

P. Elvin[1], I.B. Kerr[2], G.D. Birnie[1] & C.S. McArdle[3]

[1]*Beatson Institute for Cancer Research and University Departments of* [2]*Pathology and* [3]*Surgery, Royal Infirmary, Glasgow, UK.*

It is well recognised that the development of metastatic disease in colorectal cancer is the most critical prognostic factor. Furthermore, there is evidence to suggest that these tumours may behave consistently as metastasising or non-metastasising variants. However no clinically applicable means of predicting the metastatic potential of a primary tumour exists and the biological basis of metastasis remains poorly understood.

Phenotypic changes associated with the emergence of a metastatic population would be characterised by changes in gene expression. We have constructed cDNA libraries from the poly(A)$^+$ RNA of normal colonic mucosa and a liver metastasis from a colonic adenocarcinoma. Screening of these libraries using $^{32}$P-labelled cDNA probes from poly(A)$^+$ RNA of clinically and histologically documented samples of normal colonic mucosa, adenamatous polyps, adenocarcinomas and liver metastases by Grunstein-Hogness and dot blot hybridisation has identified a number of recombinant cDNA clones showing markedly altered expression in the metastases relative to normal and neoplastic colon.

These cDNA clones, and others identified in the libraries may be of considerable clinical importance both as diagnostic tools and in defining the phenotypic changes associated with tumour progression and metastasis.

### Rejoining of DNA strand breaks in BL6 murine melanoma and its metastases

G.V. Sherbet, S. Jackson & A.L. Harris

*Cancer Research Unit, University of Newcastle upon Tyne, Royal Victoria Infirmary, Newcastle upon Tyne NE1 4LP, UK.*

The metastatic variants of the BL6 murine melanoma show differences in sister chromatid exchange (SCE) compatible with their metastatic potential. The high metastasis variant BL6 shows higher SCE than the low metastasis variant F1. Intracranial tumours such as gliomas, which do not ordinarily metastasise to extracranial sites, show low levels of SCE. This suggests that genetic rearrangement may be associated with the evolution of the metastatic phenotype (Sherbet *et al.*, *J. Cell Biochem.*, Suppl. **10A**, 57, 1986). Since SCE occurs under conditions of reduced DNA repair, it may be predicted that primary tumours would repair DNA damage more rapidly than their metastases.

The rejoining of bleomycin-induced strand breaks in [$^3$H]-thymidine labelled DNA of BL6 and cell lines derived from its metastatic deposits (BL6-ML) was therefore examined by alkaline sucrose sedimentation. Degree of rejoining was quantified by determining % radioactivity found in high mol. wt DNA at various intervals of cell recovery and the half-maximal repair time. In BL6 cells, the rate of repair was $41 \pm 12\% \, h^{-1}$ ($n=5$). In contrast, in BL6-ML cells the rate of repair was $14 \pm 3\% \, h^{-1}$ (n=5). The difference was statistically significant ($P<0.005$) The half-max. repair time was 0.5 and 1.2 h respectively for BL6 and BL6-ML cells.

### Metastatic heterogeneity: Karyotypic evidence for genetic drift as a post-dissemination event

D.M. Teale & R.C. Rees

*Department of Virology, Sheffield University Medical School, Beech Hill Road, Sheffield S10 2RX, UK.*

The heterogeneity of primary tumours is now recognised as the probable origin of the diverse phenotypes associated with cell lines derived by spontaneous or artificial metastases. This phenomenon has been investigated further in a spontaneously metastatic HSV-2 induced hamster fibrosarcoma model, using *in vivo* and *in vitro* derived sublines, which differed with respect to their NK susceptibility, immunogenicity, metastatic potential and karyotypic make-up. Using flow cytometry, the diploid karyotype was consistently associated with the metastatic phenotype, whilst a higher ploidy number was correlated with the non-metastatic cell lines. The parent cell line however, shown to be weakly/non-metastatic, was uniformly tetraploid with no minority diploid population pre-existing within the tumour.

We conclude that in this model an unusually distinct and readily distinguishable genotype (diploid) is associated with the metastatic phenotype. In this system, metastasis would appear not to be the expression of strongly selected variant cells pre-existing within the primary neoplasm, but a genotypic drift towards the metastatic phenotype as a post-dissemination event.

### Analysis of cellular DNA content of fixed cell aspirates of breast carcinoma using flow cytometry

A. Owainati[1], R.A. Robins[1], M. Williams[2], C. Hinton[2], C. Dowle[2], R.W. Baldwin[1] & R.W. Blamey[2]

[1]*Cancer Research Campaign Laboratories and* [2]*Professorial Unit of Surgery, Nottingham University, Nottingham NG7 2RD, UK.*

The purpose of this study is to overcome inconvenience in preserving tumour cells, for DNA analysis, during the time lapse between sampling and analysis.

Cell samples from 29 human breast carcinomas were obtained by suction aspiration from the tumour mass. Each sample was divided into two. One aliquot was fixed in 10% formalin. Cells in the other aliquot were preserved fresh in sterile Eagle's minimal essential medium at 4°C until analysed. Fixed cells were washed twice with distilled water and digested with 0.5% pepsin. Isolated nuclei were stained with $1 \, \mu g \, ml^{-1}$ diamidinophenyl indol (DAPI). From the fresh sample, suspensions of tumour cells obtained by 1% collagenase were stained with a combination of mithramycin ($93.6 \, mg \, ml^{-1}$) and ethidium bromide ($37.5 \, mg \, ml^{-1}$). Both samples were analysed by flow cytometry (Becton Dickinson FACS IV).

Twenty-six of the 29 samples (90%) showed considerable modal DNA values. Those that did not, had poorly resolved histograms because of low numbers of cells. Further comparison between modal DNA values of single tumour cells dissociated from solid tumour and fresh suction aspirates from the same tumour, showed a similar high rate of comparability (48 out of 50, $P<0.0001$).

This study shows comparability between DNA modal values of solid tumours and suction aspirates, fresh or formalin fixed. This choice of preparation allows tumour samples to be preserved, stored and transported conveniently at room temperature before analysis. This, plus overall speed and patient acceptability, means that repeated sampling of inoperable tumours becomes feasible and tumour cell DNA can be monitored during cytostatic and endocrine treatment in relation to tumour progression.

### Flow cytometric analysis of the DNA content of primary breast carcinomas: Relationship to survival and other prognostic parameters

A. Owainati[1], R.A. Robins[1], C. Hinton[2], M. Williams[2], I. Ellis[3], C.W. Elston[3], R.W. Baldwin[1] & R.W. Blamey[2]

[1]*Cancer Research Campaign Laboratories,* [2]*Professorial Unit of Surgery and* [3]*Department of Pathology, University of Nottingham, Nottingham HG7 2RD, UK.*

Cellular DNA content of primary tumours from 268 patients with operable breast carcinoma was determined by flow cytometry using nuclei from paraffin sections stained with DAPI. Seventy-three of them have been further analysed for their DNA content using single suspension of fresh tumour cells stained with mithramycin and ethidium bromide. One hundred and ninety-nine were followed for 7–13 years after surgery. Histological grading, according to Bloom and Richardson, was on a scale of 1 to 3, well to poorly differentiated tumours respectively. Mantel's life table analysis was used to compare survival between different patient groups.

Overall 66% of the tumours contained aneuploid populations. Survival was not significantly different in patients with diploid and aneuploid tumours. Aneuploidy was, however, significantly related to histological grade, as has been found by others. Thus 11/49 (22%) grade 1, 55/97 (59%) grade 2, and 92/125 (72%) grade 3 tumours were aneuploid. In view of the clear relationship between histological grade and survival in this series of patients, it is surprising that there is no relationship between aneuploidy and survival. More detailed analysis shows that aneuploidy is significantly associated with better survival in grade 2 patients ($P<0.01$); a similar trend is observed in grade 1 patients, but no difference was observed in grade 3 patients. Comparisons between the modal DNA values of fresh and paraffin embedded samples showed a high rate of comparability (66/74 $P<0.0001$). These studies show that tumour aneuploidy is not associated with poor survival in primary breast carcinoma. There is a complex relationship between histological grade, aneuploidy, and survival. Aneuploidy is associated with better survival in patients whose tumours are not poorly differentiated.

### Changes in cellular DNA content in mucosa adjacent to colonic carcinoma

J. Matthews & T. Cooke

*Department of Surgery, Charing Cross and Westminster Medical School, London W6 8RF, UK.*

We have previously reported that in an animal carcinogenesis model the mean DNA content per epithelial cell in the upper regions of the colonic crypts increases as carcinogenesis progresses. We have now studied the DNA content of colonic mucosa in patients with colorectal carcinomas.

Using microdensitometry, DNA content was measured in the cells in the proliferative and functional zones of histologically normal Feulgen stained sections taken adjacent to and distal from colonic carcinomas, and related to stem cell DNA content. DNA content was measured similarly in cytological brushings from the same areas and the percentage of diploid cells calculated.

There was a significant increase in the amount of DNA in the proliferative cells adjacent to the tumours (100% $\pm$1.3) compared to distally (91% $\pm$2.0, $P<0.002$). Although similar increase was seen in the functional cells adjacent to the tumours compared to distally the difference was not significant. In cytological preparations there was an increase in the proportion of dividing or aneuploid cells in the transitional mucosa (7.3% $\pm$1.2) compared to distal mucosa (3.5% $\pm$0.9, $P<0.02$) and to the mucosa of patients with non-cancer related bowel problems (2.1% $\pm$0.5, $P<0.001$).

These results support the concept of field changes in premalignant mucosa.

### Influence of sialomucins at the resection margin on survival of patients with colorectal cancer

P.M. Dawson[1], N.A. Habib[2], J.B. Bradfield[3], R.C.N. Williamson[2] & C.B. Wood[1]

[1]*Department of Surgery, Royal Postgraduate Medical School, London and Departments of* [2]*Surgery and* [3]*Pathology, Bristol Royal Infirmary, Bristol, UK.*

In a multicentre prospective trial, 281 patients undergoing 'curative' resection for colorectal cancer were followed for a mean of 13.6 months (s.d. 7.2 months). The presence or absence of sialomucin at the resection margin was studied histochemically using the high iron diamine – alcian blue (HID-AB) stain. There were 49 deaths relating to tumour recurrence: 21 in the sialomucin positive group ($n=77$) and 28 in the sialomucin negative group ($n=204$) ($P<0.02$). Life table survival was correlated against the presence or absence of sialomucin in the resection margin. At the mean follow up (13.6 months) 85.6% of patients were alive in the sialomucin negative group, and 76.4% of patients were alive in the sialomucin positive group. Regression analysis predicts 32.8% and 18.9% five year survivals for sialomucin negative and positive groups respectively. There was no significant statistical correlation between the presence of sialomucin in the resection margin and the Duke's staging, site or tumour differentiation. The appearance of sialomucin in either resection margin appears to be an early marker of poor prognosis for patients with colorectal cancer.

**Comparison of plasminogen activator activity between transformed hamster cell lines of varying metastatic potential**

I.A. Khidair, R.C. Rees, D.M. Teale & C.W. Potter

*Department of Virology, University of Sheffield Medical School, Beech Hill Road, Sheffield S10 2RX, UK.*

Elevated levels of plasminogen activator (PA) have been strongly associated with the degradative processes involved in malignancy and a role for PA in the metastatic process has been inferred. In the present study the ability of a primary HSV-2 induced hamster fibrosarcoma and sublines derived from its *in vitro* metastases, to produce enzymes was investigated using the indirect $I^{125}$-labelled fibrin plate method. Fresh tissue culture lines established from primary HSV-2-333-2-26 tumours were shown to produce similar levels of PA to its sublines derived from lung or kidney foci which developed following resection of primary tumours. In comparison, normal hamster embryo fibroblast (NHEF) and baby hamster kidney cells produced little or no PA, although baby hamster lung fibroblasts produced intermediate levels of PA. Coculturing of tumour cells with normal baby hamster lung fibroblasts, however amplified the PA secretion in some cell lines.

We conclude that although no correlation between PA secretion and the metastatic process was detected, PA production can be stimulated when tumour cells and normal cells are co-cultured *in vitro*.

**The lack of structural requirements for activity in a series of solvents which induce the terminal differentiation of HL-60 cells**

S.P. Langdon[1], E. O'Reilly[2] & J.A. Hickman[2]

*[1]ICRF Medical Oncology Unit, Western General Hospital, Edinburgh EH4 2XU and [2]CRC Experimental Chemotherapy Group, Aston University, Birmingham B4 7ET, UK.*

We have previously reported that in a series of N-alkylformamides, acetamides and ureas which induce terminal differentiation of HL-60 human promyelocytic cells to granulocyte-like cells, there was a correlation between potency and the mol. wt of the compounds (Langdon & Hickman, *Br. J. Cancer*, **51**, 601, 1985). We have extended this study and find that the potency of solvents with unrelated molecular features is also predictable in this way. $5 \times 10^4$ HL-60 cells $ml^{-1}$ were incubated continuously for 96 h with each solvent at 37° in RPMI medium 1640 with 10% foetal calf serum. Biochemical and functional tests of differentiation were made at 96 h, (the ability to produce superoxide and phagocytose yeast). In addition to solvents like dimethylsulphoxide, dimethylformamide and dimethylacetamide methanol, ethanol and acetone were found to be potent inducers of differentiation (>70% cells NBT+) and their activity fitted a regression line ($r=0.96$) which related the logarithm of the optimal concentration to promote differentiation and mol. wt. All cells which underwent differentiation went through at least one doubling. When cell doubling was inhibited by high concentrations of solvent (i.e. no increase over $5 \times 10^4$ cells $ml^{-1}$) viable cells did not differentiate and a linear, parallel, relationship to the one above was found between the logarithm of the concentration to bring about cytostasis, without differentiation, and mol. wt. No correlation existed between potency and the octanol-water partition coefficient for each agent. We conclude that a sub-toxic challenge by solvents, irrespective of their structure, to HL-60 cells is sufficient to induce differentiation.

**Reversal of HL-60 leukaemic status by the activation of suppressed differentiation factor(s)**

B. Djulbegovic, S.E. Christmas & M. Moore

*Immunology Department, Paterson Laboratories, Christie Hospital and Holt Radium Institute, Manchester M20 9BX, UK.*

The human promyelocytic cell line HL-60 can be induced to differentiate along the granulocytic or monocytic pathways. The recent findings that HL-60 can be induced to differentiate by various physiological compounds (Metcalf, *Leukaemia Res.*, **7**, 117, 1983) as well as by conditioned medium from normal blood cells (Harris *et al.*, *Cancer Res.*, **45**, 3090, 1985) support the concept that leukaemogenesis is not an autonomous process but rather a regulated one with an abnormal set of control parameters. If this is the case, then in some instances a leukaemic system, once corrected by exogenous factors, e.g. those inducing differentiation, may then be capable of self-regulation by production of endogenous factor(s). We have shown that conditioned medium produced by HL-60 cells which had been induced to differentiate into monocyte/macrophages by $1.25(OH)_2$ Vit.$D_3$ was itself capable of inducing differentiation in fresh HL-60 cells. Differentiation was measured by induction of the monocyte enzyme non-specific esterase as well as the monocyte/macrophage antigens recognised by Mo2, EB11 and OKM-1 monoclonal antibodies. Conditioned medium acted in a dose dependent manner and also abolished HL-60 clonogenicity in a soft agar assay indicating that it was acting on leukaemia progenitor cells. Activity was shown not to be due to residual vitamin $D_3$, $\gamma$-interferon or CSF. A possible molecular mechanism might involve switching on a gene for the production of differentiation factor(s) which then compete with growth factor(s) autonomously produced by the undifferentiated cell population.

### Effects of N-methylformamide (NMF) on the glutathione status and cell cycle of TLX5 murine lymphoma cells *in vitro*

C. Bill, J.A. Hickman & A. Gescher

*CRC Experimental Chemotherapy Group, Pharmaceutical Sciences Institute, Aston University, Birmingham B4 7ET, UK.*

NMF is an experimental antitumour agent which has good activity against murine tumours *in vivo* yet has no effect on the growth of the bone marrow (Langdon *et al.*, *Eur. J. Cancer Clin. Oncol.*, **21**, 745, 1985). NMF is also one of many solvents which promotes the terminal differentiation of certain malignant cells *in vitro*. We wished to determine whether the *in vivo* and *in vitro* activities of NMF were related. $5 \times 10^4$ TLX5 cells $ml^{-1}$ incubated continuously *in vitro* with NMF over 72 h showed a progressive loss of viability and a decrease in cell growth rate over the concentration range 43 mM to 170 mM (50% growth inhibition = 68 mM). 106 mM NMF incubated for 48 h caused a 37% growth inhibition of the TLX5 cells but >80% were viable (trypan blue exclusion). Drug removal led to cells regaining normal growth characteristics. Cell cycle analysis of these cells by fluorescence activated cell sorting showed a decrease in the proportion of S and $G_2M$ phase cells with a concomittant increase in the $G_1$ population. Higher NMF concentrations (>150 mM 48 h), although causing a progressive decrease in viability, resulted in complete loss of S phase cells with 99% of cells in $G_1$. Murine lymphoma cells have been shown to be arrested in the $G_1$ phase by reduction of cellular glutathione (Ishii *et al.*, *Cell Struct. Funct.*, **10**, 89, 1985). In TLX5 cells incubated with 106 mM NMF for 48 h total intracellular glutathione (measured according to Griffiths, *Analyt. Biochem.*, **106**, 207, 1980) was reduced to 19.7 ± 6.4% of controls. We consider the fall in intracellular glutathione may be responsible for the reversible decrease in cell growth rate but that NMF does not terminally differentiate TLX5 lymphoma cells *in vitro*.

### The expression of tartrate resistant acid phosphatase in a series of B-non-Hodgkin's lymphoma stimulated with phorbol ester

S. Schrape, D.B. Jones & D.H. Wright

*University Department of Pathology, General Hospital, Southampton SO9 4XY, UK.*

Previous studies describe that the treatment of CLL cells with phorbol ester leads to the expression of tartrate resistant acid phosphatase. This enzyme is a marker for hairy cell leukaemia. This transformation is significant to the relationship of the 2 disease types. CLL cells frequently express Leu-1, as do cells from diffuse centrocytic lymphoma and we were, therefore, interested to see if centrocytic lymphoma cells are also TRAP positive after treatment. All cells were dispersed from fresh lymph node biopsies and were cultured for 48 h with, and without, phorbol ester. Cytocentrifuge preparations were stained for TRAP by a standard technique. Seven cases of CLL became TRAP positive in more than 20% of the cells following this treatment. The percentage of TRAP positivity occasionally approached 90%. Treatment of 5 cases of centrocytic lymphoma resulted in TRAP positivity in 20–50% of the tumour cells. However, when this study was extended to lymphomas classified as centroblastic/centrocytic or centroblastic/diffuse, cytoplasmic TRAP positivity was also seen in many of the tumour cells. These results suggest that the appearance of TRAP after phorbol stimulation does not specifically relate to the relative positions in differentiation of CLL cells and hairy cells. This observation was confirmed when all cells cultured were stained with a group of antibodies known to identify hairy cell leukaemia. In no case was the appearance of TRAP positivity associated with the demonstration of hairy cell antigens.

### A comparison of the effects of differentiation inducers against human ovarian adenocarcinoma cell lines

S.P. Langdon, I.P. Hayward, S.S. Lawrie & J.F. Smyth

*Imperial Cancer Research Fund Medical Oncology Unit, Western General Hospital, Edinburgh, UK.*

We wish to identify inducers of terminal differentiation (TD) in human ovarian carcinoma. No conclusive marker of TD in this disease has yet been found. However lipid (L) production has been proposed as a possible marker of this process (Buick, *J. Cell Physiol.*, suppl. **3**, 117, 1984). In colon cell lines, increases in alkaline phosphatase (AP) activity correlate with changes toward a more benign phenotype (Tsao *et al.*, *Cancer Res.*, **42**, 1052, 1982). Three agents, sodium butyrate (SB), retinoic acid (RA) and dimethylsulphoxide (DMSO), capable of inducing TD in leukaemic cell lines were studied against two human ovarian adenocarcinoma cell lines derived from the same patient before (PE01 cells) and after (PE04 cells) the onset of clinical resistance to chemotherapy. Cells ($4 \times 10^4$ 35 mm$^{-1}$ well) were plated in 10% FCS/RPMI. Three days later drugs were added. Four days later cells were stained and assessed for viability. Cells producing L and AP were identified histochemically (see Table). At cytostatic concentrations, SB produced increases in the number of PE04 cells expressing AP and L relative to control cells. Furthermore preliminary experiments with monoclonal antibodies indicate changes in antigen expression. Experiments are underway to assess whether these effects produced by SB are truly indicative of TD in ovarian carcinoma.

| | | *% cells +ve* | | | |
|---|---|---|---|---|---|
| | | *PE04 cells* | | *PE01 cells* | |
| *Inducer* | *Cytostatic conc.* | *L* | *AP* | *L* | *AP* |
| Control | — | 7±2 | 6±2 | 9±3 | <1 |
| SB | 2 mM | 84±6 | 38±8 | 81±11 | <1 |
| RA | 30 μM | 69±11 | 5±2 | 79±6 | <1 |
| DMSO | 380 mM | 1±1 | 4±2 | — | — |
| | 250 mM | — | — | 1±1 | <1 |

### Rat hepatic glutathione S-transferase isoenzymes and aflatoxin carcinogenesis *in vivo* and cell transformation *in vitro*

G.E. Neal[1], U. Neilsch[2] & D.J. Judah[1]

[1]*MRC Toxicology Unit, Carshalton and* [2]*Department of Pharmacology, Bristol University, Bristol, UK.*

The potent hepatocarcinogen, aflatoxin $B_1$, ($AFB_1$) requires metabolic activation to exert its toxic and carcinogenic effect. Detoxification can occur by glutathione S-transferase mediated conjugation with reduced glutathione (GSH). GSH-S-transferase isoenzymes have been fractionated on IEF gels followed by detection using model substrate (CDNB DCNB and monobromobimane) and assay of $AFB_1$-conjugating capacity using microsomally activated $AFB_1$. Using fractionated cytosol from control male rat liver, $AFB_1$-conjugating activity was associated with fractions having IEP ~8.8, 6.5 and 6.4. Development of pre-neoplastic and neoplastic liver lesions, by feeding $AFB_1$, results in a resistance to cytotoxicity of $AFB_1$. Total liver cytosol fractions from these animals had increased (>100%) conjugating activity towards the model substrates and towards $AFB_1$. IEF showed that the increased activity towards the model substrates was due to increased levels of the basic isoenzymes (1:1, 1:2 and 2:2), the less basic 3:3, and the appearance of the 7:7 isomer. The identity of 7:7 and its absence from fractions from control livers was confirmed by Western blotting coupled with identification by specific antibody. The 7:7 isoenzyme did not catalyse conjugation of $AFB_1$ to any significant extent. The increased activity ($AFB_1$) in preneoplastic and neoplastic tissue was mediated by the basic isoenzymes (1:1, 1:2 and 2:2). In rat liver derived epithelial cell line, transformation *in vitro* by activated aflatoxin or *ras* transfection also resulted in increased levels of GSH-S-transferase activity (>500%) as assessed by the model substrate. Increased level of the 7:7 isoenzyme in the *in vitro* transformed cells was also demonstrated by Western blotting.

### Assessment of aflatoxin exposure in rats and man with the aid of anti-aflatoxin antibodies

F. Tursi[1], C.P. Wild[2], R. Montesano[2] & R.C. Garner[1]

[1]*Cancer Research Unit, University of York, Heslington, York YO1 5DD, UK and* [2]*IARC, 150 Cours Albert-Thomas, Lyon, France.*

We are interested in determining whether or not we can devise a non-invasive technique to measure aflatoxin (AF) binding to cellular macromolecules in man in order to examine individual differences.

We have used the rat as an experimental model for our studies prior to assaying human urine samples for AF. AF administration to rats results in covalent binding to cellular and tissue macromolecules. In addition, AF metabolites are excreted in the urine and faeces. Correlations have been found between species susceptibility or resistance to the hepatocarcinogenicity of AF and how much carcinogen is covalently bound to liver DNA. After giving single doses of ($^3$H)AFB$_1$ to rats, we have found that there is a dose-related increase in liver DNA binding. Doses of 10, 100 and 200 $\mu g\,kg^{-1}$ by oral intubation resulted in 178, 1,638 and 2,401 $pg\,mg^{-1}$ liver DNA being bound respectively. Binding to serum albumin gave values of 49, 506 and 719 $pg\,mg^{-1}$ protein respectively for these doses. The liver DNA/serum albumin binding ratio for 10, 100 and 200 $\mu g\,kg^{-1}$ was 3.7, 3.2 and 3.4. After daily administration of 1 $\mu$g AF for 24 days (excluding weekends) the liver DNA binding peaked by day 14 with values of liver DNA and serum albumin binding of 260 and 150 $pg\,mg^{-1}$ respectively. Once this plateau phase had been reached, the ratio of 1.7 liver DNA/serum albumin binding remained constant. Urine and faecal analysis indicated that, in chronically dosed animals, the daily amounts of AF metabolites excreted were constant, except after days in which AF was not administered, when the amounts excreted fell.

Instead of using radioactive tracer to measure covalent binding, an immunoassay procedure and a rabbit anti-aflatoxin antibody has been utilised. A reasonable correlation between radiotracer measurement and immunoassay results was seen; the only exception to this was when urine was analysed. In all cases AF concentration as measured by immunoassay over-estimated the urine AF concentration, probably due to some metabolites having higher affinity for the antibody than AF. Using these immunoassay procedures we have examined urine from persons thought to be exposed to AF in the Gambia.

### Transformation of rat liver derived epithelial cells *in vitro*

S. Sinha[1], G.E. Neal[1], C.J. Marshall[2] & L.J. Hockin[1]

[1]*MRC Toxicology Unit, Carshalton, Surrey SM5 4EF and* [2]*Chester Beatty Labs., Institute of Cancer Research, Fulham Road, London, UK.*

A rat liver derived epithelial cell line (BL$_8$) was transformed *in vitro* by *ras* oncogenes and by aflatoxin B$_1$ (AFB$_1$). Ha-*ras* and N-*ras* plasmids, in conjunction with genes for drug (G418) resistance were introduced into cells by transfection, and colonies selected out by resistance to G418 (Sinha *et al., Cancer Res.*, **46**, 1440, 1986). Colonies of transformed cells had an altered appearance and tended to pile up in culture. To transform cells with aflatoxin, cells partially synchronised in S phase were treated with AFB$_1$ activated by the presence of quail microsomes and a NADPH generating system. Cells transformed with aflatoxin had a morphology similar to cells transformed with *ras* oncogenes. In cells transformed by both methods there was a strong association of the acquisition of a neoplastic phenotype, as shown by anchorage independent growth and mouse tumourigenicity, with the induction of $\gamma$-glutamyl transferase (GGT) which is a sensitive marker for the preneoplastic and neoplastic changes in rat hepatocarcinogenesis. A heterogeneity in staining for GGT was seen even in the early passages of transformed cells. Further subcloning showed that while there is a strong association of GGT with cell transformation in early passages, altered levels of the enzyme do not influence tumourigenesis in the nude mouse.

### The induction of specific mRNAs during aflatoxin B$_1$-induced hepatocarcinogenesis

C.A. Power[1], J.L. Simpson[1], M.M. Manson[1], Y. Laperche[2] & G. Guellaen[2]

[1]*MRC Toxicology Unit, Carshalton, Surrey SM5 4EF, UK and* [2]*Henri Mondor Hospital, Creteil, France.*

We are using aflatoxin B$_1$ (AFB$_1$)-induced carcinogenesis in rat liver and liver-derived cell lines to examine alterations in transformed cells with particular reference to actin (an abundant mRNA species) and $\gamma$-glutamyl transferase (GGT) (a low abundance mRNA species).

Recently, it has been suggested that changes in the level of specific forms of actin may be a marker of transformation in fibroblasts (Leavitt *et al., Nature*, **316**, 840, 1985). We have used Northern and dot blot analysis ot total RNA to study changes in actin expression. Nick translated probes prepared from a plasmid pRT3 (containing an 11 kb genomic clone of mouse DNA recognising and actin) revealed a 2–3 fold elevation of actin mRNA in AFB$_1$-treated liver and hepatoma. A hepatoma-derived, highly tumourigenic cell line, JB$_1$ contained ~8 times more actin mRNA than untreated hepatocytes.

GGT has for many years been used as a tumour marker. In normal adult rat liver it is confined to bile duct epithelia, but appears in preneoplastic foci of hepatocytes and tumours upon administration of $AFB_1$. Using a rat kidney GGT cDNA probe, we have shown by Northern blot analysis of poly $A^+$ mRNA that one form of GGT mRNA is elevated in $AFB_1$-treated liver and in a hepatoma-derived cell line, and is not detectable in primary hepatocytes or a cell line derived from normal liver.

These results indicate that transformation is accompanied by changes in expression of high abundance mRNAs as well as of low abundance species. Although actin and GGT may not play a central role in the carcinogenic process, they may be important in maintaining the transformed phenotype.

We thank Dr. K. Willison (ICR, London) for providing plasmid pRT3.

**Immunohistochemical detection of *ras* oncogene p21 product in human benign and malignant mammary tissue**

A.K. Ghosh[1], M. Moore[1] & M. Harris[2]

[1]*Department of Immunology, Paterson Laboratories and* [2]*Department of Pathology, Christie Hospital & Holt Radium Institute, Manchester M20 9BX, UK.*

Although activated *ras* oncogenes have been detected in a wide variety of human tumours and transformed cell lines in culture, controversy remains whether *ras* activation is a primary event in carcinogenesis or appears during tumour progression. With the introduction of monoclonal antibodies to *ras* oncogene products it is now possible to examine *ras* expression in pre-malignant and malignant conditions at the cellular level and to determine if there is a correlation between enhanced *ras* expression and a particular neoplastic state. We have used an immunohistochemical technique to study the expression of *ras* p21 in 2 cases of normal breast tissue, 23 benign and 22 maglignant breast epithelium using the monoclonal antibody RAP-5 generated against a synthetic peptide corresponding to amino acid positions 10–17 of the *ras* p21 protein. The staining intensity and intracellular distribution of RAP-5 was similar between the three epithelial populations and extended to other tissue elements including myoepithelial cells, smooth muscle, myelin, capillary endothelium and stromal fibroblasts as well as sebaceous glands and sweat glands overlying the breast. These results suggest that RAP-5 recognises a normal cellular component the expression of which is not significantly amplified in hyperplastic or neoplastic conditions. The expression of mutant forms of *ras* p21 exclusively expressed in malignant tumours would not be detected by this reagent.

**Transcription of the c-*fos* oncogene in the adenoma-carcinoma sequence in colorectal cancer**

P. Elvin[1], P. Kelly[2], G.D. Birnie[1] & I.B. Kerr[2]

[1]*Beatson Institute for Cancer Research, and* [2]*University Department of Pathology, Royal Infirmary, Glasgow, UK.*

Transcription of the c-*fos* oncogene has been described in a number of human malignancies, including carcinomas of the colon and rectum, but its significance in colorectal carcinogenesis is not clear. Ue have isolated total cellular RNA from specimens of normal colonic mucosa, adenomas and carcinomas, and have examined the level of c-*fos* homologous RNA by a doubling dilution dot blot assay using a $^{32}P$ nick-translated c-*fos* probe. Equivalent steady state levels of c-*fos* RNA were found in mucosa, adenoma, and carcinoma specimens, comparable to those in TPA induced (differentiated) HL-60 cells. In contrast, steady state levels of c-*myc* RNA were found to be considerably lower in all specimens examined, although the levels of c-*myc* RNA in carcinomas were generally higher than in mucosae and adenomas.

The presence of both c-*fos* and c-*myc* homologous RNAs in these tissues was also demonstrated by *in situ* hybridisation using [$^3H$]-labelled c-*fos* and c-*myc* probes. Both oncogene transcripts were detected throughout the epithelial crypt population as well as in stromal cells, suggesting that the transcription of the two oncogenes is not confined to proliferating cells. No differences in cell specific location of either oncogene probe was observed when the grain count distribution over epithelial and stromal elements in both normal mucosa and adenoma specimens were compared.

Thus a comparison of steady state RNA levels and of tissue localisation of oncogene transcripts suggest that c-*fos* RNA levels do not correlate with any stage of colorectal carcinogenesis.

### Correlation between staining for p21 product of K-*ras* oncogene and histological degrees of dysplasia in human colonic epithelium

J.W.B. Bradfield[1], G.U.A. Igboaka[1], N.A. Habib[2], R.C.N. Williamson[2], P.M. Dawson[3] & C.B. Wood[3]

*Departments of [1]Pathology and [2]Surgery, Bristol Royal Infirmary, Bristol and [3]Department of Surgery, Royal Postgraduate Medical School, London, UK.*

The K-*ras* oncogene has been associated with human colonic cancer in transfection studies. The object of this work was to investigate the histological distribution, and intensity of expression, of the p21 polypeptide product of this oncogene in normal and neoplastic colonic epithelium. The peptide was detected by an indirect immunoperoxidase technique in formalin-fixed paraffin-embedded sections using a monoclonal antibody directed against the amino acid sequence 96–118 of a synthetic p21 peptide (Scripps Research Center, La Jolla, USA). Cases were categorised histologically by examination of routinely stained sections; further sections were then stained for the p21 polypeptide. In the normal colonic epithelium there was some positive patchy staining in the surface epithelium with minimal staining of the base of the crypts. In contrast, with increasing degrees of dysplasia there was an increase in the distribution and intensity of staining, particularly within the crypts.

| | | | *Staining at base of crypts* | |
|---|---|---|---|---|
| | | *n* | *Intensity (Number with marked staining)* | *Distribution (Number with >50% positive)* |
| Normal | | 20 | 0 | 0 |
| Metaplastic | | 21 | 1 | 1 |
| Dysplastic | mild | 6 | 1 | 2 |
| | moderate | 27 | 11 | 14 |
| | severe | 32 | 16 | 22 |

The results show a correlation between increasing expression of p21 and increasing degrees of dysplasia in neoplastic human colonic epithelium.

### *myc* and *ras* expression in experimental carcinogenesis

N.A. Habib[1], R. Salem[2], R.C.N. Williamson[1], C.B. Wood[2] & K. Sikora[3]

*[1]Department of Surgery, Bristol Royal Infirmary, Bristol, [2]Royal Postgraduate Medical School, London and [3]Ludwig Institute for Cancer Research, Cambridge, UK.*

Activation of more than one oncogene is responsible for cancer formation. Overexpression of *myc* oncogene occurs early and is responsible for the immortalisation of the cell. Activation of *ras* oncogene is responsible for tumour propagation and is thought to be a late event in the multistep carcinogenic process. Using immunohistochemical techniques we studied the distribution of p62 (for *myc*) and p21 (for *ras*) in neoplastic and non-neoplastic colonic tissue in 42 rats undergoing chemically induced carcinogenesis with $20\,mg\,kg^{-1}$ dimethylhydrazine (DMH). We found that both p62 and p21 were absent in 10 control rats injected with normal saline solution and present in 27 rats with colonic carcinomas. In the group of rats treated with DMH without tumour formation 11 of 15 had p62 without p21 overexpression. These experimental findings suggest that both *myc* and *ras* are overexpressed in carcinomas and that *myc* activation is an early event in this experimental model as it was expressed before the development of overt carcinomas. Therefore *myc* oncogene overexpression could have useful clinical application as it may be of potential value in screening patients at risk of malignant change.

### Effect of dietary β-naphthoflavone on DNA alkylation in the rat large intestine after 1,2-dimethylhydrazine

A.M. Tacchi-Bedford, G.D. Whyman & A.E.M. McLean

*Toxicology Laboratory, Department of Clinical Pharmacology, University College London, University Street, London WC1E 6JJ, UK.*

Many vegetables, especially the Brassicas, have powerful inducing effects on the mixed function oxidase activity of the large intestine. This can be reproduced by feeding a diet containing 50 ppm β-naphthoflavone (BNF) which causes a three fold increase in MFO activity in one week (McDanell & McLean, *Biochem. Pharmacol.*, **33,** 1977, 1984). We studied the effect of feeding BNF on the large intestine DNA alkylation induced by the colon carcinogen 1,2-dimethylhydrazine (DMH) in male Wistar rats. After a week on a BNF containing diet, or a semi-purified diet with minimal inducing activity (MID), the rats were injected with $20\,mg\,kg^{-1}$ [$^{14}$C]DMH subcutaneously (~40 μCi)

and killed at various time intervals up to 24 h. DNA was isolated from the colon mucosa by phenol extraction and the abnormal purines N7- and O6-methylguanine were measured by HPLC and liquid scintillation counting or fluorimetric detection. Both N7 and O6 MeG were present to a greater extent in the BNF rats than in the MID group. At 6 h when the peak of alkylation was reached, N7 MeG accounted for 1,100 and 700 $\mu$mol mol$^{-1}$ G in the BNF and MID animals respectively. O6 MeG, however, was detectable only in the BNF group, with values in the range of 52–93 $\mu$mol mol$^{-1}$ G between 1–12 h. These results suggest that enzyme induction, metabolic activation of a carcinogen and covalent binding to DNA in the large intestine may be directly correlated and that composition of the diet may play an important role in the process of colon tumour initiation.

### Proliferative instability and neoplastic development at the site of a colonic anastomosis in experimental carcinogenesis

R. Roe & R.C.N. Williamson

*Department of Surgery, Bristol Royal Infirmary, Bristol BS2 8HW, UK.*

Colonic anastomoses are common sites for 'recurrent' cancer in man and for chemically-induced cancer in the rat. The possibility that proliferative instability around the anastomosis during the healing period promotes neoplastic development at the suture line was investigated. Sprague-Dawley rats received the first of a 5 week course of azoxymethane (total dose, 50 mg kg$^{-1}$) either immediately after transection of the descending colon or at 2, 4, 8 and 12 weeks later. Control rats received handling of the bowel. The proliferative status of colonic crypts was assessed by autoradiography following [$^3$H]TdR injection. All rats were killed 28 weeks after the first azoxymethane injection. Susceptibility to develop tumours decreased with age in both control and transected groups. However, a constant overall increase (44%) in the proportion of tumours was found in transected groups which was due to the presence of anastomotic tumours. No detectable changes in morphology or proliferative index of colonic crypts occurred between young control rats and those 12 weeks older. Changes in both parameters were observed in transected rats over time, these being most dramatic in crypt positions 1–10 away from the anastomosis. Crypts at this site were increased in height at 2, 4 and 8 weeks ($P<0.001$) respectively but returned to normal morphology by 12 weeks. Similarly, crypt labelling indices were increased at 2, 4 and 8 weeks ($P<0.001$) respectively and although diminished by 12 weeks remained higher ($P<0.05>0.02$) than control levels.

These findings suggest that increased crypt cell proliferation in the immediate vicinity of morphologically 'healed' anastomoses contributes to the susceptibility of this site for tumour development.

### Hypothermia produces intestinal adaptation without promoting experimental intestinal carcinogenesis

J.B. Rainey, P.W. Davies & R.C.N. Williamson

*University Department of Surgery, Royal Infirmary, Bristol, UK.*

Adaptive hyperplasia stimulated by intestinal operations enhances experimental intestinal carcinogenesis (Williamson & Rainey, *Scand J. Gastrienterol.*, **19**, suppl. 104, 57, 1984), but the effects of non-surgical promoters of mucosal growth, such as hypothermia (Heroux & Gridgeman, *Can. J. Biochem. Physiol.*, **36**, 209, 1958), are uncertain. The tropic and tumour-promoting potential of hypothermia were tested in two groups of male Sprague-Dawley rats housed at 10° C for 30 weeks. One group ($n=10$) received a 6 week course of azoxymethane (total dose 90 kg mg$^{-1}$); the other group ($n=7$) acted as hypothermic controls. Two further groups, kept at 22° C, received azoxymethane ($n=15$) or served as normothermic controls ($n=15$). Mean daily food intake was 42% higher in the hypothermic groups, yet at sacrifice mean body weight was 11% lower than in the normothermic groups ($P<0.01$). The combination of hypothermia and azoxymethane increased crypt cell production rate (as determined stathmokinetically) by 170% in the duodenum, 172% in the jejunum, 74% in the ileum and 227% in the proximal colon, compared with normothermic controls ($P=0.05$–$0.01$). Individually, azoxymethane and hypothermia had no such effect. In rats receiving azoxymethane, intestinal tumour yield did not differ significantly between the hypothermic group ($2.3\pm0.8$ tumours/rat: mean $\pm$ s.e.) and normothermic ($1.7\pm0.3$) group. Hypothermia causes hyperphagia and adaptive hyperplasia in small bowel and right colon when combined with azoxymethane, but does not promote carcinogenesis.

### Faecal steroids and colorectal cancer: Faecal bile acids in Scandinavian populations at different risk

R.W. Owen, C. Walton, M.J. Hill & M.H. Thompson

*PHLS-CAMR, BMRL, Salisbury, Wiltshire SP4 0JG, UK.*

Some bile acids, especially lithocholic acid (LCA) and deoxycholic acid (DCA) have been shown to act as tumour promoters in animal models of colorectal cancer (CRC). These observations support the established correlation between risk of developing CRC and the consumption of a diet depleted in fibre and containing high levels of fat and protein; a diet that will undoubtedly influence the excretion of faecal bile acids (FBA). In this IARC study the FBA excretion patterns were determined in groups of healthy male volunteers from four different Scandinavian populations with a four-fold variation in the risk of developing CRC. Unlike many earlier population studies which encompassed groups exhibiting extreme differences in life style this investigation compares the relationship between diet, faecal steroid profiles and CRC risk in comparable populations. Whilst the marker of individual risk, namely the LCA/DCA ratio (Owen *et al., Br. J. Cancer*, **52**, 445, 1985), is on average low throughout these populations, the high risk Danish population excrete LCA and DCA at higher concentrations. That is, the high risk group is exposed to higher concentrations of known tumour promoters.

| *Population* | *No.* | *LCA*[a] | *DCA* |
|---|---|---|---|
| Perrikala (F) | (30) | 1.96±1.53 | 3.11±2.04 |
| Helsinki (F) | (29) | 1.96±1.07 | 3.11±2.97 |
| Them (D) | (30) | 1.68±1.12 | 3.05±2.64 |
| Copenhagen (D) | (29) | 3.09±1.66 | 5.33±3.12 |

| *Population* | *LCA+DCA* | *LCA/DCA* | *CRC inc.*[b] |
|---|---|---|---|
| Perrikala (F) | 5.07±3.31 | 0.72±0.46 | 14.2 |
| Helsinki (F) | 5.06±2.97 | 0.74±0.35 | 25.7 |
| Them (D) | 4.74±3.60 | 0.72±0.46 | 27.9 |
| Copenhagen (D) | 8.42±4.58 | 0.64±0.29 | 42.1 |

[a]$mg\,g^{-1}$ dry faeces; [b]Cases $10^{-5}$ males p.a. (age adjusted); F = Finland; D = Denmark.

### Is intragastric nitrosation responsible for gastric cancer?

P.W.J. Houghton[1], R.W. Owen[2], S. Leach[2], N.J.McC. Mortensen[1], M.J. Hill[2] & R.C.N. Williamson

[1]*Department of Surgery, Bristol Royal Infirmary and* [2]*PHLS Centre, Porton Down, UK.*

Intragastric nitrosation, with the formation of carcinogenic N-nitrosocompounds, may be responsible for the pathogenesis of gastric carcinoma in patients with pernicious anaemia and those who have undergone gastric surgery for peptic ulcer disease. The evidence for nitrosation is controversial: some workers report high levels of intragastric nitrosamines but others find no such increase. We have assessed intragastric nitrosation in 35 at-risk patients and 11 controls by measuring their 24 h urinary excretion of N-nitrosoproline (N-NP). Following ingestion of 300 mg sodium nitrate and 500 mg L-proline, intragastric nitrosation results in the formation of N-NP which is excreted in the urine. At-risk patients had previously undergone vagotomy with or without pyloroplasty ($n=14$) or partial gastrectomy ($n=12$), or had pernicious anaemia ($n=9$). The median N-NP levels were $2.9\,ng\,24\,h^{-1}$ (range 1.6–11.8 ng) in controls, $3.6\,ng\,24\,h^{-1}$ (0.6–11.2 ng) in vagotomy patients, $2.1\,ng\,24\,h^{-1}$ (0.2–14.5 ng) in partial gastrectomy patients and $3.8\,ng\,24\,h^{-1}$ (1.3–8.1 ng) in pernicious anaemia patients. There was no significant difference in N-NP excretion between any of the groups (Mann Whitney U test).

In this study we have not confirmed the theory that achlorhydria results in increased intragastric nitrosation with the production of potentially carcinogenic nitrosamines.

### Some preliminary evaluations of bacterial N-nitrosation reactions

S. Leach[1], A. Cook[1], A. Challis[2], M.J. Hill[1] & M. Thompson[1]

[1]*PHLS-CAMR, BMRL, Porton Down, Salisbury and* [2]*Department of Chemistry, Imperial College, South Kensington, London, UK.*

It is important to rigorously determine whether bacteria have any direct and significant role in endogenous N-nitrosation reactions. A direct bacterial catalysis of this reaction has been demonstrated unequivocally (refs. a, b, c in table) but the reported rates are low, questioning their clinical relevance. Much faster rates of reaction have been obtained more recently in our laboratory using certain aerobic denitrifying isolates of bacteria (Table). An assessment of the carcinogenic risk of endogenous N-nitrosation requires a knowledge of the important factors of influence for such reactions *in vivo*. It now seems likely that an important factor for endogenous N-nitrosation in chronic bacterial infection is the metabolic capability of the particular colonising organism(s) rather than the development of a flora *per se*.

| *Organism* | $[NO_2^-]$ *mM* | *[Morpholine]* *mM* | *Rate of nitrosation mmol nMOR* $mg^{-1}$ *protein* $h^{-1}$ | *Ref.* |
|---|---|---|---|---|
| *E. coli* A10* | 25 | 7.5 | 61 | a |
| *E. coli* A10* | 25 | 25 | 154 | |
| *E. coli* (range)* | 25 | 7.5 | 0–55 | b |
| *E. coli* (clin. isolate)* | 25 | 27 | 270 | c |
| *E. coli* (range)* | 25 | 8 | 0–76 | |
| *Pseudomonas aeruginosa** | 25 | 16 | 0–24,000 | |
| Other denitrifiers* | 25 | 16 | 0–3,200 | d |
| *P. aeruginosa* BM1030 grown in chemostat model | 25 | 16 | 1,300–15,000 | |

[a]K. Suzuki & T. Mitsuoka, *IARC Sci. Pub.*, **57**, 275, 1984; [b]Calmels *et al.*, *Carcinogenesis*, **6**, 911, 1985.; [c]Leach *et al.*, *Biochem. Soc. Trans.*, **13**, 381, 1985; [d]Present study. Organisms grown anaerobically $+NO_3^-/NO_2^-$.

*Assay conditions – washed cells c. 1 mg ml$^{-1}$ protein; pH7–7.5; incubation time, 30 min–2 h.

### Radiation response of human oesophageal cells *in vitro*

C. Mothersill[1], C. Seymour[1], P.J. Byrne[2] & T.P. Hennessy[2]

[1]*Saint Luke's Hospital, Rathgar, Dublin 6 and* [2]*TCD Department of Surgery, St. James's Hospital, Dublin 8, Eire.*

Accurate experimental information on the response of human tissue to chemotherapy or radiotherapy is of great importance. However, there are major problems in extrapolating from *in vivo* or *in vitro* results obtained on animal tissue. This is mainly because most models use rodents or undifferentiated established rodent cell lines which are difficult to correlate with results from human clinical trials.

Our group has been trying to establish an assay that enables us to look directly at the dose response of human oesophageal tissue to chemotherapy or radiation in terms of reduced growth rate. Our preliminary results have used radiation as the cytotoxic agent. To determine the growth rate pieces of tissue are plated as explants. The resulting area of cellular outgrowth is irradiated and then measured at weekly intervals. This enables us to monitor the effectiveness of treatment in terms of reduction of outgrowth from an explant. Preliminary results on 8 patients, involving measurements on 160 explants, show that the method can be used to determine the dose response to radiation and show a reduction of $66 \pm 15\%$ at 2.5 Gy, $80 \pm 5\%$ at 5 Gy and $91 \pm 8\%$ at 7.5 Gy. These values correlate very well with expected survival values for human cells after irradiation. The technique has been applied to colon explants with similar success.

It is intended to extend the method to allow the effectiveness of combination therapy to chemotherapy regimes to be assessed.

### Differential radiosensitivity between human bladder and testicular tumour cell lines

C.N. Parris[1], C.F. Arlett[2], S. Harcourt[2] & J.R.W. Masters[1]

[1]*Department of Histopathology, St. Paul's Hospital, Institute of Urology, 24 Endell Street, London WC2 9AE and* [2]*MRC Cell Mutation Unit, University of Sussex, Falmer, Brighton BN1 9OG, UK.*

Chemotherapy can cure advanced non-seminomatous testicular germ cell tumours, whereas advanced transitional cell carcinomas of the bladder generally are incurable. This differential sensitivity is retained *in vitro*, in that ~5 times as much *cis*-platin or adriamycin is required to produce the same cytotoxicity in bladder cancer compared with testicular cancer cell lines (Walker *et al.*, *Br. J. Cancer*, **52**, 459, 1985). To determine whether these cell lines differ in radiosensitivity, clonogenic cell survivals of five testicular and five bladder cell lines were compared following exposure to a $^{60}Co$ source at a range of cytotoxic doses. Colony-forming abilities of control and treated cells were compared after plating cells onto $10^5$ lethally irradiated homologous feeder cells. The testicular cell lines were all radiosensitive (D10s, doses reducing clonogenic cell survival by 90%, ranging from 2.2–3.4 Gy), similar to that of a fibroblast cell line (AT5BIVA: 2.2 Gy) derived from a patient with ataxia telangiectasia. In contrast, the bladder cell lines were all relatively radioresistant (D10s ranging from 4.0–5.8 Gy), similar to a fibroblast cell line (MRC5: D10, 4.7 Gy) derived from a clinically normal individual. The data indicate that testicular tumours, potentially curable using chemotherapy, have a radiosensitivity similar to that of cells derived from a patient with a disease associated with a DNA repair deficiency.

### Radiation response of small cell lung cancer lines

T.T. Kwok, P.R. Twentyman & N.M. Bleehen

*MRC Clinical Oncology and Radiotherapeutics Unit, MRC Centre, Hills Road, Cambridge, UK.*

Human small cell lung cancer (SCLC) is usually responsive to both radio- and chemotherapy upon initial presentation but becomes resistant to both forms of therapy in late stage disease. We have examined the radiation response characteristics of 11 lines of SCLC, 7 of them derived from patients undergoing treatment in our Unit. Six of the lines are from untreated patients whilst 5 are from patients with recurrent disease following multi-drug chemotherapy. Most of the lines grow as free floating aggregates of cells with varying degrees of 'tightness of aggregation' whilst the remaining line (COR-L88 – from a recurrent patient) grows as a monolayer attached to plastic. Radiation response was determined using a soft agar clonogenic assay both immediately or 24 h after irradiation of aggregates or plateau phase monolayers.

All of the lines except one were extremely responsive to radiation with small extrapolation numbers ($n = 0.68$–2.05) and steep slopes (Do = 0.65 Gy–1.34 Gy). Line COR-L88, however, although having a small extrapolation number ($n = 1.06$) had a much less steep slope (Do = 2.35 Gy). By growing this line on an agar base, it could be induced to grow as floating aggregates rather than a monolayer, but its radiation response parameters remained unchanged. None of the lines was able to exhibit significant recovery from potentially lethal radiation damage (as determined by comparison of cell survival determined at 0 h and 24 h after irradiation). Split dose experiments in the one line studied to date indicate that recovery from sublethal damage is also absent.

Studies of line COR-L88 aiming at providing an explanation of its relatively low radiosensitivity are currently in progress.

### Does verapamil enhance cytotoxic effects of anticancer drugs for lung tumours *in vitro*?

A.P. Simmonds[1], P. Moyes[1], A. Nicol[1], K.G. Davidson[2] & A. Faichney[2]

[1]*Cell Laboratory, Biochemistry Department and* [2]*Cardiothoracic Surgical Unit, Royal Infirmary, Glasgow, UK.*

Using a modification of REL medium successful in culure of NSCCL (Simmonds *et al.*, *Br. J. Cancer*, **52**, 429, 1985) we have investigated the influence of verapamil on the measured cytotoxic effects of *cis*-platinum and vindesine against squamous cell carcinoma of lung *in vitro*. Viable cells ($2 \times 10^5$) were exposed for 1 h to concentrations of drugs representing 10% peak plasma values with and without verapamil and to verapamil alone at 1 $\mu$M. Cells were washed and plated in enriched McCoys 5A in 0.3% agar over an underlayer of the same medium +1% rat rbc in 0.5% agar. Incubation was at 37° C in 5% $CO_2$/air and colony scoring of INT stained plates was done at 12 days. Colony counts in drug treated plates were expressed as percentage survival of control, <50% classed as sensitive. Results on 12 patients have indicated resistance to both drugs *in vitro*; percentage survivals ranged from 52–100% (15/24 tests >60% survival). The presence of verapamil had no effect on 5 patients' samples, with either drug. Pronounced changes were observed in 4 patients' response to vindesine; this changed to sensitive, the magnitude of the change ranging from 24–38% in percentage survival. For one of these patients, a 40% change in response to platinum was also demonstrated. Smaller changes in response to both drugs were recorded for the remaining patients and for platinum response in those with enhanced sensitivity to vindesine. It is clear from this study that 1 $\mu$M verapamil has sensitising effects, which may be pronounced, on some 58% of patients evaluated to date. The effect is more marked with vindesine. Verapamil alone against tumour cells has minimal effect. This study is continuing.

### The development of the VmDk murine astrocytoma as a therapeutic model of human glioma

R. Bradford, J.L. Darling & D.G.T. Thomas

*Department of Neurological Surgery, Institute of Neurology, Queen Square, London WC1N 3BG, UK.*

The choice of agents in current use for the chemotherapy of glioma has largely depended on results obtained from experimental models. Few, if any of these fulfil the criteria for an ideal therapeutic model for human glioma. Cell lines derived from a spontaneous astrocytoma originally arising in the inbred Vm strain of mice have been extensively characterised (Pilkington *et al., J. Neurol. Sci.,* **62,** 115) but are poorly tumourigenic in syngeneic mice. We have isolated a panel of 9 lines from one of these lines (P.497), 2 of which 497-P(1) and 497-C(1) produce subcutaneous and intracranial tumours with a uniform latency and predictable growth rate. One of these lines, 497-P(1), has been used to study the effects of procarbazine (PCB), vincristine (VCR), BCNU and CCNU *in vivo.* PCB, VCR, CCNU and BCNU produced specific growth delays of 0.7, 1.2, 1.7 and 3.7 in s.c. tumours respectively. Mice inoculated in the right cerebral hemisphere with $1 \times 10^6$ 497-P(1) cells die with a predictable mortality distribution (range 10–14 days). A course of chemotherapy with PCB ($100\,mg\,kg^{-1} \times 5$) had no significant effect on survival while VCR ($270\,\mu g\,kg^{-1} \times 5$), CCNU ($6\,mg\,kg^{-1} \times 5$) and BCNU ($6\,mg\,kg^{-1} \times 5$) produced 128, 128 and 140% increase in survival respectively. The similarity in response between VmDk murine astrocytomas and human glioma make this a valuable system for drug screening and studies of drug resistance.

### The relationship between cell biological characteristics and drug sensitivity in six clonal lines derived from a spontaneous murine astrocytoma

J.L. Darling[1,3], R. Bradford[1], H. Koppel[2], J. Martin[2], G.J. Pilkington[2], P.L. Lantos[2] & D.G.T. Thomas[1]

*Institutes of [1]Neurology, [2]Psychiatry and [3]Cancer Research, London, UK.*

Heterogeneity in drug sensitivity must, in part, account for the relative lack of success with single agent chemotherapy for malignant glioma. In order to examine this phenomenon *in vitro* we have derived 6 clonal cell lines from the P497 cell line originally isolated directly from the VmDk spontaneous murine astrocytoma. These clones have been extensively characterised using a variety of morphological, cell biological and immunocytochemical markers (Koppel *et al., J. Neurol. Sci.,* in press). In addition the drug sensitivity of these lines has been determined using a $^{35}S$-methionine uptake assay (Morgan *et al., Br. J. Cancer,* **47,** 205, 1983). The greatest differences in sensitivity between clones was observed for vincristine (VCR) and vindesine (VDS), where the $ID_{50}$'s varied between 13.7 and 20-fold respectively. Culture doubling times varied between 19.1 and 24.2 h but there was no correlation between drug sensitivity and either growth rate or saturation density. There was a relationship between cell morphology and sensitivity; lines predominantly composed of cells with few processes were more resistant to VCR, VDS, CCNU and adriamycin (ADR). Cells with large numbers of intermediate filaments and/or microtubules tended to be resistant to vinca alkaloids. Cultures comprised of cells with 60–70 chromosomes/cell were more resistant to BCNU, CCNU and *cis*-platinum than cells with more (>90) chromosomes. The presence of minute chromosomes in more than 50% of cells in a culture was related to resistance to ADR. There appeared to be no relationship between cytoskeletal marker expression and chemosensitivity. The relationship between phenotypic characteristics and chemosensitivity is complex, although we have demonstrated a clear relationship between genotypic changes and alkylating agent sensitivity. We are currently examining combinations of these clones in an attempt to determine the effects of cell-to-cell interaction on chemosensitivity and phenotypic expression and whether this will provide useful information for the development of new treatment strategies for malignant glioma.

### Therapeutic resistance in lung cancer subpopulations

G.A. Walls & P.R. Twentyman

*MRC Clinical Oncology and Radiotherapeutics Unit, MRC Centre, Hills Road, Cambridge CB2 2QH, UK.*

We have examined the possibility that the response to therapy of small cell (SCLC) and non-small cell (NSCLC) lung cancers may depend on the intrinsic properties of different tumour cell sub-populations. Two approaches were taken: (i) cell clones from one NSCLC and 3 SCLC cell lines were isolated using the clonogenic method of Courtenay & Mills

(*Br. J. Cancer*, **37**, 261, 1978). The responses of clonal sub-populations to X-rays (2 Gy) or adriamycin (ADM, 0.5 $\mu g\,ml^{-1}$ for 1 h) were then determined; (ii) Cells from the same lines were X-irradiated 4 times (3 × 4 Gy, 1 × 6 Gy) over 2 months. One SCLC cell line was exposed on 3 to 4 occasions to various doses of ADM, CCNU or vincristine. Response of these multi-treated cells to re-treatment with either X-rays or drugs was then determined. In all experiments, cells were in log phase growth at the time of treatment and cell kill was quantitated using clonogenic assay. Within a single experiment, clonal sub-populations of SCLC and NSCLC showed 2 to 4 fold differences in response to either X-rays or ADM but the relative response of different clones was not consistent between experiments. The response of multi-treated cells to further treatment was similar to that of the untreated population. However, in almost all instances, the plating efficiencies of the multi-treated cells were 1.5 to 2 fold higher than those of untreated cells. These results indicate that clonal sub-populations derived from cultured cell lines generally do not show major intrinsic differences in response to X-rays or ADM. Although multiple treatment with X-rays or drugs did not result in cell populations with increased resistance to further treatment, it did increase the proportion of clonogenic cells.

### The effect of 3-Acetamidobenzamide on the proliferation of normal and transformed cells

M.R. Purnell, J.M. Lunn & A.L. Harris

*Cancer Research Unit, Royal Victoria Infirmary, Newcastle upon Tyne NE1 4LP, UK.*

3-Acetamidobenzamide (AAB) is the most potent inhibitor of poly(ADP-ribose)synthetase known to date with a $k_i$ of 0.4 $\mu$M. We compared the effect of AAB on the proliferation of three human cell lines (WI38, embryonic lung fibroblasts; VA13, an SV40-transformed subline of WI38; A549, an epithelial line derived from a lung adenocarcinoma). A marked difference was observed between the $IC_{50}$ values over 4 days between WI38 cells and the transformed lines – 0.8 mM AAB for WI38 *versus* 3 mM (VA13) and 2.7 mM (A549). The difference was independent of growth rate because WI38 and VA13 had similar doubling times. Treatment of cells with cytostatic concentrations of AAB [3 mM (WI38) and 5 mM (VA13, A549)] for up to 24 h were reversible as judged by recovery of cell proliferation. Closer examination revealed that viable A549 cells had been blocked in $G_1$ phase because over 20 h were required following removal of the drug before the cell number increased over controls and the increase was blocked by the presence of 2 mM hydroxyurea. WI38 cells were also blocked in $G_1$ because inclusion of AAB in complete medium added to serum-starved $G_0$ cells prevented cells from entering S phase as judged by $^3$H-thymidine incorporation ($IC_{50}$ = 0.5 mM AAB). The block appears to be relatively late in $G_1$ because if 10 h were allowed to elapse between serum stimulation and AAB addition, the inhibition was the same. ADP-ribosylation reactions appear to be more active in WI38 cells because treatment with 3 mM AAB for 1 h causes a 60% increase in NAD levels compared to <20% increase in VA13 cells. In view of the ability of AAB to protect against the cytotoxicity of cell cycle-specific drugs, the differential sensitivity of normal and transformed cells to AAB will be discussed in relation to improving the therapeutic index of these drugs.

### $N^{10}$-propargyl-5,8-dideazafolic acid polyglutamates: Synthesis, biochemical properties and formation *in vitro*

E. Sikora, D.R. Newell, A.L. Jackman, K. Pawelczak, T.R. Jones & A.H. Calvert

*Drug Development Section, Institute for Cancer Research, Sutton, Surrey, UK and Chemical Institute, Opole, Poland.*

$N^{10}$-propargyl-5,8-dideazafolic acid (CB3717), a tight binding inhibitor of thymidylate synthase (TS), is a novel antifolate currently undergoing clinical evaluation. Intracellular polyglutamation is a known metabolic pathway for both natural folates and antifolates, which results in increased cellular retention and greater affinity for certain folate-dependent enzymes. We have, therefore, investigated the formation and biological properties of CB3717 di- and triglutamate. CB3717 polyglutamates were synthesised from the tert-butyl esters of p-aminobenzoic acid di- and triglutamate, characterised by NMR and analysed by reverse phase HPLC ($\mu$Bondapak C18 column, 5–16% $CH_3CN$ (10 min) in 0.1 M $NaCH_3COO$ pH5). Against L1210 TS CB3717 di- and triglutamate were 30- and 84-fold more potent inhibitors than the parent compound, respectively. Ki apparent: CB3717 26 ± 3 nM, CB3717 diglutamate 0.89 ± 0.05 nM and CB3717 triglutamate 0.32 ± 0.02 nM. Against L1210 cells, cultured in the presence of heat-inactivated serum, CB3717 and CB3717 diglutamate were equipotent ($IC_{50}$ 48 h exposure 3 $\mu$M). However, HPLC

analyses indicated that there was no degradation of CB3717 diglutamate to CB3717 within 48 h. Following a 24 h exposure of L1210 cells to 25 μM $^3$H-CB3717, 85–95% of the cellular $^3$H could be extracted by boiling in 0.1 M Tris-HCl pH10. This extract was shown, by HPLC, to contain CB3717 and CB3717 di- and triglutamate. These studies indicate that CB3717 can be polyglutamated by L1210 cells *in vitro*, the products being markedly more potent than CB3717 as TS inhibitors. However, against whole cells CB3717 diglutamate was no more potent than the parent compound.

**Comparative studies of DNA cross-linking reactions following methylene dimethane sulphonate and its hydrolytic product formaldehyde**

P.M. O'Connor & B.W. Fox

*Paterson Laboratories, Christie Hospital & Holt Radium Institute, Manchester M20 9BX, UK.*

Methylene dimethane sulphonate (MDMS) possesses marked antitumour activity against the rodent Yoshida lymphosarcoma cell line (YS) and is currently undergoing Phase I trials at Christie Hospital, Manchester. MDMS is rapidly hydrolysed ($t\frac{1}{2}$ = 22 min., 37°C) to release Formaldehyde (HCHO). Since HCHO can itself be cytotoxic, a study of the contribution of HCHO to MDMS induced cytotoxicity was undertaken.

The technique of alkaline elution was employed to study the interaction of MDMS and HCHO with DNA from YS cells. MDMS and HCHO produced a proteinase K sensitive filter retention which indicated the presence of DNA-protein cross-links. MDMS also produced some proteinase K resistant filter retention which was believed to indicate DNA-interstrand cross-linking. Some single strand breaks were also detected in the presence of HCHO. Co-incubation with semi-carbazide prevented all DNA-protein cross-links and single strand breaks induced by MDMS and HCHO, while tending to increase the number of DNA-interstrand cross-links from MDMS. Under these conditions no DNA-interstrand cross-linking can be seen following HCHO treatment.

Semicarbazide reduced HCHO induced cytotoxicity in the YS cell line while no significant alteration in MDMS induced cytotoxicity was observed. These results suggest that HCHO induced DNA-protein cross-links and single strand breaks do not contribute to MDMS induced cytotoxicity. Therefore, MDMS induced DNA-interstrand cross-links is the likely cytotoxic lesion of this agent.

Gel electrophoretic analysis of HCHO and MDMS induced DNA-protein cross-links showed that some proteins cross-linked to DNA by MDMS differed from those cross-linked by HCHO.

**Mechanism for resistance to cyclophosphamide and its metabolites**

A. McGown & B.W. Fox

*Paterson Laboratories, Christie Hospital, Withington, Manchester M20 9BX, UK.*

A Yoshida sarcoma tumour (YR/cyclo) showing decreased sensitivity to cyclophosphamide has been developed from the parental (Ys) tumour by incremental challenge with the drug *in vivo*. Resistance to metabolically activated cyclophosphamide and phosphoramide mustard has been shown to be retained when cell lines derived for these tumours are grown in culture. The $ID_{50}$ concentrations for the YR/cyclo and Ys cell lines *in vitro*, are for activated cyclophosphamide 36.4 μM, and 1.9 μM, and for phosphoramide mustard 47 μM, and 2.1 μM respectively. These values show a similar level of resistance (~20-fold) for both the parent drug, and one of its ultimate metabolites.

Resistance is shown to be associated with a 6-fold increase in activity of the glutathione S-transferase group of enzymes, as determined by use of 1-chloro,-2,4-dinitrobenzene as a substrate. An increase in glutathione (~2-fold), the co-substrate for the enzyme, is also seen.

The YR/cyclo cell line is also shown to exhibit a decreased level of cellular damage, as measured by alkaline elution, following treatment with phosphoramide mustard, compared with the parental line.

The mechanism of resistance in the YR/cyclo cell line is proposed to be increased cellular deactivation of potentially damaging metabolites of cyclophosphamide by the glutathione S-transferase enzymes, resulting in decreased cellular damage in resistant cells.

**Proteins associated with multidrug resistance in human lung cancer cell lines**

J.G. Reeve & P.R. Twentyman

*MRC Clinical Oncology and Radiotherapeutics Unit, MRC Centre, Hills Road, Cambridge CB2 2QH, UK.*

Multidrug resistance (MDR) is often associated with reduced cellular accumulation of the drugs involved and with changes in plasma membrane protein composition. Of particular note is the expression of a 170,000 dalton plasma membrane glycoprotein which is invariably associated with this pleiotropic phenotype. A number of multidrug resistant variants of lung cancer cell lines have recently been derived *in vitro* in our laboratories. Membrane and cytoplasmic proteins of these variants together with their sensitive counterparts are currently being investigated using SDS-PAGE. To date, such studies reveal that MDR in LX4, a multidrug resistant variant of the small cell lung cancer cell line NCI-H69, is associated with loss of a 90 Kd membrane protein, hyperexpression of a 100 Kd membrane protein and with the acquisition of a 22 Kd cytosolic protein. A rabbit antiserum has been raised against LX4 cells and following extensive absorption with NCI-H69 reacts strongly with LX4 in solid phase RIA. This antiserum immunoblotted a number of membrane proteins which are apparently hyperexpressed in LX4 but not in NCI-H69. The reactivity of this antiserum with multidrug resistant variants of human non-small lung cancer cell lines and with murine multidrug resistant cell lines is currently being investigated with a view to identifying common molecular mechanisms of drug resistance.

### Elevation of glutathione and glutathione-dependent enzymes during cell division in ovarian and breast cancer cell lines

A.D. Lewis & C.R. Wolf

*Imperial Cancer Research Fund, Laboratory of Molecular Pharmacology, Hugh Robson Building, George Square, Edinburgh, UK.*

Glutathione (GSH) and glutathione-dependent enzymes play a central role in protecting tumour cells from the effects of cytotoxic drugs. Previously we have obtained evidence that these enzymes may be regulated in normal cells during mitosis. We are interested in the regulation of glutathione and dependent enzymes in tumour cells in relation to drug resistance and have investigated these factors during mitosis. Three tumour cell lines, Ovarian, PE01 and PE04, and breast cancer, MCF7 were used and GSH, glutathione transferase (GST), γ-glutamyl-cysteinyl synthetase (GCS), γ-glutamyl transpeptidase (GGT), glutathione peroxidase (GP) and glutathione reductase (GR) determined in rapidly dividing log phase and confluent cultures. A highly significant 2- to 3-fold elevation of GSH was measured in log cultures of all three cell lines. In cells synchronised by serum depletion up to 4-fold elevations were observed. In parallel with these changes a significant elevation in GCS (~2-fold) GST (2- to 3-fold) and GP (~2-fold) were measured. The increase in GCS, the rate limiting enzyme in glutathione synthesis, will account for the increased GSH levels. GGT and GR levels were not changed in the log cultures. We are currently evaluating the importance of these observations in cell division and drug sensitivity.

### Effects of glutathione depletion on the toxicity of cytotoxic agents to tumour cells

M.' d'Arcy Doherty, J. Jordan & G.M. Cohen

*Toxicology Unit, The School of Pharmacy, University of London, 29/39 Brunswick Square, London WC1N 1AX, UK.*

Glutathione (GSH) plays a critical role in cellular defences against alkylating agents, oxidative stress and free radicals. We have used buthionine sulfoximine (BSO), a specific inhibitor of GSH synthesis, to deplete GSH in human colonic tumour cells in culture. Following an overnight incubation with BSO (0.1–0.2 mM), cellular GSH was depleted to <20% of the control value. The effect of GSH depletion on the toxicities of some model compounds including alkylating agents (melphalan and helenalin), a compound which redox cycles (menadione) and a peroxide ($H_2O_2$) was investigated. Pretreatment with BSO had the greatest effect on the toxicity of helenalin resulting in a 4-fold decrease in $IC_{50}$ as measured by an inhibition of protein synthesis. BSO caused less than a 2-fold decrease in $IC_{50}$ values for the other compounds. As the degree of potentiation was not great and was only observed over a limited dose range, the relevance of these findings to the proposed use of BSO in the clinic as an agent to enhance the effectiveness of some antitumour agents will be discussed.

### The influence of pH on drug cytotoxicity *in vitro*

E. Groos, L. Walker, & J.R.W. Masters

*Department of Histopathology, Institute of Urology, St. Paul's Hospital, 24 Endell Street, London WC2H 9AE, UK.*

Intravesical chemotherapy is used in the management of superficial bladder cancer, but optimum conditions for its administration have not been defined. In this study we investigated the influence of pH on the cytotoxicities *in vitro* of the four most frequently used drugs: adriamycin, epodyl, mitomycin-c and thiotepa, in addition to *cis*-platin and epirubicin. The colony-forming ability of RT112 (a continuous cell line derived from a transitional cell carcinoma of the human bladder) was measured following a 1 h exposure to each drug at eleven pH values ranging from 5.2 to 9.7. Mitomycin-c, *cis*-platin and thiotepa showed increasing cytotoxicity as the pH became more acidic. For example, reproductive cell survival fell from 91.3% to 16.2% following exposure to 8$\mu$g ml$^{-1}$ thiotepa when the pH was reduced from 8.0 to 6.1. In contrast with adriamycin and epirubicin cytotoxicity was greatest in alkaline media. Epodyl showed similar activity throughout the pH range. It is concluded that the therapeutic value of intravesical chemotherapy might be enhanced by instilling the drugs in solutions buffered at the optimum pH for each agent.

### The influence of osmolality on drug cytotoxicity *in vitro*

E. Groos, L. Walker & J.R.W. Masters

*Department of Histopathology, St. Paul's Hospital, 24 Endell Street, London WC2H 9AE, UK.*

The osmotic strength of the instillate is one of the factors that could influence the effectiveness of intravesical chemotherapy for superficial bladder cancer. In this study we investigated the effect of osmolality on the cytotoxicities of the four drugs most frequently used: adriamycin, epodyl, mitomycin-c and thiotepa, in addition to *cis*-platin and epirubicin. The colony-forming ability of RT112 (a continuous cell line derived from a transitional cell carcinoma of the human bladder) was measured following a 1 h exposure to each drug in media of six osmotic strengths ranging from 125–590 mOsm kg$^{-1}$ $H_2O$. Osmotic strength was modified by adding either distilled water or 50% dextrose to tissue culture medium. Osmolality had little effect on the cytotoxicities of adriamycin and epodyl, but the other drugs killed more cells in hypo-osmotic media. For example, reducing the osmolality from 290 to 200 mOsm kg$^{-1}$ $H_2O$ increased the clonogenic cell kill of 2.5 $\mu$g ml$^{-1}$ of *cis*-platin from 20% to 99%. Urine from bladder cancer patients before treatment had osmolalities ranging from 187–852 mOsm kg$^{-1}$ $H_2O$, and these had decreased on average by 135 mOsm kg$^{-1}$ $H_2O$ at the completion of intravesical chemotherapy. The osmotic strengths of solutions of clinical preparations of the drugs at a concentration of 1 mg ml$^{-1}$ were (a) in distilled water, 63–1,015 mOsm kg$^{-1}$ $dH_2O$ and (b) in 0.9% saline, 301–1,038 mOsm kg$^{-1}$ $dH_2O$. It is concluded that the therapeutic value of intravesical chemotherapy might be enhanced by reducing the osmotic strength of the instillate.

### The reversal of the cytotoxicity of folate-based thymidylate synthase inhibitors in cultured L1210 cells

A.L. Jackman[1], L. Cameron[1], R. Moran[2] & A.H. Calvert

[1]*Drug Development Section, Institute for Cancer Research, Sutton, Surrey, UK and* [2]*Children's Hospital of Los Angeles, USA.*

Some analogues of folic acid have been shown to be inhibitors both of isolated thymidylate synthase (TS) and of dihydrofolate reductase (DHFR), enzymes linked in the oxidation-reduction cycle of tetrahydrofolic acid ($FH_4$). Mathematical modelling demonstrated that a dual inhibitor should act effectively at one locus, which would be TS until Ki TS/Ki DHFR approaches 3,000 (Jackson *et al.*, *Biochem. Pharmacol.*, **32,** 3783, 1983). One compound that clearly acts by inhibition of TS is $N^{10}$-propargyl-5,8-dideazafolic acid (CB3717: Ki TS 4 nM; $IC_{50}$ in culture = 5 $\mu$M). In L1210 tissue culture (48 h exposure), thymidine (dThd) reversed the cytotoxic effects of CB3717 and this reversal was dose-independent. However, if exposure to both drug and dThd was for 72 h a marked CB3717 dose-dependent reversal was observed such that any concentration above 50 $\mu$M was poorly reversed by dThd (0.1 mM and 1 mM CB3717 gave 50% and 30% of control cell growth respectively). This dose-dependent dThd reversal was even more apparent with compounds having a greater Ki TS/DHFR ratio. Cytotoxicity at 72 h was readily reversed by the addition of either a purine or folinic acid, together with dThd. A DHFR overproducing cell line (L1210:R7A) was insensitive to 1 mM CB3717 in the presence of dThd even at 72 h. In tissue culture, dividing cells are dependent on the uptake and reduction of folic acid from the medium in order to maintain the level of intracellular reduced folates. We suggest that the weaker inhibitory effects of these compounds on DHFR may become important in the presence of dThd, by preventing this reduction. When DHFR is overproduced the cells become insensitive to the DHFR effects, a purineless state is not induced and consequently dThd alone is an effective rescue agent.

**Organ-culture of colonic tissues as a model for investigating the mechanism of anti-metabolite action**

M. Moorghen[1], P. Ince[1], K.J. Finney[1], A.L. Harris[2] & A.J. Watson[1]

*Departments of [1]Pathology and [2]Clinical Oncology, University of Newcastle upon Tyne, Newcastle upon Tyne, UK.*

Organ-culture is a means of studying *in vitro* cell proliferation in human and rodent colonic tissues. We have used the technique to investigate the biochemical effects of anti-metabolite drugs on intact epithelium.

Explants of rat colonic mucosa were incubated with [methyl-$^3$H] thymidine (*TdR) together with other drugs at varying concentrations. After a 4 h incubation period, the explants were washed. They were then dissolved by overnight incubation at room temperature in 0.2 M NaOH, and the uptake of *TdR measured by liquid scintillation counting. Total DNA was also measured by the DAPI fluorometric method.

1 mM of Hydroxyurea caused maximum inhibition of *TdR uptake. This is similar to observations made in cell lines. 5-Fluorouracil (5 FU) and 5-Fluoro-2-deoxyuridine (5 FUDR) failed to show an effect on *TdR uptake in this system, despite the finding in cell-lines of an increased uptake. This absence of effect may be related to an RNA rather than a DNA-directed effect.

The metabolic effects demonstrated by this method, as compared to methods involving cell lines, are likely to reflect more closely the *in vivo* action of this class of drugs.

We are now studying 5 FU and 5 FUDR together with biochemical modulators in normal colonic mucosa and carcinoma tissues from humans and rats.

**12-0-Tetradecanoylphorbol-13-acetate (TPA) and the antitumour drug adriamycin (ADR) modulate morphological transitions of human erythrocytes (RBC)**

M.G. Thompson, S.B. Chahwala, T.F. Walker, B. Jones & J.A. Hickman

*CRC Experimental Chemotherapy Group, Aston University, Birmingham B4 7ET, UK.*

The effects of the tumour promoter TPA on cell morphology resemble those brought about by the transforming tumour viruses, and are, at least in part, the consequence of direct effects upon the cell membrane/cytoskeleton (Dreidger *et al., Cancer Res.*, **37**, 3257, 1977). Recently, attention was drawn to the similarities in the effects of TPA and the antitumour drug adriamycin on membrane structure and function (Zuckier & Tritton, *Exp. Cell. Res.*, **148**, 155, 1983). We have reported that ADR modulates the morphological transition of RBC from discocyte to echinocytes (*Cancer Res.*, **45**, 4986, 1985). When $10^7$ RBC ml$^{-1}$ were incubated under conditions of ATP depletion at 37° in an isotonic Tris-salt buffer, $10^{-7}$ M TPA delayed the formation of echinocytes: after 2 h controls contained 65% echinocytes, TPA-treated cells 23%. Echinocytosis was complete by 3 h in controls and 5 h in TPA-treated cells. Preincubation for 10 min of $10^7$ RBC ml$^{-1}$ with TPA (200 nM) inhibited by 50% the echinocytosis induced by 0.2 mM $Ca^{2+}$ and 5 $\mu$M A23187. TPA and ADR had no effect *per se* on discocyte morphology at concentrations of up to 100 $\mu$M, and are therefore unlike the amphipathic drugs, such as phenothiazines, which modulate morphological change but are themselves able to alter morphology to stomatocytes. The distinctive effects of ADR and TPA on RBC morphological change suggest that they may be acting *via* a common biochemical mechanism.

**Involvement of $Ca^{2+}$ in anthracycline resistance**

A. McGown & B.W. Fox

*Paterson Laboratories, Christie Hospital & Holt Radium Institute, Withington, Manchester M20 9BX, UK.*

Resistance to daunorubicin has been shown to be associated with a 3 fold decreased drug accumulation in resistant P388R8/13 cells as compared with the parental cell lines. The technique of flow cytometry has been used to directly quantitate daunorubicin levels in individual cells by utilising the intrinsic fluorescence of the drug. The agents verapamil, perhexiline maleate, flunarizine, cinnarizine, nicardipine, nifedipine, lidoflazine, and diltiazem are known to alter calcium transport in certain tissues. These are shown to be able to increase intracellular daunorubicin in resistant P388 cell lines, to the level observed in the parental line. No effect on daunorubicin accumulation is seen in the parental cell line. The effects of these agents on $Ca^{2+}$ influx into the parental and resistant cell lines have been studied by using $^{45}Ca^{2+}$. None of these agents show any effect on $^{45}Ca^{2+}$ influx in either the resistant or parental cell lines at concentrations which alter anthracycline accumulation. In addition daunorubicin itself does not alter $^{45}Ca^{2+}$ flux in these cell lines. The calcium ionophore A23187 which increases intracellular $^{45}Ca^{2+}$, is also shown to elevate daunorubicin levels.

Since anthracycline accumulation in resistant cells is not directly associated with inhibition of calcium transport, it is concluded that it may be possible to design agents capable of altering anthracycline sensitivity but without the toxicity associated with inhibition of calcium transport.

### Effects in spheroids of anthracyclines of different lipophilicity

T.T. Kwok & P.R. Twentyman

*MRC Clinical Oncology and Radiotherapeutics Unit, MRC Centre, Hills Road, Cambridge, UK.*

Adriamycin (ADM) is a widely used drug in the therapy of solid tumours. It has, however, been found to penetrate poorly into cellular aggregates and to be relatively ineffective against plateau phase cells *in vitro*. Using a selective disaggregation method, we have previously shown (Kwok & Twentyman, *Int. J. Cancer*, **35**, 675, 1985) that a short exposure to ADM kills relatively few cells in the innermost regions of large ($\sim 800\,\mu$ diameter) multicellular spheroids of the EMT6/Ca/VJAC mouse tumour cell line. One property of ADM which may be involved in its poor penetrating ability is its low lipophilicity (octanol/water partition coefficient (PC) = 0.46). We have therefore examined the differential cytotoxicity within spheroids of a number of anthracycline analogues produced by Roche Products Ltd and differing widely in PC. The compounds studied are Ro 31-1741 (PC = 13), Ro 31-1215 (PC = 28) and Ro 31-2035 (PC > 100). All 3 novel anthracyclines showed much greater cytotoxicity towards cells in the outer compared with the inner region of EMT6 spheroids at the dose levels studied. Direct comparison of relative cell killing is complicated by the different potencies of the drugs studied. However, the slope of the cytotoxicity curve against spheroid depth is less steep for Ro 31-2035 than for the other agents (including ADM). Fluorescence measurement of the cellular drug content within different cell fractions isolated from spheroids indicate that, for a given external medium concentration, the accumulation of Ro 31-2035 is 3–10× that of the other drugs. A considerable differential in drug content between outer and inner cells is, however, seen for all 3 agents. Studies on log and plateau phase monolayer cells (in progress) will enable a more detailed analysis of the spheroid data to be carried out.

### The effect of the surfactant Brij 30 on cellular uptake and cytotoxicity of adriamycin (ADR) in lung tumour monolayers and spheroids

D.J. Kerr[1], T.E. Wheldon[2], J. Russell[2], A.T. Florence[3] & S.B. Kaye[1]

[1]*Department of Medical Oncology and*
[2]*Radiobiology Group, Glasgow University and*
[3]*Department of Pharmacy, Strathclyde University, Glasgow, UK.*

Resistance of intact spheroid cells to adriamycin treatment has been reported and the existence of drug penetration barriers has been postulated. We have studied the effects of a polyoxethylated surfactant (Brij 30) on monolayer uptake and spheroid penetration of adriamycin. Human lung non-small cell tumour monolayers (L-DAN line) were exposed for varying times to ADR (±Brij 30, 0.001% solution) over a wide concentration range. Intracellular drug levels were measured, after organic extraction, by a sensitive HPLC assay. The cytotoxicity of ADR (±Brij 30) was tested in a standard clonogenic assay after exposure of monolayers and intact spheroids to drug for 1 hour, with subsequent disaggregation to a single cell suspension. Spheroids were exposed to ADR (±Brij 30) for 1 h, and then transferred to multiwell petri dishes where consecutive measurements of diameter were made using an image analysis system. Co-incubation with Brij 30 increases intracellular ADR levels by 2–3 fold. ADR + Brij is significantly more cytotoxic (monolayer $ID_{90}$ $0.6\,\mu g\,ml^{-1}$; disaggregated spheroid $ID_{50}$ $1.9\,\mu g\,ml^{-1}$) than ADR alone (monolayer $ID_{90}$ $2.1\,\mu g\,ml^{-1}$; disaggregated spheroid $ID_{50}$ $3.3\,\mu g\,ml^{-1}$). In addition, fluorescence microscopy has shown that Brij 30 increased the intraspheroidal depth to which ADR diffused. Conclusion: Brij 30 enhances monolayer cytotoxicity of ADR and may overcome spheroidal penetration barriers with an improved cytotoxic effect.

### Comparison of *cis*-platin-induced DNA damage in human bladder and testicular tumour cell lines

P. Bedford, J.R.W. Masters & B.T. Hill

*Imperial Cancer Research Fund Laboratories, Lincoln's Inn Fields, London WC2A 3PX and Institute of Urology, Endell Street, London WC2 9AE, UK.*

*Cis*-platin is an effective chemotherapeutic agent in the treatment of bladder and testicular tumours

(Prestayko *et al.*, *Cancer Treat. Rev.*, **6**, 17, 1979). Since DNA is an important target for this drug, we compared DNA damage and repair following *cis*-platin exposure in human tumour continuous cell lines derived from two transitional cell carcinomas of the bladder T24 and RT112) and one testicular germ cell tumour (SUSA). Concentrations of *cis*-platin reducing *in situ* colony formation to 10% of the control after a 1 h exposure were 3.1, 7.6 and 14.6 $\mu g\,ml^{-1}$ for SUSA, T24 and RT112 cells respectively. DNA-DNA interstrand crosslinks and DNA single-strand breaks were measured at 0, 5, 14 and 24 h after a one hour exposure to a range of *cis*-platin concentrations (2–20 $\mu g\,ml^{-1}$) using the technique of alkaline elution (Kohn *et al.*, In *DNA Repair: A Laboratory Manual of Research Procedures., Vol.* **1.** Part B. (eds.) Friedberg *et al.*, Marcel Dekker, NY, p. 379, 1981). Maximum numbers of crosslinks were detected at 5 h for T24 cells and at 14 h for RT112 cells with a subsequent decrease at 24 h. In the SUSA cell line crosslinking continued to increase up to 24 h. Single-strand breaks were detected only in the SUSA cells. Peak numbers of crosslinks in SUSA, T24 and RT112 cells were 28.8, 97.7 and 54.6 rad-equivalents respectively after exposure to 10 $\mu g\,ml^{-1}$ *cis*-platin. Further studies are in progress to determine if these differences in total numbers of crosslinks are a consequence of differential drug penetration or binding to DNA.

### *In vitro* drug resistance; interactions between X-irradiation and etoposide

R.B. Lock & B.T. Hill

*Laboratory of Cellular Chemotherapy, Imperial Cancer Research Fund, Lincoln's Inn Fields, London WC2A 3PX, UK.*

A human tumour continuous cell line (HN-1) has been made resistant to etoposide (VP-16-213) by either exposure to fractionated X-irradiation or continuous exposure to VP-16-213 (Lock & Hill, *Br. J. Cancer*, **52**, 425, 1985). Drug responses were established by cell survival assays using soft agar (Courtenay & Mills, *Br. J. Cancer*, **38**, 77, 1978). $IC_{50}$ values for the parent (HN-1), X-irradiation treated (HN-1/DXR-5) and drug treated (HN-1/VP-2) cells were 3.1, 5.0 and 9.7 $\mu$M respectively following a 1 h exposure to VP-16-213. These differential responses to etoposide could not be accounted for in terms of altered accumulation of VP-16-213, since drug uptake studies revealed no significant differences between the three lines: exposure to 3.1 $\mu$M [$^3$H]-etoposide for 30 min resulted in $18.3 \pm 1.9$, $20.1 \pm 0.5$ and $19.1 \pm 4.6$ $pmol\,mg^{-1}$ protein associated with the HN-1, HN-1/DXR-5 and HN-1/VP-2 cells respectively. Although altered levels of non-protein sulphydryl compounds have been implicated in resistance mechanisms, no significant differences were detectable in logarithmically growing cells from the 3 lines (Lock & Hill, 1985) and glutathione S-transferase activity was also comparable in the parental and drug treated lines with activities of $249 \pm 24$ and $234 \pm 17$ $nmol\,mg^{-1}\,min^{-1}$ respectively. However, alkaline elution studies have revealed a reduction in total DNA breakage and estimated single-strand breakage (SSB) in the drug treated subline (HN-1/VP-2) compared to the parental line after exposure to equimolar concentration of VP-16-213. Rad-equivalent values for total DNA breakage are $346 \pm 35$ and $461 \pm 11$, and for SSB are $625 \pm 131$ and $1,252 \pm 309$ for HN-1/VP-2 and HN-1 cells respectively (8.5 $\mu$M VP-16-213, 1 h). These data suggest that resistance to VP-16-213 in these cells may be associated with reduced activity of DNA topoisomerase II.

### Drug resistance in human non-small cell lung cancer cell lines – the role of membrane transport

S. Merry, E.R. Courtney, S.B. Kaye & R.I. Freshney

*Department of Medical Oncology, University of Glasgow, 1 Horselethill Road, Glasgow G12 9LX, UK.*

We have shown (Fetherston *et al.*, *Br. J. Cancer*, **51**, 598, 1985) that 6.6 $\mu$M verapamil (VPM) increased the sensitivity (up to 29-fold) of human non-small cell lung cancer cell lines to adriamycin (ADR), vincristine (VC) and etoposide (VP16). None of these cell lines had been exposed to cytotoxic drugs *in vitro*. Here we report the effects of 6.6 $\mu$M VPM on ADR, VC and VP16 accumulation in 2 resistance cell lines (A549 and SK-MES-1). Exponentially growing cells ($10^5$ $cells\,cm^{-2}$) were seeded into 10 mm diameter soda glass specimen tubes and, after 3 days, radio-labelled drug was added to replicate tubes for 0, 30, 60 or 90 min in the presence or absence of VPM. Drug accumulation was then determined by scintillation counting of the washed, solubilised cell monolayer. For VC increases in accumulation of up to 4-fold were seen, with the greatest increase seen in the cell line SK-MES-1 in which the greatest effect of VPM on cytotoxicity was noted. For ADR and VP16 smaller increases in drug levels (up to 1.6-fold) were seen. This suggests that enhancement by VPM of

ADR and VP16 activity in these cell lines may operate through mechanisms additional to its effect on intracellular drug levels. Another cell line (WIL) has been established as a subcutaneous xenograft tumour line in nude mice. In this model 40 $\mu g\, g^{-1}$ VP16 i.p. together with 25 $\mu g\, g^{-1}$ VPM i.p. was shown to produce a significantly longer ($P = 0.01$) tumour volume doubling time compared to 40 $\mu g\, g^{-1}$ VP16 alone. Studies of the effect of VPM on VP16 levels in WIL xenografts are in progress. Furthermore a randomized clinical trial assessing the role of verapamil in the treatment of lung cancer is also in progress.

### Resistance to *cis*-platinum and patterns of cross-resistance in two autologous human ovarian adenocarcinoma cell lines

I.P. Hayward, S.S. Lawrie, A.R.R. Laudonio, A. McDonald & J.F. Smyth

*Imperial Cancer Research Fund Medical Oncology Unit, Western General Hospital, Edinburgh, UK.*

Two human ovarian carcinoma cell lines have been established from ascites samples from the same patient before (PE01) and after (PE04) the onset of clinical resistance to a combination of 5-fluorouracil, chlorambucil and *cis*-platinum. Their sensitivity to various drugs *in vitro* (72 h exposure) was investigated by a clonogenic assay on plastic. PE04 shows a 3× increase in resistance to *cis*-platinum ($ID_{50}$ 0.20 $\mu$M for PE04 *vs.* 0.064 $\mu$M for PE01) and chlorambucil ($ID_{50}$ 3.2 $\mu$M *vs.* 1.0 $\mu$M) but not to 5-fluorouracil ($ID_{50}$ 5.2 $\mu$M *vs.* 4.8 $\mu$M). PE04 showed various levels of increased resistance compared with PE01 to *cis*-platinum analogues with 7× difference to malonato platinum (JM40), 2.7× difference to carboplatin (CBDCA) and no difference in sensitivity to isopropylamine platinum (CHIP). Small increases in resistance between 1.5–2 fold were observed towards adriamycin, vincristine and mitozantrone. Another alkylating agent, melphalan, showed a 3-fold increase in resistance for PE04. Possible mechanisms for the increased resistance observed in PE04 against *cis*-platinum have been investigated. PE04 has increased levels of glutathione and glutathione-S-transferases (Wolf *et al., Br. J. Cancer,* **50,** 276, 1984). However incubation with minimally toxic doses of buthionine-S-sulfoximine (100 $\mu$M for 24 h), a glutathione synthesis inhibitor, failed to significantly increase *cis*-platinum sensitivity. Preliminary experiments show no difference in *cis*-platinum uptake into the resistant cells as measured by atomic absorption spectroscopy but using the alkaline elution technique there is a 2-fold difference between the cell lines in *cis*-platinum-induced DNA cross-linking.

### Apparent lack of repair of carboxymethylated DNA by alkyltransferase proteins

D.E.G. Shuker[1], J. Brennand[2] & G.P. Margison[2]

[1]*MRC Toxicology Unit, Woodmansterne Road, Carshalton, Surrey SM5 4EF and* [2]*Paterson Laboratories, Christie Hospital, Manchester M20 9BX, UK.*

N-nitrosoglycocholic acid (NOGC) is a mutagenic and carcinogenic derivative of the naturally occurring bile acid conjugate, glycocholic acid. NOGC reacts with calf thymus DNA *in vitro* to give a number of carboxymethyl adducts of which N-7-carboxymethylguanine and N-3-carboxymethyladenine have been identified (Shuker & Tannenbaum, *Br. J. Cancer,* **52,** 443, 1985).

Using extracts of *E. coli* harbouring a plasmid containing a gene coding for $O^6$-AG alkyltransferase (AT; Margison *et al., Nucl. Acid Res.,* **13,** 1939, 1985) in an *in vitro* competition assay we found that NOGC-treated calf thymus DNA inhibited the action of the AT on methylated DNA. This suggested that repairable carboxymethyl adducts were present, however, we were unable to detect any transfer of ($^{14}C$)-carboxymethyl groups from ($^{14}C$)-NOGC treated DNA to the repair protein as has been observed for other alkyl groups. Moreover, the profile of adducts in DNA hydrolysates did not change following treatment of the DNA with the AT-containing extracts.

We have now established that $O^6$-carboxymethyl guanine ($O^6$-CMG) is present in NOGC treated DNA and that it is not removed by *E. coli* AT. Human fibroblast AT also appeared to be ineffective in removing $O^6$-CMG.

We are currently attempting to establish whether our results may be due to extremely low rates of AT action on $O^6$-CMG.

### Potentiation of the cytotoxic action of DTIC: Involvement of O[6]-methylguanine-DNA methyltransferase

J.M. Lunn[1], A.L. Harris[1], P.M. Brown[1],

C. Pierpoint [2] & B.T. Golding[2]

[1]*Cancer Research Unit and* [2]*Department of Organic Chemistry, University of Newcastle upon Tyne, Newcastle upon Tyne NE1 4LP, UK.*

The cytotoxicity of MTIC, the active metabolite of the antitumour drug 5-(3,3-dimethyl-1-triazeno)-imidazole-4-carboxamide (DTIC), was assessed by measuring proliferation following exposure of cultured cells to MTIC.

Sensitivity of cells to MTIC correlated with their Mer/Rem phenotypes. Sensitivity increased in the order HT29 ($Mer^+Rem^+$) <A549 ($Mer^+Rem^-$) <VA13 ($Mer^-Rem^-$), indicating involvement of the O[6]-methylguanine lesion in cytotoxicity. Further, O[6]-methylguanine has been detected by HPLC in DNA treated *in vitro* with MTIC. Pre-treatment of cells with non-toxic levels of MTIC rendered them ore sensitive to subsequent exposure to chloro-nitrosourea (CNU).

MTIC cytotoxicity was potentiated by 3-acetamidobenzamide (3AAB), an inhibitor of adenosine diphosphoribosyl transferase (ADPRT). The enhancement was 3–4-fold for A549 cells. However, such treatment did not render HT29 cells as sensitive to MTIC as VA13 cells. It seems probable that more than one type of cytotoxic lesion is involved.

### Sensitivity of methotrexate resistant colorectal cancer cells to monoclonal antibody targeted methotrexate

L. Durrant, N.C. Armitgage, M.C. Garnett,
R.W. Baldwin & J.D. Hardcastle

*Departments of Cancer Research and Surgery, University of Nottingham, Nottingham, UK.*

A major problem in the chemotherapy of colorectal cancers is resistance to most cytotoxic drugs which may be due to insufficient cellular transport mechanisms. Drugs conjugated to monoclonal antibodies recognising tumour antigens may overcome these difficulties providing access of active agents to the tumour cells. The antitumour monoclonal antibody 791T/36, shown to localise in patients with colorectal cancer (Armitage *et al.*, *Br. J. Surg.*, **71,** 407, 1984) has been investigated as a potential targeting antibody. Three cell lines (C146, C168, C170) were newly established from surgically resected material and the cytotoxicity of 791T/36-methotrexate compared with that of free methotrexate by a $^{75}$Se-selenomethionine incorporation assay. The dose necessary to achieve 50% cell killing ($IC_{50}$) was calculated. Antibody binding was measured flow cytometrically by indirect fluorescence.

| | Antibody binding FI units/cell | |
|---|---|---|
| *Cell lines* | *Control NMT* | *791T/36* |
| C146 | 26±8 | 377±70 |
| C168 | 19±9 | 181±44 |
| C170 | 11±5 | 182±16 |

| | Cytotoxicity LC50 ($ng\,ml^{-1}$) | |
|---|---|---|
| *Cell lines* | *Methotrexate* | *791T/36-Methotrexate* |
| C146 | 972±66 | 51±11 |
| C168 | 5,179±1,166 | 357±42 |
| C170 | 1,790±168 | 162±26 |

In all cell lines there was significant binding of antibody (T=6.7, df 8 $P<0.001$) and significant cytotoxicity for 791T/36-methotrexate with resistance to free methotrexate (T=4.1, df 9 $P<0.005$).

Monoclonal antibody directed chemotherapy rendered methotrexate resistant colorectal cancer cells sensitive to that agent, providing further impetus for trials of antibody-drug conjugates in patients with colorectal cancer.

### Cytotoxicity against human tumour cells by 'cocktails' of monoclonal antibody-drug conjugates

M.J. Embleton[1], J. Harte[1], V.S. Byers[2],

M.C. Garnett[1], J. Gallego[1] & R.W. Baldwin[1]

[1]*Cancer Research Campaign Laboratories, University of Nottingham, Nottingham NG7 2RD, UK and* [2]*The XOMA Corporation, Berkeley, CA 94710, USA.*

In an attempt to increase *in vitro* cytotoxicity against cultured human tumour cells mediated by drug-antibody or antibody-toxin conjugates, tumour cells were exposed to different conjugates in combination. The conjugates used were; (a) methotrexate linked by human serum albumin to C24, a monoclonal antibody to CEA/NCA; (b) methotrexate linked by human serum albumin to antibody 791T/36, raised against 791T osteogenic sarcoma cells; (c) daunomycin linked directly to 791T/36, and (d) ricin toxin A chain linked directly to 791T/36. Cytotoxicity was analysed by a 40 h $^{75}$Se-selenomethionine incorporation test on cell lines 791T (osteogenic sarcoma), MKN45 (gastric carcinoma), and LS174T and C170 (colon carcinomas).

Each conjugate as a single agent was toxic to cells expressing the antigen detected by its respective antibody moiety and most were selective, although their individual levels of performance were variable (d>b>a>c). Mixtures of conjugates prepared with the same antibody (791T/36) but different drugs showed no increase in cytotoxicity over that obtained with the most effective single agent, but there was a trend towards greater cytotoxicity when conjugates prepared with different antibodies reacting with the same target cell line were used in combination. These results support the conclusion that the effectiveness of agents targeted with a single antibody is limited by target antigen density, but conjugates prepared with antibodies recognising different target epitopes may have an additive effect. It is suggested that, given conjugates of optimal performance, cocktails of conjugates recognising different target cell antigens might offer a means of achieving significantly increased anti-tumour activity.

### Conjugates of N-(2-hydroxypropyl)methacrylamide copolymers and daunomycin: Toxicity against L1210 leukaemia *in vitro* and *in vivo*

R. Duncan[1], P. Kopečeková-Rejmanová[2], J. Kopeček[2] & J.B. Lloyd[1]

[1]*Department of Biological Sciences, University of Keele, Keele, Staffordshire ST5 5BG, UK and* [2]*Institute of Macromolecular Chemistry, Czechoslovak Academy of Sciences, 162 06 Prague 6, Czechoslovakia.*

Drugs used in cancer chemotherapy are notorious for their lack of specificity of action. Over recent years we have developed a soluble synthetic polymer (N-(2-hydroxypropyl)methacrylamide; HPMA) as a drug carrier for tumour-specific drug delivery (Kopeček *et al., Ann. New York Acad, Sci.*, **446**, 93, 1985). HPMA copolymers have been synthesised bearing the side-chains P-GlyPheLeuGly-daunomycin (biodegradable) or P-GlyGly-daunomycin (non-biodegradable). Certain polymers also have pendent fucose residues (L1210 leukaemia cells have a cell-surface receptor that recognises and binds this carbohydrate moiety). The conjugates were tested against L1210 cells *in vitro* and *in vivo*.

HPMA copolymers bearing daunomycin inhibited growth of L1210 *in vitro*. Although less active than equivalent concentrations of free drug, activity did correlate with the enzymic degradability of the polymer-drug linkage. The presence of fucose residues potentiated toxicity of the conjugate *in vitro*. Conjugates with biodegradable polymer-drug linkages significantly increased the lifespan of mice previously inoculated i.p. with $10^5$ L1210 cells. Conjugates with a nondegradable linkage were completely ineffective *in vivo*.

### A comparison of monoclonal anti-CEA antibodies for localisation and therapy of gastrointestinal malignancies

F. Macdonald, W.H. Allum, P. Life & J.W.L. Fielding

*Surgical Immunology Unit, Queen Elizabeth Hospital, Birmingham, UK.*

Antibodies for use as carriers of isotopes or drugs for radioimmunolocalisation or drug therapy have to be carefully characterised before use in patients. Four monoclonal antibodies to CEA, recognising four different epitopes, have been compared alone and in combination for their ability to localise CEA expressing tumours in a mouse xenograft model (HT29 and MKN-45 cell lines). One antibody, 11-359-6, lost its anti-CEA activity on labelling. 11-357-5 showed the highest tumour:blood ratios, 1.84 by 72 h but this ratio decreased by 96 h suggesting specific loss by the tumour. In addition, this antibody cross-reacted with normal stomach. Both 11-285-14 and 14-95-55 showed increasing ratios in the tumour up to 96 h (1.45 and 1.31 respectively) but no uptake by normal tissues. The maximum tumour:blood ratio obtained by either 11-285-14 or 11-357-5 alone was not increased by mixing the two antibodies. However the ratios of the mixture at 24 h and 48 h were higher than either antibody alone at these times. In addition, high specific counts were obtained in the tumour.

Combinations of 11-285-14 and 14-95-55 did not increase the tumour:blood ratio and showed significantly lower levels than either alone (max. ratio at 96 h of 0.9). The fate of cell surface associated antibodies was followed by FACS analysis. 11-357-5, 11-359-6 and 11-285-14 remained associated with the cell surface at 37° C and 4° C. However at 37° C, 14-95-55 is endocytosed.

These results suggest that 11-285-14 is the most suitable antibody for radioimmunolocalisation with $^{131}$I-labelled antibody since it may be dehalogenated if internalised, resulting in loss of isotope from the cell. 14-95-55 may be more suited to targeted therapy particularly if drugs or toxins have to enter the cell to have cytotoxic effects.

**Experimental and clinical imaging of gastrointestinal carcinomas with $^{111}$In-labelled anti-CEA monoclonal antibodies**

M.V. Pimm[1], A.C. Perkins[2], K.D. Ballantyne[3], L. Durrant[1], I. Pawluczyk[1], L. Jacobs[1], M.R. Price[1], J.D. Harcastle[3] & R.W. Baldwin[1]

[1]*Cancer Research Campaign Laboratories, University of Nottingham and Departments of* [2]*Medical Physics and* [3]*Surgery, University Hospital, Nottingham, UK.*

Monoclonal antibodies against CEA have wide potential as radiopharmaceuticals for tumour imaging and in the present study three new antibodies have been examined.

Antibodies 161 and 198 (IgGl), reacted with a CEA/NCA epitope. Antibody 228 (IgG2a), produced against purified CEA, reacted with a CEA specific epitope. Antibodies were radiolabelled with $^{111}$In because of the attractive physical characteristics of this radionuclide for immunoscintigraphic studies. To permit radiolabelling, antibodies were conjugated to diethylenetriamine penta acetic acid (DTPA) by reaction with DTPA anhydride at a 2:1 molar ratio. Subsequent chelation of $^{111}$In gave products with specific activities of 100 MBq mg$^{-1}$ and which bound to CEA producing target cells but not to antigen negative cells. Gel filtration chromatography of labelled antibodies in the serum of mice showed radiolabelled material predominantly as monomeric IgG, with no aggregated product or transfer of $^{111}$In to transferrin. Gamma scintigraphy of nude mice with LS174T and HT29 colon carcinoma and MKN 45 gastric carcinoma xenografts showed localisation of $^{111}$In-labelled antibodies in tumours.

The levels of radioactivity in tumour and normal colon from five patients given $^{111}$In-161 antibody gave mean tumour:normal issue uptake ratios of 5.8:1 but no tumour sites were detected by external imaging. $^{111}$In-198 antibody also failed to image tumours, but showed high bone marrow uptake. $^{111}$In-228 antibody showed intermediate bone and liver uptake.

These studies illustrate the diverse nature and tumour imaging properties of anti-CEA monoclonal antibodies.

**Monoclonal antibody-drug conjugate 791T/36-methotrexate localisation in colorectal cancer**

K.C. Ballantyne, A.C. Perkins, M.V. Pimm, M.C. Garnett, N.C. Armitage, R.W. Baldwin & J.D. Hardcastle

*Departments of Surgery, Medical Physics & Cancer Research Campaign Laboratories, University of Nottingham, Nottingham, UK.*

The monoclonal antibody 791T/36 has previously been shown to localise in colorectal cancer with a tumour:nontumour (T:NT) ratio of 2.5:1 (Armitage *et al., Br. J. Surg.*, **71**, 407, 1984). This antibody has been successfully conjugated to methotrexate (MTX) for use in anticancer therapy (Garnett *et al., Int. J. Cancer*, **31**, 661, 1983). The aim of this study was to establish whether conjugation of this antibody affected its tumour localisation or biodistribution.

Ten patients with primary colorectal cancer were injected intravenously with $^{131}$I-radiolabelled 791T/36-MTX (200 $\mu$g antibody and 1.6 $\mu$g methotrexate), following a subcutaneous test dose. Gamma-camera antibody images were obtained at 48–72 h and analysed using computerised subtraction. Freshly resected surgical tissue (tumour and normal colon) and daily serum samples were counted and T:NT uptake ratios and blood clearance profiles calculated. The distribution of 791T/36-MTX was similar to that of free antibody. On direct measurement there was positive tumour uptake in all cases with a mean T:NT of $3.9 \pm 2.1$:1. The clearance of the drug-antibody conjugate was biphasic with a biological half life of 22 h.

Conjugation of methotrexate to the monoclonal antibody 791T/36 did not change its tumour localisation or biodistribution and trials of drug-antibody conjugate are indicated in colorectal cancer.

**Dose dependent biodistribution and tumour discrimination with a radiolabelled monoclonal antibody**

M.V. Pimm, R.A. Robins, W. Pascoe, M.R. Price & R.W. Baldwin

*Cancer Research Campaign Laboratories, University of Nottingham, Nottingham, UK.*

There are now numerous clinical reports of *in vivo* tumour localisation of radiolabelled monoclonal antibodies against tumour associated antigens. Sometimes efficiency of tumour detection is improved by increasing the dose of radiolabelled antibody (Murray *et al., Proc. Amer. Assoc. Cancer Res.*, **25**, 250, 1984) but since doses of radionuclide remain constant the reason(s) for this are obscure. In the present study a dose dependent discrimination between tumour and normal tissues has been demonstrated and investigated in a rat mammary tumour model.

The mouse IgGl monoclonal antibody 226 was prepared against rat mammary carcinoma (Sp4). Cytofluorimetry showed that it did not react with cells of other rat tumours, including mammary carcinomas. Purified antibody was labelled with $^{125}$I and normal mouse IgGl with $^{131}$I for dual label *in vivo* distribution studies in rats with Sp4 tumour. With large doses ($20\,mg\,kg^{-1}$) of antibody, tissue to blood (T:B) ratios of $^{125}$I-antibody in tumour were higher than those of intestine, lung, heart, spleen, kidney and muscle. With lower doses ($30\,\mu g\,kg^{-1}$) those of intestine and lung exceeded that of tumour, and this was most marked with lung with T:B ratios 3.5 times that of tumour. With even smaller doses ($6\,\mu g\,kg^{-1}$) T:B ratio of kidney also exceeded that of tumour and in comparison with normal IgGl, maximum localisation indices of antibody in intestine, lung and kidney were 9, 8 and 5 compared with 2 for tumour. Circulating immune complexes were demonstrated in serum of rats receiving low but not not higher does of antibody. Complexes were ot seen with normal IgGl. Immunohistochemistry subsequently confirmed reaction of the antibody with luminal surfaces of intestine, but clear reaction with lung and kidney was not seen.

These studies indicate that improved discrimination between tumour and normal tissues with increased doses of monoclonal antibody may be due, at least in part, to low levels of antigen expression in normal tissues.

**Tissue-localisation of adriamycin (ADR) using albumin microspheres**

N. Willmott[1], D. Kerr[2], H. Lewi[3], J. McKillop[4] & C. McCardle[3]

[1]*Department of Pharmaceutics, University of Strathclyde,* [2]*Department of Medical Oncology, University of Glasgow,* [3]*Department of Surgery and* [4]*Department of Nuclear Medicine, Royal Infirmary, Glasgow, UK.*

Microspherical drug delivery systems of requisite size hold out the possibility of conferring specificity on cytotoxic anti-cancer drugs by chemo-embolisation in capillary beds of organs harbouring tumour deposits. This will lead to increased exposure of the organ to drug combined with correspondingly lower systemic levels, resulting in increased therapeutic ratio. To examine these points in experimental systems, rats ($70\,\mu g$ ADR intravenously and rabbits ($220\,\mu g$ ADR via a renal artery) were injected either with ADR in solution or in microspherical form ($15\,\mu m$ diam., rat; $25\,\mu m$ rabbit). In both systems, by the use of light and fluorescence microscopy, and $^{99m}$Tc-labelled particles, it was seen that microspheres were trapped with high efficiency in rat lung and rabbit kidney, i.e. in the first capillary beds encountered. In the rat, mean systemic serum levels of ADR immediately after injection were considerably reduced after administration in microspherical form ($270\,ng\,ml^{-1}$ ($n=2$) *versus* $46\,ng\,ml^{-1}$ ($n=6$) as were mean systemic rabbit plasma levels ($118\,ng\,ml^{-1}$ ($n=2$) *versus* $<5\,ng\,ml^{-1}$ ($n=2$). $^{99m}$Tc-labelled microspheres have been co-administered with 5-fluorouracil (1 g) to a patient with multiple hepatic metastases from colorectal carcinoma, via a catheter surgically implanted in the hepatic artery. Using radionuclide angiographic tomography it has been possible to visualise the hepatic metastases, and preliminary evidence suggests that intrahepatic arterial infusion of angiotensin II might increase delivery of microspheres to tumour deposits.

**The effect of niosome encapsulation on the distribution of adriamcyin in the mouse**

A. Rogerson[1], J. Cummings[2] & A.T. Florence[1]

*Departments of* [1]*Pharmacy, Strathclyde University and* [2]*Medical Oncology, Glasgow University, Glasgow, UK.*

Thse use of carriers (e.g. liposomes and macromolecular complexes) to deliver drugs to target organs and modify drug disposition has been widely investigated. We have been studying a new vesicle-forming synthetic system based on non-ionic surfactants. These multilamellar vesicular systems, termed niosomes, have the capacity to entrap and retain drugs such as methotrexate and adriamycin. We have compared the tissue distribution of adriamycin in two forms of niosomes (comprising 100% surfactant and 50 mol % surfactant/cholesterol) with free adriamycin in male NMRI mice bearing the subcutaneous rapidly proliferating sarcoma S180 tumour. Three mice were sacrificed per time point at intervals, up to 48 h, following an i.v. bolus of 5 mg kg$^{-1}$ adriamycin. Plasma and tissue levels of adriamycin and its metabolites were measured by an HPLC technique employing fluorescence detection. Encapsulation of adriamycin decreased its peak plasma levels (t = 10 m) from 14,700–6,800 ng ml$^{-1}$ and prolonged its circulation (there being a five-fold enhancement of drug in plasma at 16 h from 20–90 ng ml$^{-1}$), as was similarly reported for methotrexate-loaded niosomes, but there was no significant increase of adriamycin in the liver. However in the liver, spleen and heart, adriamycin levels were highest following administration of drug in cholesterol-containing niosomes. In the plasma there was no difference between the niosomes types. Levels of niosomal-adriamycin were slightly higher in the S180 tumour and its rate of growth was significantly reduced by administration of 2.5 mg kg$^{-1}$ adriamycin in cholesterol-free niosomes (mean growth rate of 0.45 *versus* 0.24 g day$^{-1}$ over a period of 16 days).

**Quantitation of anti-tumour antibody and antibody conjugate binding activity by competition with fluorochrome labelled antibody**

R.A. Robins[1], R.R. Laxton[2], M. Garnett[1], M.R. Price[1] & R.W. Baldwin

[1]*Cancer Research Campaign Laboratories and* [2]*Department of Mathematics, Nottingham University, Nottingham NG7 2RD, UK.*

Binding of unlabelled monoconal antibody preparations has been assessed by competition at saturation with fluorochrome labelled homologous antibody for binding to antigen bearing target cells. The extent of competition was measured by quantitative flow cytofluorimetry, and simple mathematical procedures have been developed to allow interpretation of competition data in terms of antibody binding activity. In the system studied, non-specific (non-competitive) fluorescence was minimal, but an iterative method to calculate its contribution to the measured signal is given. This approach has the advantage that the antibody preparation to be tested does not need to be labelled or modified; this is particularly important when evaluating the binding activity of therapeutic antibody conjugates. Comparison to a well charactertised standard antibody preparation provides a rapid, sensitive and accurate quality control procedure. Qualitative (affinity) changes in modified antibody are detectable, as well as quantitation of total loss of binding activity. This test is also simple to perform, requiring only mixing of labelled and unlabelled antibodies with target cells, a single incubation, followed by analysis without washing the target cells.

**TRITC-HSA-antibody conjugates: A convenient reagent for assessment of endocytosis of cell surface antigens**

M.C. Garnett & R.W. Baldwin

*Cancer Research Campaign Laboratories, University of Nottingham, University Park, Nottingham NG7 2RD, UK.*

When tetramethyl rhodamine isothiocynate (TRITC) was coupled to human serum albumin (HSA) at high molar substitution ratios (12 to 30 mol TRITC mol$^{-1}$ HSA), rhodamine fluorescence was quenched by over 90%. On conjugation of TRITC-HSA to 791T/36 monoclonal antibody a reagent useful for monitoring endocytosis was obtained. When 788T or 791T cells expressing high levels of 791T/36 antigen were stained at 0° C the cells could be visualised by a dim, even surface fluorescence. However, on incubation of the cells at 37° C for a few hours (4.5 h) a bright punctate perinuclear distribution of fluorescence was seen due to the endocytosis and digestion of the reagent to reveal the unquenched fluorescence. Unlike similar work performed with fluorescein compounds (*Br. J. Cancer*, **52**, 432, 1985), no further addition of modifiers was required to demonstrate the fluorescence by alteration of the pH of intracellular organelles. Results were therefore easy to obtain and their interpretation was unambiguous. The sensitivity of this assay for endocytosis of cell surface antigens has been investigated using a number of cultured cell lines expressing different amounts of cell surface antigen. In particular on a bladder carconoma cell line T24 expressing only $3 \times 10^4$ antigens per cell, initial surface fluorescence was undetectable with TRITC-HSA-791T/36, but endocytosis after 4 h was clearly visible. This suggested that levels of endocytosis of ~10,000 mol could be visualised using this method. No endocytosis was seen on a melanoma cell line Mel 57 expressing no detectable antigen. The results of this assay using conjugates with antibodies recognising other antigens will be presented. This assay which was carried out on living cells is less subject to misinterpretation than other more lengthy procedures e.g. electron miscroscopy and can be performed in a few days including synthesis of conjugate. This assay may help to resolve the question of whether all or just some cell surface antigens are endocytosed.

### Binding of a panel of monoclonal antibodies to primary and metastatic colorectal cancer

K.C. Ballantyne, L.G. Durrant, N.C. Armitage, R.A. Robins, R.W. Baldwin & J.D. Hardcastle

*Departments of Surgery and Cancer Research, University of Nottingham, Nottingham, UK.*

Although there are reports of the pattern of antigen distribution in primary colorecral tumours as defined by monoclonal antibodies, the distribution and degree of antibody binding to metastatic compared with primary colorectal cancer has not yet been fully evaluated. Using flow cytometry and immunohistology we have now assessed the binding of the monoclonal antibodies 791T/36, antiosteosarcoma, C14/1/46, anticolonic adenoma, C154/14 anticolorectal carcinoma and C161/25 anti CEA/NCA to 50 primary colorectal cancers, 17 lymph node metastases and 21 hepatic/peritoneal metastases.

After disaggregation tumour cell binding was measured by flow cytometry using indirect immunofluorescence. Median linear fluorescence values (MLF) were corrected for non-specific binding using normal mouse immunoglobin (Table). Immunohistology was performed using the indirect peroxidase technique. Immunohistology demonstrated that the antigen distribution of primary colorectal cancer is retained in metastatic tumour deposits. Confirmation that metastases retain similar pattern of antigen expression to primary colorectal cancer provides further evidence that targeted immunotherapy may effectively treat metastatic colorectal cancer.

| *Antibody* | *Median tumour MLF Primary tumours* | *(Range) Fl.U Lymph node metastases* |
|---|---|---|
| 791T/36 | 134 (0–655) | 139 (0–888) |
| C14/1/46 | 415 (44–2,481) | 689 (0–2,345) |
| C154/14 | 242 (0–1,108) | 289 (0–1,206) |
| C161/25 | 899 (0–3,432) | 547 (0–2,848) |

| *Antibody* | *Hepatic/peritoneal metastases* |
|---|---|
| 791T/36 | 139 (0–995) |
| C14/1/46 | 392 (0–2,349) |
| C154/14 | 391 (51–2,081) |
| C161/25 | 803 (96–3,614) |

### Effect of endotoxin on the expression of drug metabolising enzymes

C.R. Wolf, R.L. Lindsay, L. Roger & D.J. Adams

*Imperial Cancer Research Fund, Laboratory of Molecular Pharmacology, Hugh Robson Building, George Square, Edinburgh, UK.*

Our interest in the effects of endotoxin and interferons on the drug metabolising enzymes stems from the potential effects of inflammation and infection on the activation of chemical carcinogens and changes in normal and tumour tissues when cytotoxic drugs are used in combination with lymphokines. Using Western Blots and isozyme specific substrates a single high dose of entotoxin (25 $\mu$g) had profound effects on cytochrome P-450 gene expression in the liver. Total cytochrome P-450 levels were reduced by 40%, 44% and 61% in control, phenobarbital (PB) and methylcholanthrene (3-MC)-treated mouse liver. There was however, a differential effect on P-450 isozyme levels, the major PB-inducible isozyme ($PB_3$) being virtually unaffected by this endotoxin-treatment. It was very interesting that at lower doses of endotoxin (7.5 $\mu$g) the induction of the major 3-MC-inducible P-450 (MC1b) was potentiated (2.2 fold). The induction of the PB-inducible isozymes $PB_1$ and $PB_2$ was also significantly increased, maximal elevation being 3 and 1.7 fold above controls respectively. These changes were substantiated by measuring the metabolism of 7-ethoxyresorufin and 7-ethoxycoumarin. These data indicate that inflammatory and antiviral response could have significant effects on the activation and/or deactivation of chemical carcinogens and that the use of lymphokines may significantly alter the action and side reactions of cancer chemotherapeutic agents.

### Benznidazole inhibition of CCNU hydroxylation by hepatic microsomal cytochrome P-450

P. Workman[1], F.Y.F. Lee[1], J.T. Roberts[1], M.I. Walton[1], K.H. Cheeseman[2] & N.M. Bleehen[1]

[1]*MRC Unit and University Department of Clinical Oncology, Hills Road, Cambridge and* [2]*Biochemistry Department, Brunel University, Kingston Lane, Uxbridge, UK.*

Benznidazole is a potent nitroimidazole chemosensitiser. It produces an improved therapeutic index with the nitrosourea CCNU in mice, and this combination is now being evaluated clinically in an MRC randomised Phase 2 study of recurrent glioma. We have previously shown that benznidazole inhibits the clearance of CCNU and reduces the rate of hydroxylation by hepatic microsomal cytochrome P-450. In continuing studies of the mechanism of action of benznidazole we have used optical difference spectroscopy to demonstrate that benznidazole binds to cytochrome P-450 of phenobarbitone-induced mouse liver microsomes *in vitro*. Binding is predominantly type II but with some suggestion of a type I component. The *in vitro* kinetics of inhibition of CCNU hydroxylation by control mouse liver microsomes are of the mixed competitive-non-competitive type. Ki (0.044 mM) was lower than Ki′ (0.17 mM) indicating the predominance of the competitive component. Using these *in vitro* data we have estimated the amount of inhibition of CCNU hydroxylation likely to result from concentrations of benznidazole achieved in man. The extent of inhibition of CCNU metabolism in man, measured by the appearance of parent CCNU in the plasma, correlated well with the predicted inhibition of CCNU hydroxylation. For benznidazole doses of 20–25 $mg\,kg^{-1}$ the predicted inhibition was in range 20–43%. This was comparable to that achieved in optimal chemosensitisation protocols in mice.

### Inhibitors of aromatase based on 3-phenylpyrrolidine 2,5-dione: Structure-activity relationships

G.W. Jones, W.M.A. Nazareth, M.J. Daly, M.I. James, P.J. Nicholls & H.J. Smith

*Welsh School of Pharmacy, UWIST, Cardiff, UK.*

The aromatase inhibitor, aminoglutethimide (I, 3-(4-aminophenyl)-3-ethyl piperidine-2,6-dione) has been used for the treatment of oestrogen-receptor positive breast cancer in post menopausal women. It is co-administered with a glucocorticoid to suppress the resulting reflex-rise in ACTH level due to inhibition of the CSCC enzyme. We have recently shown that unsubstituted and 3-alkyl substituted 3-(4-aminophenyl) pyrrolidine-2,5-diones (II) are equipotent with (I) as inhibitors of placental aromatase and we consider that they are potentially more useful clinical agents than (I) since they showed little or no activity against bovine CSCC *in vitro*. We have prepared further analogues of (II) where (a) the heterocyclic ring has been altered (b) a spacer group (-$CH_2$-) placed between the two rings, (c) rotation of the aryl ring has been restricted. These changes were usually accompanied by loss of ability to inhibit aromatase. We have examined the structure-activity relationships between (I), (II) and the other types of compounds mentioned here using molecular graphics (Chemgraf) and our findings are that the active inhibitors have (1) a near-flat hydrophobic monoheterocyclic ring, (2) a p-aminophenyl (or 4-pyridyl) ring which is in a plane above (below) the plane of (1), (3) restriction of the conformer population about C3 to a limited number of low energy forms with torsion angles (C2-C3-C1′-C2′(aromatic)) of $-10°$–$+50°$ in order that the ‘active conformation’ may be relatively more populated.

### Aziquone (AZQ) instability in frozen solutions

A.G. Bosanquet

*Department of Clinical Investigation, Royal United Hospital, Combe Park, Bath BA1 3NG, UK.*

Common sense would suggest that drugs in solution would be more stable frozen than not frozen except where solubility limits are exceeded at the lower temperatures, and this is the case for most anticancer drugs. I have used a high-performance liquid chromatographic method to investigate the stability of AZQ in various solutions at temperatures from $-196°C$ to $+50°C$. AZQ dissolved at 20 $mg\,ml^{-1}$ in dimethyl-acetamide (DMA) and diluted to 1 $mg\,ml^{-1}$ with the phosphate (Ⓟ) buffer provided, was (after 9 days) most stable at $+4°$ and $\leqslant -70°C$. Minimum stability was found at $-12°C$ with increasing stability as the temperature was lowered. When diluted further with Ⓟ buffered saline (PBS), the drug was again most stable at $+4°C$ and $\leqslant -86°C$. However, the minimum stability was found to be at $-35°C$ (with $<30\%$ of the initial AZQ concentration remaining after only 24 h) with samples stored at $-12°C$ being almost as stable as those at $+4°C$. Identical samples at $-20°C$ showed enormous variation in stability – most being unstable ($<30\%$ AZQ remaining after 24 h), but a few being essentially stable ($>90\%$ remaining). In *dilute* solutions (10 $\mu g\,ml^{-1}$) in Ⓟ or other ions (but without $Cl^-$ present) AZQ was reasonably stable frozen ($\geqslant 90\%$ present at day 7). In another experiment with dilute solutions, where different ions were mixed with NS, only the Ⓟ/NS mixture produced instability at $-35°C$. The products of AZQ's degradation in PBS are chloro-AZQ and dichloro-AZQ where first one and then both aziridinyl rings open to become chlorethylamino groups. Degradation at positive temperatures results in hydroxyethylamino groups (tentative assignment) as would be expected in aqueous environments. Obviously AZQ should not be frozen in solutions containing Ⓟ or Ⓟ/NS. AZQ dissolved in DMA and diluted with 0.15 M citrate/NaOH pH 6.3 to 10 $\mu$g ml$^{-1}$ has successfully been stored at $-20°$C and $-70°$C for 2 months with no significant degradation taking place.

**Investigation into the stability of a combination of adriamycin, vincristine and etoposide**

D.G. Poppit & B.W. Fox

*Paterson Laboratories, Christie Hospital & Holt Radium Institute, Manchester M20 9BX, UK.*

The three drugs: adriamycin (doxorubicin), vincristine (oncovin) and etoposide (VP16 – vepesid) at concentrations of 1.5, 0.05 and 12 mg ml$^{-1}$ respectively were investigated both singly and in combination for stability over three days by high pressure liquid chromatography and UV absorption spectroscopy. The agents were in the formulation supplied by the manufacturers and dilutions were with saline. On a Lichrosorb 5Si60 analytical column, adriamycin and etoposide could be resolved with a running solvent of 35% acetonitrile in 0.01 M phosphoric acid but a gradient of 5%–50% acetonitrile in 0.01 M phosphoric acid was necessary to resolve vincristine from etoposide. Using this gradient system vincristine, etoposide and adriamycin have retention times of 265, 310 and 445 sec respectively at a flow rate of 1 ml min$^{-1}$ and UV detection at 254 nm. UV spectra showed absorption maxima at 496, 295 and 258 nm attributable to adriamycin, etoposide and vincristine respectively. There was no evidence of any change in either the HPLC elution profile or the UV spectra over the 3 days studied. Further tests after 14 days indicated similar stability.

**Inhibition of oestradiol biosynthesis *in vivo* by aminoglutethimide and related compounds**

M.H. Pourgholami, M.J. Daly, P.J. Nicholls & H.J. Smith

*The Welsh School of Pharmacy, PO Box 13, Redwood Building, Cardiff CF1 3XF, UK.*

We have developed a series of compounds possessing selective aromatase inhibitory activity, and have evaluated two of these compounds for their effects on oestradiol (E2) biosynthesis in the rat. Ten week old female rats were primed with pregnant mares serum gonadotrophin (100 iu s.c.) every other day for 10 days, followed on the 11th day by a single i.p. dose (50 mg kg$^{-1}$) of either aminoglutethimide (1), 3-(4-aminophenyl)-3-ethyl- pyrrolidine-2,5-dione (2) or the 1-methyl analogue of the latter (3). Three hours later, cardiac blood was collected under ether anaesthesia for the determination of E2 blood levels by radioimmunoassay. Each compound decreased E2 blood levels to 7, 7 and 28% of control levels respectively ($n=6$). Residual aromatase activity, assessed by incubating the homogenised ovaries with [1$\beta$,2$\beta$-$^3$H] androstenedione (0.2 $\mu$M), indicated that enzyme activity was also reduced compared with controls. The new aromatase inhibitors 2 and 3 are therefore effective inhibitors of E2 biosynthesis in the rat with functioning ovarian activity. The human ovary is resistant to the effects of 1, which denies pre-menopausal breast cancer patients potentially beneficial therapy. The selectivity of the new agents may enable their use in either pre- or post-menopausal women with breast cancer, or E2-dependent ovarian disease.

**Primary H/D kinetic isotope effect on the metabolism of the antitumour agent N-methylformamide**

M.D. Threadgill, P. Kestell, K.C. Farrow & A. Gescher

*CRC Experimental Chemotherapy Group, Pharmaceutical Sciences Institute, Aston University, Birmingham, UK.*

Methylamine (I) and *S*-(*N*-methylcarbamoyl)-*N*-acetylcysteine (II) have been identified as major urinary metabolites of the hepatotoxic antitumour agent *N*-methylformamide (III; CCRG 80011) in rodents and in man (Kestell *et al.*, *Drug Metab. Disposit.* **13**, 587, 1985; & this meeting). Formation of II must involve oxidative rupture of the formyl C-H bond in III, whereas methylamine may arise either from enzymatic hydrolysis of the parent III or *via* some intermediate in which this bond has already been oxidatively cleaved (e.g. in II). A known mixture of deuterated isotopomers of III ($OHCNHCD_3 + ODCNHCH_3$) was given i.p. at a total dose of 400 mg kg$^{-1}$ to 3 CBC/CA mice. Methylamine in 24 h urine collections from each mouse was converted to N-methyl-2,4-dinitroaniline (IV) by treatment with 1-fluoro-2,4-dinitrobenzene and its isotopic composition was determined by mass spectrometry. A primary hydrogen/deuterium kinetic isotope effect of $5.5 \pm 0.2$ was observed for the metabolism of III to I, such a large effect indicating that cleavage of the formyl carbon-hydrogen bond is rate-limiting in this process, thus ruling out direct hydrolysis. A H/D effect of $1.6 \pm 0.4$ was observed for the formation of II.

**The possible role of *in vivo* cardic drug metabolism in the cardiotoxic actions of adriamycin (ADR) and 4′-deoxydoxorubicin (4′-deoxy) in mice**

J.G. Morrison, D. Kerr, J. Cummings & S.B. Kaye

*Department of Medical Oncology, University of Glasgow, Glasgow, UK.*

ADR and 4′-deoxy are two anthracycline drugs which are reported to produce different degrees of cardiotoxicity in animal models. We have determined the cardiac kinetics and metabolism of ADR and 4′-deoxy. NMRI mice were injected i.p. ($10\,mg\,kg^{-1}$) with ADR and 4′-deoxy. The animals were killed with ether at intervals of up to 24 h (4 animals were used for each experimental point). A plasma sample was taken and the hearts rinsed once in saline, blotted dry and then frozen at $-20°C$ until analysed. The levels of ADR, 4′-deoxy and their metabolites were estimated by sensitive and specific HPLC techniques devised in our laboratory. The kinetics of cardiac disposition were similar for both drugs; peak levels (ADR $2.5\,\mu g\,g^{-1}$, 4′-deoxy $2.3\,\mu g\,g^{-1}$); cardiac AUC (ADR $18\,\mu g^{-1}\,h$, 4′-deoxy $20\,\mu g\,g^{-1}\,h$); elimination half-life (ADR 38 h, 4′-deoxy 36 h). It was not possible to detect formation of deoxyaglycones from 4′-deoxy, whereas the deoxyaglycones of adriamycin (4% of total drug content) and adriamycinol (3% of total drug content) were present in significant amounts. There is evidence to suggest that the cardiotoxicity of adriamycin is mediated *via* a semi-quinone free radical and superoxide free radical generated in cardiac mitochondria. 7-Deoxyaglycones are end products of semi-quinone free radical production. It is possible that differential intra-cardiac aglycone production, and by inference free radical formation, could account for different cardiotoxicity of these two 2 drugs.

**Preclinical evaluation of 1-p-Carboxy-3,3-dimethylphenyltriazene (CB 10-277) – An alternative to DTIC**

C.J. Rutty, M.A. Graham, G. Abel, I.R. Judson & P.M. Goddard

*Inst. Cancer Res., Sutton, Surrey, UK.*

1-p-Carboxy-3,3-dimethylphenyltriazene (CB 10-277) has been selected for phase I clinical study as an alternative to DTIC (Dacarbazine). This particular phenyltriazene has shown marked activity against experimental murine tumours and melanoma xenografts, and is significantly more effective than DTIC in inhibiting the growth of the Walker tumour grown in the rat. In common with other dialkyltriazenes CB 10-277 requires oxidative N-demethylation in order to exert its antitumour effect. The parent dimethyl compound is relatively non-toxic to PC6 cells in culture whereas the metabolite 1-p-carboxy-3-hydroxymethyl-3-methyltriazene (CB 10-440) is highly toxic ($ID_{50} = 5.9\,\mu M$). In rodents cytotoxic levels of this metabolite are readily achieved, especially in the rat where the peak plasma level is $56.2 \pm 3.5\,\mu M$ as opposed to $25.3 \pm 1.8\,\mu M$ in the mouse, in marked contrast to DTIC. In addition the acyl-glucuronide of CB 10-277 is a major metabolite present in the plasma and urine of both species. Both CB 10-277 and CB 10-440 are extensively bound to plasma proteins (95%) leading to stabilisation of the active metabolite ($t\frac{1}{2} = 60\,min$). Furthermore, the carboxy-triazene penetrates the mouse CNS relatively poorly (brain/plasma ratio = 0.32) which may result in reduced neurotoxicity. CB 10-277 is highly water soluble and can be readily formulated as a stable preparation in 150 mM $NaHCO_3$ pH 8.3 ($t\frac{1}{2} > 16\,h$) for i.v. administration to patients.

**Pharmacological studies with the anthrapyrazole CI 941: A new DNA complexing drug**

M.A. Graham, D.R. Newell & A.H. Calvert

*Drug Development Section, Institute for Cancer Research, Sutton, Surrey, UK.*

The anthrapyrazole CI 941 is one of a new series of mitozantrone analogues the anthra[1,9cd]pyrazol-6(2H)-ones, which have been selected for further clinical investigations because of broad spectrum activity against rodent tumours (e.g., B16, L1210 and P388) and their novel biochemical properties (Fry *et al., Biochem. Pharmacol.*, **34**, 3499, 1985). The pharmacokinetics and toxicity of CI 941 have been investigated prior to clinical studies. Following an i.v. bolus injection ($15\,mg\,kg^{-1}$) to female Wistar rats the drug is rapidly cleared from the plasma $t\frac{1}{2}\alpha = 3.11\,min$, $t\frac{1}{2}\beta = 34.4\,min$, clearance = $131\,ml\,min^{-1}\,kg^{-1}$) and is subjected to both urinary (<1% dose) and biliary excretion (12–18% dose). Tissue distribution, 3 h post administration, shows 6.4%, 7.3% and 0.5% of the dose present in the kidneys, liver and heart with corresponding tissue concentrations (wet weight) of 221 $\mu g\,g^{-1}$, 36 $\mu g\,g^{-1}$ and 20 $\mu g\,g^{-1}$ tissue respectively. Drug was undetectable in brain and red blood cells ($<2\,\mu g\,g^{-1}$). Three toxicities have been observed in male $BDF_1$ mice. *Bodyweight loss and leucopenia.* At a maximally tolerated dose (31 $mg\,kg^{-1}$) there was a 26% decrease in bodyweight (nadir on day 10) and pronounced leucopenia (87% decrease, nadir day 3). Platelet and red blood cell counts remained normal. *Convulsions.* At 48 $mg\,kg^{-1}$, convulsions were universally lethal (4/4 dead). The $LD_{50}$ was 41 $mg\,kg^{-1}$ (95% limits 36–46 $mg\,kg^{-1}$). 50 $mg\,kg^{-1}$ mitozantrone did not induce convulsions but was a lethal dose in 4/4 mice (deaths on days 5–6). The anticonvulsants phenytoin (100 $mg\,kg^{-1}$), phenobarbital (30 $mg\,kg^{-1}$) and valproic acid (300 $mg\,kg^{-1}$) administered 1 h prior to CI 941 did not prevent this effect. CI 941-induced convulsions are not due to cardiac arrest. The acute onset of convulsions and the relatively high levels of the drug found in the kidneys following administration may be important determinants in defining the dose limiting toxicity of this agent in man.

### Autoradiographic analysis of the intra-renal localisation of *cis*-platin

C. Ewen[1], J.H. Hendry[1], H. Sharma[2], A. Perara[3] & C.A. McAuliffe[3]

[1]*Department of Radiobiology, Paterson Laboratories,* [2]*Department of Medical Biophysics, Manchester University,* [3]*Department of Chemistry, UMIST, Manchester, UK.*

Knowledge of the dose of *cis*-platin or its metabolites received by the target cell populations within the kidney are essential to an understanding of dose-response relationships for long term renal injury.

In order to obtain precise information on the *in situ* renal localisation of this drug, autoradiography was performed, following administration of Pt-195m labelled *cis*-platin. $BDF_1$ mice were given 10 $mg\,kg^{-1}$ *cis*-platin i.v., and at predetermined times five animals per time point were sacrificed. The kidneys were removed and fixed for autoradiography. Slides were dipped in ILFORD K5 emulsion and exposed for four days. Following development the slides were stained with celestine blue.

Microscopic examination showed widespread distribution of label. Initially, high concentrations were seen in the renal pelvis, however at later times microdensitometric analysis showed retention of more label within the cortex than in the medulla. Autoradiographs were prepared at various times up to 2 weeks after drug administration. These showed that the label had virtually disappeared from the glomerulus, but it was retained by the tubule epithelium. There appeared to be no marked difference in the retention of platinum along the tubule. This is now being quantified in detail.

### Lack of relationship between plasma and tumour levels of thioTEPA and its metabolite tepa on the response of murine tumours

R.M. Phillips[1], M.C. Bibby[1], J.A. Double[1] & B.J. McDermott[2]

[1]*Clinical Oncology Unit, University of Bradford, Bradford BD7 1DP and* [2]*Department of Therapeutics & Pharmacology, Queen's University, Belfast, BT9 7BL, UK.*

The cytotoxic drug ThioTEPA is being used currently in the treatment of ovarian and breast cancer (Turner, *Int. Congr. Ser., Excerpta Med.*, **9**, 1, 1981). Responses are variable and may correlate with pharmacokinetic parameters. Objective assessment of clinical response is lengthy and difficult but can be easily accomplished in an experimental system. Three mouse colon tumours of varying growth characteristics and histology (MAC tumours) and the P388, L1210 have been used to examine the relationship between plasma and tumour levels of drug and metabolite and sensitivity. MAC 26, L1210 and P388 were the most sensitive tumours with MAC 13 being moderately responsive and the ascitic tumour MAC 15A showing no response, even when grown subcutaneously. Plasma levels of drug and metabolite determined by GC (McDermott *et al., J. Chromatog.*, **338**, 335, 1985) 60 min after treatment with 20 $mg\,kg^{-1}$ were similar in MAC 13 and MAC 26 but significantly higher in P388, L1210 and MAC 15A. Subcutaneous tumour concentrations of drug and metabolite were similar in MAC 13, MAC 15A and MAC 26 but significantly lower in the ascitic MAC 15A and not detected in P388 and L1210. Significant levels of both drug and metabolite in peritoneal fluid from MAC 15A indicate inherent resistance rather than availability may be responsible for insensitivity. This observation has been confirmed *in vitro*. These studies have indicated the levels of drug and metabolite required to achieve responses and that limited pharmacokinetic studies alone are insufficient to predict response. However in a corresponding clinical situation if it were possible to determine *in vitro* sensitivity then a knowledge of pharmacokinetic parameters would provide a valuable guide to predicting the outcome of therapy.

**Photodynamic therapy of cancer: Potential use of phthalocyanines as tumour localising photosensitisers**

W.-S. Chan, J. Marshall & I.R. Hart

*ICRF Laboratories, Lincoln's Inn Fields, London WC2A 3PX, UK.*

The present study was undertaken to evaluate the suitability of aluminium chloro-sulphonated phthalocyanine (AlSPc) as a photosensitiser for use in photodynamic therapy (PDT) of cancer. AlSPc absorbs red light strongly, is taken up *in vitro* by cells in a dose dependent fashion and the treated cells remain viable following exposure to normal room light. In contrast, exposure to red light (~600–700 nm) for 30 min produced 100% cytotoxicity in UV-2237 firbosarcoma cells as evaluated 3 days later. The uptake and retention of AlSPc has been examined using three murine tumours of different histological origin growing in the subcutaneous flank regions of syngeneic hosts. At various times after i.v. injection of the dye mice were killed (3 per time point per tumour), organs were recovered and weighed. Organ associated dye was extracted and determined by measuring fluorescence (excitation 603 nm; emission 673 nm) and expressed as fluorescence unit per gram of tissue. By 24–48 h after i.v. injection all three tumour types, the M5076 of macrophage origin, the UV-2237 fibrosarcoma and the colorectal Colo 26, showed significant retention of AlSPc compared to many normal tissues. These preliminary results suggest that the phthalocyanine compound AlSPc has considerable potential for use as a photosensitizer in PDT of cancer.

**Evidence for the formation of a reactive metabolite of the investigational antitumour drug N-methylformamide**

P. Kestell[1], P.B. Farmer[2], A. Gescher[1], A.P. Gledhill[1] & M.D. Threadgill[1]

[1]*CRC Experimental Chemotherapy Group, Pharmaceutical Sciences Institute, Aston University, Birmingham and* [2]*MRC Toxicology Unit, Carshalton, UK.*

Clinical trials and animal experiments have shown that the investigational antitumour drug N-methylformamide (NMF) is a hepatotoxin, but completely lacks myelotoxic properties. NMF is metabolised to a compound which is bound covalently to hepatic proteins *in vitro* and *in vivo* (Pearson, Harpur and Gescher, unpublished). NMF also depletes hepatic glutathione (Gescher *et al., Br. J. Cancer,* **45,** 843, 1982). This indicates that an electrophilic, possibly cytotoxic metabolite is formed. We have investigated the metabolites of NMF in the urine of patients who had received NMF in a phase I clinical trial (McVie *et al., Cancer Treat. Rep.,* **68,** 607, 1984) in order to get further evidence to support this hypothesis. After freeze drying, a metabolite was isolated by preparative TLC, which had acidic properties and contained sulphur. Esterification with methanolic hydrogen chloride afforded a compound which gave the following fragments on chemical ionisation mass spectrometry: m/z 235 ($M^+ + H$), 178, 136 and 60. The spectrum was identical with that of the methyl ester of authentic S-(N-methylcarbamoyl)N-acetylcysteine. This identification was supported by high-field NMR spectroscopy. It is possible and congruent with other evidence that this metabolite is the product of the hepatic reaction of a reactive NMF oxidation product, such as methylisocyanate, and glutathione. Furthermore N-alkylmonothiocarbamates such as this metabolite are electrophiles and carbamoylating agents in their own right.

**Pharmacokinetics of oral weekly Idarubicin (4DMDNR)**

D.B. Smith[1], J. Margison[2], P.M. Wilkinson[2], A. Howell[1] & G. Thomas[1]

[1]*CRC Department of Medical Oncology and* [2]*Department of Clinical Pharmacology, Christie Hospital & Holt Radium Institute, Manchester, and* [3]*Farmitalia Ltd., UK.*

Following oral or i.v. administration Idarubicin (Ida) is rapidly metabolised to 13-OH 4DMDNR. This metabolite is of equal antitumour activity to the parent drug in animal systems and is still present in the serum 7 days after dosing. The purpose of this study was to determine whether the pharmacokinetics of oral weekly Ida altered significantly with prolonged administration. Ida and 13-OH 4DMDNR concentrations were measured using reverse phase HPLC with fluorescence detection giving a sensitivity of $0.05\,ng\,ml^{-1}$. Ida was administered orally at a dose of $15\,mg\,m^2\,week^{-1}$ to 26 patients with advanced breast cancer. Serum profiles were measured at weeks 1, 4, 12 and 24 in 4 patients, at weeks 1 and 4 in 4 patients and at week 1 only in 3 patients. In an additional 5 patients the serum profile following an oral dose was compared with that following the same dose i.v. Seven day 13-OH 4DMDNR levels were measured at each clinic visit in 26 parents. After an oral dose of Ida 15 mg m$^{-2}$ the mean peak serum concentration was 3.33 ng ml$^{-1}$ $\pm$2.10 occurring 2.82 h $\pm$ 1.53 following ingestion. The mean peak 13-OH 4DMDNR concentration was 7.69 ng ml$^{-1}$ $\pm$5.19 occurring 3.46 h $\pm$ 1.84 after dosing. Mean AUC (Ida) after i.v. administration was 248 $\mu$g l$^{-1}$ h$^{-1}$ $\pm$94.64 and after oral was 75.3 $\mu$g l$^{-1}$ h$^{-1}$ $\pm$59.38 indicating oral availability of 26%. In patients studied for 24 weeks the mean AUC for Ida was 50.75 $\pm$ 7.59 $\mu$g l$^{-1}$ h$^{-1}$ at the start of treatment and 56.0 $\pm$ 9.37 $\mu$g l$^{-1}$ h$^{-1}$ at 6 months. The corresponding values of AUC for 13-OH 4DMDNR were 251.3 $\mu$g l$^{-1}$ h$^{-1}$ $\pm$117.2 and 274.3 $\mu$g l$^{-1}$ h$^{-1}$ $\pm$34.8. The mean 7 day level of the metabolite was 0.8 ng ml$^{-1}$ $\pm$0.55 and there was no indication of accumulation. The pharmacokinetics of Ida do not appear to alter with continuous oral weekly treatment.

### Bioavailability and tolerance of oral ifosfamide in patients with bronchial carcinoma

T. Cerny[1], J. Margison[2], N. Thatcher[1] & P.M. Wilkinson[2]

[1]*Department of Medical Oncology and* [2]*Department of Clinical Pharmacology, Christie Hospital, Manchester M20 9BX, UK.*

The aim of this study was to assess the bioavailability and tolerance of oral Ifosfamide (I) at different dose levels. Pharmacokinetics of (I) were determined in patients with bronchial carcinoma following bolus dose administration of 1 g and 2 g p.o. and i.v. in 7 patients and 5 g p.o. in 3 patients. Serial serum and urine samples were collected during the first 48 h after administration and concentration of (I) were assayed by HPLC using a method developed in this laboratory. The AUC following 1 and 2 g doses was the same for both i.v. and oral treatment and increased linearly after 5 g oral (I) indicating a 100% bioavailability (Table). The terminal half life was decreased with increasing dose but drug clearance was similar. The side effects of 1 and 2 g oral (I) were mild (slight nausea was noted in 4 of 26 courses and self limiting episodes of vomiting in 2 of 26 courses) whereas 2 of 3 patients showed signs of CNS toxicity and severe vomiting after 5 g oral (I). We conclude that 1 and 2 g oral (I) were well tolerated but 5 g oral (I) may not be compatible with a simple oral treatment.

Bioavailability and T½ terminal of Ifosfamide

| | *1g* | | *2g* | | *5g* |
|---|---|---|---|---|---|
| | *p.o.* | *i.v.* | *p.o.* | *i.v.* | *p.o.* |
| AUC ($\mu$g h l$^{-1}$) | 266.3 | 294.2 | 511.8 | 478.2 | 1,229.7 |
| s.d. | 12.0 | 29.8 | 83.9 | 34.8 | 156.2 |
| s.e. | 4.9 | 12.1 | 31.7 | 13.7 | 90.1 |
| T½ terminal (h$^{-1}$) | 5.3 | 5.9 | 5.3 | 5.29 | 4.07 |

### A study of a combination of two hypoxic cell radiosensitisers, Ro 03-8799 and SR 2508: Clinical toxicity and pharmacology

H.F.V. Newman, N.M. Bleehen & P. Workman

*MRC Clinical Oncology and Radiotherapeutics Unit, MRC Centre, Hills Road, Cambridge, UK.*

The hydrophilic hypoxic cell radiosensitiser SR 2508 causes peripheral neuropathy at total doses of >30 g m$^{-2}$. The basic analogue, Ro 03-8799, produces a transient syndrome of sweating, dizziness and affective changes, but no other toxicity. By combining tolerable doses of each, it may be possible to increase efficacy without increasing toxicity. In an escalating single-dose study, the drugs were infused together i.v. in 50 ml saline over 10 min, beginning at 0.5 g m$^{-2}$ of each agent, and proceeding to 0.75 g m$^{-2}$ Ro 03-8799 with 0.5, 1.0, 1.5, 2.0 and 3 g m$^{-2}$ SR 2508. Two patients were treated at each dose level. Four patients experienced the known Ro 03-8799 associated syndrome, but the severity did not vary with increasing dose of SR 2508, and no other toxicity was seen. Plasma and urine pharmacokinetic studies showed that no drug interaction occurred. Mean $t\frac{1}{2}\beta$ values were 5.15 h and 5.36 h for Ro 03-8799 and SR 2508 respectively. Subsequently, a 9 dose regimen over a 3 week period has been given to 8 patients, using 0.75 g m$^{-2}$ Ro 03-8799 with escalating doses of 0.5, 1.0, 1.5 and 2.0 g m$^{-2}$ SR 2508. All patients exhibited mild to moderate toxicity from Ro 03-8799. No other toxicity was seen. Plasma pharmacokinetics showed no change between 1st and 9th infusions. Biopsies of accessible tumours following radiosensitiser administration were taken from 11 patients. Mean tumour concentrations showed over the 30 min following infusion were 30 and 72 $\mu$g g$^{-1}$ for Ro 03-8799 and SR 2508 respectively. The predicted single-dose sensitiser enhancement ratio would be 1.5–1.6, representing a useful gain over either agent used alone. Escalation of dose and number of doses is now in progress.

### Bleomycin pulmonary toxicity

P. Goddard[1], S. Goodman[2] & J. Bell[1]

[1]*Department of Radiodiagnosis, Bristol Royal Infirmary and* [2]*Bristol Radiotherapy Centre, UK.*

Twenty men with testicular tumours treated with bleomycin underwent serial chest radiographs and computed tomography (CT scans). These were then analysed to look for changes in the lung due to bleomycin.

The films were assessed by two radiologists. The presence or absence of interstitial fibrosis, linear pulmonary opacities, a sub-pleural line and pleural opacities was noted.

CT showed changes with a much greater sensitivity than chest radiographs. It showed interstitial fibrosis in 12, only 3 of whom had plain radiographic changes. In all but 1 changes were maximal in the lower lobes. In 7 patients CT showed a sub-pleural line. The other abnormalities detected by CT were linear pulmonary opacities in 5 patients, pleural thickening or pleural plaques in 4 and pulmonary metastatic deposits in 2.

The non-metastatic CT changes were similar to those seen in fibrosing alveolitis, rheumatoid lung and asbestosis. Rapid progression was seen over a series of CT scans in some cases.

CT changes occur in some cases before the patient complains of any respiratory symptoms and before plain radiographic changes are evident.

Of 4 patients so far scanned with gallium citrate 2 have had uptake in the lungs, indicating an active inflammatory process.

### Diagnostic imaging of post-irradiation changes in the chest

J. Bell[1], J.A. Bullimore[2], E.R. Davies[1], J. Hill[2] & P.R. Goddard[1]

[1]*Department of Radiodiagnosis, Bristol Royal Infirmary and* [2]*Bristol RTC, UK.*

Chest radiographs and CT scans of 20 patients who had undergone thoracic irradiation were reviewed. A further 19 patients undergoing irradiation of the thorax were studied prospectively with ventilation-perfusion lung scanning with single photo emission computed tomography (SPECT), in addition to plain radiography and CT.

CT was more sensitive than plain radiography at detecting post-irradiation changes in the lung. In some cases it is also more specfic, showing areas of pulmonary opacification to have a straight edge corresponding to the edge of the irradiation field, when such an edge is invisible on plain radiography owing to its obliquity. Shrinkage of pulmonary vessels in areas outside the irradiation field is also seen.

Ventilation-perfusion scanning shows that defects occur in long perfusion which may be partially matched by ventilation defects. Defects occur both within the irradiation field and outside it. SPECT imaging of perfusion scans show defects with a greater sensitivity than planar scans. Defects in the irradiation field are matched by CT opacification, those outside the field may be matched by areas of vessel shrinkage. Perfusion defects outside the irradiation field appear related to irradiation of the hilum.

### A combined biochemical-histological approach to carbohydrate changes in colonic mucus glycoproteins during malignant transformation

A.P. Corfield, R. Roe, S.A. Wagner, J.R. Clamp & R.C.N. Williamson

*Department of Medicine and Surgery, Bristol Royal Infirmary, Bristol, UK.*

Changes in mucus glycoprotein histochemistry have been described in both human and rat colonic cancer. However, limited biochemical information is available concerning these carbohydrate changes. Recently, alterations have been detected during human colonic malignant transformation using peanut lectin, reportedly specific for detection of the 'T'-antigen (galactosyl-$\beta$(1-3)N-acetyl-galactosamine), and polyclonal anti-'T' antibodies. Interpretation of binding by these ligands is however difficult. The use of mono-specific reagents towards the 'T'-antigen and enzymic analysis of its formation and blocking may clarify these anomalies.

We have developed a methodology to evaluate specific mucus carbohydrate changes during experimental colonic carcinogenesis in rats. This involves transfer of galactose, N-acetylglucosamine and sialic acid to specific acceptors containing 'Tn'-antigen (N-acetylgalactosamine-) or 'T'-antigen. Total glycosyltransferase activity and the nature of the products are determined using radioactive nucleotide sugars and tritiated acceptors respectively. Tritiated products are isolated after alkali $\beta$-elimination and characterised.

Parallel immunohistological studies using both lectins (including peanut and Limax Flavus) and monoclonal antibodies directed against 'Tn' and 'T' antigens are also carried out.

This novel approach enables us to detect and locate specific mucus glycoprotein changes during colonic carcinogenesis and to determine the precise nature of enzymic modifications. These studies are continuing in human colonic cancer.